# Plasma circRNA microarray profiling identifies novel circRNA biomarkers for the diagnosis of ovarian cancer

**DOI:** 10.1186/s13048-022-00988-0

**Published:** 2022-05-12

**Authors:** Lili Ge, Yu Sun, Yaqian Shi, Guangquan Liu, Fang Teng, Zhe Geng, Xiyi Chen, Hanzi Xu, Juan Xu, Xuemei Jia

**Affiliations:** 1 Department of Gynecology, Women’s Hospital of Nanjing Medical University (Nanjing Maternity and Child Health Care Hospital), 123 Mochou Rd, 210004 Nanjing, China; 2 Department of Radiation Oncology, The Affiliated Cancer Hospital of Nanjing Medical University & Jiangsu Cancer Hospital & Jiangsu Institute of Cancer Research, 210009 Nanjing, China

**Keywords:** Ovarian cancer, circRNA, Diagnostic biomarker, Plasma

## Abstract

**Background:**

Circular RNA (circRNA), a class of RNA with a covalent closed circular structure that widely existed in serum and plasma, has been considered an ideal liquid biopsy marker in many diseases. In this study, we employed microarray and qRT-PCR to evaluate the potential circulating circRNAs with diagnostic efficacy in ovarian cancer.

**Methods:**

We used microarray to explore the circRNA expression profile in ovarian cancer patients’ plasma and quantitative real-time (qRT)-PCR approach to assessing the candidate circRNA’s expression. Then the receiver operating characteristic (ROC) curve was employed to analyze the diagnostic values of candidate circRNAs. The diagnostic model circCOMBO was a combination of hsa_circ_0003972 and hsa_circ_0007288 built by binary logistic regression. Then bioinformatic tools were used to predict their potential mechanisms.

**Results:**

Hsa_circ_0003972 and hsa_circ_0007288 were downregulated in ovarian cancer patients’ plasma, tissues, and cell lines, comparing with the controls. Hsa_circ_0003972 and hsa_circ_0007288 exhibited diagnostic values with the Area Under Curve (AUC) of 0.724 and 0.790, respectively. circCOMBO showed a better diagnostic utility (AUC: 0.781), while the combination of circCOMBO and carbohydrate antigen 125 (CA125) showed the highest diagnostic value (AUC: 0.923). Furthermore, the higher expression level of hsa_circ_0007288 in both plasma and ovarian cancer tissues was associated with lower lymph node metastasis potential in ovarian cancer.

**Conclusions:**

Our results revealed that hsa_circ_0003972 and hsa_circ_0007288 may serve as novel circulating biomarkers for ovarian cancer diagnosis.

**Supplementary information:**

The online version contains supplementary material available at 10.1186/s13048-022-00988-0.

## Introduction

Ovarian cancer (OC) is the most malignant female tumor and is estimated to account for approximately 21,410 new cases and 13,770 deaths in 2021 in America [1]. The five-year survival rate of women diagnosed with early localized disease is over 90%, but drops precipitously when diagnosed at stages III or IV (30%) [1]. Notably, the five-year survival rate for stage I OC patients is as high as 92.6%, but only 16.3% of them would be diagnosed within that stage [1]. Therefore, discovering novel biomarkers for OC may be essential for improving the diagnosis and prognosis of OC patients.

At present, protein levels, such as carbohydrate antigen 125 (CA125), Human epididymis protein 4 (HE4), and the algorithms (Risk of Malignancy Index (RMI), and Risk of Ovarian Malignancy Algorithm (ROMA)) which combined CA125 and HE4, are widely applied in the diagnosis and prognosis of OC. However, the sensitivity and specificity of these approaches remain somewhat limited [2–5]. CircRNA, a newly discovered non-coding RNA generated by back-splicing [6], is highly stable (half-life > 48 h) and resistant to exonuclease-mediated RNA decay [7–9]. Its functions have been reported in various diseases, including cancer, where it could regulate cancer’s progression via sponging microRNAs (miRNAs), interacting with proteins, regulating transcription or being translation templates [10, 11]. Apart from dictating cancer fate, they are also promising biomarkers for cancer due to their abundance in blood, saliva, and exosomes [12–14].

With the development of high-throughput sequencing and microarray technologies, increasing studies showed that circulating circRNAs had diagnostic or prognostic value in many cancers [15, 16]. CircRNAs are differentially expressed in tumours with different clinical characteristics, suggesting that they may have potential prognostic values. Several investigations have confirmed their prognostic role in different cancers, including OC [17]. Decreased circ-ITCH expression was correlated with the poor prognosis of OC patients [18]. Reduced abundance of hsa_circ_0078607 showed parameters associated with poor prognosis, including advanced FIGO stage and higher serum CA125 level [19]. To date, several studies revealed the diagnostic role of circRNAs in cancer via lipid biopsies [15]. Serum circFoxO3a was downregulated in squamous cervical cancer and correlated with tumor invasion, lymph node metastasis, and poor prognosis [20]. The microarray-based approach has been employed to identify patterns of abnormal circRNA expression in plasma of breast cancer patients, and hsa_circ_0001785 was found as a potentially valuable diagnostic biomarker in breast cancer [21].

Currently, the diagnostic value of circulating circRNAs in OC remains unclear. In this study, we used circRNA microarray, illustrating the circulating circRNA expression profile in plasma of OC patients to find potential diagnostic plasma circRNA biomarkers for OC.

## Materials and methods

### Patients and samples

The plasma and tissue samples utilized in this study were collected between January 2020 and December 2020 from Women’s Hospital of Nanjing Medical University (Nanjing Maternity and Child Health Care Hospital). The clinical characteristics of the patients with plasma samples were shown in Table S1. Two experienced pathologists confirmed the diagnosis of OC through the clinicopathological analysis of surgical tissues. The age-matched benign control (47.9 ± 9.6 versus 52.5 ± 11.8 in OC group) samples were obtained from patients with benign diseases. Moreover, forty-one OC tissues and 15 adjacent tissues were obtained from OC patients (age: 53 ± 7.9 and 50 ± 14.1, respectively). All the plasma samples were collected before treatment, and the tissue samples were collected immediately after surgery. All the samples were stored at -80 °C before use. The Ethics Committee of Nanjing Maternity and Child Health Care Hospital has approved this study (Approval Number: 2021KY-040), and informed consent was obtained from all patients.

### Peripheral blood sample collection

Peripheral blood samples (2 ml) were obtained from preoperative patients and controls using BD Vacutainer tubes (New Jersey, USA). Peripheral blood was centrifuged at 3000 rpm for 10 min. The isolated plasma was preserved in the 1.5 mL nuclease-free EP tube and stored at -80℃ until use.

### RNA extraction, reverse transcription and quantitative real-time polymerase chain reaction (qRT-PCR)

Total RNA from patients’ plasma was extracted using QIAGEN miRNeasy Serum/Plasma Advanced Kit (QIAGEN, Dusseldorf, Germany) according to the manufacturer’s instructions. And total RNA from patients’ tissue was extracted using GENEJET RNA purification kit (Fermentas, Lithuanin, USA) according to manufacturer’s instructions. RNA was reverse transcribed into complementary DNA (cDNA) in a reaction volume of 20 µL using Revert Aid First Strand cDNA Synthesis Kit (Thermo Fisher Scientific, Waltham, USA). qRT-PCR was performed using AceQ Universal SYBR qPCR Master Mix (Vazyme, Nanjing, China) on Applied Biosystems ABI Viia7 (Thermo Fisher Scientific, Waltham, USA). The sequences of the primers used in the PCR assay were listed in Table S2. The levels of circRNAs were calculated using 2^−∆Ct^ method and GAPDH was used as a control.

### RNase R treatment and Sanger sequencing

A total of 3 µg RNA was treated with RNase R as previously described [22]. The control group was treated with nuclease-free water. Then the RNase R treated RNA and control RNA were subjected to cDNA synthesis with random primers according to the instructions for the RevertAid First Strand cDNA Synthesis Kit (Thermo Fisher Scientific, Waltham, USA). The PCR products of the circRNAs were analyzed by sanger sequencing and examined by agarose electrophoresis at 120 V for 20 min.

### ceRNA microarray

The SBC Human (4 × 180 K) ceRNA array V1.0 (Shanghai Biotechnology Corporation, Shanghai, China) was used to analyze the expression of circRNA. RNAs were extracted from the plasma of four OC patients and four uterine myoma patients as controls by using Serum/Plasma Kit (QIAGEN, Dusseldorf, Germany). Then, NanoDrop ND-1000 instrument (Thermo, Waltham, MA, USA) and Bioanalyzer 2100 (Agilent, Santa Clara, CA, USA) were performed to detect the purity and integrity of the RNA. Total RNAs were amplified and labeled using Low Input Quick Amp Labeling Kit, One-Color (Agilent, Santa Clara, CA, USA) according to the manufacturer’s instructions and the labeled complementary RNAs (cRNAs) were purified by RNeasy Mini Kit (QIAGEN, GmbH, Germany). The labeled cRNAs were hybridized using Gene Expression Hybridization Kit (Agilent, Santa Clara, CA, USA). The completed hybridizations were scanned by an Agilent Microarray Scanner (Agilent, Santa Clara, CA, USA), and the Dye channel was set by the software: Green, Scan resolution = 3 μm, PMT 100%, 20 bit. Feature Extraction Software 10.7 (Agilent, Santa Clara, CA, USA) was used to read the data. Finally, quantile normalization and subsequent data processing were performed using the R software package. The screening threshold was set as fold change > 2.0 or < 0.5, *P*-value < 0.05.

### Statistical analysis

SPSS 26.0 (SPSS, Inc., IL, USA) and GraphPad Prism 8.0 (GraphPad, Inc., CA, USA) were used for statistical analysis. Data were compared using Student’s t-tests and Man-Whitney tests, as appropriate. The Chi square test was employed to analyze associations between expression of plasma circRNAs and clinical characteristics. The circCOMBO diagnostic model was developed through binary logistic regression analyses. ROC curve analyses were used to determine optimal plasma circRNA expression cutoff values, so that diagnostic utility could be maximized after using SPSS 26.0 to generate ROC curves. The pROC package [23] in R Studio was used to perform DeLong’s test between two ROC curves. *P*-value < 0.05 was considered as statistically significant.

### The competing endogenous RNAs (ceRNA) network construction and the identification of hub genes

The miRNA binding sites of the circRNAs were predicted by circInteractome and circBank (Tables S3-S4) [24, 25]. Candidate miRNAs should meet the criteria: highly expressed in OC tissues comparing to normal ovarian tissues (fold change > 1.5, *P*-value < 0.05). The expression of selected miRNAs was downloaded from GSE47841 (Table S5) [26].

TargetScan [27] and the miRDB [28] database were used to predict the miRNA targeted messenger RNAs (mRNAs). We identified down regulated mRNAs in OC tissues in TCGA database compared with normal ovarian tissues in GTEx database (Table S6). Then the overlap between the downregulated mRNAs and the predicted target mRNAs were obtained, with 137 candidate mRNAs were obtained for further analysis (Table S7).

The Search Tool for the Retrieval of Interacting Genes (STRING) (v11.0) was used to predict the association between candidate miRNAs and mRNAs. A protein-protein interaction (PPI) network was constructed using Cytoscape v3.7.0. The hub genes were selected using the Maximal Clique Centrality (MCC) method. A circRNA-miRNA-hubgene (ceRNA) network was constructed using Cytoscape v3.7.0.

## Results

### CircRNA expression profile in plasma of OC and control patients

Firstly, circRNA microarray was used to investigate differently expressed circRNAs in the plasma of OC patients and benign individuals. A total of 46 circRNAs were upregulated (red spots), and 595 circRNAs were downregulated (green spots) in OC patients compared with the control (fold change > 2 or <0.5 and *P*-value < 0.05) (Fig. S1 and Table S8).

### Validation of candidate circRNA expression

To validate the microarray results, four candidate circRNAs (hsa_circ_0053221, hsa_circ_0062215, hsa_circ_0003972, hsa_circ_0007288) with the following criteria: (1) high normalized signal (2) fold-change > 2 or <0.5 and *P*-value < 0.01, were randomly selected. The head-to-tail splicing of these circRNAs was further analyzed by RNase R treatment and Sanger sequencing. All these four circRNAs were resistant to RNase, while their liner compartments were sensitive to RNase R (Fig S2a-d, left), which indicated the existence of these circRNAs. Compared with hsa_circ_0003972 and hsa_circ_0007288, the expression of hsa_circ_0053221 and hsa_circ_0062215 were relatively low, as shown in both the microarray data and the qRT-PCR results (Table S8 and Fig S2a-b, left). In addition, head-to-tail splicing was validated by sanger sequencing of the qRT-PCR products (Fig S2a-d, right). We next evaluated the expression of these circRNAs in plasma samples from 60 OC patients and 60 benign patients to validate the clinical relevance of these circRNAs. The results indicated that hsa_circ_0003972 and hsa_circ_0007288 were downregulated in the plasma of OC patients, comparing with benign controls, which were consistent with the microarray results (Fig S2e-f). Meanwhile, hsa_circ_0053221 and hsa_circ_0062215 were undetermined in more than half of the plasma samples, which led to unreliable results. Hence, we selected hsa_circ_0003972 and hsa_circ_0007288 for further analysis.

### Correlation between candidate circRNAs expression and clinical characteristics

Then we analyzed the correlation between the expression of these two circRNAs in the benign control group (uterine fibroids versus other benign diseases) and found that the expression levels of these two candidate circRNAs showed no significant difference between uterine fibroids and other benign diseases (Table S9), which could exclude the possible influence of the proliferative characteristic of uterine fibroids. The 60 OC patients were divided into two groups based on the median expression levels of circRNA: high (*n* = 30) and low (*n* = 30) groups. Then we assessed the correlation between plasma levels of these two candidate circRNAs and the clinical characteristics of OC patients (Table 1). The results showed that the expression of plasma hsa_circ_0007288 was only significantly associated with lymph node metastasis (*P* = 0.02). And the plasma level of hsa_circ_0003972 was not significantly associated with all the clinical characteristics we analyzed.

**Table 1:** The correlation between the expression of plasma circRNAs and clinical characteristics of ovarian cancer patients

Variables	circ-0003972		*P*	circ-0007288		*P*
	low expression	high expression		low expression	high expression	
Age			0.605			1
≤53	16	14		15	15	
>53	14	16		15	15	
Menopausal Stage			0.292			0.598
pre-M	14	10		13	11	
post-M	16	20		17	19	
Ovarian Cancer type			0.519			0.519
EOC	23	25		25	23	
others	7	5		5	7	
FIGO Stage			0.787			0.284
I-II	10	11		9	13	
III-IV	20	19		21	17	
Lymphatic metastasis			0.559			0.02
No	23	21		18	26	
Yes	7	9		12	4	
Distant metastasis			0.612			0.612
No	27	29		27	29	
Yes	3	1		3	1	
Histological grade			0.785			0.243
Low	7	9		10	6	
High	21	23		20	24	
CA125(U/mL)			1			0.559
≤35	8	8		9	7	
>35	22	22		21	23	

### The diagnostic performance of the two candidate circRNAs in OC

**Fig. 1:**
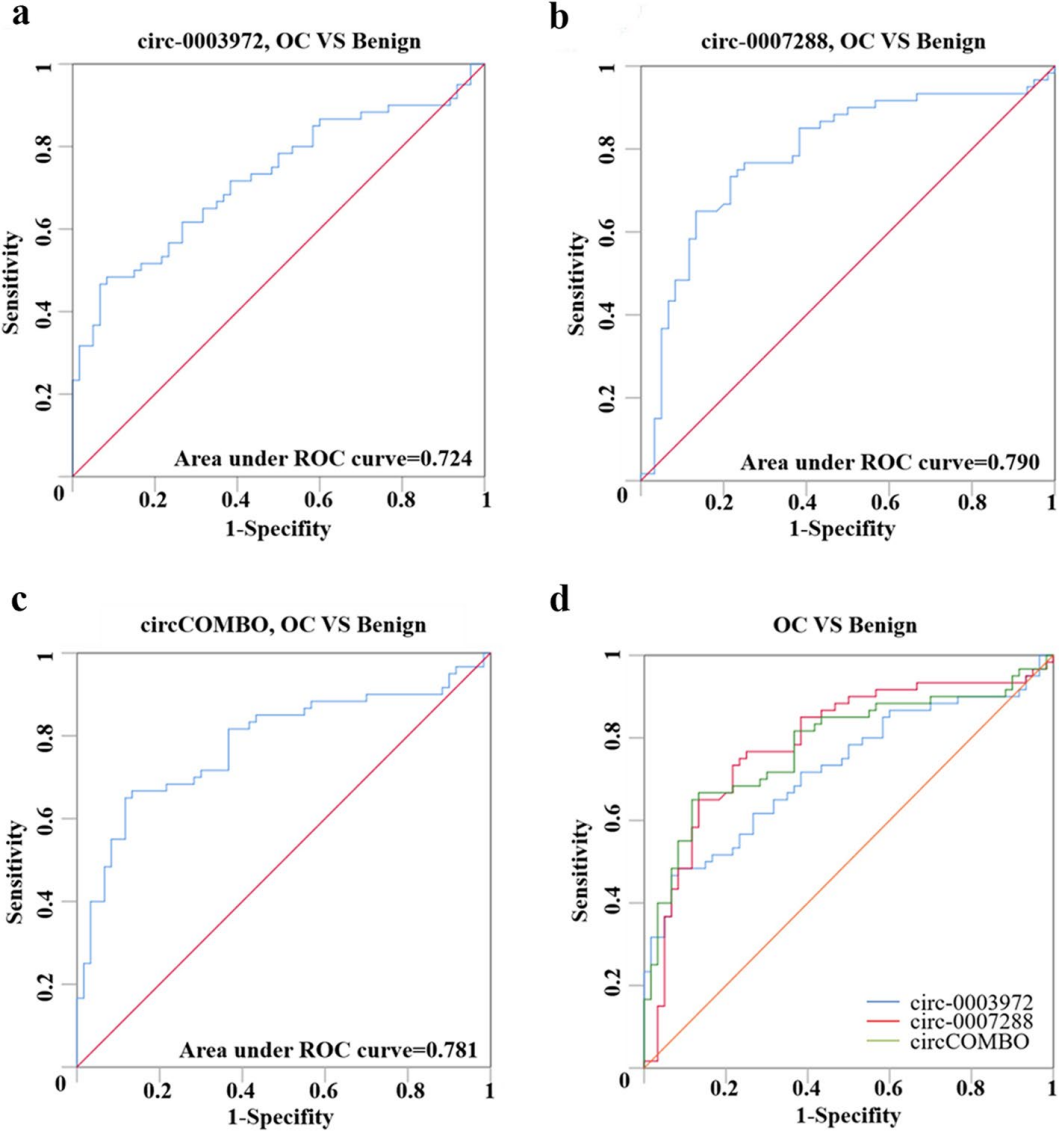
The diagnostic performance of circ_0003972, hsa_circ_0007288, and circCOMBO. **a** ROC curve analysis for the diagnostic performance of hsa_circ_0003972. **b** ROC curve analysis for the diagnostic performance of hsa_circ_0007288. **c** ROC curve analysis for the diagnostic performance of circCOMBO. **d** The diagnostic performance of hsa_circ_0003972, hsa_circ_0007288, and circCOMBO

Then, the ROC curve analysis was used to investigate the diagnostic values of these two candidate circRNAs. As shown in Fig. 1a-b; Table 1, the ROC curve showed that hsa_circ_0003972 had an AUC of 0.724 (95% CI: 0.632–0.815), and hsa_circ_0007288 had an AUC of 0.790 (95% CI: 0.705–0.875) in distinguishing OC patients from benign individuals. We next established a diagnostic model consisted of these two circRNAs (circCOMBO) using binary logistic regression analysis. The AUC, sensitivity, and specificity of circCOMBO in distinguishing OC and benign control patients were 0.781 (95% CI: 0.696–0.867), 66.7%, and 86.7%, respectively (Fig. 1c-d; Table 2). The Z test was used to compare the AUC of two circRNAs with that of circCOMBO. We found that the diagnostic utility of circCOMBO was better than that of hsa_circ_0003972, while there was no difference between hsa_circ_0003972 and hsa_circ_0007288, or between circCOMBO and hsa_circ_0007288 for the diagnosis of OC patients (Table 2).

**Table 2:** The performance of two candidate circRNAs and circCOMBO for the diagnosis of ovarian cancer

Groups	Best cut-off	AUC(95%CI)	Sensitivity(%)	Specificity(%)	*P*-value	Comparison of AUC	
						Groups	*P*-value
circ-0003972	0.26	0.724(0.632–0.815)	48	92	*p* < 0.0001	circ-0003972 vs. circ-0007288	0.2261
circ-0007288	0.011	0.790(0.705–0.875)	77	75	*p* < 0.0001	circ-0003972 vs. circCOMBO	0.0322
circCOMBO	0.45	0.781(0.696–0.867)	66.7	86.7	NA	circ-0007288 vs. circCOMBO	0.8210

### The diagnostic performance of circRNA and/or CA125 in the detection of OC

**Fig. 2:**
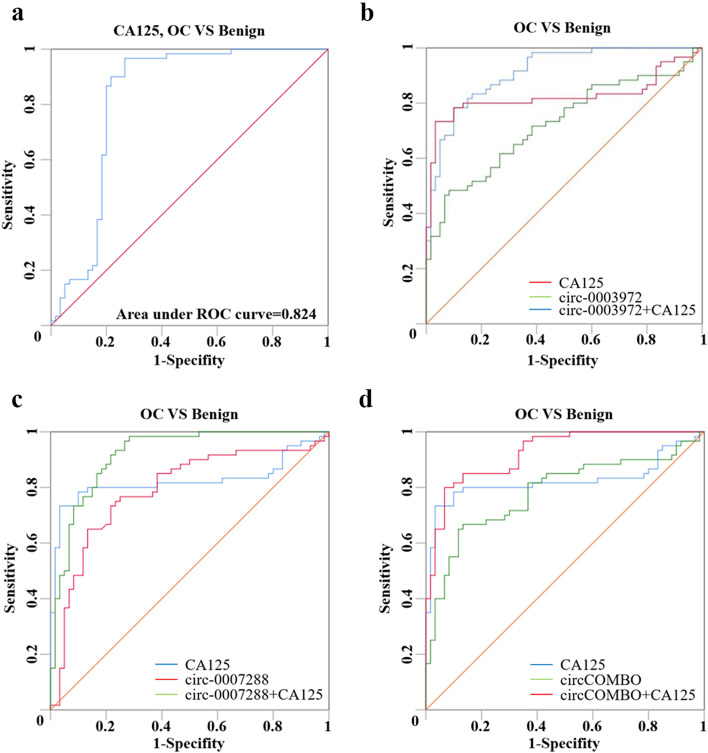
The diagnostic performance of CA125, hsa_circ_0003972 + CA125, hsa_circ_0007288 + CA125 and circCOMBO + CA125. **a** ROC curve analysis for the diagnostic performance of CA125. **b** ROC curve analysis for the diagnostic performance of CA125, hsa_circ_0003972, and hsa_circ_0003972 + CA125. **c** ROC curve analysis for the diagnostic performance of CA125, hsa_circ_0007288, and hsa_circ_0007288 + CA125. **d** ROC curve analysis for the diagnostic performance of CA125, circCOMBO, and circCOMBO + CA125

CA125 has been the most commonly used diagnostic marker of OC patients. Therefore, we further studied the combined diagnostic value of hsa_circ_0003972, hsa_circ_0007288, or circCOMBO with CA125(AUC: 0.824 (95%CI: 0.739–0.908)) in differentiating OC from benign controls. The results showed that the diagnostic performance of CA125 + hsa_circ_0007288, CA125 + hsa_circ_0003972, CA125 + circCOMBO were better than hsa_circ_0007288, hsa_circ_0003972, circCOMBO or CA125 alone, and CA125 + circCOMBO had the highest diagnostic value. However, there was no significant difference between CA125 + hsa_circ_0007288, CA125 + hsa_circ_0003972 and CA125 + circCOMBO (Fig. 2; Table 3 and Table S10).

**Table 3:** The diagnostic performance and the comparison between the diagnostic performance of CA125, hsa_circ_0003972 + CA125, hsa_circ_0007288 + CA125 and circCOMBO + CA125

	Best cut-off	AUC(95%CI)	Sensitivity (%)	Specificity (%)	*P*-value	Comparison of AUC	
						Groups	*P*-value
CA125	38.825U/ml	0.824(0.739–0.908)	97	73	*p* < 0.0001		
CA125 + circ-0003972	−	0.912(0.864–0.961)	78.3	90.0	NA	circ-0003972 vs. CA125	0.1201
						circ-0003972 vs. circ-0003972 + CA125	*p* < 0.0001
						CA125 vs. circ-0003972 + CA125	0.0781
CA125 + circ-0007288	−	0.918(0.869–0.968)	98.3	71.7	NA	circ-0007288 vs. CA125	0.5839
						circ-0007288 vs. circ-0007288 + CA125	0.0011
						CA125 vs. circ-0007288 + CA125	0.0613
CA125 + circCOMBO	−	0.923(0.878–0.968)	80.0	93.3	NA	circCOMBO vs. CA125	0.4947
						circCOMBO vs. circCOMBO + CA125	*p* < 0.0001
						CA125 vs. circCOMBO + CA125	0.0449

### Relative expression of circRNAs in OC tissues and cell lines

**Fig. 3:**
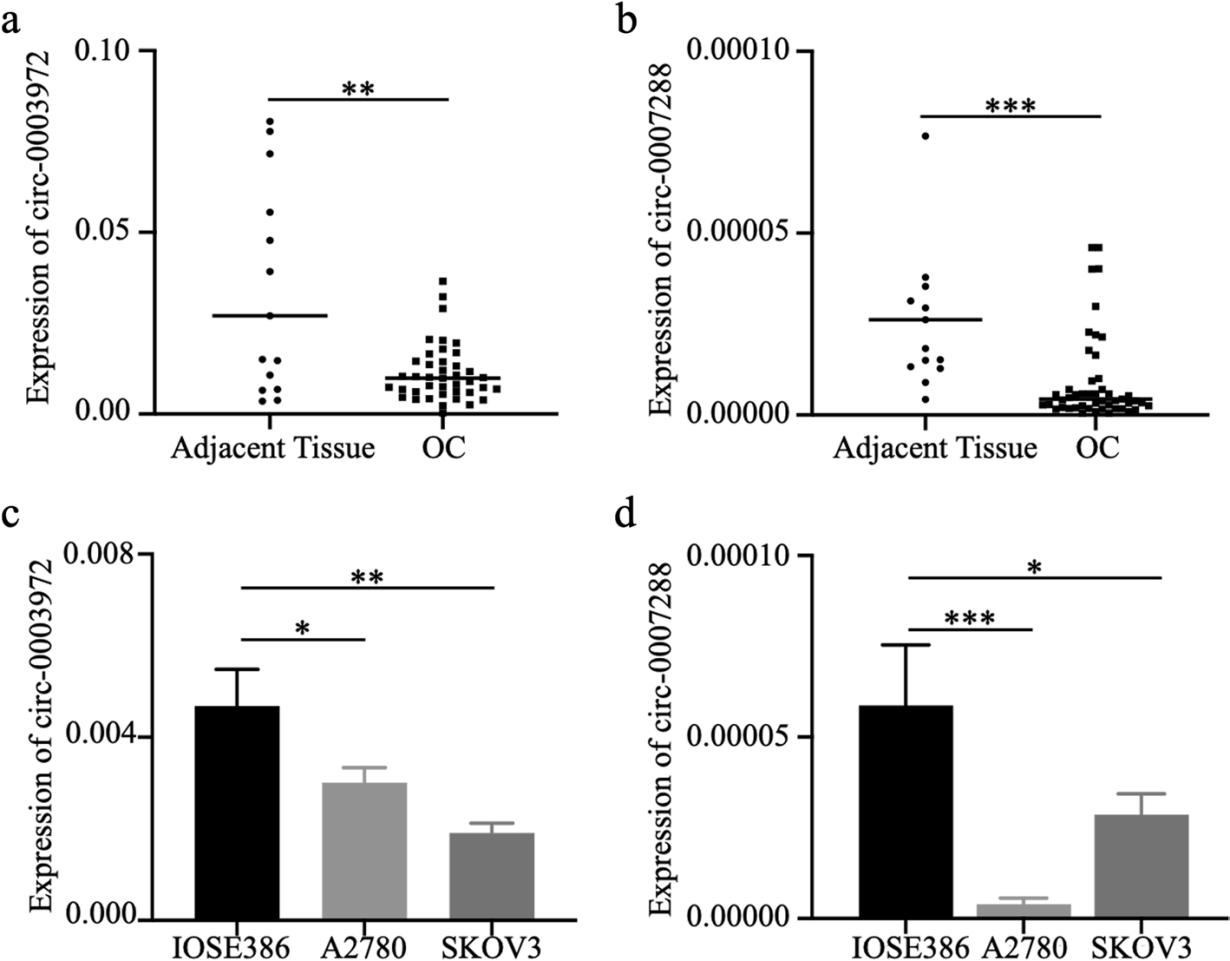
Expressions of hsa_circ_0003972 and hsa_circ_0007288 in OC cell lines and tissues. **a**-**b** qRT-PCR analysis of the expression of hsa_circ_0003972, hsa_circ_0007288 in adjacent tissues (*n* = 15) and OC tissues (*n* = 41). **c**-**d** qRT-PCR analysis of the expression of hsa_circ_0003972, hsa_circ_0007288 in IOSE386, A2780, and SKOV3 cell lines

We hypothesized that the two candidate circRNAs might also be involved in OC development since hsa_circ_0007288 was significantly associated with lymph node metastasis. To verify our presumptions, we used qRT-PCR to confirm the expression patterns of the two selected circRNAs in OC tissues, OC cell lines (A2780 and SKOV3), normal ovarian tissues, and normal ovarian epithelial cell line IOSE386, and found that both of these two circRNAs were downregulated in OC tissues (*n* = 41) and OC cell lines as compared with adjacent tissues (*n* = 15) (Fig. 3a-b) and IOSE386 (Fig. 3c-d), respectively. We also analyzed the correlation between the tissue levels of two circRNAs and the clinical characteristics of OC patients (Table 4). Interestingly, we found that the expression of hsa_circ_0007288 was negatively correlated with lymph node metastasis while hsa_circ_0003972 wasn’t related to any clinical characteristics, which is similar with the results in the plasma level.

**Table 4:** The correlation between the expression of tissue circRNAs and clinical characteristics of ovarian cancer patients

Variables	circ-0003972		*P*	circ-0007288		*P*
	low expression	high expression		low expression	high expression	
Age			0.155			0.867
≤53	9	13		10	9	
>53	12	7		11	11	
Menopausal Stage			0.153			0.91
pre-M	5	9		7	7	
post-M	16	11		14	13	
FIGO Stage			0.853			0.212
I,II	9	8		7	11	
III,IV	12	12		14	10	
Lymphatic metastasis			0.248			0.037
No	11	14		9	15	
Yes	10	6		12	5	
Distant metastasis			0.37			0.954
No	17	19		19	17	
Yes	4	1		2	3	
Histological grade			0.651			0.651
Low	4	6		4	6	
High	17	14		17	14	
CA125(U/mL)			0.954			0.954
≤35	2	3		2	3	
>35	19	17		19	17	

### Construction of the ceRNA network

As hsa_circ_0003972 and hsa_circ_0007288 were significantly downregulated in OC patients, we chose six miRNAs (hsa-miR-1228-3p, hsa-miR-1825, hsa-miR-183-5p (also named as hsa_miR_183-5p.1), hsa-miR-203, hsa-miR-421, hsa-miR-935) which harbored at least one binding site in either of these two circRNAs and were significantly upregulated in OC patients to construct the circRNA-miRNA-mRNA network. A total of 14,086 and 4239 target mRNAs of the six miRNAs were predicted by TargetScan and miRDB, respectively. Compared with normal ovarian tissues in GTEx database, 2919 mRNAs were downregulated in OC tissues in TCGA database. After taking intersections of these predicted and downregulated mRNAs, 137 mRNAs were selected as the targets of the six miRNAs for further analysis.

**Fig. 4:**
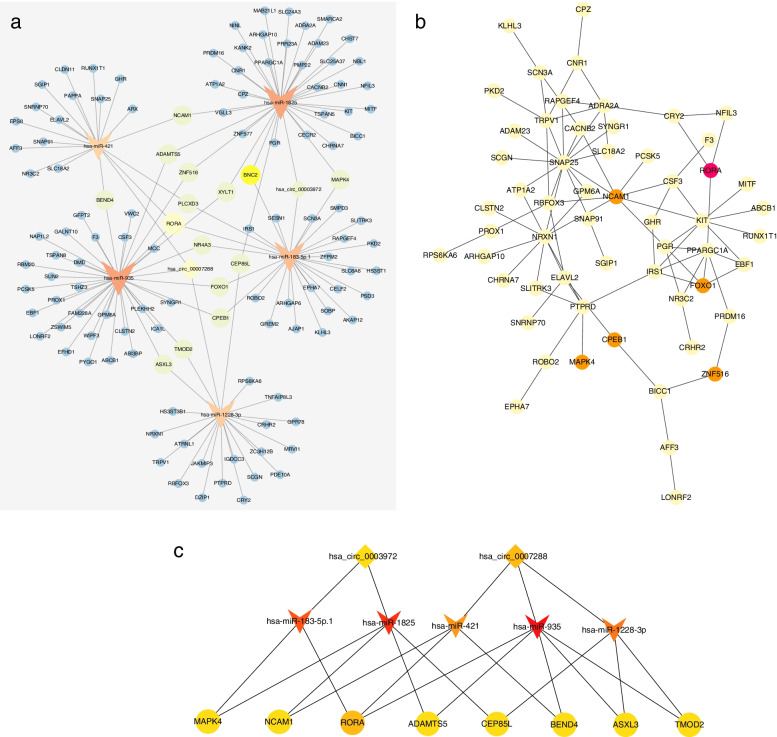
The circRNA-miRNA-hub gene network. **a** The circRNA-miRNA-mRNA network was constructed based on 2 circRNAs, 5 miRNAs, and 137 mRNAs. **b** Hub genes identified from the PPI network by cytoscape. **c** The circRNA-miRNA-hub gene network was constructed based on two circRNAs, five miRNAs, and eight hub genes

During the construction of the circRNA-miRNA-mRNA network, hsa-miR-203 failed to link with other miRNAs and mRNAs. Therefore, two circRNAs, five miRNAs, and 137 mRNAs were seen in the ceRNA network (Fig. 4a). We used the STRING database to build a PPI network composed of the 137 overlapping mRNAs (Fig. 4b). The hub genes were identified using the MCC method in Cytoscape, and the circRNA-miRNA-hub gene network was visualized by cytoscape (Fig. 4c).

## Discussion

CircRNA in cancer mainly functions through sponging miRNAs [29], interacting with proteins [30], or encoding peptides [31, 32]. Its high stability in body fluids and different expression in cancer made them ideal cancer biomarkers [13, 33–35]. It has been reported that plasma hsa_circ_0001785 could be a diagnostic biomarker for breast cancer [21], plasma hsa_circ_0005927, hsa_circ_0001900, hsa_circ_0001178 for colorectal cancer [36] and plasma circFoxO3a for cervical cancer [20]. However, only plasma circBNC2 (hsa_circ_0008732) has been reported as a diagnostic biomarker in OC patients [37, 38], and the circRNA expression profile in the plasma of OC patients has not been studied yet. Hence, we employed microarray to further identify the potential diagnostic circRNAs in the plasma of OC patients. Our results indicated that hsa_circ_0003972 and hsa_circ_0007288 may serve as important diagnostic biomarker of OC, while circBNC2 (hsa_circ_0008732), the known diagnostic biomarker in the plasma of OC patient, was not detected by microarray in our study and couldn’t be detected in most of our plasma samples by qRT-PCR, which indicated that its expression is very low, at least lower than hsa_circ_0003972 and hsa_circ_0007288. Furthermore, qRT-PCR indicated that its expression was comparable between ovarian cancer cell lines and normal ovarian epithelial cell lines (data not shown).

In this study, we also confirmed that the novel circRNAs: hsa_circ_0003972 and hsa_circ_0007288 are downregulated in both the OC tissues and the plasma of OC patients as compare with the adjacent normal ovarian tissues and the plasma of patients with benign disease, indicating that they may produce from normal tissues/cells and perform as the regulator of OC progression. And their down-regulation in the plasma or tissue levels of ovarian cancer patients was possibly caused by the suppression of the oncogenic RNA binding proteins, such as in DHX9 and ADAR1 [39–41]. CeRNAs are RNA transcripts that can regulate each other through miRNA response elements [29]. miRNAs have proven as robust biomarkers in gynecological cancers, including cervical cancer [42], endometrial cancer [43] as well as OC [44]. Increasing evidence has suggested that circRNAs may perform their functions by acting as ceRNAs [45]. Their interactions indicated approachable fields for early detection and prognosis of cancer [46]. Circular RNA ciRS-7 acted as a sponge of miR-7, upregulating several oncogenes (mTOR, EGFR, PIK3CD) to promote the progression of cancers in which high ciRS-7 expression correlated with worse prognosis and miR-7 acted as tumor suppressor [47–50]. Circular RNA itchy E3 ubiquitin protein ligase (circ-ITCH) has proven to be a suppressor in various cancers such as melanoma and OC [51]. Its high expression was an independent prognostic biomarker of OC. Meanwhile, it can also function by sponging miR-106a and thereby suppressing OC cell proliferation and invasion [18, 52]. Using bioinformatics analysis, we found that two differentially expressed circRNAs may sponge five different miRNAs. Previous studies have shown that miR-183-5p exerted an oncogenic function in breast cancers [53] and could be a prognostic biomarker of OC [54]. It has been reported that miR-421, miR-935, and miR-1825 promoted the progression of glioblastoma, osteosarcoma, and liver cancer, respectively [55–57], and high expression of serum miR-1228-3p was related to poor prognosis of non-small cell lung cancer [58].

We selected downregulated genes in TCGA OC database to establish a circRNA-miRNA-hub gene network, while MAPK4, NCAM1, RORA, ADAMTS5, CEP85L, BEND4, ASXL3, TMOD2 were considered as the hub genes. Interestingly, RORA, acting as a potent tumor suppressor, was downregulated in several cancers, such as breast cancer and colorectal cancer, which inhibited cancers’ growth through attenuating Wnt/beta-catenin signaling, inducing SEMA3F expression or stabilizing p53 to activate apoptosis [59–61]. ADAMTS5, a zinc metalloprotease, has been involved in many biological processes, including inflammation, angiogenesis, and tumorigenesis [62]. Overexpressed MAPK4 was correlated with poor survival in various cancers [63] and played an oncogenic role in prostate cancer [64]. NCAM1 helped cancer cell’s perineural invasion [65] and promoted leukemogenesis and drug resistance in acute myeloid leukemia [66]. However, no studies demonstrated the role of ASXL3, TMOD2, and CEP85L in cancer. Based on the above studies, we speculated that both hsa_circ_0003972 and hsa_circ_0007288 may also act as tumor suppressors in OC through sponging the oncogenic miRNAs, thereby regulating target genes’ expression. Indeed, hsa_circ_0003972 was also detected in PBMC of Rheumatoid Arthritis patients, and suppressing the expression of hsa_circ_0003972 could inhibit the progression of Rheumatoid Arthritis at least partially through anti-inflammatory effect [67–69]. Besides, hsa_circ_0003972 mainly exerted its function in Rheumatoid Arthritis as the miRNA sponger [68, 69]. However, the exact role of hsa_circ_0003972 and hsa_circ_0007288 in ovarian cancer progression should be illustrated by extensive *in vitro* and *in vivo* studies in the future.

Although the exact mechanisms of the two identified circRNAs in OC were unknown, our results helped to underline the potential mechanisms of their pathogenesis in OC. Besides, examination of the circRNA’s expression in tissues may also provide the clue to derivation of circRNAs and point out the potential systemic effect.

## Conclusions

Our research revealed that novel plasma circRNA hsa_circ_0003972 and hsa_circ_0007288, the combination of hsa_circ_0003972 and hsa_circ_0007288 (circCOMBO), the combination of circCOMBO and CA125 could serve as a novel circulating biomarker for OC diagnosis. Meanwhile, the combination of circCOMBO and CA125 showed the highest diagnostic performance. The lower plasma level of hsa_circ_0007288 could serve as a potential biomarker for OC lymph node metastasis. Besides, hsa_circ_0003972 and hsa_circ_0007288 may also become the potential therapeutic target of OC.

## Supplementary Information

**Additional file 1: Figure S1.** CircRNA expression profiles in OC patients and benign individuals. The expression profile detected by circRNA microarray assay was shown in volcano plot. Four OC patients and four benign individuals were enrolled. Red spots indicated upregulated circRNAs and green spots indicated downregulated circRNAs.

**Additional file 2: Figure S2.** Validation of the four candidate circRNAs. (a-d). (Left): The electrophoresis of the qRT-PCR product of circRNA and linear RNA treated with or without RNase R. (Right): Sanger sequencing of the RT-PCR product in the left, and the base in the red square represented the head-to-tail splicing sites. (e-f). qRT-PCR analysis of the expression of hsa_circ_0003972, hsa_circ_0007288 in 60 OC patients and 60 benign controls’ plasma.

**Additional file 3: Table S1.** Clinicopathological characteristics of the enrolled patients. **Table S2.** Primer sequences for real-time PCR or quantitative real-time PCR. **Table S3.** The miRNA target prediction software circInteratome and circbank predicted miRNAs which could bind hsa_circ_0003972. **Table S4.** The miRNA target prediction software circInteratome and circbank predicted miRNAs which could bind hsa_circ_0007288. **Table S5.** The expression profile of the miRNAs that were upregulated in OC tissues as compared with normal ovarian tissues downloaded from GSE47841. **Table S6.** The downregulated mRNAs in OC tissues in TCGA database compared with normal ovarian tissues in GTEx database. **Table S7.** The 137 candidate mRNAs expression pattern in OC tissues as compared with normal ovarian tissues. **Table S8.** The microarray results of the 46 upregulated circRNAs and 595 downregulated circRNAs in OC patients compared with the benign control (fold change > 2 and *P*-value < 0.05). **Table S9.** The expression difference of candidate circRNAs between patients with uterine myoma or other benign diseases. **Table S10.** Comparison of AUC in circ-0003972 + CA125, circ-0007288 + CA125, circCOMBO + CA125.

## Data Availability

All data generated or analyzed during this study are included in this published article [and its supplementary information files].

## Abbreviations

circRNA: Circular RNA
OC: Ovarian cancer
qRT-PCR: Quantitative real-time Polymerase Chain Reaction
ROC: Receiver operating characteristic
AUC: Area Under Curve
CA125: Carbohydrate antigen 125
HE4: Human epididymis protein 4
RMI: Risk of Malignancy Index
ROMA: Risk of Ovarian Malignancy Algorithm
cDNA: Complementary DNA
ceRNA: Competing endogenous RNAs
PPI: Protein-protein interaction
STRING: The Search Tool for the Retrieval of Interacting Genes
MCC: Maximal Clique Centrality
