# Supplementary figures and images for: Plasma circRNA microarray profiling identifies novel circRNA biomarkers for the diagnosis of ovarian cancer
Source: J Ovarian Res. 2022 May 12;15:58. doi: 10.1186/s13048-022-00988-0 (PMC9097182; doi:10.1186/s13048-022-00988-0)

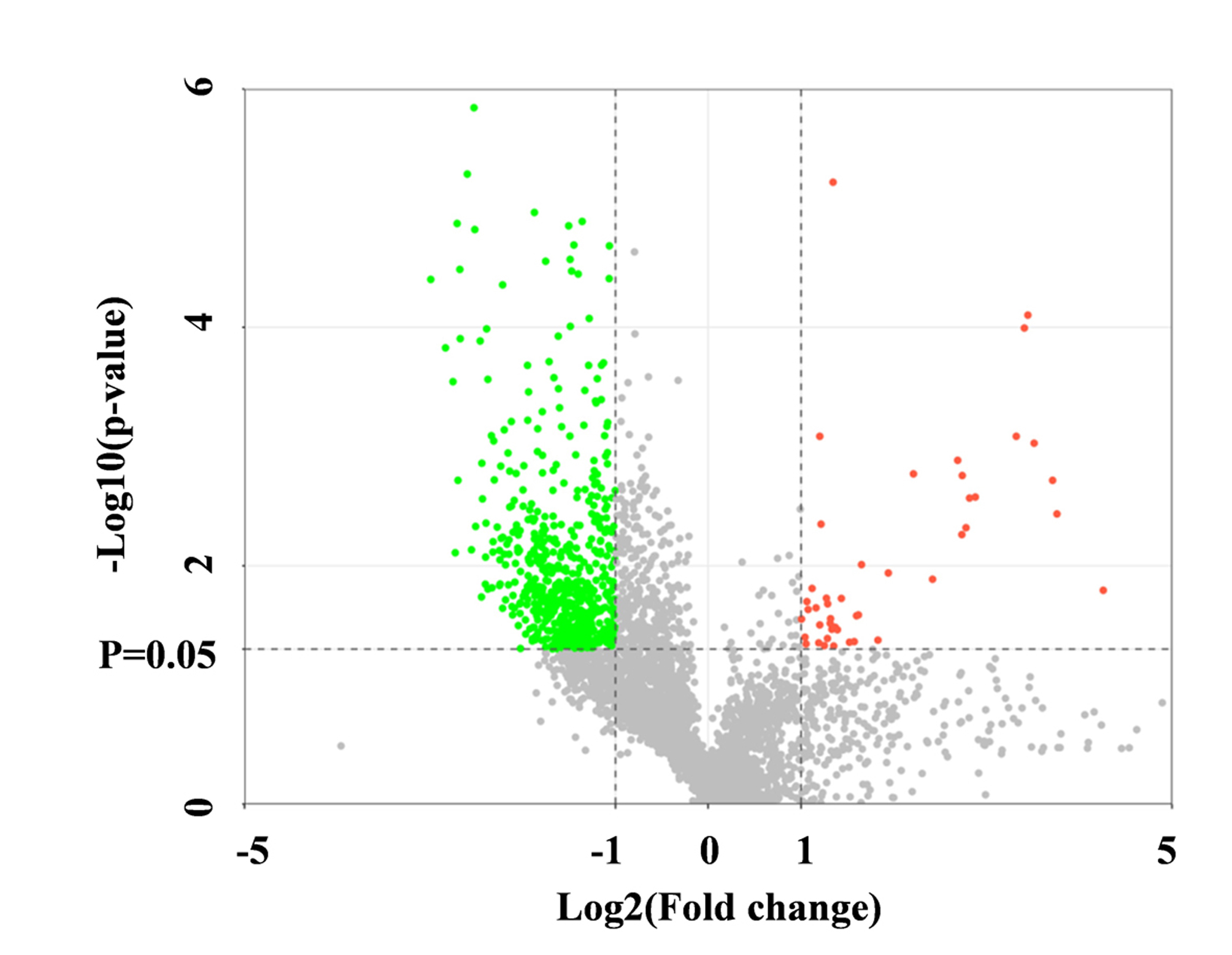

Supplement: Supplementary file 1 — Additional file 1: Figure S1. CircRNA expression profiles in OC patients and benign individuals. The expression profile detected by circRNA microarray assay was shown in volcano plot. Four OC patients and four benign individuals were enrolled. Red spots indicated upregulated circRNAs and green spots indicated downregulated circRNAs. [file 13048_2022_988_MOESM1_ESM.jpg]

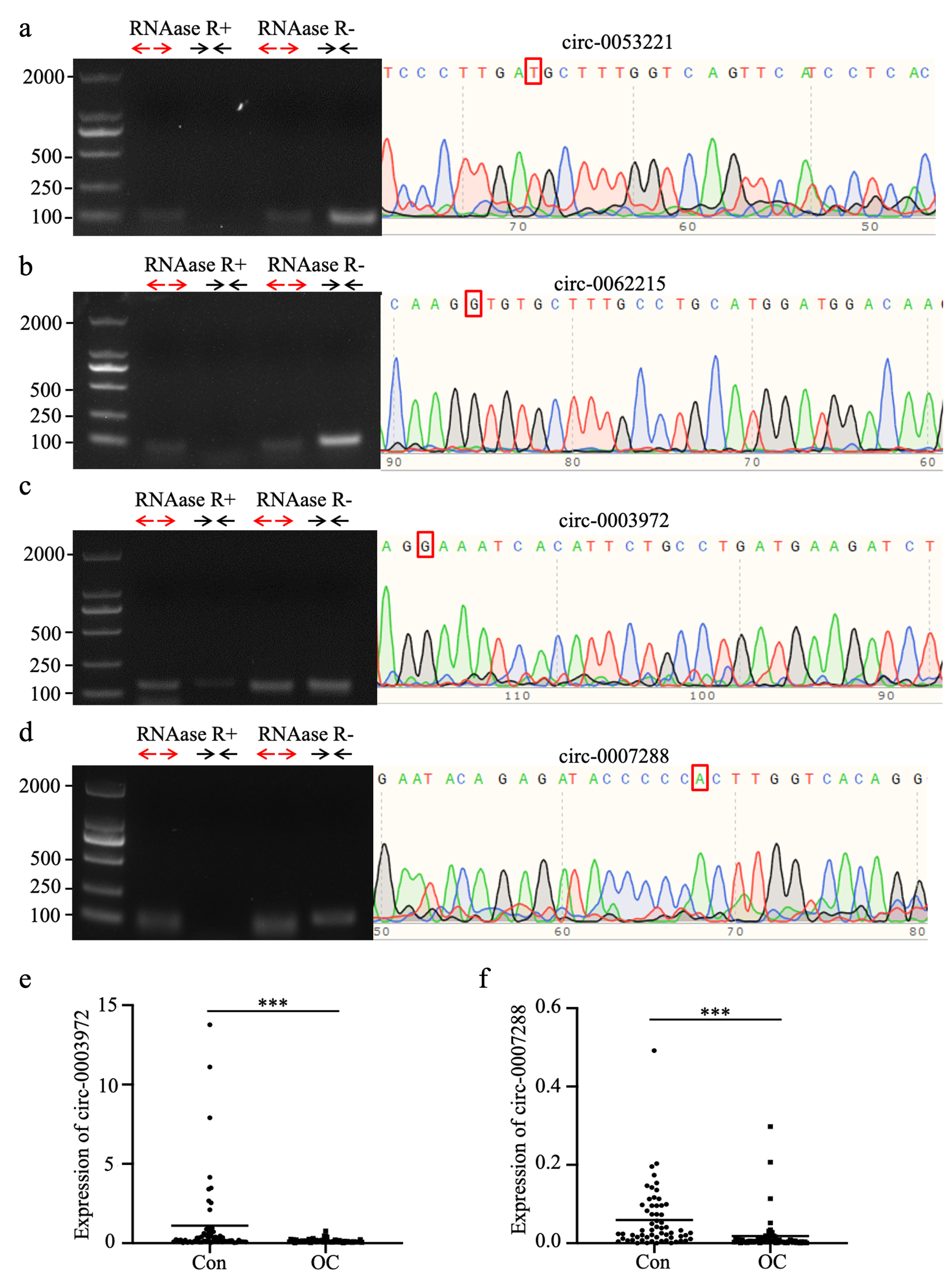

Supplement: Supplementary file 2 — Additional file 2: Figure S2. Validation of the four candidate circRNAs. (a-d). (Left): The electrophoresis of the qRT-PCR product of circRNA and linear RNA treated with or without RNase R. (Right): Sanger sequencing of the RT-PCR product in the left, and the base in the red square represented the head-to-tail splicing sites. (e-f). qRT-PCR analysis of the expression of hsa_circ_0003972, hsa_circ_0007288 in 60 OC patients and 60 benign controls’ plasma. [file 13048_2022_988_MOESM2_ESM.jpg]
