# Supplementary material for: Plasma circRNA microarray profiling identifies novel circRNA biomarkers for the diagnosis of ovarian cancer
Source: J Ovarian Res. 2022 May 12;15:58. doi: 10.1186/s13048-022-00988-0 (PMC9097182; doi:10.1186/s13048-022-00988-0)
Supplement: Supplementary file 3 — Additional file 3: Table S1. Clinicopathological characteristics of the enrolled patients. Table S2. Primer sequences for real-time PCR or quantitative real-time PCR. Table S3. The miRNA target prediction software circInteratome and circbank predicted miRNAs which could bind hsa_circ_0003972. Table S4. The miRNA target prediction software circInteratome and circbank predicted miRNAs which could bind hsa_circ_0007288. Table S5. The expression profile of the miRNAs that were upregulated in OC tissues as compared with normal ovarian tissues downloaded from GSE47841. Table S6. The downregulated mRNAs in OC tissues in TCGA database compared with normal ovarian tissues in GTEx database. Table S7. The 137 candidate mRNAs expression pattern in OC tissues as compared with normal ovarian tissues. Table S8. The microarray results of the 46 upregulated circRNAs and 595 downregulated circRNAs in OC patients compared with the benign control (fold change > 2 and P-value < 0.05). Table S9. The expression difference of candidate circRNAs between patients with uterine myoma or other benign diseases. Table S10. Comparison of AUC in circ-0003972 + CA125, circ-0007288 + CA125, circCOMBO + CA125. [file 13048_2022_988_MOESM3_ESM.docx]

Table S1 Clinicopathological characteristics of the enrolled patients. (Page 1)

|  |  | N (%) |
| --- | --- | --- |
| Age^A^ | ≤50 | 66 (55) |
|  | >50 | 54 (45) |
| Menopause | Pre-M | 70 (58.3) |
|  | Post-M | 50 (41.7) |
| Sample type |  |  |
| Benign control^B^ | Uterine myoma | 37 (30.8) |
|  | Abnormal uterine echo | 11 (9.2) |
|  | Bartholin cyst | 3 (2.5) |
|  | Pelvic organ prolapse | 5 (4.2) |
|  | Healthy volunteers | 4 (3.3) |
| Tumor | EOC | 48 (40) |
|  | Non-EOC | 12 (10) |
| EOC^C^ |  |  |
| Histological subtype | Serous | 35 (72.9) |
|  | Others | 13 (27.1) |
| FIGO stage | I-II | 15 (31.2) |
|  | III-IV | 33 (68.8) |
| Lymph node metastasis | Yes | 13 (27.1) |
|  | No | 35 (72.9) |
| Distant metastasis | Yes | 3 (6.2) |
|  | No | 45 (93.8) |
| Histological grade | Low | 11 (22.9) |
|  | Median | 5 (10.4) |
|  | High | 1. (66.7) |

A. Average age for 120 age-matched enrolled women; B. Histological subtype of patients with benign diseases (n=60); C. Clinicopathologic characteristics of patients with epithelial ovarian cancer (EOC, n=48)

| Table S2 Primer sequences for real-time PCR or quantitative real-time PCR. (Page 2)   \| Primers for PCR \| Sequence (5’-3’) \| \| --- \| --- \| \| GAPDH-F \| CAATGACCCCTTCATTGACC \| \| GAPDH-R \| TTGATTTTGGAGGGATCTCG \| \| hsa_circ_0053221-F \| TCCCTTGATGCTTTGGTCAGT \| \| hsa_circ_0053221-R \| TTATCTCGTTGACACGGGGC \| \| has_circ_0062215-F \| CAAGAAGGATCATCTCTACAAGG \| \| has_circ_0062215-R \| CCTGGTAATAGCACATCAGC \| \| hsa_circ_0003972-F \| AGGAAATCACATTCTGCCTGA \| \| hsa_circ_0003972-R \| CAACGGCTTTGATCACTACG \| \| hsa_circ_0007288-F \| CAGCCTTCCAAGTTCTGATGT \| \| has_circ_0007288-R \| GGGAAGCCCTGTGACCAAGTGG \|   Table S3. The miRNA target prediction software circInteratome and circbank predicted miRNAs which could bind hsa_circ_0003972. (Page 3) | | |
| --- | --- | --- | --- | --- | --- | --- | --- | --- | --- | --- | --- | --- | --- | --- | --- | --- | --- | --- | --- | --- | --- | --- | --- | --- |
| circInteratome | circBank |  |
| hsa-miR-1257 | hsa-miR-3691-3p |  |
| hsa-miR-1287 | hsa-miR-3691-5p |  |
| hsa-miR-1299 | hsa-miR-4427 |  |
| hsa-miR-151-3p | hsa-miR-1257 |  |
| hsa-miR-1825 | hsa-miR-151a-3p |  |
| hsa-miR-183-5p | hsa-miR-1538 |  |
| hsa-miR-203 | hsa-miR-203a-3p |  |
| hsa-miR-296-5p | hsa-miR-31-5p |  |
| hsa-miR-31 | hsa-miR-3165 |  |
| hsa-miR-409-3p | hsa-miR-3681-5p |  |
| hsa-miR-488 | hsa-miR-4316 |  |
| hsa-miR-516b | hsa-miR-4761-5p |  |
| hsa-miR-545 | hsa-miR-4786-3p |  |
| hsa-miR-570 | hsa-miR-508-5p |  |
| hsa-miR-671-5p | hsa-miR-541-3p |  |
| hsa-miR-889 | hsa-miR-5692b |  |
| hsa-miR-921 | hsa-miR-5692c |  |
|  | hsa-miR-570-3p |  |
|  | hsa-miR-654-5p |  |
|  | hsa-miR-659-5p |  |
|  | hsa-miR-6865-5p |  |
|  | hsa-miR-7161-3p |  |

| Table S4. The miRNA target prediction software circInteratome and circbank predicted miRNAs which could bind hsa_circ_0007288. (Page 4-5) | |
| --- | --- |
| circInteractome | circBank |
| hsa-miR-1179 | hsa-miR-629-3p |
| hsa-miR-1184 | hsa-miR-515-5p |
| hsa-miR-1205 | hsa-miR-143-5p |
| hsa-miR-1208 | hsa-miR-1976 |
| hsa-miR-1228 | hsa-miR-3140-5p |
| hsa-miR-1248 | hsa-miR-3199 |
| hsa-miR-1276 | hsa-miR-365a-5p |
| hsa-miR-136 | hsa-miR-365b-5p |
| hsa-miR-146b-3p | hsa-miR-382-5p |
| hsa-miR-194 | hsa-miR-4723-3p |
| hsa-miR-197 | hsa-miR-499a-3p |
| hsa-miR-382 | hsa-miR-499b-3p |
| hsa-miR-421 | hsa-miR-500a-5p |
| hsa-miR-431 | hsa-miR-630 |
| hsa-miR-486-3p | hsa-miR-653-3p |
| hsa-miR-496 | hsa-miR-664a-3p |
| hsa-miR-515-5p | hsa-miR-6894-5p |
| hsa-miR-515-5p | hsa-miR-766-5p |
| hsa-miR-515-5p | hsa-miR-1273f |
| hsa-miR-526b | hsa-miR-1321 |
| hsa-miR-532-3p | hsa-miR-138-5p |
| hsa-miR-579 | hsa-miR-181b-5p |
| hsa-miR-607 | hsa-miR-181d-5p |
| hsa-miR-614 | hsa-miR-2115-3p |
| hsa-miR-621 | hsa-miR-3150b-3p |
| hsa-miR-628-3p | hsa-miR-3677-5p |
| hsa-miR-629 | hsa-miR-3691-5p |
| hsa-miR-630 | hsa-miR-379-3p |
| hsa-miR-630 | hsa-miR-3928-5p |
| hsa-miR-652 | hsa-miR-411-3p |
| hsa-miR-654-3p | hsa-miR-4314 |
| hsa-miR-663b | hsa-miR-4328 |
| hsa-miR-665 | hsa-miR-4419a |
| hsa-miR-671-5p | hsa-miR-4456 |
| hsa-miR-890 | hsa-miR-4487 |
| hsa-miR-935 | hsa-miR-4496 |
|  | hsa-miR-4510 |
|  | hsa-miR-4652-5p |
|  | hsa-miR-4668-3p |
|  | hsa-miR-4680-3p |
|  | hsa-miR-4709-5p |
|  | hsa-miR-4730 |
|  | hsa-miR-4738-3p |
|  | hsa-miR-4739 |
|  | hsa-miR-4742-3p |
|  | hsa-miR-4784 |
|  | hsa-miR-501-5p |
|  | hsa-miR-5096 |
|  | hsa-miR-548q |

| Table S5. The expression profile of the miRNAs that were up-regulated in ovarian cancer tissues as compared with normal ovarian tissues downloaded from GSE47841. (Page 6-8) | | | | | | | | |
| --- | --- | --- | --- | --- | --- | --- | --- | --- |
|  | miRID_latest | logFC | AveExpr | t | P.Value | adj.P.Val | B |  |
| hsa-miR-200a* | hsa-miR-200a-5p | 5.307523 | 5.5155411 | 14.827241 | 5.52E-15 | 6.10E-12 | 24.162488 |  |
| hsa-miR-200c | hsa-miR-200c-3p | 4.1269534 | 13.13619 | 12.26469 | 6.22E-13 | 3.44E-10 | 19.571175 |  |
| hsa-miR-141 | hsa-miR-141-3p | 5.9532484 | 7.5889769 | 12.003786 | 1.05E-12 | 3.85E-10 | 19.061904 |  |
| hsa-miR-200b* | hsa-miR-200b-5p | 3.821851 | 7.3262342 | 11.728934 | 1.82E-12 | 5.04E-10 | 18.516417 |  |
| hsa-miR-200a | hsa-miR-200a-3p | 5.4461882 | 7.0362693 | 10.326533 | 3.57E-11 | 6.58E-09 | 15.583629 |  |
| hsa-miR-200b | hsa-miR-200b-3p | 5.2471629 | 7.5967283 | 9.7872486 | 1.20E-10 | 1.89E-08 | 14.386693 |  |
| hsa-miR-106a | hsa-miR-106a-5p | 1.7558328 | 11.802567 | 9.1674171 | 5.04E-10 | 6.36E-08 | 12.962086 |  |
| hsa-miR-182 | hsa-miR-182-5p | 5.3812281 | 8.0827426 | 9.152337 | 5.22E-10 | 6.36E-08 | 12.926771 |  |
| hsa-miR-425 | hsa-miR-425-5p | 2.5276073 | 9.8221669 | 9.1109866 | 5.76E-10 | 6.36E-08 | 12.829775 |  |
| hsa-miR-17 | hsa-miR-17-5p | 1.6684902 | 12.145141 | 8.5185494 | 2.39E-09 | 1.66E-07 | 11.414457 |  |
| hsa-miR-93 | hsa-miR-93-5p | 1.9152668 | 11.718836 | 8.0646507 | 7.36E-09 | 4.07E-07 | 10.298079 |  |
| hsa-miR-18a | hsa-miR-18a-5p | 2.8158791 | 8.2544959 | 7.9124293 | 1.08E-08 | 5.18E-07 | 9.9176116 |  |
| hsa-miR-296-3p | hsa-miR-296-3p | 2.8585405 | 4.595762 | 7.8041986 | 1.42E-08 | 6.27E-07 | 9.6452801 |  |
| hsa-miR-93* | hsa-miR-93-3p | 2.2865016 | 7.0639166 | 7.6426109 | 2.14E-08 | 8.76E-07 | 9.2359296 |  |
| hsa-miR-200c* | hsa-miR-200c-5p | 2.4905971 | 2.7452453 | 7.2590659 | 5.76E-08 | 2.19E-06 | 8.2514801 |  |
| hsa-miR-18b | hsa-miR-18b-5p | 2.8298554 | 4.1183714 | 6.8055277 | 1.90E-07 | 5.83E-06 | 7.0656394 |  |
| hsa-miR-20a | hsa-miR-20a-5p | 1.599597 | 11.403497 | 6.7226021 | 2.37E-07 | 6.70E-06 | 6.8464778 |  |
| hsa-miR-378* | hsa-miR-378a-5p | 2.9975473 | 5.3499044 | 6.5978018 | 3.30E-07 | 8.52E-06 | 6.5153886 |  |
| hsa-miR-15b | hsa-miR-15b-5p | 2.2467919 | 8.9325365 | 6.4341366 | 5.12E-07 | 1.26E-05 | 6.079026 |  |
| hsa-miR-106b | hsa-miR-106b-5p | 1.5498901 | 11.154103 | 6.3364754 | 6.66E-07 | 1.53E-05 | 5.8175481 |  |
| hsa-miR-664 | hsa-miR-664a-3p | 1.0935082 | 2.5953516 | 5.9562631 | 1.87E-06 | 4.05E-05 | 4.7928081 |  |
| hsa-miR-4298 | hsa-miR-4298 | 1.6807549 | 8.109364 | 5.943928 | 1.94E-06 | 4.06E-05 | 4.7594061 |  |
| hsa-miR-183 | hsa-miR-183-5p | 3.7634886 | 4.5310235 | 5.9421304 | 1.95E-06 | 4.06E-05 | 4.7545377 |  |
| hsa-miR-24-2* | hsa-miR-24-2-5p | 2.1436607 | 5.0568548 | 5.8461382 | 2.53E-06 | 4.90E-05 | 4.4943113 |  |
| hsa-miR-106b* | hsa-miR-106b-3p | 1.4373352 | 8.2202403 | 5.7770461 | 3.06E-06 | 5.54E-05 | 4.3067247 |  |
| hsa-miR-27a | hsa-miR-27a-3p | 1.3668011 | 10.783737 | 5.706999 | 3.70E-06 | 6.53E-05 | 4.1163304 |  |
| hsa-miR-18a* | hsa-miR-18a-3p | 2.334376 | 3.086342 | 5.5930599 | 5.07E-06 | 8.36E-05 | 3.8062409 |  |
| hsa-miR-203 | hsa-miR-203a-3p | 4.1536336 | 6.5281335 | 5.5251977 | 6.11E-06 | 9.78E-05 | 3.6213622 |  |
| hsa-miR-378c | hsa-miR-378c | 2.2447456 | 8.5561166 | 5.4999428 | 6.55E-06 | 0.0001034 | 3.5525304 |  |
| hsa-miR-1913 | hsa-miR-1913 | 1.2047582 | 2.8775996 | 5.3704672 | 9.36E-06 | 0.0001416 | 3.1994653 |  |
| hsa-miR-103 | hsa-miR-103a-3p | 0.6916593 | 13.382315 | 5.3632262 | 9.55E-06 | 0.0001426 | 3.1797134 |  |
| hsa-miR-15a | hsa-miR-15a-5p | 1.6463918 | 6.9288323 | 5.3496462 | 9.91E-06 | 0.000146 | 3.142669 |  |
| hsa-miR-23a | hsa-miR-23a-3p | 1.0288241 | 12.760799 | 5.2382996 | 1.35E-05 | 0.000196 | 2.8389002 |  |
| hsa-miR-107 | hsa-miR-107 | 0.7899214 | 12.51542 | 5.1610868 | 1.67E-05 | 0.0002305 | 2.6282724 |  |
| hsa-miR-23a* | hsa-miR-23a-5p | 2.2213141 | 5.2014677 | 5.140131 | 1.77E-05 | 0.0002413 | 2.5711177 |  |
| hsa-miR-4317 | hsa-miR-4317 | 1.652669 | 3.3678372 | 5.1345784 | 1.80E-05 | 0.000242 | 2.5559745 |  |
| hsa-miR-205 | hsa-miR-205-5p | 6.8754715 | 6.4046533 | 5.0746885 | 2.12E-05 | 0.0002755 | 2.3926742 |  |
| hsa-miR-625 | hsa-miR-625-5p | 1.6887972 | 6.9505223 | 5.0561569 | 2.23E-05 | 0.0002866 | 2.342159 |  |
| hsa-miR-187 | hsa-miR-187-3p | 3.9156051 | 5.4988891 | 4.988713 | 2.69E-05 | 0.0003338 | 2.1583877 |  |
| hsa-miR-378 | hsa-miR-378a-3p | 1.9615084 | 10.213616 | 4.9631343 | 2.89E-05 | 0.0003543 | 2.0887261 |  |
| hsa-miR-3195 | hsa-miR-3195 | 1.0850722 | 5.8587555 | 4.9507375 | 2.99E-05 | 0.0003622 | 2.0549721 |  |
| hsa-miR-671-3p | hsa-miR-671-3p | 1.3457527 | 2.4545752 | 4.9471627 | 3.02E-05 | 0.0003622 | 2.0452399 |  |
| hsa-miR-130b | hsa-miR-130b-3p | 2.035063 | 8.3676949 | 4.9063758 | 3.38E-05 | 0.0004011 | 1.9342318 |  |
| hsa-miR-2277 | hsa-miR-2277-3p | 1.5928529 | 4.7137663 | 4.8889048 | 3.54E-05 | 0.0004165 | 1.8867021 |  |
| hsa-miR-135b* | hsa-miR-135b-3p | 2.0663005 | 2.4501 | 4.7911276 | 4.64E-05 | 0.00054 | 1.6209636 |  |
| hsa-miR-940 | hsa-miR-940 | 1.0599129 | 4.0981168 | 4.7257082 | 5.56E-05 | 0.0006336 | 1.4434575 |  |
| hsa-miR-1307 | hsa-miR-1307-3p | 1.5436141 | 8.2330202 | 4.7046412 | 5.90E-05 | 0.0006584 | 1.3863521 |  |
| hsa-miR-422a | hsa-miR-422a | 2.629096 | 6.2478095 | 4.7043944 | 5.90E-05 | 0.0006584 | 1.3856833 |  |
| hsa-miR-16 | hsa-miR-16-5p | 1.1346714 | 12.047131 | 4.5845037 | 8.21E-05 | 0.0008896 | 1.0613108 |  |
| hsa-miR-27a* | hsa-miR-27a-5p | 2.3205538 | 4.0167797 | 4.5151141 | 9.94E-05 | 0.0010461 | 0.8741094 |  |
| hsa-miR-421 | hsa-miR-421 | 2.4161048 | 4.8288816 | 4.4592433 | 0.0001159 | 0.0011751 | 0.723707 |  |
| hsa-miR-181d | hsa-miR-181d-5p | 1.3868721 | 6.4371413 | 4.3830202 | 0.0001429 | 0.0013852 | 0.5190398 |  |
| hsa-miR-629* | hsa-miR-629-3p | 0.9103391 | 3.263527 | 4.2536389 | 0.0002037 | 0.0018913 | 0.1731993 |  |
| hsa-miR-1539 | hsa-miR-1539 | 0.6350971 | 2.2327817 | 4.2260482 | 0.0002196 | 0.0020223 | 0.0997302 |  |
| hsa-miR-4321 | hsa-miR-4321 | 0.9250323 | 2.6609393 | 4.207743 | 0.0002309 | 0.0020741 | 0.0510455 |  |
| hsa-miR-1228 | hsa-miR-1228-3p | 1.2987556 | 3.8034872 | 4.1557282 | 0.000266 | 0.0023518 | -0.087028 |  |
| hsa-miR-766 | hsa-miR-766-3p | 0.9621371 | 3.3464071 | 4.130423 | 0.000285 | 0.0024995 | -0.154053 |  |
| hsa-miR-595 | hsa-miR-595 | 1.6040088 | 2.6316717 | 4.1208748 | 0.0002925 | 0.0025451 | -0.179317 |  |
| hsa-miR-429 | hsa-miR-429 | 2.0989936 | 2.749718 | 3.9905102 | 0.0004166 | 0.0034872 | -0.522744 |  |
| hsa-miR-92a-1* | hsa-miR-92a-1-5p | 1.5805543 | 4.7772572 | 3.9802345 | 0.0004283 | 0.0035319 | -0.549687 |  |
| hsa-miR-425* | hsa-miR-425-3p | 1.0305322 | 7.0328973 | 3.9529762 | 0.000461 | 0.0037736 | -0.621065 |  |
| hsa-miR-1910 | hsa-miR-1910-5p | 1.073424 | 6.8468951 | 3.8933896 | 0.0005413 | 0.0043797 | -0.776601 |  |
| hsa-miR-126 | hsa-miR-126-3p | 1.3433732 | 9.5930644 | 3.8922401 | 0.000543 | 0.0043797 | -0.779595 |  |
| hsa-miR-20b | hsa-miR-20b-5p | 1.6975701 | 8.3147501 | 3.8836313 | 0.0005557 | 0.0044498 | -0.802006 |  |
| hsa-miR-301a | hsa-miR-301a-3p | 1.1418506 | 2.2080382 | 3.7830771 | 0.0007277 | 0.0055604 | -1.062632 |  |
| hsa-miR-934 | hsa-miR-934 | 2.5950267 | 3.1189155 | 3.7789564 | 0.0007358 | 0.0055604 | -1.073265 |  |
| hsa-miR-224 | hsa-miR-224-5p | 2.2828263 | 2.9739959 | 3.7769545 | 0.0007397 | 0.0055604 | -1.07843 |  |
| hsa-miR-25* | hsa-miR-25-5p | 1.3346095 | 6.0212469 | 3.7472511 | 0.0008008 | 0.0059787 | -1.154955 |  |
| hsa-miR-885-5p | hsa-miR-885-5p | 1.8303525 | 5.2243674 | 3.6539722 | 0.0010262 | 0.0074602 | -1.393925 |  |
| hsa-miR-23b | hsa-miR-23b-3p | 0.6241511 | 13.128221 | 3.634654 | 0.0010801 | 0.00775 | -1.443151 |  |
| hsa-miR-92b | hsa-miR-92b-3p | 1.2919597 | 7.6998167 | 3.5346177 | 0.0014062 | 0.009897 | -1.696507 |  |
| hsa-miR-138-1* | hsa-miR-138-1-3p | 1.7235925 | 3.195029 | 3.4699298 | 0.0016659 | 0.0116511 | -1.858885 |  |
| hsa-miR-221 | hsa-miR-221-3p | 1.1277289 | 11.438771 | 3.4613079 | 0.0017039 | 0.0118415 | -1.880437 |  |
| hsa-miR-1825 | hsa-miR-1825 | 1.0182735 | 3.5187812 | 3.4459288 | 0.0017737 | 0.0122495 | -1.918826 |  |
| hsa-miR-25 | hsa-miR-25-3p | 1.1567782 | 8.5777444 | 3.4224346 | 0.0018856 | 0.012862 | -1.977337 |  |
| hsa-miR-3187 | hsa-miR-3187-3p | 1.2287575 | 3.8696526 | 3.3903468 | 0.0020497 | 0.0135621 | -2.056981 |  |
| hsa-miR-484 | hsa-miR-484 | 0.9883346 | 3.4265808 | 3.3792183 | 0.0021097 | 0.0137941 | -2.08453 |  |
| hsa-miR-449b* | hsa-miR-449b-3p | 1.7320314 | 3.2643891 | 3.3171773 | 0.0024767 | 0.0158196 | -2.237395 |  |
| hsa-miR-149 | hsa-miR-149-5p | 1.9860392 | 7.1705966 | 3.2944998 | 0.0026257 | 0.0164849 | -2.292961 |  |
| hsa-miR-128 | hsa-miR-128-3p | 1.4375948 | 5.0452912 | 3.283715 | 0.0026995 | 0.0167579 | -2.319328 |  |
| hsa-miR-877 | hsa-miR-877-5p | 1.1971422 | 7.0156533 | 3.24323 | 0.0029948 | 0.0183845 | -2.417956 |  |
| hsa-miR-1909* | hsa-miR-1909-5p | 1.1434065 | 4.3672396 | 3.1892308 | 0.0034372 | 0.0204197 | -2.548631 |  |
| hsa-miR-935 | hsa-miR-935 | 0.8585709 | 2.3629051 | 3.1580466 | 0.0037205 | 0.0218678 | -2.623624 |  |
| hsa-miR-30d | hsa-miR-30d-5p | 0.8999654 | 9.3648049 | 3.1228125 | 0.0040675 | 0.0231176 | -2.707931 |  |
| hsa-miR-183* | hsa-miR-183-3p | 1.330643 | 3.9692371 | 3.1216369 | 0.0040796 | 0.0231176 | -2.710736 |  |
| hsa-miR-30c | hsa-miR-30c-5p | 0.9228921 | 10.036254 | 3.0951541 | 0.0043613 | 0.0243396 | -2.773788 |  |
| hsa-miR-449b | hsa-miR-449b-5p | 2.5629707 | 3.0977474 | 3.0568475 | 0.0048018 | 0.026243 | -2.864518 |  |
| hsa-miR-3178 | hsa-miR-3178 | 1.0481872 | 10.112155 | 3.0553575 | 0.0048198 | 0.026243 | -2.868036 |  |
| hsa-miR-7-1* | hsa-miR-7-1-3p | 0.6330996 | 2.0628118 | 3.0342222 | 0.0050816 | 0.0271741 | -2.91784 |  |
| hsa-miR-449a | hsa-miR-449a | 2.908846 | 3.1877998 | 2.9951795 | 0.0056012 | 0.0294727 | -3.009376 |  |
| hsa-miR-346 | hsa-miR-346 | 1.0378346 | 4.9297004 | 2.9740142 | 0.0059035 | 0.0309165 | -3.058741 |  |
| hsa-miR-27b* | hsa-miR-27b-5p | 1.5171094 | 5.0980933 | 2.9551324 | 0.0061863 | 0.0317945 | -3.102626 |  |
| hsa-miR-30b* | hsa-miR-30b-3p | 1.1119162 | 5.3604777 | 2.9315794 | 0.0065569 | 0.0329338 | -3.15716 |  |
| hsa-miR-1181 | hsa-miR-1181 | 0.6044014 | 2.1760614 | 2.9136632 | 0.0068529 | 0.034081 | -3.198486 |  |
| hsa-miR-3147 | hsa-miR-3147 | 0.7271329 | 2.8500173 | 2.9000373 | 0.0070864 | 0.0349574 | -3.229825 |  |
| hsa-miR-449c | hsa-miR-449c-5p | 3.2228693 | 3.6117533 | 2.8441312 | 0.0081253 | 0.0397278 | -3.357563 |  |
| hsa-miR-210 | hsa-miR-210-3p | 1.4481649 | 9.8699138 | 2.8340239 | 0.0083279 | 0.0405387 | -3.38051 |  |
| hsa-miR-1226 | hsa-miR-1226-3p | 0.7731218 | 2.2372012 | 2.8202885 | 0.0086107 | 0.0415495 | -3.411621 |  |
| hsa-miR-1281 | hsa-miR-1281 | 0.6766484 | 6.7575489 | 2.8098377 | 0.0088319 | 0.0420979 | -3.435235 |  |
| hsa-miR-222 | hsa-miR-222-3p | 0.7508713 | 11.933494 | 2.7373296 | 0.0105204 | 0.0486402 | -3.597694 |  |
| hsa-miR-1973 | hsa-miR-1973 | 1.4362322 | 5.0904304 | 2.7332102 | 0.0106249 | 0.0489186 | -3.60685 |  |

| Table S6. The down regulated mRNAs in ovarian cancer tissues in TCGA database compared with normal ovarian tissues in GTEx database. (Page 9-71) | | | | |
| --- | --- | --- | --- | --- |
| Ensembl_ID | pvalue | mean_x | mean_y | foldchange |
| ENSG00000280038.1 | 8.12E-56 | 4.5556981 | 6.3722091 | -1.816511 |
| ENSG00000112309.10 | 2.79E-29 | 5.156806 | 6.3111443 | -1.154338 |
| ENSG00000226530.1 | 1.13E-17 | 1.426715 | 2.4324489 | -1.005734 |
| ENSG00000167768.4 | 6.95E-39 | 1.0066809 | 5.7452 | -4.738519 |
| ENSG00000280159.1 | 2.29E-39 | 1.4374477 | 3.27465 | -1.837202 |
| ENSG00000259952.1 | 9.40E-08 | 0.52828 | 2.018225 | -1.489945 |
| ENSG00000206260.3 | 1.05E-18 | 0.0836606 | 1.112592 | -1.028931 |
| ENSG00000279345.1 | 3.40E-33 | 1.9395277 | 3.2936898 | -1.354162 |
| ENSG00000036448.9 | 5.00E-28 | 4.3151539 | 5.802717 | -1.487563 |
| ENSG00000113971.18 | 1.26E-86 | 9.3115024 | 11.366289 | -2.054786 |
| ENSG00000279700.1 | 1.12E-65 | 3.588322 | 5.9955966 | -2.407275 |
| ENSG00000169992.9 | 4.05E-43 | 11.200943 | 12.269685 | -1.068742 |
| ENSG00000112511.17 | 4.10E-42 | 11.244692 | 12.346768 | -1.102076 |
| ENSG00000034239.10 | 2.14E-30 | 5.8203723 | 7.9279068 | -2.107535 |
| ENSG00000259847.1 | 5.60E-42 | 1.1535551 | 5.1430307 | -3.989476 |
| ENSG00000260729.1 | 6.47E-81 | 5.8160737 | 8.4106841 | -2.59461 |
| ENSG00000203818.7 | 2.90E-27 | 1.369543 | 3.751758 | -2.382215 |
| ENSG00000259799.1 | 3.94E-46 | 1.8970609 | 3.8108307 | -1.91377 |
| ENSG00000268442.1 | 9.83E-43 | 2.9154002 | 4.8095909 | -1.894191 |
| ENSG00000226235.1 | 1.75E-37 | 4.8907668 | 8.720142 | -3.829375 |
| ENSG00000251615.3 | 3.56E-60 | 7.5452332 | 9.3620114 | -1.816778 |
| ENSG00000278126.1 | 3.89E-53 | 4.7583959 | 6.2947227 | -1.536327 |
| ENSG00000009724.16 | 2.47E-26 | 5.2472544 | 6.6743 | -1.427046 |
| ENSG00000177519.3 | 2.61E-18 | 5.7715141 | 7.8181068 | -2.046593 |
| ENSG00000206047.2 | 1.75E-12 | 0.6757425 | 2.042458 | -1.366715 |
| ENSG00000231252.1 | 9.37E-17 | 0.3385031 | 1.3953898 | -1.056887 |
| ENSG00000272734.1 | 8.76E-40 | 8.5249804 | 10.194883 | -1.669903 |
| ENSG00000152049.6 | 6.26E-19 | 7.0155749 | 8.7216023 | -1.706027 |
| ENSG00000223561.6 | 7.84E-56 | 1.7160547 | 5.7749114 | -4.058857 |
| ENSG00000257151.1 | 4.40E-69 | 6.9256759 | 9.077392 | -2.151716 |
| ENSG00000167332.7 | 1.63E-13 | 2.0009907 | 3.7161886 | -1.715198 |
| ENSG00000255248.6 | 1.43E-43 | 10.19486 | 12.100093 | -1.905233 |
| ENSG00000213777.5 | 8.47E-48 | 1.838326 | 3.9184602 | -2.080134 |
| ENSG00000248309.5 | 2.90E-114 | 2.375021 | 5.9332705 | -3.558249 |
| ENSG00000236998.2 | 9.63E-19 | 0.6016874 | 1.7540727 | -1.152385 |
| ENSG00000237390.1 | 6.31E-16 | 0.1646914 | 1.2147182 | -1.050027 |
| ENSG00000188385.11 | 3.72E-139 | 4.0534053 | 8.0265943 | -3.973189 |
| ENSG00000179111.8 | 1.96E-47 | 1.8181573 | 3.97445 | -2.156293 |
| ENSG00000204314.10 | 1.38E-50 | 8.1413556 | 10.203334 | -2.061978 |
| ENSG00000226232.8 | 2.49E-21 | 6.5134642 | 7.565542 | -1.052078 |
| ENSG00000183570.16 | 8.84E-24 | 5.5325847 | 6.8252739 | -1.292689 |
| ENSG00000126562.16 | 3.48E-28 | 4.8826317 | 7.789658 | -2.907026 |
| ENSG00000203356.2 | 5.68E-20 | 1.2391019 | 2.3485523 | -1.10945 |
| ENSG00000270015.1 | 8.63E-106 | 6.6140938 | 8.3165352 | -1.702441 |
| ENSG00000135905.18 | 4.55E-40 | 8.3050921 | 10.207875 | -1.902783 |
| ENSG00000279576.1 | 6.20E-07 | 0.0930305 | 2.5579034 | -2.464873 |
| ENSG00000251429.1 | 8.70E-46 | 2.7070697 | 5.3746807 | -2.667611 |
| ENSG00000186675.6 | 1.81E-18 | 2.6062079 | 3.8314125 | -1.225205 |
| ENSG00000233996.1 | 1.32E-28 | 0.9569224 | 2.4227659 | -1.465843 |
| ENSG00000092529.22 | 1.39E-100 | 6.6964845 | 9.1060955 | -2.409611 |
| ENSG00000154975.13 | 3.98E-23 | 1.3645012 | 4.6242784 | -3.259777 |
| ENSG00000016602.9 | 1.65E-12 | 0.8130468 | 2.182 | -1.368953 |
| ENSG00000165646.11 | 1.27E-47 | 2.8266224 | 7.4264534 | -4.599831 |
| ENSG00000106809.10 | 2.47E-99 | 7.0466807 | 11.154683 | -4.108002 |
| ENSG00000092054.12 | 2.60E-31 | 1.6280365 | 5.5996852 | -3.971649 |
| ENSG00000151632.16 | 2.45E-30 | 5.8090069 | 7.8190591 | -2.010052 |
| ENSG00000078295.15 | 6.67E-117 | 4.1440795 | 8.2839398 | -4.13986 |
| ENSG00000154678.16 | 4.58E-69 | 4.52088 | 8.0730023 | -3.552122 |
| ENSG00000081853.14 | 7.35E-49 | 6.6430322 | 8.4996455 | -1.856613 |
| ENSG00000104804.7 | 1.89E-53 | 1.7600303 | 3.7120136 | -1.951983 |
| ENSG00000184271.15 | 2.74E-119 | 8.1805119 | 10.371511 | -2.190999 |
| ENSG00000123560.13 | 1.69E-60 | 3.1928091 | 7.2016375 | -4.008828 |
| ENSG00000259660.2 | 1.20E-117 | 1.8980403 | 6.4563352 | -4.558295 |
| ENSG00000175745.11 | 3.62E-69 | 9.3402905 | 11.517631 | -2.17734 |
| ENSG00000225920.2 | 4.64E-73 | 1.4862501 | 3.9988625 | -2.512612 |
| ENSG00000160867.14 | 8.32E-21 | 8.6493076 | 10.112397 | -1.463089 |
| ENSG00000255176.1 | 3.38E-38 | 2.6139809 | 4.6732534 | -2.059273 |
| ENSG00000280269.1 | 1.31E-25 | 1.3325893 | 2.8029045 | -1.470315 |
| ENSG00000101425.12 | 3.70E-59 | 2.5938115 | 5.8902318 | -3.29642 |
| ENSG00000261496.1 | 3.39E-96 | 1.8745568 | 4.7643398 | -2.889783 |
| ENSG00000123572.16 | 1.85E-70 | 6.56668 | 11.352506 | -4.785826 |
| ENSG00000231827.3 | 3.08E-24 | 0.8901611 | 2.2543307 | -1.36417 |
| ENSG00000141639.11 | 5.80E-40 | 4.6044384 | 6.7160159 | -2.111577 |
| ENSG00000274292.1 | 2.14E-44 | 6.0758907 | 7.4551375 | -1.379247 |
| ENSG00000103742.11 | 8.66E-70 | 7.4815742 | 9.995992 | -2.514418 |
| ENSG00000235268.2 | 2.06E-21 | 0.555384 | 1.8796125 | -1.324228 |
| ENSG00000233452.6 | 1.93E-43 | 4.0256661 | 5.3205295 | -1.294863 |
| ENSG00000254595.1 | 5.88E-45 | 3.2620282 | 4.6912352 | -1.429207 |
| ENSG00000087495.16 | 1.81E-61 | 4.6930086 | 7.8230977 | -3.130089 |
| ENSG00000218052.5 | 9.65E-13 | 2.9133831 | 4.0182352 | -1.104852 |
| ENSG00000242349.5 | 1.75E-24 | 4.0804709 | 5.3027477 | -1.222277 |
| ENSG00000070193.4 | 4.20E-128 | 1.8204301 | 7.6380523 | -5.817622 |
| ENSG00000167371.16 | 2.37E-124 | 7.3555582 | 11.951164 | -4.595605 |
| ENSG00000228925.1 | 2.55E-31 | 4.3290344 | 5.4541977 | -1.125163 |
| ENSG00000161912.17 | 1.77E-64 | 5.4159878 | 8.1502682 | -2.73428 |
| ENSG00000137726.15 | 6.22E-76 | 10.718894 | 12.970808 | -2.251914 |
| ENSG00000279104.1 | 6.00E-43 | 0.5759 | 4.190775 | -3.614875 |
| ENSG00000163297.16 | 6.41E-87 | 9.0860134 | 11.648613 | -2.562599 |
| ENSG00000183578.5 | 6.55E-42 | 6.1374885 | 8.5508489 | -2.41336 |
| ENSG00000142233.11 | 6.48E-32 | 5.3117081 | 6.7711341 | -1.459426 |
| ENSG00000196581.10 | 4.72E-19 | 6.6549248 | 8.6363614 | -1.981437 |
| ENSG00000269737.1 | 5.10E-24 | 4.9626184 | 5.9761977 | -1.013579 |
| ENSG00000168243.10 | 1.02E-53 | 6.8407203 | 10.165463 | -3.324742 |
| ENSG00000143382.13 | 8.58E-24 | 9.1045792 | 10.740285 | -1.635706 |
| ENSG00000177133.10 | 4.19E-73 | 2.9659974 | 6.8054216 | -3.839424 |
| ENSG00000112619.7 | 6.78E-54 | 5.1715947 | 6.9888875 | -1.817293 |
| ENSG00000129596.4 | 1.39E-96 | 5.3174086 | 8.4694466 | -3.152038 |
| ENSG00000102290.21 | 4.95E-64 | 2.0135745 | 6.9033909 | -4.889816 |
| ENSG00000270344.2 | 1.29E-29 | 6.5783174 | 7.5858352 | -1.007518 |
| ENSG00000259417.2 | 4.23E-12 | 4.1069348 | 5.1691818 | -1.062247 |
| ENSG00000197815.4 | 5.36E-62 | 4.1580449 | 6.4492648 | -2.29122 |
| ENSG00000185565.11 | 3.26E-23 | 6.6212315 | 8.1533398 | -1.532108 |
| ENSG00000197976.10 | 7.04E-52 | 10.773692 | 11.945414 | -1.171722 |
| ENSG00000279311.1 | 6.22E-33 | 1.8397181 | 3.1528602 | -1.313142 |
| ENSG00000064309.14 | 9.57E-23 | 9.9465053 | 11.090047 | -1.143541 |
| ENSG00000259719.5 | 8.86E-31 | 0.260111 | 1.6461648 | -1.386054 |
| ENSG00000250909.1 | 1.33E-29 | 2.2525377 | 3.4663773 | -1.21384 |
| ENSG00000280029.3 | 3.50E-14 | 0.7206656 | 2.2077455 | -1.48708 |
| ENSG00000274956.2 | 4.48E-18 | 1.5973298 | 3.0879693 | -1.490639 |
| ENSG00000255438.2 | 1.79E-61 | 1.0655642 | 3.5875182 | -2.521954 |
| ENSG00000254835.1 | 3.53E-07 | 1.3933181 | 2.4320216 | -1.038703 |
| ENSG00000140937.13 | 1.77E-30 | 11.015533 | 12.649258 | -1.633725 |
| ENSG00000241684.5 | 1.04E-141 | 4.2669444 | 8.6882273 | -4.421283 |
| ENSG00000237807.3 | 9.34E-31 | 6.2885924 | 7.6796102 | -1.391018 |
| ENSG00000226342.1 | 2.36E-22 | 1.4896425 | 2.7549943 | -1.265352 |
| ENSG00000228643.1 | 4.53E-45 | 0.8001165 | 4.1027682 | -3.302652 |
| ENSG00000128482.15 | 8.95E-36 | 4.1230928 | 6.6031955 | -2.480103 |
| ENSG00000263126.1 | 2.72E-44 | 6.2778728 | 7.6420636 | -1.364191 |
| ENSG00000230630.2 | 5.95E-68 | 7.0294076 | 9.8822875 | -2.85288 |
| ENSG00000151572.16 | 4.70E-36 | 3.6273303 | 7.3483523 | -3.721022 |
| ENSG00000273664.1 | 2.02E-32 | 0.1379067 | 2.9014 | -2.763493 |
| ENSG00000118503.14 | 5.37E-17 | 10.815039 | 11.90174 | -1.086701 |
| ENSG00000165617.14 | 2.73E-55 | 7.6522728 | 9.755975 | -2.103702 |
| ENSG00000256028.2 | 5.42E-41 | 7.0415847 | 8.5613648 | -1.51978 |
| ENSG00000276417.1 | 7.74E-26 | 3.102442 | 4.109408 | -1.006966 |
| ENSG00000230185.4 | 1.56E-14 | 3.9658933 | 5.0381239 | -1.072231 |
| ENSG00000213398.7 | 5.49E-52 | 7.7655174 | 9.1141477 | -1.34863 |
| ENSG00000266872.1 | 9.87E-128 | 2.1158146 | 6.662208 | -4.546393 |
| ENSG00000144649.8 | 2.50E-25 | 7.2094554 | 8.6799386 | -1.470483 |
| ENSG00000187134.12 | 1.29E-48 | 6.3084196 | 8.7169284 | -2.408509 |
| ENSG00000163531.15 | 2.48E-66 | 8.3532325 | 11.092065 | -2.738832 |
| ENSG00000226413.2 | 1.41E-26 | 2.0071334 | 3.5170511 | -1.509918 |
| ENSG00000267653.1 | 3.41E-17 | 0.4446659 | 2.4899125 | -2.045247 |
| ENSG00000225333.6 | 1.48E-26 | 0.0545482 | 1.5115114 | -1.456963 |
| ENSG00000110811.19 | 1.75E-33 | 9.6408344 | 10.796722 | -1.155887 |
| ENSG00000111879.18 | 4.35E-80 | 5.6329456 | 8.0163182 | -2.383373 |
| ENSG00000103449.11 | 1.83E-23 | 5.8276019 | 7.816117 | -1.988515 |
| ENSG00000167434.9 | 1.03E-23 | 3.7802289 | 5.8084795 | -2.028251 |
| ENSG00000150893.10 | 1.07E-13 | 4.9997549 | 6.3798443 | -1.380089 |
| ENSG00000157399.14 | 8.38E-28 | 4.9887496 | 6.5976545 | -1.608905 |
| ENSG00000091592.15 | 5.05E-62 | 9.1229854 | 10.581682 | -1.458696 |
| ENSG00000182983.14 | 6.46E-71 | 8.3249766 | 9.7861614 | -1.461185 |
| ENSG00000280198.1 | 5.66E-34 | 1.8262198 | 3.3853273 | -1.559107 |
| ENSG00000123384.13 | 1.28E-54 | 13.469451 | 14.777623 | -1.308172 |
| ENSG00000162733.16 | 1.58E-82 | 9.943416 | 11.843132 | -1.899716 |
| ENSG00000266743.1 | 7.00E-24 | 0.2605453 | 1.8525102 | -1.591965 |
| ENSG00000205795.4 | 7.75E-20 | 6.9068138 | 8.1658273 | -1.259013 |
| ENSG00000223839.7 | 7.11E-10 | 4.4925852 | 5.6160216 | -1.123436 |
| ENSG00000197261.11 | 2.00E-07 | 3.535026 | 4.554375 | -1.019349 |
| ENSG00000088280.18 | 1.17E-39 | 10.610571 | 11.627165 | -1.016594 |
| ENSG00000143515.16 | 6.05E-55 | 10.232201 | 11.573135 | -1.340934 |
| ENSG00000136826.14 | 8.17E-31 | 9.0847611 | 10.953986 | -1.869225 |
| ENSG00000226380.7 | 5.22E-18 | 5.8818098 | 6.9374284 | -1.055619 |
| ENSG00000130037.4 | 6.73E-17 | 4.1045642 | 5.929475 | -1.824911 |
| ENSG00000126733.20 | 3.85E-09 | 3.0586248 | 4.1454307 | -1.086806 |
| ENSG00000267361.1 | 2.58E-61 | 0.3434107 | 5.88245 | -5.539039 |
| ENSG00000275017.1 | 1.40E-18 | 1.4349692 | 2.5277898 | -1.092821 |
| ENSG00000166250.11 | 1.83E-24 | 8.0708678 | 9.3510364 | -1.280169 |
| ENSG00000135333.13 | 1.41E-13 | 4.9451332 | 6.0360909 | -1.090958 |
| ENSG00000273000.5 | 9.77E-79 | 4.6287005 | 7.1730284 | -2.544328 |
| ENSG00000216809.1 | 4.27E-17 | 1.5928084 | 2.9697614 | -1.376953 |
| ENSG00000120820.12 | 4.71E-40 | 8.4977566 | 9.8297398 | -1.331983 |
| ENSG00000176165.9 | 1.97E-25 | 2.5073069 | 4.8644659 | -2.357159 |
| ENSG00000163395.16 | 6.52E-17 | 3.2267869 | 5.6152318 | -2.388445 |
| ENSG00000178115.11 | 1.18E-73 | 1.9781403 | 5.5183977 | -3.540257 |
| ENSG00000172247.3 | 6.75E-30 | 6.5599193 | 7.9470239 | -1.387105 |
| ENSG00000271930.1 | 6.05E-27 | 2.300622 | 3.9226966 | -1.622075 |
| ENSG00000228252.8 | 2.66E-51 | 4.3720621 | 7.6434 | -3.271338 |
| ENSG00000263647.1 | 5.08E-39 | 4.2518282 | 5.8488455 | -1.597017 |
| ENSG00000257752.1 | 4.62E-34 | 0.9877358 | 2.5654 | -1.577664 |
| ENSG00000257542.4 | 4.48E-22 | 4.5732377 | 6.140742 | -1.567504 |
| ENSG00000240291.1 | 5.51E-59 | 5.0659921 | 6.7139932 | -1.648001 |
| ENSG00000172399.5 | 3.00E-62 | 2.1534418 | 4.5351568 | -2.381715 |
| ENSG00000213121.2 | 2.37E-25 | 0.5741155 | 2.0880648 | -1.513949 |
| ENSG00000126785.12 | 3.10E-45 | 7.6922864 | 8.9316898 | -1.239403 |
| ENSG00000254433.1 | 9.29E-59 | 1.5484549 | 3.7509511 | -2.202496 |
| ENSG00000251667.1 | 3.31E-43 | 2.4130002 | 4.9386318 | -2.525632 |
| ENSG00000273348.1 | 8.88E-61 | 2.6858155 | 5.9052852 | -3.21947 |
| ENSG00000241404.6 | 3.97E-92 | 8.0949141 | 10.564805 | -2.46989 |
| ENSG00000123612.15 | 3.12E-25 | 5.2596115 | 7.5378761 | -2.278265 |
| ENSG00000177294.6 | 1.61E-20 | 3.4202399 | 4.784483 | -1.364243 |
| ENSG00000034971.14 | 8.20E-15 | 2.8872002 | 4.5506727 | -1.663472 |
| ENSG00000140955.10 | 9.42E-20 | 2.6322482 | 3.9853 | -1.353052 |
| ENSG00000130224.14 | 7.37E-57 | 7.1009351 | 8.8025591 | -1.701624 |
| ENSG00000167676.4 | 7.26E-51 | 7.9984831 | 10.810689 | -2.812206 |
| ENSG00000272597.1 | 3.25E-44 | 1.5959874 | 4.3539352 | -2.757948 |
| ENSG00000130222.10 | 5.84E-25 | 8.0866735 | 9.6856784 | -1.599005 |
| ENSG00000225746.8 | 4.79E-74 | 1.6839592 | 5.6725432 | -3.988584 |
| ENSG00000227744.4 | 2.64E-42 | 0.5107697 | 2.7316023 | -2.220833 |
| ENSG00000238133.6 | 1.83E-30 | 1.4172399 | 3.1257239 | -1.708484 |
| ENSG00000158560.14 | 2.35E-49 | 5.9698893 | 7.8115659 | -1.841677 |
| ENSG00000179242.15 | 2.47E-56 | 4.3903339 | 8.8608034 | -4.47047 |
| ENSG00000234338.1 | 4.28E-32 | 3.9471126 | 5.3119841 | -1.364871 |
| ENSG00000185271.6 | 1.23E-60 | 0.8841905 | 3.9384307 | -3.05424 |
| ENSG00000090534.17 | 1.72E-123 | 3.148401 | 6.064342 | -2.915941 |
| ENSG00000126218.11 | 3.47E-93 | 6.0079926 | 9.2812386 | -3.273246 |
| ENSG00000179008.8 | 3.35E-21 | 1.3954878 | 3.5184693 | -2.122981 |
| ENSG00000279357.1 | 1.16E-27 | 1.8818957 | 3.2746534 | -1.392758 |
| ENSG00000225342.2 | 2.82E-56 | 2.9145284 | 5.0517216 | -2.137193 |
| ENSG00000150995.17 | 9.70E-90 | 8.8693017 | 11.63333 | -2.764028 |
| ENSG00000249212.1 | 1.65E-19 | 1.2548248 | 2.6527625 | -1.397938 |
| ENSG00000280322.1 | 6.64E-57 | 0.2513771 | 4.0132148 | -3.761838 |
| ENSG00000279447.1 | 3.55E-16 | 0.8745644 | 2.0614307 | -1.186866 |
| ENSG00000235674.2 | 1.44E-33 | 1.3708406 | 3.1841034 | -1.813263 |
| ENSG00000174403.15 | 3.62E-17 | 1.8488179 | 3.9863443 | -2.137526 |
| ENSG00000143536.7 | 1.29E-21 | 1.7356704 | 4.2934159 | -2.557746 |
| ENSG00000268350.7 | 2.54E-70 | 9.0462339 | 10.515498 | -1.469264 |
| ENSG00000229582.3 | 1.38E-32 | 1.1667229 | 2.6503886 | -1.483666 |
| ENSG00000213519.2 | 1.36E-25 | 0.5074048 | 1.792175 | -1.28477 |
| ENSG00000132622.10 | 3.70E-33 | 7.5449914 | 8.6732295 | -1.128238 |
| ENSG00000226237.1 | 6.83E-24 | 5.3972821 | 6.4971114 | -1.099829 |
| ENSG00000064205.10 | 6.63E-146 | 6.1343594 | 11.598009 | -5.46365 |
| ENSG00000139597.16 | 1.08E-24 | 9.3257236 | 10.383001 | -1.057278 |
| ENSG00000227329.2 | 4.27E-26 | 0.0644604 | 1.243533 | -1.179073 |
| ENSG00000151006.7 | 8.12E-71 | 6.0958582 | 8.1731114 | -2.077253 |
| ENSG00000280136.2 | 1.75E-21 | 2.8714807 | 4.667433 | -1.795952 |
| ENSG00000133083.14 | 6.38E-49 | 7.0286391 | 9.2660341 | -2.237395 |
| ENSG00000144596.11 | 7.34E-59 | 5.4871511 | 9.221133 | -3.733982 |
| ENSG00000270093.1 | 3.20E-87 | 3.4674928 | 7.156017 | -3.688524 |
| ENSG00000198099.8 | 1.06E-09 | 0.9070234 | 1.9688148 | -1.061791 |
| ENSG00000196990.8 | 5.63E-10 | 1.206311 | 2.3109841 | -1.104673 |
| ENSG00000168785.7 | 4.37E-70 | 8.3424926 | 10.827206 | -2.484713 |
| ENSG00000205090.8 | 7.16E-63 | 5.2678477 | 7.0745284 | -1.806681 |
| ENSG00000166743.9 | 6.30E-81 | 3.8320446 | 6.1139523 | -2.281908 |
| ENSG00000099622.13 | 6.83E-76 | 12.14617 | 14.030935 | -1.884766 |
| ENSG00000265142.6 | 1.03E-27 | 1.3915143 | 2.8771477 | -1.485633 |
| ENSG00000241975.1 | 7.07E-42 | 2.375669 | 4.1605864 | -1.784917 |
| ENSG00000169116.11 | 7.27E-29 | 8.3626043 | 10.229394 | -1.86679 |
| ENSG00000261520.5 | 3.49E-26 | 0.672501 | 2.6612955 | -1.988795 |
| ENSG00000229419.1 | 1.20E-47 | 4.5196432 | 5.668875 | -1.149232 |
| ENSG00000116652.6 | 4.20E-74 | 3.352543 | 5.6732682 | -2.320725 |
| ENSG00000146054.17 | 1.99E-20 | 4.9650535 | 6.1712727 | -1.206219 |
| ENSG00000153956.15 | 2.10E-34 | 7.9124575 | 9.1885477 | -1.27609 |
| ENSG00000106278.11 | 6.14E-53 | 2.6646162 | 5.4572102 | -2.792594 |
| ENSG00000143127.12 | 2.84E-34 | 6.0142005 | 8.022017 | -2.007817 |
| ENSG00000205929.9 | 1.38E-65 | 2.8898384 | 7.8242739 | -4.934435 |
| ENSG00000272810.1 | 5.86E-21 | 2.3050959 | 3.7690636 | -1.463968 |
| ENSG00000127324.8 | 5.24E-36 | 6.0184444 | 8.8674636 | -2.849019 |
| ENSG00000265690.7 | 3.94E-54 | 5.5834568 | 7.5785625 | -1.995106 |
| ENSG00000270300.1 | 6.50E-33 | 1.1513277 | 2.6046773 | -1.45335 |
| ENSG00000119326.14 | 3.52E-24 | 10.434733 | 11.565009 | -1.130276 |
| ENSG00000240247.6 | 1.37E-10 | 0.4566239 | 1.748817 | -1.292193 |
| ENSG00000183092.15 | 5.12E-23 | 6.1503339 | 7.9526466 | -1.802313 |
| ENSG00000271417.2 | 3.72E-78 | 0.819374 | 5.6977614 | -4.878387 |
| ENSG00000233231.1 | 1.19E-30 | 2.4731518 | 3.901133 | -1.427981 |
| ENSG00000234648.1 | 1.27E-58 | 0.1804613 | 7.1669216 | -6.98646 |
| ENSG00000278601.1 | 1.09E-39 | 3.2519854 | 4.5479182 | -1.295933 |
| ENSG00000227999.1 | 2.33E-70 | 1.7066413 | 4.4765091 | -2.769868 |
| ENSG00000172476.3 | 8.20E-46 | 5.3489055 | 7.0952341 | -1.746329 |
| ENSG00000267919.1 | 3.32E-38 | 0.6742356 | 3.6556841 | -2.981449 |
| ENSG00000249379.1 | 2.50E-16 | 0.4434177 | 1.597783 | -1.154365 |
| ENSG00000231845.3 | 7.03E-26 | 0.4367523 | 1.903592 | -1.46684 |
| ENSG00000149927.17 | 2.00E-23 | 6.4034322 | 8.4768386 | -2.073406 |
| ENSG00000213073.4 | 1.36E-65 | 5.2798413 | 7.2367261 | -1.956885 |
| ENSG00000152580.8 | 2.49E-60 | 8.341484 | 12.019663 | -3.678178 |
| ENSG00000137766.16 | 2.61E-19 | 1.7390389 | 4.456608 | -2.717569 |
| ENSG00000250240.5 | 7.08E-24 | 1.6974167 | 2.9319943 | -1.234578 |
| ENSG00000112837.16 | 4.66E-20 | 5.6843351 | 7.52055 | -1.836215 |
| ENSG00000170323.8 | 2.56E-15 | 4.1949632 | 6.0715648 | -1.876602 |
| ENSG00000067048.16 | 5.48E-44 | 0.0338558 | 2.2469295 | -2.213074 |
| ENSG00000239839.5 | 8.10E-09 | 0.4398516 | 1.4598773 | -1.020026 |
| ENSG00000143768.11 | 6.15E-58 | 4.8894769 | 9.4978852 | -4.608408 |
| ENSG00000241218.1 | 4.49E-62 | 2.4023769 | 6.1913307 | -3.788954 |
| ENSG00000274678.1 | 1.92E-53 | 1.8930062 | 4.3458773 | -2.452871 |
| ENSG00000180229.12 | 1.08E-31 | 7.4862136 | 9.2654273 | -1.779214 |
| ENSG00000213077.5 | 3.41E-66 | 1.9083668 | 5.3655239 | -3.457157 |
| ENSG00000214578.5 | 3.12E-54 | 1.4138926 | 3.5755375 | -2.161645 |
| ENSG00000077616.10 | 2.34E-87 | 5.7790048 | 8.7845136 | -3.005509 |
| ENSG00000268416.1 | 7.62E-22 | 4.439511 | 7.0545966 | -2.615086 |
| ENSG00000261490.1 | 1.69E-65 | 6.0029683 | 8.1998614 | -2.196893 |
| ENSG00000167780.11 | 9.61E-15 | 1.683457 | 2.920467 | -1.23701 |
| ENSG00000256436.1 | 3.69E-26 | 2.3979742 | 3.6768716 | -1.278897 |
| ENSG00000234911.1 | 1.06E-42 | 2.8291177 | 4.28625 | -1.457132 |
| ENSG00000255974.6 | 1.07E-34 | 2.3507449 | 4.5206705 | -2.169926 |
| ENSG00000204657.3 | 1.87E-28 | 0.999794 | 2.583642 | -1.583848 |
| ENSG00000271778.1 | 3.21E-39 | 2.4229382 | 4.4341227 | -2.011185 |
| ENSG00000266486.1 | 1.66E-08 | 0.5899473 | 1.6342841 | -1.044337 |
| ENSG00000278195.1 | 1.99E-29 | 4.2363654 | 7.1506761 | -2.914311 |
| ENSG00000132702.12 | 1.94E-21 | 2.9858854 | 4.5609761 | -1.575091 |
| ENSG00000203785.8 | 4.52E-30 | 0.5168301 | 3.6791443 | -3.162314 |
| ENSG00000104760.16 | 2.74E-29 | 0.6534115 | 2.65145 | -1.998039 |
| ENSG00000175535.6 | 1.15E-34 | 0.1412162 | 4.8969659 | -4.75575 |
| ENSG00000018625.14 | 8.60E-49 | 5.6692854 | 9.6229375 | -3.953652 |
| ENSG00000153291.15 | 4.92E-101 | 7.8072098 | 10.887105 | -3.079895 |
| ENSG00000241020.1 | 7.77E-59 | 0.8656 | 3.6653148 | -2.799715 |
| ENSG00000241288.7 | 4.06E-28 | 6.2664296 | 7.3672545 | -1.100825 |
| ENSG00000260572.1 | 3.74E-34 | 4.5961697 | 5.9021886 | -1.306019 |
| ENSG00000224999.1 | 4.52E-21 | 0.2223344 | 1.577717 | -1.355383 |
| ENSG00000252464.1 | 7.22E-16 | 0.9663384 | 2.3231818 | -1.356843 |
| ENSG00000276248.1 | 2.11E-40 | 4.9320776 | 6.6218307 | -1.689753 |
| ENSG00000280214.1 | 5.58E-49 | 2.9107723 | 5.188592 | -2.27782 |
| ENSG00000101004.14 | 1.98E-29 | 9.3475554 | 10.608717 | -1.261162 |
| ENSG00000256508.2 | 8.16E-120 | 1.1448585 | 5.4774034 | -4.332545 |
| ENSG00000131459.12 | 1.10E-68 | 8.2953337 | 10.586377 | -2.291044 |
| ENSG00000169291.9 | 1.85E-65 | 6.8778141 | 8.6292864 | -1.751472 |
| ENSG00000249931.4 | 8.19E-94 | 2.1640807 | 4.8844011 | -2.72032 |
| ENSG00000182636.5 | 5.12E-59 | 9.2527484 | 11.008911 | -1.756163 |
| ENSG00000242735.1 | 8.30E-10 | 2.5765804 | 3.7888534 | -1.212273 |
| ENSG00000100968.13 | 1.41E-72 | 10.887262 | 12.976615 | -2.089353 |
| ENSG00000183747.11 | 5.02E-18 | 0.1522993 | 1.2199136 | -1.067614 |
| ENSG00000268278.1 | 1.19E-28 | 0.8907322 | 2.5248875 | -1.634155 |
| ENSG00000279746.1 | 8.20E-31 | 1.2280186 | 3.7172727 | -2.489254 |
| ENSG00000224812.2 | 5.07E-39 | 2.3362611 | 4.190725 | -1.854464 |
| ENSG00000197083.11 | 2.04E-75 | 5.1728148 | 8.8636932 | -3.690878 |
| ENSG00000263096.1 | 1.86E-38 | 0.4043914 | 2.8511 | -2.446709 |
| ENSG00000269910.1 | 9.63E-40 | 3.9810905 | 5.4106591 | -1.429569 |
| ENSG00000281832.1 | 4.80E-45 | 0.4675236 | 4.123033 | -3.655509 |
| ENSG00000250064.1 | 1.81E-15 | 0.1516993 | 1.3154011 | -1.163702 |
| ENSG00000100242.15 | 5.48E-29 | 11.644771 | 12.819588 | -1.174817 |
| ENSG00000167311.13 | 1.02E-25 | 5.2142217 | 6.5414375 | -1.327216 |
| ENSG00000205212.3 | 4.17E-23 | 1.5066745 | 3.5804659 | -2.073791 |
| ENSG00000163596.16 | 1.83E-29 | 8.3326604 | 9.3538648 | -1.021204 |
| ENSG00000223039.1 | 3.18E-14 | 0.4302413 | 1.5894966 | -1.159255 |
| ENSG00000187690.3 | 5.22E-29 | 1.8285458 | 3.7016989 | -1.873153 |
| ENSG00000270105.1 | 5.90E-22 | 0.7200007 | 2.0792955 | -1.359295 |
| ENSG00000095627.9 | 1.30E-17 | 1.8554866 | 3.4530761 | -1.59759 |
| ENSG00000230953.2 | 5.70E-20 | 1.179311 | 2.4036182 | -1.224307 |
| ENSG00000115468.11 | 2.79E-27 | 9.5498971 | 10.775002 | -1.225105 |
| ENSG00000260305.1 | 5.21E-13 | 0.1090313 | 1.1666432 | -1.057612 |
| ENSG00000124006.14 | 3.86E-26 | 12.794247 | 14.115217 | -1.320971 |
| ENSG00000260706.5 | 9.73E-19 | 0.0198236 | 1.4427193 | -1.422896 |
| ENSG00000166960.16 | 4.06E-39 | 4.4805246 | 6.180808 | -1.700283 |
| ENSG00000181800.5 | 1.10E-81 | 1.7685513 | 5.548508 | -3.779957 |
| ENSG00000276772.1 | 1.28E-25 | 1.4256093 | 2.7297545 | -1.304145 |
| ENSG00000180190.11 | 6.64E-35 | 8.5900823 | 9.9346136 | -1.344531 |
| ENSG00000227363.1 | 8.90E-13 | 0.2980057 | 1.4680511 | -1.170045 |
| ENSG00000223806.7 | 1.58E-16 | 0.2137475 | 1.2399727 | -1.026225 |
| ENSG00000189409.12 | 8.50E-16 | 8.886811 | 9.9592523 | -1.072441 |
| ENSG00000231544.3 | 5.67E-35 | 0.5381726 | 2.3972364 | -1.859064 |
| ENSG00000095564.13 | 1.29E-65 | 10.230982 | 11.674525 | -1.443543 |
| ENSG00000128596.16 | 1.66E-38 | 5.4879329 | 6.8391455 | -1.351213 |
| ENSG00000103168.16 | 1.38E-60 | 10.767785 | 12.108241 | -1.340455 |
| ENSG00000236266.1 | 3.26E-11 | 2.4505566 | 3.4858432 | -1.035287 |
| ENSG00000258399.6 | 1.66E-72 | 1.7719143 | 6.0993807 | -4.327466 |
| ENSG00000232149.1 | 2.11E-62 | 2.7795339 | 4.8768852 | -2.097351 |
| ENSG00000134007.3 | 7.44E-34 | 1.7213936 | 2.9491648 | -1.227771 |
| ENSG00000187758.7 | 1.03E-19 | 0.6660931 | 2.2423557 | -1.576263 |
| ENSG00000158258.15 | 9.39E-89 | 8.946395 | 13.135734 | -4.189339 |
| ENSG00000171817.16 | 1.19E-107 | 5.066895 | 8.8493614 | -3.782466 |
| ENSG00000120160.10 | 1.11E-16 | 1.460027 | 2.4990841 | -1.039057 |
| ENSG00000187987.9 | 3.13E-31 | 4.1301432 | 5.5215261 | -1.391383 |
| ENSG00000272234.1 | 3.68E-07 | 1.4495234 | 2.5403273 | -1.090804 |
| ENSG00000176716.4 | 8.00E-30 | 0.623701 | 2.1057386 | -1.482038 |
| ENSG00000259275.2 | 3.52E-134 | 5.0425582 | 8.5097761 | -3.467218 |
| ENSG00000220378.3 | 8.04E-17 | 0.3974981 | 1.5446909 | -1.147193 |
| ENSG00000226516.7 | 5.99E-39 | 0.6262329 | 3.3743739 | -2.748141 |
| ENSG00000143632.14 | 6.48E-25 | 3.0716504 | 5.9938716 | -2.922221 |
| ENSG00000259511.1 | 3.85E-38 | 1.7121215 | 3.6107625 | -1.898641 |
| ENSG00000185303.15 | 4.53E-09 | 1.9762172 | 3.0070932 | -1.030876 |
| ENSG00000100867.14 | 3.97E-18 | 5.6588895 | 8.1192068 | -2.460317 |
| ENSG00000140459.17 | 1.79E-41 | 6.726615 | 9.8124125 | -3.085797 |
| ENSG00000269194.1 | 8.63E-24 | 4.2494451 | 5.406375 | -1.15693 |
| ENSG00000124194.15 | 3.53E-42 | 3.0887368 | 5.3445159 | -2.255779 |
| ENSG00000150625.16 | 1.93E-13 | 5.7384468 | 7.2895614 | -1.551115 |
| ENSG00000214021.15 | 1.72E-86 | 10.225723 | 12.691495 | -2.465772 |
| ENSG00000089472.16 | 2.68E-30 | 8.4406205 | 9.838867 | -1.398247 |
| ENSG00000241489.7 | 4.30E-54 | 7.6006146 | 9.4965716 | -1.895957 |
| ENSG00000273674.4 | 1.36E-50 | 1.1412537 | 3.3948523 | -2.253599 |
| ENSG00000237975.6 | 1.47E-46 | 4.7470146 | 6.8486852 | -2.101671 |
| ENSG00000224888.4 | 1.92E-28 | 3.9167284 | 5.0929239 | -1.176195 |
| ENSG00000244953.1 | 1.73E-47 | 1.9903866 | 6.9632477 | -4.972861 |
| ENSG00000157796.17 | 2.34E-48 | 10.04431 | 11.203913 | -1.159603 |
| ENSG00000142178.7 | 1.21E-11 | 7.6089979 | 9.5135011 | -1.904503 |
| ENSG00000178809.11 | 9.68E-51 | 6.3223759 | 7.9122761 | -1.5899 |
| ENSG00000224986.2 | 1.15E-18 | 1.0868305 | 2.1805045 | -1.093674 |
| ENSG00000158163.14 | 1.74E-69 | 6.7684523 | 8.2930432 | -1.524591 |
| ENSG00000084636.17 | 4.24E-83 | 10.269877 | 13.60442 | -3.334544 |
| ENSG00000138688.15 | 1.36E-79 | 10.529722 | 11.860407 | -1.330685 |
| ENSG00000008277.14 | 2.26E-22 | 7.114263 | 8.2794136 | -1.165151 |
| ENSG00000273287.2 | 8.38E-37 | 1.5043955 | 4.5670511 | -3.062656 |
| ENSG00000233766.7 | 1.09E-22 | 1.0494119 | 2.5185432 | -1.469131 |
| ENSG00000008118.9 | 5.32E-39 | 6.2556499 | 9.0172398 | -2.76159 |
| ENSG00000225828.1 | 2.31E-26 | 7.9733749 | 8.9761432 | -1.002768 |
| ENSG00000163207.6 | 4.27E-08 | 0.8264931 | 1.9625216 | -1.136029 |
| ENSG00000230910.2 | 1.11E-74 | 1.5738864 | 5.7121636 | -4.138277 |
| ENSG00000216921.7 | 2.35E-37 | 0.881227 | 2.9865989 | -2.105372 |
| ENSG00000120049.18 | 2.47E-47 | 6.3389508 | 8.1731909 | -1.83424 |
| ENSG00000279166.1 | 1.74E-57 | 0.9446394 | 3.4241114 | -2.479472 |
| ENSG00000228830.1 | 6.82E-112 | 3.1193745 | 8.6269409 | -5.507566 |
| ENSG00000144227.4 | 2.91E-30 | 1.7297322 | 4.5132989 | -2.783567 |
| ENSG00000205853.10 | 5.14E-23 | 4.0238401 | 5.0380364 | -1.014196 |
| ENSG00000129467.13 | 2.45E-12 | 7.8547838 | 8.8903557 | -1.035572 |
| ENSG00000224713.4 | 8.63E-135 | 4.2006344 | 8.0011693 | -3.800535 |
| ENSG00000172935.8 | 1.09E-29 | 8.3001339 | 10.030519 | -1.730385 |
| ENSG00000241728.5 | 4.66E-30 | 2.2068241 | 4.3570682 | -2.150244 |
| ENSG00000104879.4 | 2.22E-11 | 3.7990933 | 5.4912807 | -1.692187 |
| ENSG00000083067.22 | 1.52E-56 | 3.6206351 | 7.5735909 | -3.952956 |
| ENSG00000124762.13 | 7.94E-28 | 10.965967 | 12.394357 | -1.42839 |
| ENSG00000100285.9 | 1.85E-19 | 7.5630685 | 9.3413125 | -1.778244 |
| ENSG00000272505.1 | 6.77E-44 | 4.1479761 | 5.8916636 | -1.743688 |
| ENSG00000214944.9 | 3.11E-33 | 9.6921802 | 10.778926 | -1.086746 |
| ENSG00000167549.18 | 1.52E-32 | 5.5507444 | 8.1050489 | -2.554304 |
| ENSG00000280157.1 | 6.63E-37 | 4.2290169 | 5.8053875 | -1.576371 |
| ENSG00000221866.9 | 2.54E-09 | 8.1543341 | 9.2246 | -1.070266 |
| ENSG00000179915.20 | 1.31E-26 | 1.7198222 | 4.3468341 | -2.627012 |
| ENSG00000260409.1 | 6.85E-25 | 2.589089 | 4.545675 | -1.956586 |
| ENSG00000204241.7 | 2.24E-39 | 3.2641926 | 5.0435352 | -1.779343 |
| ENSG00000279555.1 | 9.86E-23 | 2.6699095 | 4.5754477 | -1.905538 |
| ENSG00000197380.10 | 4.57E-36 | 7.4602458 | 9.2635295 | -1.803284 |
| ENSG00000102239.4 | 9.16E-28 | 1.2623019 | 4.6171136 | -3.354812 |
| ENSG00000277778.1 | 4.11E-83 | 2.9375255 | 7.1627261 | -4.225201 |
| ENSG00000165072.9 | 1.19E-54 | 7.2391045 | 11.232633 | -3.993528 |
| ENSG00000151892.14 | 6.65E-24 | 6.3958823 | 8.7813761 | -2.385494 |
| ENSG00000271997.1 | 2.49E-74 | 5.319236 | 7.682567 | -2.363331 |
| ENSG00000186462.8 | 1.21E-34 | 5.5061026 | 6.980258 | -1.474155 |
| ENSG00000162383.11 | 1.12E-133 | 3.1189019 | 7.9081125 | -4.789211 |
| ENSG00000280320.1 | 5.07E-08 | 1.8728912 | 2.891625 | -1.018734 |
| ENSG00000267603.1 | 1.66E-19 | 0.1829165 | 1.8436295 | -1.660713 |
| ENSG00000151360.9 | 7.46E-20 | 1.5220776 | 3.2213239 | -1.699246 |
| ENSG00000259803.6 | 8.71E-15 | 4.8836969 | 6.4014523 | -1.517755 |
| ENSG00000174233.11 | 2.73E-51 | 10.424404 | 11.714941 | -1.290537 |
| ENSG00000243480.7 | 1.00E-31 | 1.4037554 | 4.4751682 | -3.071413 |
| ENSG00000277135.1 | 9.05E-108 | 1.5628451 | 6.9448489 | -5.382004 |
| ENSG00000117425.13 | 4.46E-52 | 7.1598064 | 10.418265 | -3.258458 |
| ENSG00000248174.5 | 2.86E-43 | 1.1255456 | 4.524608 | -3.399062 |
| ENSG00000267774.2 | 7.64E-46 | 0.8959496 | 4.9851511 | -4.089201 |
| ENSG00000172915.18 | 2.43E-107 | 6.9981141 | 9.4524614 | -2.454347 |
| ENSG00000267532.3 | 1.15E-65 | 6.2645618 | 8.3517466 | -2.087185 |
| ENSG00000271880.1 | 3.39E-89 | 4.8158289 | 8.5916193 | -3.77579 |
| ENSG00000149633.11 | 5.39E-94 | 5.4842332 | 9.1031386 | -3.618905 |
| ENSG00000126266.3 | 4.15E-52 | 2.5321683 | 6.0629443 | -3.530776 |
| ENSG00000280077.1 | 1.83E-54 | 7.3996897 | 9.1257159 | -1.726026 |
| ENSG00000116285.12 | 4.64E-43 | 10.915981 | 12.571834 | -1.655853 |
| ENSG00000047648.21 | 4.30E-115 | 6.8484155 | 10.02247 | -3.174055 |
| ENSG00000272256.1 | 1.02E-20 | 2.1194348 | 3.4913455 | -1.371911 |
| ENSG00000197256.10 | 2.56E-60 | 11.717488 | 13.202305 | -1.484817 |
| ENSG00000259030.6 | 6.76E-48 | 4.7704699 | 6.5707011 | -1.800231 |
| ENSG00000124406.16 | 1.63E-55 | 8.4457444 | 10.759464 | -2.313719 |
| ENSG00000261554.1 | 5.80E-22 | 0.4071986 | 2.398075 | -1.990876 |
| ENSG00000150672.16 | 9.55E-86 | 5.3628024 | 8.2455545 | -2.882752 |
| ENSG00000212643.2 | 2.41E-15 | 2.9122179 | 4.5836068 | -1.671389 |
| ENSG00000132386.10 | 6.70E-44 | 11.390295 | 13.310695 | -1.9204 |
| ENSG00000150907.6 | 1.50E-49 | 10.806375 | 12.211823 | -1.405448 |
| ENSG00000260060.1 | 4.97E-19 | 2.0372914 | 3.193217 | -1.155926 |
| ENSG00000165092.12 | 1.59E-96 | 9.576457 | 12.1346 | -2.558143 |
| ENSG00000259322.1 | 1.61E-27 | 0.9021776 | 2.1602568 | -1.258079 |
| ENSG00000119698.11 | 4.56E-77 | 4.550111 | 8.0769011 | -3.52679 |
| ENSG00000176438.12 | 3.79E-54 | 7.5431649 | 9.428033 | -1.884868 |
| ENSG00000232133.1 | 2.94E-14 | 2.4279119 | 4.0154318 | -1.58752 |
| ENSG00000187800.13 | 4.32E-117 | 7.1862353 | 10.300782 | -3.114546 |
| ENSG00000115295.19 | 2.73E-48 | 9.6360325 | 10.681776 | -1.045744 |
| ENSG00000130528.11 | 3.25E-41 | 4.0600406 | 6.8813034 | -2.821263 |
| ENSG00000112599.8 | 3.91E-73 | 3.2792792 | 5.2921898 | -2.012911 |
| ENSG00000275995.1 | 8.12E-33 | 0.8218358 | 2.2072909 | -1.385455 |
| ENSG00000248587.6 | 2.76E-74 | 3.3554625 | 7.1684568 | -3.812994 |
| ENSG00000161281.10 | 5.94E-58 | 6.4944172 | 8.4074648 | -1.913048 |
| ENSG00000249633.1 | 1.09E-18 | 0.6952155 | 1.7957727 | -1.100557 |
| ENSG00000280344.1 | 2.76E-45 | 0.8621642 | 3.7391068 | -2.876943 |
| ENSG00000161640.15 | 1.34E-67 | 5.6556317 | 11.01944 | -5.363808 |
| ENSG00000184304.14 | 1.92E-33 | 8.1899943 | 9.3338114 | -1.143817 |
| ENSG00000214688.4 | 1.18E-50 | 3.5322678 | 5.5072784 | -1.975011 |
| ENSG00000168970.20 | 4.93E-95 | 8.8342876 | 11.093993 | -2.259706 |
| ENSG00000113494.16 | 3.42E-39 | 6.5422697 | 8.5051375 | -1.962868 |
| ENSG00000134201.10 | 6.13E-137 | 5.6843933 | 11.893316 | -6.208923 |
| ENSG00000170820.11 | 2.90E-29 | 1.1514597 | 3.9730875 | -2.821628 |
| ENSG00000279118.1 | 1.21E-38 | 6.2145322 | 7.5928284 | -1.378296 |
| ENSG00000267896.1 | 8.11E-70 | 5.1329465 | 6.6009534 | -1.468007 |
| ENSG00000270127.2 | 4.20E-11 | 5.4542119 | 6.5027477 | -1.048536 |
| ENSG00000000971.15 | 1.44E-47 | 10.711705 | 13.079917 | -2.368212 |
| ENSG00000144230.16 | 1.86E-08 | 4.3013048 | 5.4204693 | -1.119165 |
| ENSG00000280323.1 | 3.23E-13 | 1.009163 | 2.8233489 | -1.814186 |
| ENSG00000170935.6 | 1.23E-19 | 0.3163165 | 1.3319659 | -1.015649 |
| ENSG00000277701.4 | 9.42E-24 | 7.2468322 | 9.2578261 | -2.010994 |
| ENSG00000204403.9 | 3.80E-76 | 1.9877993 | 6.2484057 | -4.260606 |
| ENSG00000171016.11 | 9.66E-73 | 7.1324888 | 9.0788989 | -1.94641 |
| ENSG00000173714.7 | 1.13E-82 | 3.6752862 | 12.518276 | -8.84299 |
| ENSG00000154721.14 | 3.57E-81 | 8.0878659 | 9.8727602 | -1.784894 |
| ENSG00000277829.1 | 3.44E-47 | 1.064873 | 3.2137307 | -2.148858 |
| ENSG00000135346.8 | 3.07E-10 | 0.5954215 | 1.8347545 | -1.239333 |
| ENSG00000118271.9 | 1.09E-12 | 0.3725587 | 1.387942 | -1.015383 |
| ENSG00000267838.2 | 6.93E-24 | 4.5702757 | 5.8519273 | -1.281652 |
| ENSG00000254551.1 | 4.10E-26 | 0.8549695 | 2.374233 | -1.519264 |
| ENSG00000139636.15 | 3.16E-48 | 9.8293477 | 10.83272 | -1.003373 |
| ENSG00000173641.17 | 3.42E-43 | 6.8576136 | 9.5878466 | -2.730233 |
| ENSG00000213338.3 | 1.56E-25 | 1.0509439 | 2.156075 | -1.105131 |
| ENSG00000147119.3 | 6.72E-58 | 6.8340549 | 10.045606 | -3.211551 |
| ENSG00000171401.14 | 1.14E-24 | 4.3736749 | 7.2630977 | -2.889423 |
| ENSG00000259236.1 | 3.85E-22 | 0.2777606 | 1.4911261 | -1.213366 |
| ENSG00000089692.8 | 1.60E-25 | 8.0194243 | 9.2930523 | -1.273628 |
| ENSG00000249258.2 | 1.94E-35 | 0.748205 | 2.3676227 | -1.619418 |
| ENSG00000279690.1 | 5.77E-47 | 0.9755783 | 5.1447386 | -4.16916 |
| ENSG00000258893.1 | 2.64E-15 | 0.1108408 | 1.1464023 | -1.035561 |
| ENSG00000073067.13 | 9.50E-09 | 4.2481752 | 5.4010523 | -1.152877 |
| ENSG00000281732.1 | 1.29E-18 | 0.4428308 | 1.811067 | -1.368236 |
| ENSG00000248668.2 | 4.30E-33 | 3.7543136 | 5.1501239 | -1.39581 |
| ENSG00000270510.1 | 5.09E-20 | 1.7984243 | 2.8525818 | -1.054157 |
| ENSG00000188176.11 | 1.12E-53 | 4.6890038 | 7.4106091 | -2.721605 |
| ENSG00000255394.4 | 9.94E-91 | 1.7906036 | 6.9533511 | -5.162748 |
| ENSG00000266708.1 | 3.12E-54 | 1.7453554 | 3.8019455 | -2.05659 |
| ENSG00000132639.12 | 6.94E-18 | 7.2057539 | 8.7529534 | -1.547199 |
| ENSG00000243243.5 | 2.06E-24 | 1.1952169 | 2.4846091 | -1.289392 |
| ENSG00000196208.13 | 9.07E-57 | 10.396032 | 13.950215 | -3.554183 |
| ENSG00000165995.18 | 2.64E-128 | 5.4391298 | 9.3914693 | -3.952339 |
| ENSG00000157502.12 | 1.32E-99 | 6.4570695 | 12.551097 | -6.094027 |
| ENSG00000147465.11 | 4.82E-129 | 5.4819141 | 14.341842 | -8.859928 |
| ENSG00000266050.2 | 4.96E-30 | 0.246915 | 2.718142 | -2.471227 |
| ENSG00000107736.19 | 1.03E-59 | 9.0161893 | 11.946997 | -2.930807 |
| ENSG00000167992.12 | 2.99E-15 | 8.5367129 | 9.589242 | -1.052529 |
| ENSG00000197816.13 | 1.17E-47 | 7.2627115 | 8.7096375 | -1.446926 |
| ENSG00000261616.1 | 2.22E-48 | 3.7456129 | 5.3778716 | -1.632259 |
| ENSG00000259910.1 | 1.02E-24 | 0.4103623 | 1.6517795 | -1.241417 |
| ENSG00000124749.16 | 1.41E-47 | 4.5221124 | 7.2998398 | -2.777727 |
| ENSG00000243368.2 | 2.57E-33 | 3.9212967 | 5.4700489 | -1.548752 |
| ENSG00000154263.17 | 5.44E-155 | 4.3967341 | 11.061911 | -6.665177 |
| ENSG00000240979.1 | 5.44E-86 | 2.0124107 | 4.7846341 | -2.772223 |
| ENSG00000261739.2 | 3.82E-108 | 1.9308795 | 5.7701398 | -3.83926 |
| ENSG00000066405.12 | 1.68E-68 | 1.7807542 | 4.5573295 | -2.776575 |
| ENSG00000154265.15 | 4.41E-74 | 8.2034974 | 10.049939 | -1.846441 |
| ENSG00000198547.8 | 1.04E-32 | 3.9852274 | 5.8889057 | -1.903678 |
| ENSG00000248564.1 | 8.63E-12 | 0.9329239 | 2.0760864 | -1.143162 |
| ENSG00000228069.1 | 2.75E-26 | 0.6214449 | 2.2056307 | -1.584186 |
| ENSG00000172824.14 | 1.17E-90 | 6.5260289 | 9.2408591 | -2.71483 |
| ENSG00000005249.12 | 1.86E-20 | 9.4908208 | 10.568007 | -1.077186 |
| ENSG00000169184.5 | 7.54E-29 | 8.4189623 | 10.043199 | -1.624237 |
| ENSG00000271009.2 | 2.35E-19 | 4.0870303 | 5.2052159 | -1.118186 |
| ENSG00000131584.18 | 7.89E-45 | 10.385686 | 11.503016 | -1.11733 |
| ENSG00000014914.19 | 4.56E-57 | 9.0097255 | 10.815647 | -1.805921 |
| ENSG00000196507.10 | 4.95E-31 | 10.068694 | 11.260497 | -1.191803 |
| ENSG00000265188.1 | 2.73E-60 | 1.1005859 | 4.9198273 | -3.819241 |
| ENSG00000205746.9 | 4.37E-40 | 9.7236621 | 11.104345 | -1.380683 |
| ENSG00000105609.16 | 1.66E-09 | 6.1284136 | 7.1328148 | -1.004401 |
| ENSG00000255104.7 | 7.68E-42 | 2.5457014 | 4.7890636 | -2.243362 |
| ENSG00000183054.11 | 1.06E-63 | 9.275459 | 11.028856 | -1.753397 |
| ENSG00000166147.13 | 6.41E-51 | 10.582037 | 12.315285 | -1.733248 |
| ENSG00000101883.4 | 1.51E-16 | 2.9342236 | 4.5255943 | -1.591371 |
| ENSG00000273129.1 | 3.80E-14 | 1.1802057 | 2.4108557 | -1.23065 |
| ENSG00000164066.12 | 7.99E-40 | 9.1508193 | 10.380913 | -1.230093 |
| ENSG00000189423.11 | 3.53E-44 | 4.1800826 | 6.177158 | -1.997075 |
| ENSG00000266527.1 | 6.42E-31 | 0.605158 | 2.3695932 | -1.764435 |
| ENSG00000188730.4 | 2.69E-59 | 4.109957 | 8.9621318 | -4.852175 |
| ENSG00000144406.18 | 1.60E-20 | 3.6158764 | 5.6343295 | -2.018453 |
| ENSG00000250451.5 | 9.75E-16 | 2.0101041 | 3.9262341 | -1.91613 |
| ENSG00000171766.15 | 5.31E-37 | 9.1439103 | 11.390881 | -2.24697 |
| ENSG00000006071.11 | 3.19E-35 | 1.6185771 | 4.6151068 | -2.99653 |
| ENSG00000105419.17 | 2.35E-30 | 7.5820458 | 9.042517 | -1.460471 |
| ENSG00000280429.1 | 4.59E-23 | 0.7372947 | 2.7737955 | -2.036501 |
| ENSG00000012817.15 | 9.68E-44 | 0.0276508 | 2.2249602 | -2.197309 |
| ENSG00000113319.11 | 2.04E-48 | 7.7282277 | 10.244555 | -2.516327 |
| ENSG00000177679.15 | 1.56E-29 | 6.4994272 | 8.1477364 | -1.648309 |
| ENSG00000272491.1 | 1.84E-104 | 2.317853 | 5.7283398 | -3.410487 |
| ENSG00000253369.1 | 2.69E-52 | 1.4700566 | 4.8919545 | -3.421898 |
| ENSG00000271075.1 | 1.54E-47 | 1.3730518 | 3.6130625 | -2.240011 |
| ENSG00000241186.7 | 1.04E-31 | 4.4224143 | 7.3224727 | -2.900058 |
| ENSG00000144366.15 | 2.95E-50 | 8.2597928 | 9.6985977 | -1.438805 |
| ENSG00000107611.14 | 2.61E-82 | 6.1817993 | 8.806617 | -2.624818 |
| ENSG00000171502.14 | 5.52E-12 | 5.6175702 | 7.2281375 | -1.610567 |
| ENSG00000240771.6 | 3.05E-52 | 9.6999745 | 11.143995 | -1.444021 |
| ENSG00000266993.3 | 1.26E-46 | 4.3220489 | 5.5014568 | -1.179408 |
| ENSG00000163687.13 | 7.43E-12 | 2.7489551 | 3.938033 | -1.189078 |
| ENSG00000249363.1 | 7.03E-28 | 0.628842 | 1.8523 | -1.223458 |
| ENSG00000243697.1 | 3.77E-25 | 0.9810733 | 2.2755216 | -1.294448 |
| ENSG00000242337.5 | 3.41E-24 | 1.7955012 | 3.0565455 | -1.261044 |
| ENSG00000091428.17 | 9.81E-42 | 7.0751835 | 8.4697966 | -1.394613 |
| ENSG00000174306.21 | 7.31E-35 | 9.7711251 | 10.944565 | -1.17344 |
| ENSG00000118245.2 | 3.32E-15 | 0.3036062 | 1.4246091 | -1.121003 |
| ENSG00000160539.4 | 2.08E-119 | 5.5044823 | 8.1175727 | -2.61309 |
| ENSG00000183889.12 | 7.79E-47 | 11.276109 | 12.398876 | -1.122767 |
| ENSG00000262209.2 | 1.75E-18 | 5.2469308 | 6.3214477 | -1.074517 |
| ENSG00000267206.5 | 4.02E-65 | 1.7579379 | 6.4705091 | -4.712571 |
| ENSG00000237624.1 | 2.79E-43 | 3.5232995 | 6.7074557 | -3.184156 |
| ENSG00000229807.9 | 5.51E-124 | 12.036358 | 15.515699 | -3.47934 |
| ENSG00000254266.5 | 1.28E-08 | 1.0548888 | 2.1095205 | -1.054632 |
| ENSG00000142173.14 | 1.61E-33 | 14.02551 | 15.270376 | -1.244866 |
| ENSG00000116883.8 | 2.65E-42 | 7.2176475 | 8.9401761 | -1.722529 |
| ENSG00000100505.13 | 4.09E-13 | 5.2736845 | 6.4175 | -1.143816 |
| ENSG00000231625.4 | 6.29E-66 | 0.7435377 | 4.4925886 | -3.749051 |
| ENSG00000246375.2 | 5.69E-14 | 1.7273539 | 3.2663057 | -1.538952 |
| ENSG00000129194.7 | 3.20E-27 | 6.501921 | 7.8196477 | -1.317727 |
| ENSG00000254656.1 | 1.42E-51 | 1.3776912 | 5.2579943 | -3.880303 |
| ENSG00000141086.17 | 3.16E-35 | 4.1704971 | 5.789958 | -1.619461 |
| ENSG00000171885.13 | 9.98E-10 | 3.5765189 | 4.9630295 | -1.386511 |
| ENSG00000236627.1 | 1.31E-14 | 0.1797632 | 1.2474284 | -1.067665 |
| ENSG00000166405.14 | 1.23E-64 | 7.5897069 | 10.061828 | -2.472121 |
| ENSG00000260413.1 | 1.88E-56 | 0.9618093 | 4.3068716 | -3.345062 |
| ENSG00000035862.12 | 2.30E-50 | 13.690325 | 15.183939 | -1.493613 |
| ENSG00000224660.1 | 3.47E-70 | 8.81758 | 11.112917 | -2.295337 |
| ENSG00000163145.12 | 3.76E-52 | 6.5634811 | 8.4734534 | -1.909972 |
| ENSG00000125414.18 | 3.41E-20 | 0.6931797 | 3.4123534 | -2.719174 |
| ENSG00000185168.5 | 2.04E-23 | 0.8508045 | 2.2916625 | -1.440858 |
| ENSG00000274444.1 | 1.34E-40 | 1.6153969 | 3.34305 | -1.727653 |
| ENSG00000255642.1 | 5.41E-32 | 5.1843547 | 6.2876591 | -1.103304 |
| ENSG00000165323.15 | 1.68E-10 | 4.6454021 | 5.8292386 | -1.183836 |
| ENSG00000223542.1 | 1.58E-25 | 1.3674107 | 3.4252114 | -2.057801 |
| ENSG00000270189.1 | 6.48E-53 | 7.1257138 | 8.7164636 | -1.59075 |
| ENSG00000080007.7 | 3.50E-28 | 3.1106685 | 5.2171557 | -2.106487 |
| ENSG00000246090.6 | 4.33E-33 | 5.843399 | 7.1021807 | -1.258782 |
| ENSG00000279845.1 | 1.93E-26 | 1.3518387 | 3.1390932 | -1.787255 |
| ENSG00000232687.1 | 1.79E-17 | 0.364384 | 1.4381864 | -1.073802 |
| ENSG00000118526.6 | 1.11E-112 | 7.0449952 | 11.186055 | -4.141059 |
| ENSG00000188906.13 | 3.32E-102 | 6.8257816 | 9.8399773 | -3.014196 |
| ENSG00000073146.15 | 3.43E-41 | 4.1314041 | 6.5299011 | -2.398497 |
| ENSG00000278134.1 | 3.66E-33 | 2.8707685 | 4.6209136 | -1.750145 |
| ENSG00000274892.1 | 8.40E-29 | 0.7525821 | 2.6390977 | -1.886516 |
| ENSG00000166510.13 | 1.30E-51 | 6.1098556 | 9.1937341 | -3.083878 |
| ENSG00000228798.1 | 5.66E-85 | 3.1775126 | 6.0859295 | -2.908417 |
| ENSG00000232043.1 | 4.47E-24 | 3.9447308 | 5.6117239 | -1.666993 |
| ENSG00000134245.17 | 7.79E-78 | 7.9974995 | 10.369636 | -2.372137 |
| ENSG00000220785.7 | 3.20E-109 | 7.0123718 | 10.640114 | -3.627742 |
| ENSG00000277954.1 | 5.01E-86 | 3.3433489 | 6.4290386 | -3.08569 |
| ENSG00000181227.3 | 6.12E-25 | 3.7867967 | 4.8314648 | -1.044668 |
| ENSG00000181195.10 | 1.69E-44 | 2.8804874 | 7.904492 | -5.024005 |
| ENSG00000261671.1 | 2.66E-20 | 2.1743518 | 3.313533 | -1.139181 |
| ENSG00000099954.18 | 1.22E-46 | 5.606305 | 8.3310727 | -2.724768 |
| ENSG00000186732.13 | 3.03E-76 | 1.3004962 | 6.1680761 | -4.86758 |
| ENSG00000108176.14 | 1.79E-31 | 6.215311 | 7.5090705 | -1.293759 |
| ENSG00000168679.17 | 4.22E-33 | 7.650099 | 8.9593284 | -1.309229 |
| ENSG00000261744.1 | 1.14E-60 | 1.2755647 | 4.1128875 | -2.837323 |
| ENSG00000261556.8 | 3.93E-55 | 5.8717621 | 7.8056216 | -1.93386 |
| ENSG00000230149.2 | 2.16E-50 | 2.5972473 | 4.1024227 | -1.505175 |
| ENSG00000280407.2 | 2.53E-49 | 5.4375119 | 6.7351284 | -1.297616 |
| ENSG00000275228.1 | 2.79E-18 | 0.32919 | 1.4548977 | -1.125708 |
| ENSG00000243686.2 | 2.29E-14 | 1.5160632 | 2.6568295 | -1.140766 |
| ENSG00000066813.14 | 1.90E-18 | 0.1957542 | 1.3551148 | -1.159361 |
| ENSG00000152784.15 | 2.53E-84 | 5.1207883 | 8.0500386 | -2.92925 |
| ENSG00000170629.14 | 2.32E-24 | 6.4928267 | 7.7482443 | -1.255418 |
| ENSG00000260876.5 | 6.69E-67 | 1.0746193 | 4.4168091 | -3.34219 |
| ENSG00000007171.16 | 1.28E-12 | 3.8780174 | 4.8996148 | -1.021597 |
| ENSG00000279624.3 | 1.32E-32 | 2.1846332 | 3.8942625 | -1.709629 |
| ENSG00000280604.1 | 1.09E-27 | 1.6954189 | 3.1348864 | -1.439468 |
| ENSG00000261625.1 | 9.23E-15 | 3.3892267 | 4.6722682 | -1.283041 |
| ENSG00000100307.12 | 1.45E-105 | 9.7310773 | 11.912669 | -2.181592 |
| ENSG00000267776.1 | 1.31E-19 | 2.705757 | 3.802883 | -1.097126 |
| ENSG00000269068.1 | 8.15E-58 | 3.7552508 | 6.8245534 | -3.069303 |
| ENSG00000182109.7 | 9.78E-52 | 6.7412193 | 8.5299341 | -1.788715 |
| ENSG00000116981.3 | 1.72E-13 | 0.4622461 | 1.5069455 | -1.044699 |
| ENSG00000121297.6 | 3.26E-109 | 8.3982835 | 11.347734 | -2.949451 |
| ENSG00000270231.3 | 9.26E-76 | 9.2466554 | 10.759952 | -1.513297 |
| ENSG00000235531.9 | 8.44E-30 | 6.4424131 | 7.8145761 | -1.372163 |
| ENSG00000269720.1 | 8.24E-20 | 4.0644594 | 5.6775318 | -1.613072 |
| ENSG00000096060.14 | 8.38E-23 | 10.828891 | 12.508066 | -1.679175 |
| ENSG00000206262.8 | 2.40E-106 | 4.2706148 | 11.426847 | -7.156232 |
| ENSG00000146469.12 | 1.02E-44 | 1.4411258 | 3.8714386 | -2.430313 |
| ENSG00000282339.1 | 2.06E-30 | 6.0175072 | 7.3652773 | -1.34777 |
| ENSG00000265511.1 | 9.31E-77 | 3.4999107 | 5.4810773 | -1.981167 |
| ENSG00000279246.1 | 1.43E-42 | 4.2577578 | 5.9372068 | -1.679449 |
| ENSG00000187479.5 | 7.86E-63 | 9.0828253 | 13.360445 | -4.27762 |
| ENSG00000248334.6 | 1.15E-123 | 5.1282988 | 8.2345023 | -3.106203 |
| ENSG00000164292.12 | 4.00E-96 | 9.550457 | 12.208751 | -2.658294 |
| ENSG00000254461.1 | 7.23E-57 | 2.9792348 | 5.6288705 | -2.649636 |
| ENSG00000205683.11 | 1.17E-37 | 6.6674234 | 9.0897068 | -2.422283 |
| ENSG00000196263.7 | 7.98E-81 | 8.0918523 | 10.199994 | -2.108142 |
| ENSG00000005981.12 | 1.72E-21 | 1.9655897 | 3.411808 | -1.446218 |
| ENSG00000279765.3 | 2.11E-13 | 4.9186325 | 6.0064977 | -1.087865 |
| ENSG00000143028.8 | 1.13E-23 | 4.5213566 | 6.8857114 | -2.364355 |
| ENSG00000231104.8 | 1.02E-15 | 1.5417427 | 2.6147602 | -1.073018 |
| ENSG00000215252.11 | 4.21E-102 | 10.262857 | 13.235451 | -2.972594 |
| ENSG00000197376.2 | 5.87E-33 | 0.7264554 | 3.581033 | -2.854578 |
| ENSG00000159251.6 | 1.87E-15 | 3.5814265 | 5.5838989 | -2.002472 |
| ENSG00000264290.1 | 1.06E-34 | 1.7179339 | 3.1552625 | -1.437329 |
| ENSG00000233110.1 | 3.99E-31 | 2.0962702 | 3.5199443 | -1.423674 |
| ENSG00000230224.1 | 1.22E-38 | 3.9378258 | 5.1202659 | -1.18244 |
| ENSG00000250906.1 | 9.13E-42 | 0.7288728 | 3.003725 | -2.274852 |
| ENSG00000254995.4 | 6.03E-25 | 5.195632 | 6.6222466 | -1.426615 |
| ENSG00000211683.3 | 1.43E-41 | 1.3037289 | 3.9556739 | -2.651945 |
| ENSG00000267127.6 | 9.59E-18 | 5.4676382 | 6.5452841 | -1.077646 |
| ENSG00000256973.1 | 2.89E-71 | 1.211527 | 4.118033 | -2.906506 |
| ENSG00000277831.1 | 4.36E-37 | 2.1271277 | 3.7469739 | -1.619846 |
| ENSG00000224975.1 | 1.23E-25 | 5.4867597 | 6.5021727 | -1.015413 |
| ENSG00000251323.2 | 4.34E-16 | 2.8907733 | 4.2336045 | -1.342831 |
| ENSG00000243107.1 | 3.27E-45 | 3.34081 | 4.9789205 | -1.63811 |
| ENSG00000107147.11 | 4.49E-11 | 7.7235807 | 8.9118205 | -1.18824 |
| ENSG00000172179.11 | 2.94E-14 | 1.2727735 | 3.8724591 | -2.599686 |
| ENSG00000132561.13 | 2.45E-41 | 11.191731 | 12.537392 | -1.345661 |
| ENSG00000141540.10 | 8.30E-55 | 8.2739749 | 9.9729966 | -1.699022 |
| ENSG00000279858.1 | 1.24E-21 | 0.3586814 | 1.5445239 | -1.185842 |
| ENSG00000213443.2 | 4.94E-34 | 5.2814969 | 6.7950716 | -1.513575 |
| ENSG00000189182.9 | 2.36E-14 | 0.3738652 | 1.3771784 | -1.003313 |
| ENSG00000090975.12 | 5.96E-81 | 9.1078117 | 11.248297 | -2.140485 |
| ENSG00000214456.8 | 6.15E-77 | 6.2055957 | 11.601118 | -5.395522 |
| ENSG00000135740.16 | 3.57E-39 | 5.8394172 | 7.1940886 | -1.354671 |
| ENSG00000131080.14 | 3.98E-46 | 6.0900726 | 8.1592909 | -2.069218 |
| ENSG00000181656.6 | 7.76E-34 | 3.1558527 | 6.189783 | -3.03393 |
| ENSG00000222019.7 | 4.31E-43 | 6.084999 | 7.6365284 | -1.551529 |
| ENSG00000254226.5 | 3.32E-56 | 1.9567439 | 4.5688784 | -2.612134 |
| ENSG00000258679.1 | 6.98E-46 | 0.7522033 | 5.5473795 | -4.795176 |
| ENSG00000224747.1 | 6.23E-38 | 0.6783243 | 2.4951841 | -1.81686 |
| ENSG00000223893.1 | 5.18E-16 | 0.193852 | 1.4289477 | -1.235096 |
| ENSG00000277435.1 | 1.27E-26 | 0.8262411 | 2.7690205 | -1.942779 |
| ENSG00000246985.7 | 1.27E-43 | 3.9905776 | 6.193967 | -2.203389 |
| ENSG00000141448.8 | 2.07E-21 | 10.596226 | 11.96528 | -1.369054 |
| ENSG00000184617.10 | 3.79E-29 | 1.673668 | 3.0098216 | -1.336154 |
| ENSG00000184786.5 | 6.90E-30 | 6.5600625 | 7.7837409 | -1.223678 |
| ENSG00000070388.11 | 7.40E-83 | 2.1624962 | 6.1935943 | -4.031098 |
| ENSG00000198483.12 | 1.20E-59 | 5.4750971 | 7.7696011 | -2.294504 |
| ENSG00000135824.12 | 4.14E-11 | 2.1401597 | 3.3051614 | -1.165002 |
| ENSG00000261584.1 | 1.42E-15 | 6.3930766 | 7.4520761 | -1.059 |
| ENSG00000234290.2 | 2.58E-29 | 6.4998322 | 7.5096966 | -1.009864 |
| ENSG00000267784.1 | 2.75E-100 | 0.8594234 | 4.3032977 | -3.443874 |
| ENSG00000261505.1 | 1.64E-62 | 7.1489644 | 8.8986989 | -1.749734 |
| ENSG00000187955.11 | 4.88E-66 | 9.6228434 | 12.227853 | -2.60501 |
| ENSG00000241399.6 | 7.88E-39 | 8.8040742 | 10.221723 | -1.417649 |
| ENSG00000121361.3 | 7.45E-27 | 8.0505301 | 9.1945807 | -1.144051 |
| ENSG00000075651.15 | 5.01E-74 | 8.7053313 | 10.387619 | -1.682288 |
| ENSG00000223774.5 | 1.10E-47 | 1.0071251 | 3.0173284 | -2.010203 |
| ENSG00000225891.1 | 7.57E-31 | 0.8078294 | 1.9754989 | -1.16767 |
| ENSG00000155093.17 | 2.32E-57 | 6.9333874 | 8.7204307 | -1.787043 |
| ENSG00000279833.1 | 7.31E-130 | 4.3296905 | 7.6950068 | -3.365316 |
| ENSG00000215302.8 | 3.17E-31 | 4.7104021 | 5.9924636 | -1.282061 |
| ENSG00000237821.1 | 2.69E-21 | 3.0466652 | 4.1625932 | -1.115928 |
| ENSG00000151914.17 | 1.52E-119 | 10.481277 | 12.986289 | -2.505011 |
| ENSG00000225125.2 | 1.04E-56 | 2.9917582 | 5.1862932 | -2.194535 |
| ENSG00000249476.1 | 1.04E-66 | 3.1854516 | 5.3212614 | -2.13581 |
| ENSG00000164125.15 | 6.17E-61 | 10.011726 | 12.090085 | -2.078359 |
| ENSG00000165521.15 | 2.64E-28 | 8.8492284 | 10.441356 | -1.592127 |
| ENSG00000225649.5 | 1.28E-63 | 1.8765332 | 5.4461795 | -3.569646 |
| ENSG00000274248.1 | 1.05E-24 | 1.3378251 | 2.9635102 | -1.625685 |
| ENSG00000072952.18 | 6.61E-30 | 8.8290437 | 10.613152 | -1.784109 |
| ENSG00000157110.15 | 7.50E-69 | 10.827287 | 12.653256 | -1.825968 |
| ENSG00000177990.11 | 3.51E-130 | 6.2225396 | 9.917917 | -3.695377 |
| ENSG00000259807.1 | 4.77E-25 | 3.2732413 | 4.6098875 | -1.336646 |
| ENSG00000183251.3 | 2.21E-16 | 0.5205575 | 1.5332602 | -1.012703 |
| ENSG00000254667.2 | 3.35E-21 | 3.2169341 | 4.7856773 | -1.568743 |
| ENSG00000082397.15 | 1.46E-21 | 8.7397117 | 9.8357693 | -1.096058 |
| ENSG00000175084.11 | 2.30E-33 | 8.6114169 | 11.485673 | -2.874256 |
| ENSG00000114923.16 | 2.38E-56 | 9.8146816 | 13.052624 | -3.237942 |
| ENSG00000274341.1 | 3.15E-20 | 1.1531742 | 2.2189864 | -1.065812 |
| ENSG00000117594.9 | 1.44E-49 | 6.2438692 | 9.6197489 | -3.37588 |
| ENSG00000154188.9 | 2.74E-23 | 6.2999098 | 7.668942 | -1.369032 |
| ENSG00000196616.12 | 2.42E-75 | 5.562716 | 12.01626 | -6.453544 |
| ENSG00000231329.7 | 6.72E-47 | 1.6493055 | 3.573525 | -1.92422 |
| ENSG00000232196.3 | 6.90E-41 | 1.5645835 | 3.9344659 | -2.369882 |
| ENSG00000133392.16 | 1.65E-56 | 8.697726 | 13.283131 | -4.585405 |
| ENSG00000111405.8 | 5.10E-17 | 4.8876453 | 6.1616364 | -1.273991 |
| ENSG00000267655.1 | 1.49E-23 | 3.100406 | 4.3415216 | -1.241116 |
| ENSG00000280239.1 | 5.55E-62 | 5.706305 | 7.1942932 | -1.487988 |
| ENSG00000203392.3 | 4.59E-38 | 5.2406778 | 6.5833284 | -1.342651 |
| ENSG00000169439.11 | 2.10E-48 | 10.676017 | 12.294367 | -1.61835 |
| ENSG00000227253.3 | 1.21E-20 | 2.2663308 | 3.9554807 | -1.68915 |
| ENSG00000227063.5 | 1.39E-15 | 0.5242317 | 1.6039023 | -1.079671 |
| ENSG00000276923.1 | 1.98E-52 | 0.6061356 | 3.4242432 | -2.818108 |
| ENSG00000259219.1 | 1.80E-26 | 1.6354597 | 2.7832023 | -1.147743 |
| ENSG00000230795.2 | 1.55E-24 | 4.4364492 | 6.0747977 | -1.638349 |
| ENSG00000151834.15 | 3.22E-26 | 1.3366072 | 3.8188727 | -2.482266 |
| ENSG00000204065.2 | 2.63E-20 | 4.5698057 | 6.0671739 | -1.497368 |
| ENSG00000233608.3 | 1.16E-56 | 6.9291279 | 9.3880216 | -2.458894 |
| ENSG00000248290.1 | 2.12E-27 | 2.3150377 | 5.2182659 | -2.903228 |
| ENSG00000142319.17 | 5.47E-26 | 2.8147675 | 4.6986432 | -1.883876 |
| ENSG00000188738.13 | 1.29E-48 | 4.9670733 | 6.7246807 | -1.757607 |
| ENSG00000186204.14 | 3.82E-48 | 4.2865936 | 7.8840148 | -3.597421 |
| ENSG00000122870.11 | 6.86E-44 | 8.4111401 | 9.9265227 | -1.515383 |
| ENSG00000234961.1 | 2.19E-27 | 4.0977933 | 5.5346409 | -1.436848 |
| ENSG00000262700.1 | 8.60E-18 | 1.4449126 | 2.534425 | -1.089512 |
| ENSG00000257043.1 | 1.60E-26 | 1.0881795 | 2.7913409 | -1.703161 |
| ENSG00000154803.12 | 5.82E-30 | 10.196446 | 11.33498 | -1.138534 |
| ENSG00000279342.1 | 2.11E-26 | 3.5515203 | 4.7962943 | -1.244774 |
| ENSG00000233334.3 | 4.16E-44 | 1.0702196 | 3.1407273 | -2.070508 |
| ENSG00000168010.10 | 3.07E-52 | 9.2791442 | 11.092681 | -1.813537 |
| ENSG00000168314.17 | 3.55E-12 | 3.0828489 | 4.2723284 | -1.189479 |
| ENSG00000163072.14 | 1.83E-50 | 6.9029582 | 8.4458932 | -1.542935 |
| ENSG00000166432.14 | 3.48E-124 | 7.4829709 | 10.808007 | -3.325036 |
| ENSG00000118777.10 | 4.94E-38 | 6.6835933 | 7.9620341 | -1.278441 |
| ENSG00000259976.1 | 1.14E-82 | 8.4858475 | 10.310872 | -1.825024 |
| ENSG00000259433.2 | 2.24E-32 | 0.4014687 | 3.1726295 | -2.771161 |
| ENSG00000077522.12 | 8.70E-68 | 3.7121353 | 6.5723102 | -2.860175 |
| ENSG00000217455.8 | 8.52E-09 | 1.9210957 | 3.3261227 | -1.405027 |
| ENSG00000197565.15 | 2.40E-68 | 6.5039098 | 9.7149386 | -3.211029 |
| ENSG00000123999.4 | 2.65E-47 | 6.6513833 | 10.754036 | -4.102653 |
| ENSG00000176769.9 | 1.27E-52 | 2.9657465 | 6.521492 | -3.555746 |
| ENSG00000102547.18 | 4.00E-20 | 8.8655408 | 9.9542091 | -1.088668 |
| ENSG00000235209.1 | 6.58E-12 | 1.0673375 | 2.1405227 | -1.073185 |
| ENSG00000214940.8 | 2.03E-08 | 4.4830601 | 6.4819739 | -1.998914 |
| ENSG00000166206.13 | 5.16E-17 | 5.0920811 | 6.4017466 | -1.309665 |
| ENSG00000125430.8 | 8.70E-21 | 6.6246888 | 8.0721636 | -1.447475 |
| ENSG00000235703.5 | 3.92E-70 | 6.7569499 | 8.8799977 | -2.123048 |
| ENSG00000228107.1 | 2.17E-19 | 2.7248558 | 4.1118545 | -1.386999 |
| ENSG00000260306.1 | 1.39E-58 | 4.2717518 | 6.6764705 | -2.404719 |
| ENSG00000223611.5 | 6.98E-25 | 0.6237305 | 2.3999784 | -1.776248 |
| ENSG00000204338.8 | 2.22E-20 | 5.091989 | 6.8963227 | -1.804334 |
| ENSG00000198106.8 | 2.68E-98 | 4.8215936 | 7.9173716 | -3.095778 |
| ENSG00000137077.7 | 8.64E-12 | 5.5980098 | 7.5377966 | -1.939787 |
| ENSG00000236991.6 | 1.03E-49 | 2.1454974 | 3.7667057 | -1.621208 |
| ENSG00000111245.14 | 5.74E-27 | 0.9410399 | 4.2190352 | -3.277995 |
| ENSG00000224153.2 | 2.55E-34 | 0.3254931 | 2.069933 | -1.74444 |
| ENSG00000122863.5 | 2.23E-45 | 9.7571146 | 11.167095 | -1.409981 |
| ENSG00000254647.6 | 5.12E-19 | 0.0627473 | 1.389517 | -1.32677 |
| ENSG00000105695.14 | 2.59E-33 | 7.0378401 | 9.6922636 | -2.654424 |
| ENSG00000121310.16 | 1.11E-65 | 10.99552 | 13.183519 | -2.187999 |
| ENSG00000272054.1 | 5.36E-53 | 5.4605372 | 6.742658 | -1.282121 |
| ENSG00000280285.1 | 1.51E-11 | 1.2681673 | 2.4548727 | -1.186705 |
| ENSG00000260425.1 | 5.17E-15 | 0.0191179 | 1.518692 | -1.499574 |
| ENSG00000129757.12 | 2.32E-25 | 10.717757 | 12.011816 | -1.294059 |
| ENSG00000173991.5 | 2.40E-26 | 5.1896847 | 6.3494614 | -1.159777 |
| ENSG00000149557.12 | 8.32E-38 | 7.5575525 | 8.7012523 | -1.1437 |
| ENSG00000109819.8 | 1.21E-09 | 4.7544301 | 5.9024864 | -1.148056 |
| ENSG00000204740.9 | 2.10E-27 | 2.4256974 | 4.4479443 | -2.022247 |
| ENSG00000278991.1 | 6.05E-30 | 2.4497191 | 3.7129773 | -1.263258 |
| ENSG00000265678.1 | 7.18E-35 | 3.7715322 | 4.8685341 | -1.097002 |
| ENSG00000230725.5 | 3.45E-33 | 1.8524692 | 3.7509409 | -1.898472 |
| ENSG00000198358.4 | 2.75E-29 | 0.1879158 | 2.2682682 | -2.080352 |
| ENSG00000139675.11 | 5.44E-59 | 7.5193172 | 8.6689318 | -1.149615 |
| ENSG00000281026.1 | 2.72E-89 | 5.6680229 | 8.4426352 | -2.774612 |
| ENSG00000110318.13 | 1.45E-97 | 7.1230816 | 9.3937727 | -2.270691 |
| ENSG00000132872.11 | 4.37E-26 | 1.3597186 | 4.009317 | -2.649598 |
| ENSG00000260548.1 | 9.56E-29 | 0.6413236 | 2.4066989 | -1.765375 |
| ENSG00000280789.1 | 1.29E-26 | 10.671835 | 11.857033 | -1.185198 |
| ENSG00000277726.4 | 4.42E-10 | 1.7517642 | 2.8051102 | -1.053346 |
| ENSG00000183929.7 | 9.50E-48 | 1.5227979 | 4.7480273 | -3.225229 |
| ENSG00000228492.2 | 1.48E-36 | 6.369584 | 7.6023477 | -1.232764 |
| ENSG00000133519.12 | 1.78E-42 | 7.462889 | 10.664991 | -3.202102 |
| ENSG00000223403.3 | 2.15E-66 | 3.4412551 | 8.6338273 | -5.192572 |
| ENSG00000183458.13 | 8.64E-50 | 10.014666 | 11.397223 | -1.382557 |
| ENSG00000264204.2 | 2.58E-08 | 4.3104169 | 5.3961295 | -1.085713 |
| ENSG00000128881.16 | 2.91E-56 | 8.8406926 | 9.8788636 | -1.038171 |
| ENSG00000124479.8 | 1.00E-12 | 7.3488148 | 8.6325432 | -1.283728 |
| ENSG00000102878.15 | 1.16E-54 | 8.3275475 | 10.530933 | -2.203385 |
| ENSG00000163833.7 | 2.72E-30 | 0.8600041 | 3.1810261 | -2.321022 |
| ENSG00000177875.4 | 5.04E-27 | 6.3063907 | 7.8425148 | -1.536124 |
| ENSG00000138685.12 | 4.82E-165 | 6.3523699 | 9.7330477 | -3.380678 |
| ENSG00000228962.1 | 1.03E-30 | 1.9305315 | 3.3180716 | -1.38754 |
| ENSG00000233025.1 | 2.33E-18 | 2.836142 | 3.9464409 | -1.110299 |
| ENSG00000215218.3 | 1.39E-13 | 5.5858924 | 6.9550034 | -1.369111 |
| ENSG00000258489.3 | 1.29E-68 | 0.4388465 | 3.3464625 | -2.907616 |
| ENSG00000230470.1 | 5.31E-16 | 0.1523241 | 1.279267 | -1.126943 |
| ENSG00000138134.11 | 9.13E-41 | 7.0574967 | 8.7011841 | -1.643687 |
| ENSG00000173175.14 | 1.02E-23 | 9.3862745 | 10.441433 | -1.055158 |
| ENSG00000242941.1 | 2.92E-31 | 0.3616425 | 2.2008386 | -1.839196 |
| ENSG00000169554.16 | 1.69E-100 | 9.6048594 | 11.644324 | -2.039464 |
| ENSG00000166313.18 | 2.39E-71 | 9.7090372 | 11.064788 | -1.35575 |
| ENSG00000270580.5 | 3.66E-50 | 8.2881718 | 9.7394557 | -1.451284 |
| ENSG00000156920.10 | 3.46E-18 | 0.380084 | 3.46655 | -3.086466 |
| ENSG00000270433.1 | 4.61E-23 | 1.9457224 | 3.2580977 | -1.312375 |
| ENSG00000109906.13 | 1.24E-62 | 5.6516348 | 9.7552636 | -4.103629 |
| ENSG00000196660.10 | 2.28E-25 | 1.9751711 | 4.9739989 | -2.998828 |
| ENSG00000259658.3 | 7.26E-96 | 3.4695391 | 6.2779 | -2.808361 |
| ENSG00000124224.16 | 1.38E-43 | 7.9791375 | 9.1046045 | -1.125467 |
| ENSG00000013441.15 | 5.99E-63 | 11.194837 | 12.75769 | -1.562853 |
| ENSG00000234750.1 | 3.28E-20 | 2.1810802 | 3.2774386 | -1.096358 |
| ENSG00000280140.1 | 6.48E-22 | 0.5777537 | 1.7082216 | -1.130468 |
| ENSG00000172403.10 | 4.03E-103 | 8.9681504 | 11.966086 | -2.997936 |
| ENSG00000132906.17 | 1.42E-15 | 9.6721294 | 10.728577 | -1.056448 |
| ENSG00000269352.1 | 4.86E-64 | 6.617837 | 8.0107239 | -1.392887 |
| ENSG00000235875.3 | 2.10E-36 | 0.323132 | 2.7349273 | -2.411795 |
| ENSG00000219085.1 | 6.78E-18 | 0.9768998 | 2.1692807 | -1.192381 |
| ENSG00000224238.2 | 6.50E-21 | 2.183301 | 3.5508409 | -1.36754 |
| ENSG00000267125.2 | 4.07E-81 | 0.0760969 | 4.6702841 | -4.594187 |
| ENSG00000273291.5 | 6.55E-54 | 3.4155124 | 5.5887136 | -2.173201 |
| ENSG00000262061.5 | 1.28E-49 | 5.3067537 | 8.1494943 | -2.842741 |
| ENSG00000254648.1 | 1.31E-16 | 2.0957399 | 3.3434534 | -1.247714 |
| ENSG00000262075.3 | 3.31E-36 | 2.2246692 | 3.9987227 | -1.774054 |
| ENSG00000259132.1 | 2.44E-21 | 3.7707714 | 6.3071148 | -2.536343 |
| ENSG00000247765.2 | 3.79E-35 | 2.6139146 | 5.078025 | -2.46411 |
| ENSG00000197608.11 | 1.16E-56 | 8.4866916 | 9.680992 | -1.1943 |
| ENSG00000145246.13 | 7.14E-47 | 8.6749964 | 9.7053045 | -1.030308 |
| ENSG00000101251.11 | 2.67E-10 | 1.6492998 | 2.7397239 | -1.090424 |
| ENSG00000129244.8 | 2.27E-50 | 7.7805229 | 10.074582 | -2.294059 |
| ENSG00000262477.1 | 9.15E-32 | 4.7716833 | 6.020208 | -1.248525 |
| ENSG00000177627.9 | 4.38E-34 | 1.1488291 | 3.320592 | -2.171763 |
| ENSG00000261240.1 | 1.28E-34 | 1.2708735 | 2.7411045 | -1.470231 |
| ENSG00000124212.5 | 4.88E-29 | 10.050154 | 11.631657 | -1.581503 |
| ENSG00000114790.12 | 2.45E-44 | 7.3780332 | 8.6672602 | -1.289227 |
| ENSG00000181074.3 | 5.05E-55 | 1.3381871 | 4.2735045 | -2.935317 |
| ENSG00000161180.10 | 3.72E-86 | 2.7878986 | 5.085808 | -2.297909 |
| ENSG00000111058.7 | 7.91E-28 | 9.2156377 | 10.258959 | -1.043321 |
| ENSG00000215493.3 | 2.81E-16 | 4.2241165 | 5.3682011 | -1.144085 |
| ENSG00000255390.1 | 6.95E-41 | 5.4475666 | 7.4348364 | -1.98727 |
| ENSG00000142303.13 | 2.48E-72 | 7.8920217 | 9.8877057 | -1.995684 |
| ENSG00000214140.10 | 2.33E-35 | 4.3574816 | 6.4336977 | -2.076216 |
| ENSG00000102230.13 | 1.04E-15 | 5.5791716 | 6.5926341 | -1.013462 |
| ENSG00000273203.1 | 7.90E-57 | 4.3699179 | 7.2265307 | -2.856613 |
| ENSG00000168079.16 | 5.04E-55 | 3.3375442 | 8.7229341 | -5.38539 |
| ENSG00000153157.12 | 3.00E-27 | 3.8722382 | 5.3961318 | -1.523894 |
| ENSG00000161649.12 | 9.21E-22 | 3.0045635 | 5.2974807 | -2.292917 |
| ENSG00000225472.1 | 4.71E-12 | 2.0324706 | 3.0739818 | -1.041511 |
| ENSG00000273771.1 | 1.25E-148 | 2.47751 | 8.3968852 | -5.919375 |
| ENSG00000172348.14 | 2.81E-24 | 8.4024086 | 9.7010625 | -1.298654 |
| ENSG00000227467.3 | 1.70E-14 | 1.9124601 | 3.1848 | -1.27234 |
| ENSG00000224413.1 | 1.14E-69 | 2.5346208 | 6.0999159 | -3.565295 |
| ENSG00000251348.1 | 4.00E-66 | 0.8644993 | 5.1749364 | -4.310437 |
| ENSG00000144642.20 | 2.62E-97 | 8.0651019 | 10.27933 | -2.214228 |
| ENSG00000172346.14 | 4.94E-182 | 7.2588162 | 12.478765 | -5.219949 |
| ENSG00000267042.1 | 1.09E-28 | 0.8272294 | 3.508342 | -2.681113 |
| ENSG00000274810.3 | 3.40E-13 | 5.7158033 | 6.7246523 | -1.008849 |
| ENSG00000148935.10 | 3.90E-28 | 4.2703976 | 5.6843068 | -1.413909 |
| ENSG00000089199.9 | 9.42E-31 | 4.4616208 | 8.4106489 | -3.949028 |
| ENSG00000056487.15 | 6.43E-146 | 2.8654759 | 7.7009386 | -4.835463 |
| ENSG00000272668.1 | 6.02E-24 | 5.2074981 | 6.826908 | -1.61941 |
| ENSG00000259363.5 | 2.56E-59 | 3.6337644 | 5.3809057 | -1.747141 |
| ENSG00000222012.1 | 1.53E-52 | 1.839731 | 4.9546318 | -3.114901 |
| ENSG00000223374.1 | 1.86E-68 | 2.7506258 | 4.7061182 | -1.955492 |
| ENSG00000248409.1 | 2.06E-38 | 1.5492613 | 4.5909511 | -3.04169 |
| ENSG00000280039.1 | 1.38E-37 | 4.4240988 | 5.6325477 | -1.208449 |
| ENSG00000108773.10 | 1.10E-30 | 11.635779 | 12.720209 | -1.08443 |
| ENSG00000205363.5 | 6.46E-64 | 5.914537 | 8.8946943 | -2.980157 |
| ENSG00000196123.12 | 2.83E-94 | 9.2068928 | 11.901011 | -2.694119 |
| ENSG00000242853.3 | 1.15E-20 | 0.1472394 | 1.5136648 | -1.366425 |
| ENSG00000183722.7 | 8.97E-23 | 9.0492721 | 10.391925 | -1.342653 |
| ENSG00000161643.12 | 8.63E-62 | 4.9098831 | 9.8481739 | -4.938291 |
| ENSG00000244056.3 | 2.72E-70 | 0.9849635 | 3.9083375 | -2.923374 |
| ENSG00000161551.12 | 1.51E-68 | 8.4297062 | 10.265975 | -1.836269 |
| ENSG00000224729.5 | 2.01E-38 | 4.3499193 | 6.0470534 | -1.697134 |
| ENSG00000254744.3 | 4.17E-30 | 0.8142611 | 3.0570807 | -2.24282 |
| ENSG00000274295.1 | 5.84E-51 | 1.2440415 | 4.4626591 | -3.218618 |
| ENSG00000100181.21 | 5.74E-33 | 6.3616212 | 8.63785 | -2.276229 |
| ENSG00000213366.12 | 2.03E-64 | 10.869237 | 13.262169 | -2.392932 |
| ENSG00000281852.1 | 1.28E-97 | 4.6836897 | 8.1463239 | -3.462634 |
| ENSG00000226491.1 | 3.73E-61 | 2.1938399 | 4.3093761 | -2.115536 |
| ENSG00000260597.1 | 5.74E-85 | 2.4666432 | 6.7437193 | -4.277076 |
| ENSG00000147234.10 | 5.83E-47 | 4.5324831 | 7.848325 | -3.315842 |
| ENSG00000168925.10 | 6.71E-35 | 0.5901232 | 4.7641148 | -4.173992 |
| ENSG00000169085.11 | 1.38E-24 | 4.9517172 | 6.7622886 | -1.810571 |
| ENSG00000254639.1 | 1.69E-23 | 0.427189 | 1.6028909 | -1.175702 |
| ENSG00000123843.12 | 1.81E-57 | 2.6721816 | 9.7185591 | -7.046377 |
| ENSG00000138834.12 | 2.19E-56 | 11.133942 | 12.463397 | -1.329455 |
| ENSG00000203685.9 | 1.25E-26 | 6.1625403 | 8.0529909 | -1.890451 |
| ENSG00000277214.1 | 1.12E-54 | 0.6237477 | 3.4018011 | -2.778053 |
| ENSG00000116254.17 | 1.15E-30 | 3.244142 | 5.501608 | -2.257466 |
| ENSG00000235661.2 | 9.70E-23 | 0.1055062 | 1.9488182 | -1.843312 |
| ENSG00000257763.1 | 1.01E-28 | 1.2168442 | 2.7169864 | -1.500142 |
| ENSG00000233665.8 | 5.07E-65 | 0.9970723 | 3.5558398 | -2.558767 |
| ENSG00000111339.10 | 1.73E-14 | 2.1492578 | 3.6629466 | -1.513689 |
| ENSG00000264635.1 | 1.74E-24 | 1.5294687 | 2.6889727 | -1.159504 |
| ENSG00000117020.16 | 7.80E-100 | 8.9725453 | 11.510502 | -2.537957 |
| ENSG00000223509.8 | 1.32E-68 | 6.3457496 | 8.1000739 | -1.754324 |
| ENSG00000149634.4 | 3.55E-38 | 4.3927518 | 5.5917227 | -1.198971 |
| ENSG00000254152.1 | 4.69E-17 | 0.0492286 | 1.1436205 | -1.094392 |
| ENSG00000253250.2 | 4.23E-41 | 5.410022 | 6.884342 | -1.47432 |
| ENSG00000276409.4 | 1.79E-15 | 5.602921 | 7.683933 | -2.081012 |
| ENSG00000123561.14 | 9.50E-21 | 0.0721413 | 1.5129205 | -1.440779 |
| ENSG00000269570.2 | 2.27E-40 | 2.2598193 | 3.7686773 | -1.508858 |
| ENSG00000230393.1 | 4.32E-61 | 1.9654644 | 4.4420318 | -2.476567 |
| ENSG00000128285.4 | 5.52E-38 | 3.6301298 | 6.4608568 | -2.830727 |
| ENSG00000274049.4 | 3.18E-24 | 7.4116742 | 8.948308 | -1.536634 |
| ENSG00000266820.1 | 1.03E-57 | 5.2525924 | 7.0566966 | -1.804104 |
| ENSG00000164484.11 | 2.40E-33 | 7.4735661 | 9.1474648 | -1.673899 |
| ENSG00000260784.1 | 4.08E-27 | 2.295253 | 4.154417 | -1.859164 |
| ENSG00000229694.6 | 2.01E-17 | 1.7767919 | 2.8287523 | -1.05196 |
| ENSG00000223842.1 | 1.05E-19 | 0.6800678 | 1.8317625 | -1.151695 |
| ENSG00000166816.13 | 3.57E-52 | 7.1498272 | 9.039817 | -1.88999 |
| ENSG00000115593.14 | 9.95E-13 | 0.6389642 | 1.7142216 | -1.075257 |
| ENSG00000269699.5 | 1.23E-95 | 2.7158532 | 6.9246955 | -4.208842 |
| ENSG00000239908.3 | 3.96E-24 | 0.5099449 | 1.6593511 | -1.149406 |
| ENSG00000254419.1 | 9.29E-24 | 1.8824348 | 2.9638148 | -1.08138 |
| ENSG00000144035.3 | 1.72E-41 | 1.9186623 | 3.8856511 | -1.966989 |
| ENSG00000224017.1 | 4.25E-21 | 0.5276461 | 1.5416466 | -1.014001 |
| ENSG00000230982.1 | 5.64E-28 | 3.2315029 | 4.3890455 | -1.157543 |
| ENSG00000183793.13 | 5.84E-18 | 7.2927914 | 9.1567852 | -1.863994 |
| ENSG00000153902.13 | 1.55E-38 | 5.8735131 | 8.692508 | -2.818995 |
| ENSG00000270903.1 | 1.32E-22 | 3.2987726 | 4.5347489 | -1.235976 |
| ENSG00000160339.15 | 7.09E-24 | 1.3169979 | 2.7468977 | -1.4299 |
| ENSG00000213706.2 | 2.05E-28 | 1.0461988 | 2.290908 | -1.244709 |
| ENSG00000133142.17 | 4.91E-54 | 11.672395 | 13.611364 | -1.938969 |
| ENSG00000254966.1 | 4.76E-62 | 3.5529191 | 5.4572614 | -1.904342 |
| ENSG00000122824.10 | 1.94E-58 | 5.7462358 | 8.6475205 | -2.901285 |
| ENSG00000197457.9 | 1.50E-27 | 9.5817792 | 10.727932 | -1.146153 |
| ENSG00000244257.5 | 1.80E-20 | 9.384442 | 11.018026 | -1.633584 |
| ENSG00000229152.2 | 7.45E-66 | 5.8606332 | 7.6079295 | -1.747296 |
| ENSG00000189212.12 | 2.90E-23 | 3.291326 | 4.6653898 | -1.374064 |
| ENSG00000245526.8 | 3.77E-18 | 1.6510654 | 3.2235841 | -1.572519 |
| ENSG00000203812.2 | 1.04E-48 | 3.093037 | 7.9302523 | -4.837215 |
| ENSG00000224431.1 | 5.31E-21 | 2.4335773 | 3.9237511 | -1.490174 |
| ENSG00000225930.3 | 1.03E-26 | 1.3986587 | 3.5180761 | -2.119417 |
| ENSG00000166793.10 | 5.63E-40 | 6.4276878 | 8.9563386 | -2.528651 |
| ENSG00000228983.8 | 9.73E-28 | 0.0531122 | 1.5306545 | -1.477542 |
| ENSG00000166450.12 | 6.66E-42 | 5.9832551 | 7.6497432 | -1.666488 |
| ENSG00000269289.5 | 2.32E-11 | 2.4000119 | 3.8317795 | -1.431768 |
| ENSG00000241416.1 | 1.31E-35 | 0.7642296 | 2.8653375 | -2.101108 |
| ENSG00000241111.1 | 6.24E-46 | 1.1414193 | 3.8491864 | -2.707767 |
| ENSG00000242034.1 | 4.64E-27 | 0.6846489 | 2.3112455 | -1.626597 |
| ENSG00000164344.15 | 2.48E-19 | 2.9239014 | 4.2745795 | -1.350678 |
| ENSG00000174740.7 | 4.89E-87 | 4.5189179 | 7.377725 | -2.858807 |
| ENSG00000137504.13 | 6.38E-65 | 10.895588 | 12.0887 | -1.193112 |
| ENSG00000103196.11 | 2.36E-09 | 9.9639284 | 11.398 | -1.434072 |
| ENSG00000165359.14 | 2.11E-73 | 8.6819315 | 10.019736 | -1.337805 |
| ENSG00000270589.1 | 3.03E-54 | 4.029948 | 5.7377477 | -1.7078 |
| ENSG00000078814.15 | 2.43E-34 | 6.0410286 | 7.6762159 | -1.635187 |
| ENSG00000175414.6 | 2.40E-35 | 8.6060976 | 9.6376023 | -1.031505 |
| ENSG00000137869.13 | 4.96E-12 | 3.5212277 | 5.4363545 | -1.915127 |
| ENSG00000258232.2 | 8.20E-138 | 0.9762695 | 7.346842 | -6.370573 |
| ENSG00000067445.20 | 2.17E-45 | 9.7691461 | 11.444251 | -1.675105 |
| ENSG00000240207.6 | 5.70E-18 | 5.3656678 | 6.38705 | -1.021382 |
| ENSG00000156564.8 | 1.24E-55 | 1.4009196 | 4.4898841 | -3.088965 |
| ENSG00000256870.2 | 7.24E-29 | 0.4787446 | 4.4373284 | -3.958584 |
| ENSG00000141449.14 | 2.46E-89 | 5.2362585 | 8.7113386 | -3.47508 |
| ENSG00000185203.11 | 2.29E-08 | 1.5327938 | 2.8145352 | -1.281741 |
| ENSG00000263400.6 | 9.99E-38 | 3.7846508 | 5.0477636 | -1.263113 |
| ENSG00000232057.1 | 1.16E-14 | 0.8932021 | 2.8422159 | -1.949014 |
| ENSG00000279488.1 | 2.83E-80 | 4.7528382 | 7.1769409 | -2.424103 |
| ENSG00000147437.9 | 1.81E-62 | 4.8569403 | 7.3182114 | -2.461271 |
| ENSG00000225880.5 | 1.07E-41 | 5.4025084 | 7.1810455 | -1.778537 |
| ENSG00000277299.1 | 7.31E-18 | 3.668079 | 4.7930455 | -1.124966 |
| ENSG00000267272.5 | 2.28E-29 | 7.2783874 | 8.3718455 | -1.093458 |
| ENSG00000095739.10 | 3.05E-66 | 7.2346549 | 10.572824 | -3.338169 |
| ENSG00000185689.15 | 3.31E-61 | 2.2616081 | 4.2730148 | -2.011407 |
| ENSG00000235370.6 | 3.35E-95 | 4.485295 | 7.4253307 | -2.940036 |
| ENSG00000154874.14 | 6.96E-93 | 4.3561845 | 7.8986602 | -3.542476 |
| ENSG00000163216.6 | 1.36E-14 | 0.4322761 | 2.0627227 | -1.630447 |
| ENSG00000260760.1 | 1.50E-23 | 0.2713523 | 2.5722375 | -2.300885 |
| ENSG00000277351.1 | 4.03E-16 | 2.0954205 | 3.3162568 | -1.220836 |
| ENSG00000263986.1 | 6.50E-18 | 3.5311761 | 5.8029852 | -2.271809 |
| ENSG00000113240.12 | 9.99E-52 | 9.2367752 | 10.282993 | -1.046218 |
| ENSG00000269994.1 | 1.70E-22 | 2.7521661 | 4.2851375 | -1.532971 |
| ENSG00000196696.12 | 1.72E-34 | 8.9979253 | 10.063255 | -1.065329 |
| ENSG00000169347.16 | 1.76E-38 | 0.2058549 | 5.1512989 | -4.945444 |
| ENSG00000259694.1 | 2.83E-24 | 0.6495554 | 1.6735489 | -1.023993 |
| ENSG00000205221.12 | 3.42E-127 | 3.1609835 | 9.3151159 | -6.154132 |
| ENSG00000185052.11 | 1.90E-30 | 8.3216623 | 9.7101932 | -1.388531 |
| ENSG00000273442.1 | 2.43E-44 | 1.8089878 | 4.312133 | -2.503145 |
| ENSG00000158815.10 | 6.25E-12 | 4.7208652 | 5.8867375 | -1.165872 |
| ENSG00000144331.18 | 1.70E-45 | 4.2595131 | 8.229333 | -3.96982 |
| ENSG00000177374.12 | 6.99E-53 | 8.840694 | 10.554234 | -1.71354 |
| ENSG00000280623.1 | 6.91E-30 | 4.2295971 | 7.0376489 | -2.808052 |
| ENSG00000224401.2 | 7.11E-35 | 0.8602036 | 2.3613023 | -1.501099 |
| ENSG00000163827.12 | 6.22E-61 | 5.4615184 | 8.3683295 | -2.906811 |
| ENSG00000136872.17 | 3.12E-39 | 2.121111 | 3.7939318 | -1.672821 |
| ENSG00000239899.3 | 8.77E-32 | 0.6011057 | 3.0235955 | -2.42249 |
| ENSG00000100024.14 | 4.54E-32 | 1.932022 | 3.7799784 | -1.847956 |
| ENSG00000239528.1 | 2.34E-20 | 0.859264 | 2.4184841 | -1.55922 |
| ENSG00000173401.9 | 2.13E-35 | 0.833006 | 2.239125 | -1.406119 |
| ENSG00000236013.5 | 2.47E-47 | 1.1611243 | 4.0945125 | -2.933388 |
| ENSG00000182230.11 | 7.04E-100 | 6.9051773 | 11.628924 | -4.723747 |
| ENSG00000164619.8 | 3.79E-77 | 4.4653045 | 7.3991318 | -2.933827 |
| ENSG00000265194.1 | 2.20E-48 | 3.6964649 | 5.786742 | -2.090277 |
| ENSG00000277715.1 | 4.86E-39 | 4.9252716 | 6.5669568 | -1.641685 |
| ENSG00000249228.1 | 1.68E-12 | 0.1390267 | 1.3464466 | -1.20742 |
| ENSG00000119938.8 | 4.76E-11 | 6.6978537 | 7.7697625 | -1.071909 |
| ENSG00000274925.1 | 2.82E-34 | 6.3432924 | 7.6375659 | -1.294274 |
| ENSG00000234353.2 | 4.11E-32 | 4.6845208 | 6.212467 | -1.527946 |
| ENSG00000263345.1 | 1.05E-41 | 4.1702358 | 5.552625 | -1.382389 |
| ENSG00000206530.8 | 2.16E-41 | 8.1913862 | 9.3732545 | -1.181868 |
| ENSG00000203801.8 | 7.06E-37 | 1.561257 | 3.5236023 | -1.962345 |
| ENSG00000271653.1 | 2.38E-09 | 1.2039086 | 2.2587182 | -1.05481 |
| ENSG00000171495.16 | 2.33E-24 | 0.3319026 | 1.5873511 | -1.255449 |
| ENSG00000145087.12 | 1.38E-99 | 3.4985795 | 9.1888977 | -5.690318 |
| ENSG00000227953.6 | 9.21E-21 | 5.981868 | 7.1588909 | -1.177023 |
| ENSG00000256223.5 | 2.22E-25 | 8.5594783 | 9.8873489 | -1.327871 |
| ENSG00000280893.1 | 1.23E-25 | 0.4527461 | 2.6469045 | -2.194158 |
| ENSG00000275695.1 | 6.96E-57 | 1.0676181 | 3.4010875 | -2.333469 |
| ENSG00000259324.1 | 1.78E-16 | 0.189552 | 1.470683 | -1.281131 |
| ENSG00000224858.5 | 3.07E-49 | 3.5044539 | 5.3684489 | -1.863995 |
| ENSG00000278959.1 | 1.49E-19 | 3.090022 | 4.3747136 | -1.284692 |
| ENSG00000228315.11 | 2.30E-120 | 8.5011697 | 11.048824 | -2.547654 |
| ENSG00000255245.3 | 1.02E-18 | 1.1683895 | 2.4124432 | -1.244054 |
| ENSG00000275443.1 | 6.37E-23 | 0.185105 | 1.4496352 | -1.26453 |
| ENSG00000128045.6 | 7.10E-42 | 7.3877274 | 10.579608 | -3.191881 |
| ENSG00000233578.1 | 3.00E-16 | 1.8999642 | 3.021067 | -1.121103 |
| ENSG00000212743.2 | 4.13E-26 | 3.5248616 | 4.9350364 | -1.410175 |
| ENSG00000157404.15 | 4.03E-60 | 6.3974251 | 10.138955 | -3.741529 |
| ENSG00000238061.2 | 3.82E-18 | 1.5516167 | 2.5882364 | -1.03662 |
| ENSG00000162461.7 | 1.28E-26 | 6.3127181 | 7.5252 | -1.212482 |
| ENSG00000273108.1 | 3.03E-50 | 3.7476117 | 5.7307216 | -1.98311 |
| ENSG00000229657.2 | 2.29E-42 | 0.5281866 | 2.8975977 | -2.369411 |
| ENSG00000262528.2 | 5.33E-47 | 3.3895601 | 5.4553773 | -2.065817 |
| ENSG00000280048.1 | 2.09E-52 | 1.7641678 | 4.4930148 | -2.728847 |
| ENSG00000103647.12 | 4.60E-19 | 8.6098475 | 9.6894216 | -1.079574 |
| ENSG00000122707.11 | 1.79E-43 | 8.3419165 | 9.4563102 | -1.114394 |
| ENSG00000273650.1 | 2.94E-40 | 1.1309726 | 3.1475852 | -2.016613 |
| ENSG00000253731.2 | 3.28E-41 | 6.9632599 | 8.2712511 | -1.307991 |
| ENSG00000106100.10 | 1.14E-27 | 8.8713265 | 9.9123773 | -1.041051 |
| ENSG00000198879.11 | 2.30E-79 | 7.9330587 | 9.8044375 | -1.871379 |
| ENSG00000081818.3 | 1.87E-52 | 6.3572663 | 7.8124114 | -1.455145 |
| ENSG00000079689.13 | 6.63E-20 | 0.2749179 | 2.0548739 | -1.779956 |
| ENSG00000116176.6 | 1.44E-70 | 0.8579391 | 4.7379409 | -3.880002 |
| ENSG00000196091.12 | 7.03E-23 | 1.5380084 | 4.4080114 | -2.870003 |
| ENSG00000234345.2 | 1.16E-69 | 0.6602852 | 3.8871216 | -3.226836 |
| ENSG00000230461.8 | 2.44E-42 | 0.9950735 | 2.963858 | -1.968784 |
| ENSG00000275966.1 | 3.28E-49 | 1.5141554 | 3.5207568 | -2.006601 |
| ENSG00000165633.12 | 1.30E-103 | 8.2585224 | 11.12101 | -2.862488 |
| ENSG00000261054.1 | 1.21E-08 | 2.7263687 | 3.8235057 | -1.097137 |
| ENSG00000271913.5 | 4.66E-31 | 3.9198983 | 5.1796023 | -1.259704 |
| ENSG00000259462.2 | 1.50E-81 | 1.9434902 | 5.4494261 | -3.505936 |
| ENSG00000240194.6 | 1.09E-14 | 0.2450141 | 1.5285205 | -1.283506 |
| ENSG00000126947.11 | 1.03E-47 | 9.0599666 | 10.201235 | -1.141269 |
| ENSG00000184206.11 | 2.19E-114 | 6.3545258 | 9.9423557 | -3.58783 |
| ENSG00000263232.2 | 2.87E-16 | 0.7852647 | 1.8221784 | -1.036914 |
| ENSG00000179914.4 | 3.29E-19 | 3.8921356 | 6.0024159 | -2.11028 |
| ENSG00000180660.7 | 1.73E-78 | 2.2392702 | 4.8121102 | -2.57284 |
| ENSG00000272316.1 | 6.21E-96 | 7.1250726 | 9.1576125 | -2.03254 |
| ENSG00000147588.6 | 9.92E-22 | 0.7563425 | 2.7560648 | -1.999722 |
| ENSG00000175147.11 | 8.19E-42 | 6.9790928 | 8.6330239 | -1.653931 |
| ENSG00000264943.1 | 6.44E-37 | 1.7970888 | 3.3854841 | -1.588395 |
| ENSG00000132170.19 | 4.19E-64 | 6.6843995 | 8.6053932 | -1.920994 |
| ENSG00000106483.11 | 2.89E-11 | 10.195646 | 11.272056 | -1.07641 |
| ENSG00000242479.1 | 5.09E-08 | 0.0969487 | 1.3359659 | -1.239017 |
| ENSG00000274297.1 | 5.66E-18 | 1.8540735 | 2.9666523 | -1.112579 |
| ENSG00000269958.1 | 9.09E-45 | 6.489694 | 7.4969773 | -1.007283 |
| ENSG00000189419.6 | 2.47E-58 | 3.6111513 | 5.4613432 | -1.850192 |
| ENSG00000167723.14 | 9.72E-35 | 4.8122375 | 6.585842 | -1.773605 |
| ENSG00000244094.1 | 3.97E-51 | 2.7895415 | 8.7234886 | -5.933947 |
| ENSG00000129422.13 | 5.06E-66 | 10.317592 | 11.73214 | -1.414547 |
| ENSG00000230565.1 | 2.38E-37 | 4.1650315 | 5.3006943 | -1.135663 |
| ENSG00000278192.1 | 3.76E-39 | 2.356084 | 4.2908182 | -1.934734 |
| ENSG00000106511.5 | 4.63E-27 | 3.5925737 | 5.9257102 | -2.333136 |
| ENSG00000153253.15 | 2.47E-25 | 3.1746351 | 5.6812648 | -2.50663 |
| ENSG00000265018.6 | 2.67E-21 | 4.6713365 | 6.7475318 | -2.076195 |
| ENSG00000260641.1 | 1.40E-50 | 2.6228838 | 5.3489955 | -2.726112 |
| ENSG00000234336.6 | 7.54E-34 | 2.6099716 | 4.4522932 | -1.842322 |
| ENSG00000160460.15 | 1.44E-61 | 6.9466067 | 8.7925284 | -1.845922 |
| ENSG00000112320.11 | 7.12E-63 | 8.1675289 | 9.8129545 | -1.645426 |
| ENSG00000123454.10 | 1.25E-33 | 2.8678637 | 4.3451227 | -1.477259 |
| ENSG00000134259.3 | 6.26E-74 | 5.1133826 | 8.0465148 | -2.933132 |
| ENSG00000267287.1 | 6.34E-30 | 4.1943862 | 6.2784159 | -2.08403 |
| ENSG00000106018.13 | 8.89E-133 | 5.2102234 | 9.3105875 | -4.100364 |
| ENSG00000214455.4 | 1.41E-32 | 6.7628487 | 7.8214023 | -1.058554 |
| ENSG00000118004.17 | 4.77E-34 | 6.7981673 | 9.5283375 | -2.73017 |
| ENSG00000259820.1 | 4.58E-38 | 7.6344683 | 8.9822148 | -1.347747 |
| ENSG00000132554.19 | 6.43E-74 | 5.669653 | 8.794758 | -3.125105 |
| ENSG00000253230.6 | 1.58E-12 | 0.2752208 | 1.5662932 | -1.291072 |
| ENSG00000111052.7 | 1.29E-82 | 5.7347964 | 8.0771011 | -2.342305 |
| ENSG00000183230.16 | 2.52E-45 | 3.2731062 | 5.7538523 | -2.480746 |
| ENSG00000168874.12 | 3.35E-33 | 7.6423484 | 9.5022386 | -1.85989 |
| ENSG00000140398.13 | 3.48E-92 | 8.4020788 | 10.293813 | -1.891734 |
| ENSG00000250961.1 | 1.23E-20 | 0.3140453 | 1.876275 | -1.56223 |
| ENSG00000258400.1 | 3.94E-18 | 0.5578912 | 1.5637864 | -1.005895 |
| ENSG00000004776.11 | 3.60E-59 | 10.501734 | 13.208393 | -2.706659 |
| ENSG00000122574.10 | 1.38E-52 | 8.501701 | 11.672323 | -3.170622 |
| ENSG00000278371.2 | 2.34E-19 | 0.4057203 | 1.8907602 | -1.48504 |
| ENSG00000248514.1 | 8.10E-23 | 0.3720014 | 1.6741989 | -1.302197 |
| ENSG00000119042.16 | 2.06E-34 | 6.1479212 | 7.3503114 | -1.20239 |
| ENSG00000170919.15 | 5.05E-38 | 9.4935835 | 10.573113 | -1.079529 |
| ENSG00000115641.18 | 3.26E-48 | 11.53592 | 13.350663 | -1.814742 |
| ENSG00000266896.1 | 3.92E-32 | 3.7439857 | 4.8800943 | -1.136109 |
| ENSG00000267328.1 | 1.17E-37 | 3.9354578 | 6.735658 | -2.8002 |
| ENSG00000274985.1 | 1.57E-31 | 0.5407878 | 3.2797057 | -2.738918 |
| ENSG00000077044.9 | 3.26E-37 | 9.7462952 | 10.871951 | -1.125656 |
| ENSG00000110080.18 | 1.41E-39 | 10.040057 | 11.39459 | -1.354533 |
| ENSG00000234043.3 | 7.17E-28 | 2.850664 | 4.2986636 | -1.448 |
| ENSG00000236031.1 | 2.62E-29 | 0.7937379 | 2.197567 | -1.403829 |
| ENSG00000274403.1 | 3.76E-88 | 2.3290153 | 4.8903955 | -2.56138 |
| ENSG00000026025.13 | 2.51E-36 | 15.450402 | 16.644798 | -1.194395 |
| ENSG00000264885.1 | 3.21E-56 | 4.7741272 | 6.2977 | -1.523573 |
| ENSG00000280927.1 | 6.72E-35 | 5.9003578 | 7.0300193 | -1.129662 |
| ENSG00000185186.8 | 7.49E-16 | 2.2569714 | 4.3077977 | -2.050826 |
| ENSG00000132677.12 | 2.65E-62 | 2.5007115 | 8.8603739 | -6.359662 |
| ENSG00000241720.2 | 1.16E-23 | 0.1689496 | 1.4031807 | -1.234231 |
| ENSG00000223749.7 | 2.55E-65 | 5.5076566 | 9.2294784 | -3.721822 |
| ENSG00000231728.3 | 1.32E-51 | 1.5305122 | 3.5176057 | -1.987094 |
| ENSG00000272622.1 | 6.41E-57 | 2.3018881 | 4.2980693 | -1.996181 |
| ENSG00000229186.4 | 4.68E-12 | 1.982199 | 4.020708 | -2.038509 |
| ENSG00000213071.10 | 5.04E-18 | 2.4006403 | 3.5539091 | -1.153269 |
| ENSG00000255072.1 | 1.12E-09 | 0.4007969 | 1.598775 | -1.197978 |
| ENSG00000135298.13 | 4.15E-61 | 2.5444563 | 5.678342 | -3.133886 |
| ENSG00000158747.13 | 1.64E-50 | 12.20664 | 13.864164 | -1.657524 |
| ENSG00000275542.1 | 5.33E-40 | 0.72431 | 2.6690284 | -1.944718 |
| ENSG00000247624.6 | 2.13E-18 | 0.7341136 | 1.7487841 | -1.01467 |
| ENSG00000237531.5 | 1.30E-38 | 0.4474943 | 2.582025 | -2.134531 |
| ENSG00000266010.1 | 1.04E-69 | 5.7173449 | 8.8326159 | -3.115271 |
| ENSG00000131386.17 | 1.63E-48 | 6.078399 | 9.9300659 | -3.851667 |
| ENSG00000117707.15 | 3.59E-80 | 4.9008196 | 8.8705386 | -3.969719 |
| ENSG00000268926.2 | 4.15E-27 | 0.3837986 | 1.94625 | -1.562451 |
| ENSG00000179094.13 | 1.19E-56 | 10.545898 | 13.487058 | -2.94116 |
| ENSG00000187922.13 | 4.05E-34 | 5.984752 | 8.4542477 | -2.469496 |
| ENSG00000268218.1 | 1.36E-91 | 4.8506909 | 7.2739148 | -2.423224 |
| ENSG00000223414.2 | 2.88E-56 | 1.9959215 | 9.5725261 | -7.576605 |
| ENSG00000281691.1 | 1.88E-88 | 3.9803874 | 6.4502398 | -2.469852 |
| ENSG00000258461.5 | 4.27E-118 | 6.264173 | 8.8236909 | -2.559518 |
| ENSG00000162595.4 | 1.32E-95 | 6.9199869 | 10.08565 | -3.165663 |
| ENSG00000276724.1 | 1.56E-98 | 3.4784391 | 6.2932727 | -2.814834 |
| ENSG00000279954.1 | 7.70E-29 | 1.9903413 | 3.090867 | -1.100526 |
| ENSG00000204071.9 | 4.26E-69 | 1.3290239 | 6.5322159 | -5.203192 |
| ENSG00000270071.1 | 1.74E-32 | 0.3312635 | 2.1863284 | -1.855065 |
| ENSG00000279619.1 | 2.48E-54 | 2.152759 | 4.1730545 | -2.020296 |
| ENSG00000259244.1 | 5.61E-117 | 3.5500644 | 6.8775534 | -3.327489 |
| ENSG00000271401.1 | 6.01E-65 | 0.734094 | 3.299042 | -2.564948 |
| ENSG00000250651.1 | 2.20E-43 | 0.9735315 | 3.0136852 | -2.040154 |
| ENSG00000108405.3 | 4.61E-11 | 4.7007007 | 6.2052977 | -1.504597 |
| ENSG00000168497.4 | 4.98E-88 | 7.5224403 | 10.279817 | -2.757377 |
| ENSG00000233806.7 | 8.96E-37 | 3.0820613 | 4.9358398 | -1.853778 |
| ENSG00000230500.1 | 1.03E-27 | 0.7346351 | 2.3034011 | -1.568766 |
| ENSG00000246922.8 | 1.01E-95 | 7.2851637 | 9.5610216 | -2.275858 |
| ENSG00000268038.1 | 4.34E-31 | 0.9540573 | 2.962358 | -2.008301 |
| ENSG00000250273.1 | 1.24E-17 | 1.6107558 | 3.1282284 | -1.517473 |
| ENSG00000072657.8 | 2.05E-81 | 4.381405 | 8.2968716 | -3.915467 |
| ENSG00000242288.9 | 1.12E-24 | 4.4517484 | 6.1662114 | -1.714463 |
| ENSG00000260279.2 | 1.20E-15 | 2.0416835 | 3.0873886 | -1.045705 |
| ENSG00000225032.5 | 1.19E-60 | 6.7921618 | 9.5920057 | -2.799844 |
| ENSG00000188641.12 | 6.57E-30 | 8.7556652 | 9.8300102 | -1.074345 |
| ENSG00000112214.10 | 9.78E-35 | 3.1846079 | 6.837642 | -3.653034 |
| ENSG00000261215.1 | 4.87E-53 | 3.8660613 | 5.435683 | -1.569622 |
| ENSG00000109339.18 | 2.74E-72 | 6.7560379 | 9.0384364 | -2.282398 |
| ENSG00000259134.5 | 1.06E-82 | 3.6122936 | 7.9378136 | -4.32552 |
| ENSG00000162526.6 | 1.11E-29 | 4.7968382 | 6.0023261 | -1.205488 |
| ENSG00000272168.5 | 1.02E-50 | 5.9893434 | 8.2392693 | -2.249926 |
| ENSG00000185304.13 | 1.73E-11 | 5.9032695 | 7.2622205 | -1.358951 |
| ENSG00000266964.5 | 1.19E-36 | 8.2507947 | 9.8248886 | -1.574094 |
| ENSG00000254852.8 | 1.78E-12 | 4.2075396 | 5.4188886 | -1.211349 |
| ENSG00000270025.2 | 5.08E-11 | 1.15479 | 2.7710102 | -1.61622 |
| ENSG00000236393.1 | 1.58E-27 | 3.0951687 | 5.9122307 | -2.817062 |
| ENSG00000076555.15 | 3.81E-64 | 9.4925258 | 10.824876 | -1.33235 |
| ENSG00000169946.13 | 2.25E-61 | 8.5890568 | 10.507011 | -1.917955 |
| ENSG00000278668.1 | 4.37E-37 | 1.6986594 | 3.3817102 | -1.683051 |
| ENSG00000203709.9 | 1.02E-39 | 7.5575033 | 9.4938011 | -1.936298 |
| ENSG00000281195.1 | 1.58E-53 | 2.483927 | 4.716883 | -2.232956 |
| ENSG00000270409.1 | 5.01E-36 | 1.1827315 | 3.177867 | -1.995136 |
| ENSG00000108342.12 | 3.16E-05 | 1.8008654 | 2.8251352 | -1.02427 |
| ENSG00000170890.13 | 1.15E-60 | 2.5610962 | 5.3457477 | -2.784652 |
| ENSG00000127528.5 | 1.60E-40 | 9.4958573 | 11.777086 | -2.281229 |
| ENSG00000113108.17 | 2.06E-58 | 9.6466391 | 11.120349 | -1.47371 |
| ENSG00000275322.1 | 1.82E-28 | 3.3093835 | 4.6975386 | -1.388155 |
| ENSG00000189221.9 | 4.16E-56 | 8.4567169 | 11.16277 | -2.706054 |
| ENSG00000258913.1 | 3.67E-43 | 1.3202169 | 3.6035102 | -2.283293 |
| ENSG00000122121.10 | 1.47E-16 | 3.4028659 | 5.708392 | -2.305526 |
| ENSG00000183801.7 | 2.42E-46 | 8.6230599 | 10.175428 | -1.552369 |
| ENSG00000279400.1 | 1.05E-14 | 4.0802351 | 5.5733352 | -1.4931 |
| ENSG00000114948.12 | 2.01E-37 | 5.6393434 | 7.2773955 | -1.638052 |
| ENSG00000214491.8 | 4.03E-16 | 5.2134952 | 6.4844341 | -1.270939 |
| ENSG00000236015.1 | 4.42E-24 | 4.3141821 | 5.5520034 | -1.237821 |
| ENSG00000145075.11 | 1.37E-116 | 5.4679516 | 8.7507034 | -3.282752 |
| ENSG00000226009.1 | 1.48E-25 | 2.3289375 | 3.3521568 | -1.023219 |
| ENSG00000244617.2 | 6.42E-53 | 5.7367131 | 7.2279648 | -1.491252 |
| ENSG00000146477.5 | 3.05E-135 | 4.4952327 | 10.059289 | -5.564056 |
| ENSG00000261379.1 | 7.05E-58 | 0.7421527 | 3.7755045 | -3.033352 |
| ENSG00000279530.1 | 6.25E-34 | 1.5457274 | 3.476283 | -1.930556 |
| ENSG00000280178.1 | 5.20E-16 | 0.0746988 | 2.0241182 | -1.949419 |
| ENSG00000280025.1 | 1.58E-21 | 0.3326993 | 1.5546659 | -1.221967 |
| ENSG00000121871.3 | 1.55E-09 | 2.0126654 | 3.5404773 | -1.527812 |
| ENSG00000282728.1 | 7.54E-65 | 1.730831 | 4.8518045 | -3.120974 |
| ENSG00000109072.13 | 2.61E-20 | 4.8161451 | 5.9262602 | -1.110115 |
| ENSG00000230018.1 | 1.47E-22 | 1.8394969 | 3.5851068 | -1.74561 |
| ENSG00000197442.9 | 1.59E-85 | 9.0511322 | 11.910289 | -2.859156 |
| ENSG00000257259.1 | 2.72E-15 | 0.6825291 | 2.7445977 | -2.062069 |
| ENSG00000255008.2 | 2.83E-26 | 0.2396095 | 1.7224159 | -1.482806 |
| ENSG00000231721.6 | 2.95E-27 | 8.3318103 | 9.8675091 | -1.535699 |
| ENSG00000135426.14 | 1.48E-20 | 5.0845558 | 6.5731591 | -1.488603 |
| ENSG00000048740.17 | 2.35E-56 | 9.5668814 | 11.33989 | -1.773008 |
| ENSG00000142156.14 | 1.84E-45 | 14.038821 | 15.536593 | -1.497772 |
| ENSG00000258636.1 | 5.32E-23 | 0.8372625 | 2.1784023 | -1.34114 |
| ENSG00000231121.3 | 4.56E-48 | 1.3461344 | 3.3807818 | -2.034647 |
| ENSG00000089847.12 | 6.26E-30 | 5.2369506 | 6.8000159 | -1.563065 |
| ENSG00000102383.13 | 4.76E-25 | 6.1151714 | 7.240442 | -1.125271 |
| ENSG00000243701.5 | 3.30E-31 | 8.0000819 | 9.2325875 | -1.232506 |
| ENSG00000135063.17 | 3.45E-79 | 7.2964411 | 10.474659 | -3.178218 |
| ENSG00000096088.16 | 1.05E-43 | 1.1097153 | 5.2523568 | -4.142642 |
| ENSG00000080503.19 | 7.62E-40 | 11.15599 | 12.231372 | -1.075382 |
| ENSG00000102385.12 | 9.80E-36 | 5.2820864 | 6.6824034 | -1.400317 |
| ENSG00000185559.13 | 8.33E-82 | 4.8925802 | 12.472128 | -7.579548 |
| ENSG00000271551.2 | 7.13E-31 | 4.0201086 | 5.6517716 | -1.631663 |
| ENSG00000187151.7 | 1.05E-82 | 1.1331663 | 7.735917 | -6.602751 |
| ENSG00000116983.12 | 1.40E-28 | 4.2180117 | 6.7727977 | -2.554786 |
| ENSG00000164530.13 | 5.04E-13 | 2.840858 | 4.8244727 | -1.983615 |
| ENSG00000107518.16 | 1.35E-62 | 5.3377766 | 9.15835 | -3.820573 |
| ENSG00000226167.1 | 6.36E-18 | 4.5740217 | 5.5942 | -1.020178 |
| ENSG00000282393.1 | 2.53E-62 | 6.2552561 | 7.5426716 | -1.287416 |
| ENSG00000229447.2 | 1.89E-25 | 2.0624601 | 3.1681227 | -1.105663 |
| ENSG00000127241.16 | 5.41E-28 | 4.5320635 | 6.8566784 | -2.324615 |
| ENSG00000185551.12 | 5.97E-74 | 11.841495 | 13.639535 | -1.798041 |
| ENSG00000081052.11 | 1.36E-35 | 6.104694 | 8.1346568 | -2.029963 |
| ENSG00000278330.1 | 8.89E-33 | 4.1058208 | 5.6080761 | -1.502255 |
| ENSG00000274492.1 | 2.56E-20 | 2.4101666 | 3.8679023 | -1.457736 |
| ENSG00000236882.7 | 1.24E-83 | 1.38262 | 4.8097773 | -3.427157 |
| ENSG00000253764.1 | 1.91E-18 | 1.1156313 | 2.3290466 | -1.213415 |
| ENSG00000187098.14 | 1.58E-60 | 8.2897554 | 9.6714193 | -1.381664 |
| ENSG00000278434.1 | 5.73E-29 | 2.332047 | 3.67695 | -1.344903 |
| ENSG00000263812.5 | 3.44E-49 | 5.1312415 | 7.1868375 | -2.055596 |
| ENSG00000169297.7 | 5.34E-47 | 3.1833496 | 6.2793932 | -3.096044 |
| ENSG00000270016.1 | 9.05E-33 | 1.6633501 | 3.1654432 | -1.502093 |
| ENSG00000255304.1 | 3.31E-15 | 0.0252189 | 1.0763307 | -1.051112 |
| ENSG00000258951.1 | 1.48E-22 | 1.7813091 | 3.0383375 | -1.257028 |
| ENSG00000269502.5 | 5.13E-33 | 1.5861317 | 4.074775 | -2.488643 |
| ENSG00000117477.12 | 1.26E-28 | 5.5770878 | 7.2711102 | -1.694022 |
| ENSG00000262304.1 | 5.84E-34 | 7.917389 | 9.2558057 | -1.338417 |
| ENSG00000259726.1 | 2.48E-86 | 6.0026575 | 8.7055295 | -2.702872 |
| ENSG00000251023.1 | 1.30E-23 | 4.1979587 | 5.4275023 | -1.229544 |
| ENSG00000267163.1 | 4.29E-72 | 2.9869897 | 5.1016977 | -2.114708 |
| ENSG00000227544.8 | 7.40E-23 | 2.5774403 | 3.820658 | -1.243218 |
| ENSG00000008311.14 | 6.87E-90 | 6.723858 | 11.315566 | -4.591708 |
| ENSG00000185070.10 | 7.87E-75 | 8.5798983 | 11.434377 | -2.854479 |
| ENSG00000143355.15 | 7.83E-78 | 4.4760551 | 8.2198466 | -3.743791 |
| ENSG00000146067.15 | 8.83E-42 | 10.527514 | 11.793486 | -1.265973 |
| ENSG00000069667.15 | 6.92E-52 | 8.5857494 | 10.198116 | -1.612367 |
| ENSG00000111886.10 | 1.13E-20 | 2.9389463 | 4.0146739 | -1.075728 |
| ENSG00000188130.13 | 1.40E-67 | 7.7008556 | 9.8166898 | -2.115834 |
| ENSG00000261292.2 | 1.69E-39 | 2.3442697 | 5.1748216 | -2.830552 |
| ENSG00000154330.12 | 3.39E-29 | 8.1044391 | 9.6781659 | -1.573727 |
| ENSG00000112562.18 | 2.40E-66 | 10.046287 | 12.31585 | -2.269563 |
| ENSG00000214922.9 | 2.97E-65 | 5.0705224 | 6.8067341 | -1.736212 |
| ENSG00000183770.5 | 2.17E-81 | 5.5899981 | 10.697632 | -5.107634 |
| ENSG00000171195.10 | 7.92E-07 | 0.415089 | 1.986942 | -1.571853 |
| ENSG00000184226.14 | 3.74E-14 | 5.1806718 | 6.2392102 | -1.058538 |
| ENSG00000227543.4 | 1.47E-36 | 5.137621 | 6.2791182 | -1.141497 |
| ENSG00000279926.1 | 1.97E-17 | 4.976478 | 6.0828795 | -1.106402 |
| ENSG00000279050.1 | 5.03E-20 | 1.5184057 | 2.9131 | -1.394694 |
| ENSG00000259205.2 | 1.05E-19 | 2.823215 | 4.2572682 | -1.434053 |
| ENSG00000152932.7 | 1.00E-18 | 2.2842578 | 4.9652432 | -2.680985 |
| ENSG00000259211.1 | 2.91E-45 | 1.1757847 | 3.3456523 | -2.169868 |
| ENSG00000274225.1 | 4.85E-34 | 2.9528504 | 4.4009864 | -1.448136 |
| ENSG00000198682.12 | 5.01E-51 | 8.884189 | 10.493475 | -1.609286 |
| ENSG00000150594.6 | 1.34E-21 | 8.6138718 | 9.9871739 | -1.373302 |
| ENSG00000259946.1 | 1.01E-13 | 0.0640112 | 1.1129591 | -1.048948 |
| ENSG00000260565.6 | 6.33E-37 | 8.5274747 | 9.7305966 | -1.203122 |
| ENSG00000111799.20 | 2.44E-17 | 12.423703 | 13.430152 | -1.006449 |
| ENSG00000188783.5 | 2.60E-59 | 10.994671 | 13.403376 | -2.408705 |
| ENSG00000260577.2 | 4.76E-62 | 5.7432387 | 8.9625943 | -3.219356 |
| ENSG00000181997.8 | 1.40E-09 | 0.3485986 | 1.401675 | -1.053076 |
| ENSG00000260296.1 | 6.99E-51 | 4.379969 | 6.4267409 | -2.046772 |
| ENSG00000260012.1 | 5.78E-20 | 0.8357162 | 1.9334648 | -1.097749 |
| ENSG00000213018.2 | 8.18E-24 | 1.6894222 | 2.8410216 | -1.151599 |
| ENSG00000258017.1 | 7.93E-20 | 5.0235757 | 7.5026284 | -2.479053 |
| ENSG00000270387.1 | 1.99E-27 | 0.194143 | 1.5480034 | -1.35386 |
| ENSG00000238358.2 | 2.83E-27 | 0.3298563 | 1.8060523 | -1.476196 |
| ENSG00000189398.5 | 2.10E-46 | 1.6596795 | 4.5082716 | -2.848592 |
| ENSG00000229036.7 | 6.86E-41 | 5.7676403 | 6.9406216 | -1.172981 |
| ENSG00000138379.4 | 4.05E-35 | 2.5085356 | 4.1312193 | -1.622684 |
| ENSG00000219642.2 | 4.02E-12 | 1.9337816 | 3.048592 | -1.11481 |
| ENSG00000276564.1 | 2.63E-28 | 4.3992909 | 5.5168614 | -1.11757 |
| ENSG00000197181.11 | 1.50E-82 | 4.0736155 | 6.7997091 | -2.726094 |
| ENSG00000056558.10 | 1.01E-50 | 8.5916372 | 10.070895 | -1.479258 |
| ENSG00000188803.14 | 2.89E-09 | 3.4491088 | 4.5374182 | -1.088309 |
| ENSG00000242068.1 | 9.56E-55 | 0.5353432 | 4.0160534 | -3.48071 |
| ENSG00000211643.2 | 1.20E-17 | 2.0096482 | 3.3305398 | -1.320892 |
| ENSG00000196295.11 | 2.02E-38 | 9.1102198 | 10.134331 | -1.024111 |
| ENSG00000162438.11 | 3.84E-22 | 1.6283351 | 3.5178807 | -1.889546 |
| ENSG00000133878.8 | 9.44E-96 | 3.5819186 | 8.7045727 | -5.122654 |
| ENSG00000251634.2 | 1.01E-26 | 4.9858967 | 6.6701023 | -1.684206 |
| ENSG00000092421.16 | 6.25E-14 | 8.7249964 | 9.9631045 | -1.238108 |
| ENSG00000213213.13 | 2.24E-31 | 5.5724177 | 7.5888477 | -2.01643 |
| ENSG00000248703.2 | 1.18E-32 | 1.7053117 | 3.8582295 | -2.152918 |
| ENSG00000168490.13 | 3.07E-36 | 4.8817122 | 7.8131182 | -2.931406 |
| ENSG00000273476.1 | 1.78E-14 | 0.6066057 | 1.6361909 | -1.029585 |
| ENSG00000243978.8 | 9.79E-39 | 3.0854964 | 5.6434148 | -2.557918 |
| ENSG00000167995.15 | 4.38E-29 | 7.4172444 | 8.6235125 | -1.206268 |
| ENSG00000263321.1 | 3.36E-25 | 1.426178 | 2.7928557 | -1.366678 |
| ENSG00000280312.1 | 9.14E-20 | 0.1930196 | 1.3764 | -1.18338 |
| ENSG00000172640.3 | 7.09E-18 | 1.8348551 | 2.8731602 | -1.038305 |
| ENSG00000146966.12 | 9.83E-125 | 7.34738 | 10.153665 | -2.806285 |
| ENSG00000137731.13 | 5.83E-50 | 4.5410551 | 6.9331614 | -2.392106 |
| ENSG00000278740.1 | 5.23E-23 | 2.1863294 | 3.5088295 | -1.3225 |
| ENSG00000173421.16 | 6.16E-63 | 4.2880368 | 7.0379193 | -2.749883 |
| ENSG00000229867.1 | 1.04E-31 | 2.5143518 | 3.6255739 | -1.111222 |
| ENSG00000109046.14 | 3.57E-60 | 11.380901 | 13.2102 | -1.829299 |
| ENSG00000171116.7 | 1.53E-37 | 5.0121272 | 6.3756398 | -1.363513 |
| ENSG00000239704.10 | 2.59E-20 | 6.9256706 | 8.6389898 | -1.713319 |
| ENSG00000261063.1 | 1.54E-13 | 0.1550143 | 1.624467 | -1.469453 |
| ENSG00000155511.17 | 1.61E-30 | 1.4136406 | 3.4968705 | -2.08323 |
| ENSG00000260859.1 | 7.57E-18 | 0.1633666 | 1.3627864 | -1.19942 |
| ENSG00000274565.1 | 5.10E-96 | 4.8106647 | 9.0138602 | -4.203196 |
| ENSG00000235100.3 | 9.87E-16 | 1.4924687 | 2.5064625 | -1.013994 |
| ENSG00000179938.12 | 1.54E-14 | 1.5610976 | 2.7223489 | -1.161251 |
| ENSG00000213435.3 | 1.56E-38 | 0.873285 | 2.5435716 | -1.670287 |
| ENSG00000198848.12 | 7.08E-38 | 6.1212229 | 9.0024625 | -2.88124 |
| ENSG00000165837.11 | 1.22E-54 | 1.9320711 | 3.8752591 | -1.943188 |
| ENSG00000225399.4 | 4.88E-54 | 2.944953 | 5.3884182 | -2.443465 |
| ENSG00000082175.14 | 4.42E-81 | 7.398595 | 10.804544 | -3.405949 |
| ENSG00000140285.9 | 6.72E-58 | 6.6596205 | 9.7917273 | -3.132107 |
| ENSG00000130988.12 | 7.28E-34 | 7.0831582 | 8.923842 | -1.840684 |
| ENSG00000256250.1 | 4.43E-13 | 0.6963711 | 1.7056614 | -1.00929 |
| ENSG00000091704.9 | 1.35E-16 | 3.8541131 | 5.9059591 | -2.051846 |
| ENSG00000271959.1 | 4.85E-21 | 4.5472773 | 6.7823795 | -2.235102 |
| ENSG00000271265.1 | 1.06E-34 | 1.5410394 | 3.1392023 | -1.598163 |
| ENSG00000260563.3 | 9.92E-66 | 6.2825833 | 8.0069148 | -1.724331 |
| ENSG00000158805.11 | 2.56E-62 | 9.6869685 | 10.946389 | -1.25942 |
| ENSG00000188848.15 | 6.87E-11 | 2.7124305 | 3.9222966 | -1.209866 |
| ENSG00000186908.14 | 6.75E-69 | 9.7022005 | 10.768581 | -1.06638 |
| ENSG00000005187.11 | 2.46E-68 | 9.9128272 | 12.738293 | -2.825466 |
| ENSG00000154262.12 | 4.71E-113 | 5.5938809 | 10.924651 | -5.33077 |
| ENSG00000254254.5 | 7.79E-21 | 0.2638628 | 1.8743795 | -1.610517 |
| ENSG00000110786.17 | 1.92E-39 | 3.855163 | 6.176375 | -2.321212 |
| ENSG00000091137.11 | 1.02E-26 | 3.4623597 | 4.7852523 | -1.322893 |
| ENSG00000259370.2 | 6.02E-20 | 2.9504678 | 4.29715 | -1.346682 |
| ENSG00000085563.14 | 8.46E-34 | 5.8860916 | 7.2905307 | -1.404439 |
| ENSG00000165495.15 | 6.93E-22 | 8.3128427 | 9.3272398 | -1.014397 |
| ENSG00000241135.5 | 3.33E-17 | 1.0989737 | 2.6597182 | -1.560744 |
| ENSG00000180139.11 | 4.33E-23 | 6.1518919 | 8.1038477 | -1.951956 |
| ENSG00000240303.7 | 4.43E-62 | 9.4036771 | 11.120244 | -1.716567 |
| ENSG00000261326.2 | 2.97E-33 | 8.2390351 | 9.548583 | -1.309548 |
| ENSG00000244560.6 | 3.07E-94 | 7.4076914 | 9.5876352 | -2.179944 |
| ENSG00000149150.8 | 1.67E-74 | 7.8636604 | 9.6768068 | -1.813146 |
| ENSG00000247809.7 | 1.23E-159 | 4.0139351 | 8.3297955 | -4.31586 |
| ENSG00000241644.2 | 7.24E-20 | 6.2521074 | 8.6077705 | -2.355663 |
| ENSG00000185031.6 | 4.48E-34 | 2.0426029 | 4.0470886 | -2.004486 |
| ENSG00000138080.13 | 4.36E-60 | 11.003966 | 13.052959 | -2.048993 |
| ENSG00000273343.1 | 1.21E-32 | 2.6476718 | 3.9700489 | -1.322377 |
| ENSG00000234377.7 | 4.18E-22 | 1.672753 | 3.1144784 | -1.441725 |
| ENSG00000089060.11 | 9.97E-40 | 10.717261 | 11.901535 | -1.184275 |
| ENSG00000260837.1 | 4.13E-36 | 4.6407232 | 5.8647068 | -1.223984 |
| ENSG00000246022.2 | 6.72E-60 | 1.4754628 | 6.4528966 | -4.977434 |
| ENSG00000013293.5 | 4.89E-84 | 1.2463764 | 4.4594318 | -3.213055 |
| ENSG00000111450.13 | 1.53E-46 | 8.5546685 | 9.5984125 | -1.043744 |
| ENSG00000225449.3 | 7.82E-14 | 1.477453 | 2.7656886 | -1.288236 |
| ENSG00000254362.1 | 1.83E-25 | 0.2843365 | 1.8815273 | -1.597191 |
| ENSG00000188257.10 | 1.53E-40 | 5.6864754 | 11.209461 | -5.522986 |
| ENSG00000258604.1 | 2.53E-30 | 1.1975434 | 2.6997807 | -1.502237 |
| ENSG00000149403.11 | 1.34E-14 | 5.8145683 | 7.0896352 | -1.275067 |
| ENSG00000151303.11 | 7.58E-75 | 3.5991893 | 6.3362205 | -2.737031 |
| ENSG00000180422.3 | 4.98E-117 | 1.0035329 | 5.6997864 | -4.696253 |
| ENSG00000163092.19 | 5.52E-26 | 0.7793558 | 3.1255045 | -2.346149 |
| ENSG00000261821.2 | 1.48E-74 | 2.9084363 | 8.042842 | -5.134406 |
| ENSG00000214988.4 | 1.19E-23 | 0.637636 | 2.0589455 | -1.421309 |
| ENSG00000236173.1 | 1.55E-34 | 0.3580172 | 2.4718636 | -2.113846 |
| ENSG00000197321.14 | 1.43E-57 | 11.440842 | 12.667388 | -1.226545 |
| ENSG00000229127.1 | 3.35E-43 | 4.5505666 | 5.873125 | -1.322558 |
| ENSG00000185873.7 | 2.78E-08 | 0.6311403 | 1.7195364 | -1.088396 |
| ENSG00000240929.2 | 1.56E-36 | 3.5407947 | 5.7212523 | -2.180458 |
| ENSG00000279271.1 | 2.32E-37 | 0.4224377 | 4.0160989 | -3.593661 |
| ENSG00000271992.1 | 5.38E-20 | 2.3879671 | 3.7936807 | -1.405714 |
| ENSG00000124568.10 | 9.95E-16 | 0.1656578 | 1.6935989 | -1.527941 |
| ENSG00000188038.7 | 6.77E-39 | 3.3347014 | 5.0747807 | -1.740079 |
| ENSG00000226891.6 | 5.25E-64 | 3.8503995 | 6.2503466 | -2.399947 |
| ENSG00000110888.17 | 2.42E-57 | 9.7486399 | 10.932715 | -1.184075 |
| ENSG00000277767.1 | 8.70E-31 | 3.6844842 | 5.2010227 | -1.516538 |
| ENSG00000261621.1 | 1.53E-16 | 0.1383007 | 1.1875784 | -1.049278 |
| ENSG00000118596.11 | 6.13E-46 | 6.2985604 | 7.7994295 | -1.500869 |
| ENSG00000104381.12 | 2.45E-27 | 7.4660019 | 8.5098045 | -1.043803 |
| ENSG00000272281.6 | 3.57E-21 | 1.9173897 | 3.3371023 | -1.419713 |
| ENSG00000175066.15 | 5.28E-28 | 10.013315 | 11.036167 | -1.022852 |
| ENSG00000139220.16 | 2.12E-37 | 2.6742196 | 5.2739159 | -2.599696 |
| ENSG00000244124.1 | 7.20E-13 | 1.1493368 | 3.060067 | -1.91073 |
| ENSG00000104938.16 | 1.34E-43 | 2.632805 | 6.2302193 | -3.597414 |
| ENSG00000269934.1 | 1.70E-31 | 3.1450704 | 4.3256773 | -1.180607 |
| ENSG00000261011.1 | 3.83E-47 | 4.4074444 | 6.8779295 | -2.470485 |
| ENSG00000105928.13 | 6.88E-42 | 8.1709723 | 9.3022148 | -1.131242 |
| ENSG00000147145.12 | 1.23E-31 | 4.1131947 | 5.9787318 | -1.865537 |
| ENSG00000126368.5 | 1.25E-13 | 9.9106969 | 11.155457 | -1.24476 |
| ENSG00000077157.20 | 4.06E-61 | 10.034657 | 12.067294 | -2.032638 |
| ENSG00000113721.13 | 7.48E-87 | 11.196449 | 13.287155 | -2.090706 |
| ENSG00000261270.1 | 2.18E-23 | 2.7774086 | 4.4478852 | -1.670477 |
| ENSG00000230257.1 | 6.95E-15 | 0.7014728 | 1.7413216 | -1.039849 |
| ENSG00000272084.1 | 1.16E-26 | 4.7897344 | 5.8328568 | -1.043122 |
| ENSG00000138039.14 | 6.35E-40 | 2.0582036 | 5.9459841 | -3.887781 |
| ENSG00000241860.6 | 1.02E-35 | 6.574268 | 7.9869318 | -1.412664 |
| ENSG00000089057.14 | 2.22E-39 | 10.81244 | 12.00202 | -1.18958 |
| ENSG00000212124.2 | 5.60E-40 | 2.0717286 | 3.9953693 | -1.923641 |
| ENSG00000226622.5 | 9.94E-59 | 0.4046735 | 5.0274625 | -4.622789 |
| ENSG00000143839.13 | 2.08E-21 | 4.3914408 | 7.2968295 | -2.905389 |
| ENSG00000145020.14 | 7.65E-76 | 8.4453444 | 10.699524 | -2.254179 |
| ENSG00000175164.13 | 2.94E-33 | 9.1705029 | 10.959858 | -1.789355 |
| ENSG00000243478.7 | 8.77E-24 | 0.1682062 | 2.0569818 | -1.888776 |
| ENSG00000226443.3 | 1.42E-17 | 1.0739181 | 2.2327307 | -1.158813 |
| ENSG00000258168.5 | 2.63E-28 | 3.6411196 | 5.4393932 | -1.798274 |
| ENSG00000184716.13 | 4.65E-49 | 1.8522671 | 3.841142 | -1.988875 |
| ENSG00000099840.13 | 1.73E-66 | 5.8082797 | 9.123683 | -3.315403 |
| ENSG00000272374.1 | 3.76E-62 | 5.431589 | 7.6576443 | -2.226055 |
| ENSG00000229688.7 | 3.01E-17 | 0.7960389 | 1.9212068 | -1.125168 |
| ENSG00000271141.1 | 1.51E-37 | 2.6835835 | 4.1290432 | -1.44546 |
| ENSG00000253304.1 | 2.98E-124 | 7.368721 | 10.268358 | -2.899637 |
| ENSG00000225756.1 | 3.99E-60 | 4.1269995 | 6.3840352 | -2.257036 |
| ENSG00000164318.17 | 6.52E-19 | 8.8125582 | 9.827025 | -1.014467 |
| ENSG00000070808.15 | 2.93E-29 | 2.5963721 | 4.5291807 | -1.932809 |
| ENSG00000233705.6 | 3.32E-16 | 2.1606282 | 3.6697545 | -1.509126 |
| ENSG00000140009.18 | 2.84E-66 | 5.2641169 | 8.230067 | -2.96595 |
| ENSG00000244161.1 | 1.53E-52 | 4.9710924 | 6.6933193 | -1.722227 |
| ENSG00000188993.3 | 4.02E-25 | 3.3873554 | 4.5980557 | -1.2107 |
| ENSG00000013297.10 | 2.18E-72 | 8.8432274 | 11.76345 | -2.920223 |
| ENSG00000223006.1 | 5.17E-17 | 0.0218513 | 1.0538636 | -1.032012 |
| ENSG00000169031.18 | 1.80E-69 | 4.5984418 | 8.5222102 | -3.923768 |
| ENSG00000132855.4 | 1.80E-27 | 1.672916 | 3.0106932 | -1.337777 |
| ENSG00000245711.2 | 7.38E-41 | 1.3498169 | 2.9174443 | -1.567627 |
| ENSG00000130176.7 | 1.56E-13 | 8.4964203 | 10.043678 | -1.547258 |
| ENSG00000236849.5 | 5.91E-39 | 0.5549434 | 2.1747932 | -1.61985 |
| ENSG00000275993.2 | 1.32E-11 | 10.119277 | 11.728998 | -1.609721 |
| ENSG00000170962.12 | 3.03E-70 | 8.8699084 | 11.229882 | -2.359973 |
| ENSG00000205959.3 | 1.49E-95 | 4.1812179 | 7.7860341 | -3.604816 |
| ENSG00000168386.18 | 8.50E-20 | 10.118714 | 11.214991 | -1.096277 |
| ENSG00000090539.15 | 4.83E-141 | 6.97302 | 10.544824 | -3.571804 |
| ENSG00000271547.1 | 6.16E-45 | 1.6123303 | 3.6626648 | -2.050334 |
| ENSG00000154734.14 | 7.36E-53 | 11.929855 | 14.875316 | -2.945461 |
| ENSG00000111664.10 | 2.83E-79 | 5.6663031 | 8.6506568 | -2.984354 |
| ENSG00000259562.2 | 3.94E-40 | 3.1738215 | 4.4663966 | -1.292575 |
| ENSG00000260693.1 | 5.21E-45 | 4.0396563 | 5.4849545 | -1.445298 |
| ENSG00000066735.14 | 3.60E-36 | 6.4828351 | 8.5304864 | -2.047651 |
| ENSG00000267022.1 | 5.98E-25 | 1.7697895 | 4.3478977 | -2.578108 |
| ENSG00000215478.8 | 1.19E-37 | 2.6740198 | 6.2892455 | -3.615226 |
| ENSG00000230415.1 | 2.62E-39 | 3.5091253 | 4.9657205 | -1.456595 |
| ENSG00000203782.5 | 3.43E-32 | 0.7786277 | 2.8860625 | -2.107435 |
| ENSG00000264187.1 | 2.96E-40 | 1.321248 | 4.2889807 | -2.967733 |
| ENSG00000244301.5 | 1.67E-57 | 0.4128191 | 3.5209386 | -3.10812 |
| ENSG00000071575.11 | 5.12E-24 | 11.113969 | 12.224686 | -1.110717 |
| ENSG00000275672.1 | 1.35E-31 | 0.6367747 | 2.4636 | -1.826825 |
| ENSG00000118432.12 | 2.58E-28 | 4.7591874 | 7.2799534 | -2.520766 |
| ENSG00000180875.4 | 4.68E-44 | 4.0691334 | 7.5931602 | -3.524027 |
| ENSG00000224057.1 | 1.57E-33 | 1.1123432 | 3.2102091 | -2.097866 |
| ENSG00000276298.1 | 6.25E-29 | 0.2378897 | 1.662633 | -1.424743 |
| ENSG00000150048.10 | 6.23E-24 | 5.9629845 | 7.0894739 | -1.126489 |
| ENSG00000020181.17 | 2.73E-57 | 10.163703 | 11.767419 | -1.603716 |
| ENSG00000151789.9 | 6.78E-15 | 4.1475666 | 5.3068614 | -1.159295 |
| ENSG00000272021.1 | 2.19E-17 | 0.5644146 | 1.6514057 | -1.086991 |
| ENSG00000249926.2 | 9.42E-20 | 1.2941289 | 2.7235193 | -1.42939 |
| ENSG00000123360.11 | 4.77E-64 | 7.8067387 | 9.8580614 | -2.051323 |
| ENSG00000279807.1 | 6.43E-28 | 1.2930695 | 2.4721011 | -1.179032 |
| ENSG00000279245.1 | 7.53E-41 | 1.8231916 | 2.953825 | -1.130633 |
| ENSG00000261247.1 | 5.86E-67 | 1.2252007 | 4.2663773 | -3.041177 |
| ENSG00000237499.6 | 3.20E-30 | 5.11341 | 6.5663432 | -1.452933 |
| ENSG00000276550.4 | 2.99E-37 | 10.376731 | 12.11422 | -1.737489 |
| ENSG00000272887.1 | 1.32E-127 | 2.2582506 | 6.8989795 | -4.640729 |
| ENSG00000236194.3 | 6.21E-46 | 1.1554578 | 3.309883 | -2.154425 |
| ENSG00000277737.2 | 7.08E-22 | 0.8817496 | 3.0879773 | -2.206228 |
| ENSG00000116783.14 | 8.32E-23 | 3.2238 | 5.0657773 | -1.841977 |
| ENSG00000145536.15 | 7.71E-32 | 6.0137821 | 9.0443136 | -3.030532 |
| ENSG00000154258.16 | 6.08E-181 | 5.5928146 | 11.29951 | -5.706696 |
| ENSG00000259683.1 | 4.82E-111 | 2.0308093 | 5.7579352 | -3.727126 |
| ENSG00000158458.19 | 2.43E-47 | 6.4802408 | 8.2967477 | -1.816507 |
| ENSG00000263272.1 | 6.10E-49 | 7.0901091 | 8.6749443 | -1.584835 |
| ENSG00000165966.14 | 8.66E-34 | 4.4209284 | 7.0046307 | -2.583702 |
| ENSG00000238039.1 | 1.01E-23 | 4.0103702 | 5.567517 | -1.557147 |
| ENSG00000021826.14 | 1.84E-33 | 6.2251019 | 7.665767 | -1.440665 |
| ENSG00000263164.1 | 1.11E-53 | 3.2596604 | 5.0613989 | -1.801738 |
| ENSG00000164303.10 | 4.44E-58 | 4.0993033 | 8.002308 | -3.903005 |
| ENSG00000260618.1 | 2.27E-47 | 2.8592339 | 4.4973364 | -1.638102 |
| ENSG00000169605.5 | 3.81E-20 | 0.1128773 | 1.9119591 | -1.799082 |
| ENSG00000231459.1 | 4.44E-27 | 1.3536878 | 2.8365477 | -1.48286 |
| ENSG00000080166.15 | 1.46E-55 | 3.00358 | 5.3095182 | -2.305938 |
| ENSG00000111962.7 | 3.75E-46 | 8.0633086 | 9.2132966 | -1.149988 |
| ENSG00000163631.16 | 7.36E-23 | 3.6539482 | 5.5105136 | -1.856565 |
| ENSG00000219361.1 | 6.22E-33 | 0.3263131 | 1.6558989 | -1.329586 |
| ENSG00000168621.14 | 5.72E-14 | 5.1101916 | 6.422642 | -1.31245 |
| ENSG00000261646.1 | 2.47E-41 | 0.5217 | 2.64685 | -2.12515 |
| ENSG00000153707.15 | 4.47E-79 | 6.9211119 | 10.569483 | -3.648371 |
| ENSG00000101470.9 | 5.05E-43 | 4.1088346 | 5.9116716 | -1.802837 |
| ENSG00000170356.8 | 6.37E-11 | 6.7771931 | 8.0401761 | -1.262983 |
| ENSG00000178803.10 | 2.77E-53 | 2.2872962 | 4.0177034 | -1.730407 |
| ENSG00000168530.15 | 1.29E-17 | 0.2156706 | 2.439033 | -2.223362 |
| ENSG00000163492.13 | 1.12E-22 | 2.7694064 | 4.3092398 | -1.539833 |
| ENSG00000227060.6 | 1.09E-36 | 1.7265298 | 3.517033 | -1.790503 |
| ENSG00000242282.6 | 3.42E-27 | 5.7987181 | 6.8062591 | -1.007541 |
| ENSG00000119946.10 | 1.44E-80 | 4.3515437 | 7.429042 | -3.077498 |
| ENSG00000273598.1 | 1.85E-11 | 1.0018427 | 2.3773977 | -1.375555 |
| ENSG00000256812.1 | 1.22E-12 | 0.8121759 | 1.8297159 | -1.01754 |
| ENSG00000115896.15 | 9.76E-36 | 7.0166752 | 8.5586409 | -1.541966 |
| ENSG00000237125.8 | 1.02E-105 | 4.1952582 | 10.045426 | -5.850168 |
| ENSG00000119865.8 | 7.72E-130 | 7.8322179 | 10.156664 | -2.324446 |
| ENSG00000162944.10 | 1.71E-36 | 7.0367957 | 8.3643398 | -1.327544 |
| ENSG00000267515.1 | 3.02E-19 | 0.7803687 | 1.9181273 | -1.137759 |
| ENSG00000135502.16 | 4.24E-80 | 5.3543878 | 8.2964091 | -2.942021 |
| ENSG00000259381.2 | 8.35E-29 | 1.7536742 | 3.2122341 | -1.45856 |
| ENSG00000142611.16 | 3.95E-43 | 4.9899434 | 7.5389261 | -2.548983 |
| ENSG00000278029.1 | 6.39E-41 | 1.105506 | 3.1742409 | -2.068735 |
| ENSG00000276107.1 | 1.35E-34 | 2.9796573 | 5.5913091 | -2.611652 |
| ENSG00000281538.1 | 9.27E-47 | 3.0732224 | 5.2631659 | -2.189943 |
| ENSG00000243335.8 | 7.23E-64 | 9.0094253 | 10.448948 | -1.439522 |
| ENSG00000269227.1 | 1.15E-41 | 2.1011379 | 3.7221591 | -1.621021 |
| ENSG00000188760.10 | 4.26E-17 | 7.5143678 | 8.6756011 | -1.161233 |
| ENSG00000272848.1 | 9.56E-45 | 3.2177174 | 4.8273352 | -1.609618 |
| ENSG00000141338.13 | 2.72E-209 | 6.0494902 | 12.195372 | -6.145881 |
| ENSG00000279232.1 | 3.26E-07 | 4.6479955 | 5.7241045 | -1.076109 |
| ENSG00000270108.1 | 2.07E-63 | 2.8845277 | 4.5022875 | -1.61776 |
| ENSG00000261656.5 | 3.34E-22 | 2.0665902 | 3.3046943 | -1.238104 |
| ENSG00000117525.13 | 4.11E-33 | 8.8445737 | 11.780181 | -2.935607 |
| ENSG00000164949.7 | 1.29E-11 | 7.4593251 | 8.7576216 | -1.298297 |
| ENSG00000267283.1 | 4.09E-17 | 1.9897014 | 3.44055 | -1.450849 |
| ENSG00000273188.1 | 5.41E-48 | 3.1548597 | 4.9274716 | -1.772612 |
| ENSG00000273923.1 | 5.52E-86 | 0.6539348 | 4.1222955 | -3.468361 |
| ENSG00000140090.17 | 8.15E-27 | 3.2640912 | 4.9220375 | -1.657946 |
| ENSG00000260517.2 | 7.57E-39 | 3.9507735 | 5.3907375 | -1.439964 |
| ENSG00000162873.14 | 1.74E-62 | 8.859442 | 13.28833 | -4.428888 |
| ENSG00000005379.15 | 2.26E-44 | 10.161294 | 11.539814 | -1.37852 |
| ENSG00000260139.6 | 8.44E-10 | 3.0887198 | 4.26365 | -1.17493 |
| ENSG00000270641.1 | 1.47E-27 | 4.0477671 | 5.3609795 | -1.313212 |
| ENSG00000164440.14 | 5.52E-29 | 6.0673945 | 8.3495443 | -2.28215 |
| ENSG00000234927.1 | 1.72E-30 | 0.3697332 | 2.7774705 | -2.407737 |
| ENSG00000264673.1 | 4.60E-21 | 1.1313909 | 2.4887636 | -1.357373 |
| ENSG00000231628.1 | 1.52E-51 | 2.9973048 | 4.4952659 | -1.497961 |
| ENSG00000269930.1 | 2.56E-21 | 5.398969 | 6.7367011 | -1.337732 |
| ENSG00000198947.14 | 1.64E-38 | 8.6148475 | 9.9361295 | -1.321282 |
| ENSG00000277595.1 | 1.34E-16 | 1.798448 | 2.913083 | -1.114635 |
| ENSG00000254832.1 | 2.24E-25 | 0.0347229 | 2.1222261 | -2.087503 |
| ENSG00000254636.1 | 7.33E-30 | 0.7154411 | 2.5159864 | -1.800545 |
| ENSG00000233984.1 | 3.45E-23 | 0.9828637 | 2.1492023 | -1.166339 |
| ENSG00000181804.14 | 5.27E-30 | 7.5355859 | 8.5405182 | -1.004932 |
| ENSG00000070404.9 | 2.44E-26 | 9.4131499 | 10.87341 | -1.46026 |
| ENSG00000260510.1 | 4.89E-21 | 0.3345914 | 1.4456545 | -1.111063 |
| ENSG00000277959.1 | 1.35E-17 | 3.2076021 | 4.4289977 | -1.221396 |
| ENSG00000259969.1 | 1.23E-57 | 4.3646427 | 6.3882352 | -2.023593 |
| ENSG00000267096.1 | 4.62E-18 | 2.0475453 | 3.1170614 | -1.069516 |
| ENSG00000188933.14 | 1.43E-13 | 5.9919914 | 8.5591761 | -2.567185 |
| ENSG00000226179.5 | 1.71E-19 | 3.2903363 | 4.3715773 | -1.081241 |
| ENSG00000272167.2 | 1.46E-22 | 0.1075535 | 1.6315011 | -1.523948 |
| ENSG00000230373.8 | 1.63E-137 | 6.7278854 | 10.682444 | -3.954559 |
| ENSG00000163239.12 | 1.79E-141 | 4.1158155 | 8.3752182 | -4.259403 |
| ENSG00000130032.15 | 3.19E-06 | 5.5448303 | 6.5827057 | -1.037875 |
| ENSG00000230371.1 | 1.56E-48 | 0.7674501 | 2.770808 | -2.003358 |
| ENSG00000203761.5 | 1.08E-55 | 6.066189 | 7.4941227 | -1.427934 |
| ENSG00000277152.1 | 1.04E-52 | 2.5776537 | 4.3670125 | -1.789359 |
| ENSG00000277007.1 | 5.43E-83 | 2.7173776 | 5.6891432 | -2.971766 |
| ENSG00000243708.8 | 1.16E-60 | 7.5494079 | 9.9952239 | -2.445816 |
| ENSG00000280291.1 | 1.72E-38 | 0.3177745 | 2.932325 | -2.614551 |
| ENSG00000272789.1 | 5.08E-17 | 2.1339048 | 4.0042136 | -1.870309 |
| ENSG00000160882.11 | 8.21E-27 | 0.1074797 | 3.2567818 | -3.149302 |
| ENSG00000275149.1 | 5.73E-27 | 3.5265315 | 4.7590727 | -1.232541 |
| ENSG00000135409.10 | 4.25E-86 | 3.9645002 | 9.3343932 | -5.369893 |
| ENSG00000157514.16 | 2.29E-26 | 11.387721 | 13.051528 | -1.663807 |
| ENSG00000173757.9 | 9.54E-111 | 10.694159 | 12.255293 | -1.561134 |
| ENSG00000254245.2 | 5.21E-75 | 5.2813549 | 7.5752 | -2.293845 |
| ENSG00000067798.13 | 3.62E-97 | 6.7210632 | 9.9369943 | -3.215931 |
| ENSG00000250056.5 | 2.56E-50 | 5.0956776 | 7.9734102 | -2.877733 |
| ENSG00000273259.2 | 6.40E-27 | 8.8858945 | 11.276816 | -2.390921 |
| ENSG00000196302.5 | 7.16E-10 | 2.3234527 | 3.6185852 | -1.295132 |
| ENSG00000178573.6 | 3.97E-64 | 10.148582 | 11.889178 | -1.740596 |
| ENSG00000153086.13 | 1.29E-17 | 2.0272979 | 3.0351568 | -1.007859 |
| ENSG00000147894.14 | 3.37E-22 | 9.3818258 | 10.566368 | -1.184542 |
| ENSG00000171357.5 | 5.58E-61 | 5.1420291 | 6.755283 | -1.613254 |
| ENSG00000242267.6 | 3.12E-60 | 1.2463384 | 4.2104148 | -2.964076 |
| ENSG00000180287.16 | 1.28E-64 | 1.2724308 | 5.8803239 | -4.607893 |
| ENSG00000131016.16 | 8.54E-45 | 10.938762 | 13.186206 | -2.247444 |
| ENSG00000279903.1 | 3.06E-44 | 0.5279735 | 4.0811727 | -3.553199 |
| ENSG00000156011.16 | 3.25E-73 | 8.9174967 | 11.196297 | -2.2788 |
| ENSG00000230779.1 | 3.12E-15 | 0.3357055 | 1.5620091 | -1.226304 |
| ENSG00000167615.16 | 2.68E-90 | 12.771744 | 14.608286 | -1.836543 |
| ENSG00000123570.3 | 7.47E-86 | 5.425315 | 8.425817 | -3.000502 |
| ENSG00000248469.1 | 1.40E-39 | 1.3613129 | 3.863492 | -2.502179 |
| ENSG00000205037.2 | 1.49E-12 | 2.9669759 | 4.371192 | -1.404216 |
| ENSG00000133107.14 | 5.37E-79 | 3.4899377 | 6.7739523 | -3.284015 |
| ENSG00000138356.13 | 2.45E-126 | 7.2727943 | 11.488088 | -4.215293 |
| ENSG00000118729.11 | 4.07E-14 | 4.5493487 | 6.4091 | -1.859751 |
| ENSG00000272508.1 | 1.73E-39 | 1.7007217 | 4.0560364 | -2.355315 |
| ENSG00000109771.15 | 3.63E-28 | 6.9187766 | 8.3557909 | -1.437014 |
| ENSG00000213199.7 | 6.40E-44 | 6.2603504 | 7.8342852 | -1.573935 |
| ENSG00000161634.11 | 9.46E-19 | 0.1516893 | 1.7847091 | -1.63302 |
| ENSG00000164330.16 | 1.62E-33 | 7.6639668 | 9.2578114 | -1.593845 |
| ENSG00000069702.10 | 2.54E-73 | 10.296621 | 12.759849 | -2.463227 |
| ENSG00000250397.2 | 1.46E-53 | 4.6011952 | 6.4708773 | -1.869682 |
| ENSG00000253485.2 | 1.93E-55 | 5.5798377 | 7.2331011 | -1.653263 |
| ENSG00000243230.1 | 1.07E-33 | 2.7195475 | 4.0573068 | -1.337759 |
| ENSG00000241158.5 | 2.28E-108 | 5.0480131 | 9.5709636 | -4.522951 |
| ENSG00000255201.1 | 2.48E-51 | 3.478827 | 5.5816693 | -2.102842 |
| ENSG00000116132.11 | 3.54E-22 | 9.174822 | 10.445055 | -1.270233 |
| ENSG00000179304.16 | 6.90E-40 | 8.7485616 | 10.100368 | -1.351807 |
| ENSG00000176868.2 | 2.65E-73 | 3.1177931 | 9.7562852 | -6.638492 |
| ENSG00000269332.5 | 3.34E-26 | 0.3074263 | 2.4953784 | -2.187952 |
| ENSG00000270055.1 | 9.81E-49 | 7.5122274 | 9.3232705 | -1.811043 |
| ENSG00000272864.1 | 5.36E-18 | 3.2419169 | 4.4186023 | -1.176685 |
| ENSG00000254109.5 | 3.29E-25 | 4.3162673 | 5.8716545 | -1.555387 |
| ENSG00000119440.8 | 1.04E-27 | 0.7176816 | 2.3723148 | -1.654633 |
| ENSG00000132938.18 | 8.97E-40 | 4.6524229 | 7.080125 | -2.427702 |
| ENSG00000279041.1 | 2.43E-130 | 3.7874196 | 7.5467523 | -3.759333 |
| ENSG00000232931.5 | 2.40E-86 | 7.474716 | 9.6605352 | -2.185819 |
| ENSG00000159403.15 | 9.62E-31 | 13.378663 | 14.786442 | -1.407779 |
| ENSG00000101280.7 | 1.25E-48 | 3.2298695 | 7.6137761 | -4.383907 |
| ENSG00000162881.6 | 3.45E-80 | 5.1270081 | 7.8368602 | -2.709852 |
| ENSG00000264176.1 | 5.64E-52 | 2.0881501 | 4.1501261 | -2.061976 |
| ENSG00000022267.16 | 3.87E-18 | 10.601087 | 11.665398 | -1.06431 |
| ENSG00000221125.2 | 7.37E-31 | 1.0590126 | 3.7655364 | -2.706524 |
| ENSG00000259125.1 | 2.17E-20 | 0.5576644 | 1.6567227 | -1.099058 |
| ENSG00000271699.5 | 6.41E-113 | 3.7006778 | 7.4512432 | -3.750565 |
| ENSG00000102053.12 | 6.79E-78 | 6.128037 | 7.8195864 | -1.691549 |
| ENSG00000150627.15 | 2.24E-52 | 4.90011 | 7.102258 | -2.202148 |
| ENSG00000115226.9 | 4.60E-50 | 8.077748 | 10.075236 | -1.997488 |
| ENSG00000151778.10 | 1.01E-56 | 6.3142831 | 7.5747023 | -1.260419 |
| ENSG00000226194.5 | 1.78E-26 | 4.1200263 | 5.7452966 | -1.62527 |
| ENSG00000260844.2 | 4.21E-17 | 0.5021162 | 2.0698193 | -1.567703 |
| ENSG00000231249.1 | 6.34E-26 | 2.7612878 | 4.1034 | -1.342112 |
| ENSG00000228275.1 | 1.84E-17 | 1.2481399 | 2.5613523 | -1.313212 |
| ENSG00000106823.12 | 4.90E-95 | 7.7498508 | 10.741113 | -2.991262 |
| ENSG00000146414.15 | 1.96E-46 | 8.8674439 | 9.8763364 | -1.008892 |
| ENSG00000279159.1 | 1.55E-58 | 7.4897594 | 8.9408864 | -1.451127 |
| ENSG00000065609.14 | 1.68E-30 | 3.8626644 | 5.7856693 | -1.923005 |
| ENSG00000172867.3 | 6.41E-29 | 0.5048766 | 3.7407034 | -3.235827 |
| ENSG00000273137.1 | 2.72E-82 | 3.8442394 | 5.9116273 | -2.067388 |
| ENSG00000255837.1 | 5.56E-59 | 3.4098179 | 5.5283034 | -2.118486 |
| ENSG00000077009.13 | 1.82E-18 | 0.6783274 | 2.5301091 | -1.851782 |
| ENSG00000260266.1 | 1.70E-46 | 2.7932527 | 6.2472602 | -3.454007 |
| ENSG00000182333.14 | 5.95E-25 | 1.4645317 | 4.2885557 | -2.824024 |
| ENSG00000231154.1 | 1.49E-44 | 4.4698532 | 6.7228659 | -2.253013 |
| ENSG00000166676.14 | 6.78E-34 | 6.7229002 | 8.2102227 | -1.487322 |
| ENSG00000214457.3 | 6.39E-18 | 0.102543 | 1.2452648 | -1.142722 |
| ENSG00000274751.1 | 1.63E-23 | 3.3686881 | 4.5370568 | -1.168369 |
| ENSG00000261611.5 | 3.67E-14 | 3.4074881 | 4.5443989 | -1.136911 |
| ENSG00000185386.14 | 1.76E-68 | 6.942631 | 8.8699375 | -1.927306 |
| ENSG00000185739.13 | 2.49E-08 | 4.645385 | 5.6939727 | -1.048588 |
| ENSG00000129675.15 | 4.26E-55 | 8.6005821 | 9.8543932 | -1.253811 |
| ENSG00000231609.5 | 7.35E-115 | 4.3061181 | 7.1147023 | -2.808584 |
| ENSG00000116678.18 | 1.49E-28 | 8.6364811 | 10.184381 | -1.5479 |
| ENSG00000140400.14 | 2.46E-59 | 11.35727 | 12.62595 | -1.26868 |
| ENSG00000104324.15 | 4.68E-42 | 10.033237 | 11.064006 | -1.030769 |
| ENSG00000266074.8 | 3.13E-118 | 7.8900239 | 10.514573 | -2.624549 |
| ENSG00000259298.1 | 1.81E-51 | 2.1527652 | 3.9901318 | -1.837367 |
| ENSG00000277494.1 | 2.69E-21 | 5.5299733 | 7.3439614 | -1.813988 |
| ENSG00000259774.1 | 1.12E-18 | 3.3682699 | 4.432717 | -1.064447 |
| ENSG00000167711.13 | 1.51E-20 | 6.2684045 | 7.8468932 | -1.578489 |
| ENSG00000230521.1 | 2.02E-32 | 4.2827947 | 5.7491045 | -1.46631 |
| ENSG00000187021.14 | 9.59E-28 | 0.4405807 | 2.9275045 | -2.486924 |
| ENSG00000278772.1 | 6.63E-21 | 0.4998074 | 1.7778648 | -1.278057 |
| ENSG00000237669.1 | 2.33E-15 | 3.7014757 | 4.7957227 | -1.094247 |
| ENSG00000165379.13 | 2.53E-32 | 3.5880484 | 5.6646511 | -2.076603 |
| ENSG00000254528.7 | 1.53E-30 | 4.3948126 | 5.9761568 | -1.581344 |
| ENSG00000229029.2 | 4.12E-23 | 1.2163952 | 2.3251841 | -1.108789 |
| ENSG00000260230.2 | 2.80E-63 | 6.2770396 | 9.8287136 | -3.551674 |
| ENSG00000234405.1 | 3.55E-11 | 1.8664747 | 2.874083 | -1.007608 |
| ENSG00000100650.15 | 4.48E-73 | 12.652995 | 14.008431 | -1.355436 |
| ENSG00000248019.2 | 3.36E-110 | 5.332785 | 8.1609864 | -2.828201 |
| ENSG00000129824.15 | 5.31E-53 | 0.0321771 | 2.8974886 | -2.865312 |
| ENSG00000250334.5 | 1.12E-24 | 0.8020661 | 3.1741034 | -2.372037 |
| ENSG00000112936.18 | 1.63E-103 | 9.3807532 | 15.480302 | -6.099549 |
| ENSG00000157103.10 | 1.17E-34 | 4.7941914 | 6.9645341 | -2.170343 |
| ENSG00000256674.1 | 2.44E-26 | 0.2669442 | 1.6762557 | -1.409312 |
| ENSG00000279119.1 | 2.00E-114 | 2.4905981 | 6.9119034 | -4.421305 |
| ENSG00000168928.12 | 9.67E-28 | 1.6823451 | 5.0381182 | -3.355773 |
| ENSG00000257773.1 | 8.13E-39 | 2.5092656 | 4.1912125 | -1.681947 |
| ENSG00000260551.1 | 2.28E-19 | 0.1638936 | 1.5011705 | -1.337277 |
| ENSG00000122367.19 | 5.69E-20 | 5.6630473 | 7.7079909 | -2.044944 |
| ENSG00000155657.23 | 3.63E-64 | 7.2403902 | 8.9873591 | -1.746969 |
| ENSG00000215704.9 | 1.23E-26 | 0.4324649 | 2.4057943 | -1.973329 |
| ENSG00000100146.16 | 4.53E-21 | 0.5740578 | 4.1033295 | -3.529272 |
| ENSG00000120907.17 | 2.63E-30 | 1.1490881 | 4.0346341 | -2.885546 |
| ENSG00000278875.1 | 5.54E-107 | 1.5889723 | 6.7845955 | -5.195623 |
| ENSG00000263508.5 | 5.60E-37 | 0.7653864 | 2.5831352 | -1.817749 |
| ENSG00000119508.17 | 1.36E-20 | 6.9812394 | 10.091199 | -3.109959 |
| ENSG00000196569.11 | 2.22E-131 | 8.208289 | 12.969992 | -4.761703 |
| ENSG00000196381.10 | 1.19E-96 | 4.9996637 | 7.2497898 | -2.250126 |
| ENSG00000274487.1 | 8.68E-35 | 7.0449191 | 8.138408 | -1.093489 |
| ENSG00000166091.19 | 1.40E-09 | 1.5542592 | 2.6982625 | -1.144003 |
| ENSG00000266283.1 | 1.35E-37 | 2.2541427 | 4.4372114 | -2.183069 |
| ENSG00000122420.9 | 8.40E-77 | 3.3907969 | 7.2088795 | -3.818083 |
| ENSG00000163792.6 | 3.22E-69 | 6.5420919 | 10.062978 | -3.520887 |
| ENSG00000106631.8 | 2.02E-12 | 2.0236513 | 3.3834534 | -1.359802 |
| ENSG00000120729.9 | 1.49E-09 | 2.797273 | 3.8295136 | -1.032241 |
| ENSG00000198932.12 | 2.56E-188 | 7.0841315 | 12.280189 | -5.196057 |
| ENSG00000259551.1 | 4.26E-26 | 0.6391561 | 1.7073102 | -1.068154 |
| ENSG00000148541.12 | 1.50E-90 | 6.6300193 | 9.1210284 | -2.491009 |
| ENSG00000109061.9 | 6.29E-24 | 0.5013348 | 3.7131091 | -3.211774 |
| ENSG00000111860.13 | 1.20E-54 | 7.9921723 | 9.2705659 | -1.278394 |
| ENSG00000237940.3 | 6.70E-46 | 4.0675983 | 6.5615955 | -2.493997 |
| ENSG00000115290.9 | 2.10E-19 | 5.8345103 | 7.4641398 | -1.62963 |
| ENSG00000077264.14 | 7.25E-33 | 5.0327566 | 6.8499557 | -1.817199 |
| ENSG00000220563.1 | 5.59E-55 | 3.8346012 | 5.8668261 | -2.032225 |
| ENSG00000164169.12 | 1.52E-13 | 8.3236215 | 9.4931523 | -1.169531 |
| ENSG00000170807.11 | 3.36E-23 | 0.5967714 | 2.1243966 | -1.527625 |
| ENSG00000279569.1 | 1.23E-32 | 5.6761444 | 7.0403125 | -1.364168 |
| ENSG00000204789.4 | 8.40E-41 | 7.4012921 | 8.7347648 | -1.333473 |
| ENSG00000247982.6 | 1.62E-53 | 5.1244084 | 6.8424955 | -1.718087 |
| ENSG00000214274.9 | 4.64E-83 | 7.517048 | 9.6820375 | -2.16499 |
| ENSG00000272620.1 | 5.40E-09 | 6.8665449 | 7.9510705 | -1.084526 |
| ENSG00000196565.12 | 2.19E-23 | 2.0367496 | 3.8179625 | -1.781213 |
| ENSG00000064692.18 | 5.79E-75 | 7.0728563 | 10.043107 | -2.97025 |
| ENSG00000198010.11 | 1.60E-24 | 2.2931706 | 3.8060545 | -1.512884 |
| ENSG00000012124.14 | 4.69E-49 | 8.9458644 | 12.377867 | -3.432003 |
| ENSG00000264272.1 | 4.27E-06 | 1.2707845 | 2.5003807 | -1.229596 |
| ENSG00000166402.8 | 6.05E-86 | 8.7654289 | 10.842817 | -2.077388 |
| ENSG00000274602.4 | 5.17E-37 | 8.5763284 | 10.736967 | -2.160639 |
| ENSG00000231711.2 | 6.64E-59 | 7.0632683 | 9.1867455 | -2.123477 |
| ENSG00000260455.1 | 3.92E-12 | 1.8361845 | 2.8770909 | -1.040906 |
| ENSG00000255548.1 | 9.06E-35 | 0.1102356 | 2.157775 | -2.047539 |
| ENSG00000164574.15 | 2.83E-21 | 10.695505 | 11.82029 | -1.124784 |
| ENSG00000129204.16 | 4.97E-25 | 5.8713265 | 7.9204614 | -2.049135 |
| ENSG00000279161.1 | 8.16E-49 | 1.0063291 | 4.6549364 | -3.648607 |
| ENSG00000231340.1 | 7.80E-29 | 2.1408955 | 3.3355227 | -1.194627 |
| ENSG00000007908.15 | 1.02E-11 | 4.0528251 | 5.6530034 | -1.600178 |
| ENSG00000255893.1 | 7.20E-14 | 1.9676566 | 3.1713807 | -1.203724 |
| ENSG00000147231.13 | 3.19E-145 | 3.7689785 | 8.4473943 | -4.678416 |
| ENSG00000198754.5 | 4.07E-10 | 4.2807298 | 5.352175 | -1.071445 |
| ENSG00000141314.12 | 4.39E-79 | 4.5490628 | 7.1982636 | -2.649201 |
| ENSG00000004534.14 | 4.91E-51 | 11.176534 | 12.63458 | -1.458046 |
| ENSG00000268896.1 | 1.00E-60 | 1.1398296 | 4.575442 | -3.435612 |
| ENSG00000225849.1 | 1.10E-46 | 2.2072919 | 4.2674523 | -2.06016 |
| ENSG00000177599.12 | 4.54E-46 | 5.0113291 | 6.3118148 | -1.300486 |
| ENSG00000259763.1 | 1.49E-30 | 0.1165876 | 1.6110591 | -1.494472 |
| ENSG00000069535.13 | 1.27E-89 | 9.2376792 | 12.129926 | -2.892247 |
| ENSG00000150051.13 | 5.57E-17 | 7.2452764 | 8.2981773 | -1.052901 |
| ENSG00000258537.5 | 9.38E-39 | 0.2596191 | 3.2995568 | -3.039938 |
| ENSG00000169509.5 | 1.40E-11 | 0.302326 | 1.7219216 | -1.419596 |
| ENSG00000248115.1 | 1.31E-60 | 1.9513635 | 6.7927386 | -4.841375 |
| ENSG00000233478.1 | 1.39E-23 | 0.9675902 | 2.5545148 | -1.586925 |
| ENSG00000147454.13 | 1.95E-45 | 10.414245 | 11.719049 | -1.304804 |
| ENSG00000258708.1 | 2.21E-22 | 6.2596625 | 7.3237568 | -1.064094 |
| ENSG00000228549.3 | 3.05E-26 | 2.7146382 | 4.3589057 | -1.644267 |
| ENSG00000183171.5 | 1.59E-62 | 2.5763081 | 5.6000875 | -3.023779 |
| ENSG00000165424.6 | 2.60E-37 | 9.697326 | 10.985872 | -1.288546 |
| ENSG00000171791.11 | 3.41E-48 | 8.7431499 | 10.231595 | -1.488446 |
| ENSG00000183506.16 | 5.64E-44 | 9.4979575 | 10.954661 | -1.456704 |
| ENSG00000213642.3 | 9.41E-31 | 1.4302807 | 3.0979659 | -1.667685 |
| ENSG00000129946.10 | 3.41E-30 | 7.7687578 | 9.623542 | -1.854784 |
| ENSG00000273183.1 | 9.78E-45 | 3.7851021 | 5.1691989 | -1.384097 |
| ENSG00000206538.7 | 2.28E-48 | 7.4070093 | 9.542833 | -2.135824 |
| ENSG00000079102.16 | 3.86E-41 | 6.6272158 | 8.2394489 | -1.612233 |
| ENSG00000151650.7 | 2.54E-15 | 4.9417138 | 6.0803227 | -1.138609 |
| ENSG00000153246.11 | 1.54E-72 | 8.1513341 | 9.897342 | -1.746008 |
| ENSG00000006283.17 | 3.76E-46 | 6.3049143 | 9.0107375 | -2.705823 |
| ENSG00000184905.8 | 6.70E-103 | 6.5994874 | 10.238623 | -3.639135 |
| ENSG00000231768.1 | 1.25E-39 | 3.4989554 | 5.6746284 | -2.175673 |
| ENSG00000245648.1 | 7.27E-24 | 3.6007592 | 4.961017 | -1.360258 |
| ENSG00000223878.1 | 1.54E-51 | 2.6558208 | 4.5728034 | -1.916983 |
| ENSG00000216624.2 | 3.51E-15 | 1.032836 | 2.2870364 | -1.2542 |
| ENSG00000279940.1 | 5.07E-32 | 0.3646057 | 3.119875 | -2.755269 |
| ENSG00000153820.12 | 1.50E-35 | 0.8026227 | 5.3654955 | -4.562873 |
| ENSG00000251226.1 | 7.37E-30 | 2.0263551 | 4.3806898 | -2.354335 |
| ENSG00000162552.14 | 1.42E-19 | 7.4404191 | 9.240933 | -1.800514 |
| ENSG00000012504.13 | 2.14E-70 | 1.6590379 | 7.9541341 | -6.295096 |
| ENSG00000269235.1 | 1.15E-12 | 2.8468098 | 4.0699125 | -1.223103 |
| ENSG00000075826.16 | 9.86E-126 | 7.5635272 | 10.16888 | -2.605352 |
| ENSG00000237212.1 | 8.03E-21 | 0.2289964 | 1.4902818 | -1.261285 |
| ENSG00000244055.1 | 3.95E-34 | 4.1145029 | 5.1701307 | -1.055628 |
| ENSG00000154822.15 | 4.51E-47 | 7.9948468 | 9.4750443 | -1.480198 |
| ENSG00000112541.13 | 1.31E-28 | 6.5679055 | 8.2755648 | -1.707659 |
| ENSG00000180881.19 | 2.08E-62 | 5.925732 | 7.4251057 | -1.499374 |
| ENSG00000074047.20 | 5.06E-28 | 8.7717914 | 10.098203 | -1.326412 |
| ENSG00000105398.3 | 1.64E-11 | 0.1238217 | 1.3041545 | -1.180333 |
| ENSG00000225194.2 | 2.28E-66 | 3.3157766 | 6.4232068 | -3.10743 |
| ENSG00000132837.14 | 2.56E-27 | 5.4356411 | 6.6229977 | -1.187357 |
| ENSG00000231875.1 | 8.38E-39 | 0.6093165 | 2.2516977 | -1.642381 |
| ENSG00000251532.1 | 9.39E-22 | 1.469437 | 3.110317 | -1.64088 |
| ENSG00000237916.1 | 1.24E-22 | 0.7199907 | 1.9450011 | -1.22501 |
| ENSG00000261451.1 | 2.12E-24 | 1.7251098 | 3.0947102 | -1.3696 |
| ENSG00000235821.1 | 3.32E-19 | 0.4665437 | 1.7335341 | -1.26699 |
| ENSG00000262180.1 | 1.71E-45 | 2.7256017 | 4.0812636 | -1.355662 |
| ENSG00000196368.4 | 1.12E-49 | 5.6399286 | 7.837433 | -2.197504 |
| ENSG00000003137.8 | 5.84E-14 | 6.5388857 | 7.7597591 | -1.220873 |
| ENSG00000261997.1 | 3.11E-45 | 3.9040143 | 5.987883 | -2.083869 |
| ENSG00000166183.15 | 1.17E-107 | 6.0309057 | 9.6289841 | -3.598078 |
| ENSG00000235373.1 | 8.78E-66 | 6.3419988 | 8.3860341 | -2.044035 |
| ENSG00000183426.15 | 7.64E-72 | 11.017982 | 12.569105 | -1.551122 |
| ENSG00000124564.17 | 1.72E-26 | 0.7085119 | 2.9291818 | -2.22067 |
| ENSG00000228852.6 | 1.78E-19 | 1.7277434 | 2.810133 | -1.08239 |
| ENSG00000213906.9 | 4.79E-44 | 6.5331148 | 7.835767 | -1.302652 |
| ENSG00000165863.16 | 2.71E-14 | 5.2155714 | 6.5637659 | -1.348195 |
| ENSG00000231690.2 | 8.38E-55 | 1.7346919 | 3.849158 | -2.114466 |
| ENSG00000110852.4 | 5.81E-27 | 8.2185265 | 9.3813705 | -1.162844 |
| ENSG00000123342.15 | 8.45E-12 | 8.8449081 | 9.9150682 | -1.07016 |
| ENSG00000141052.17 | 6.89E-67 | 4.3810895 | 8.7512648 | -4.370175 |
| ENSG00000258465.6 | 1.14E-26 | 1.6340308 | 3.2760727 | -1.642042 |
| ENSG00000267636.1 | 3.22E-51 | 0.1678819 | 4.315417 | -4.147535 |
| ENSG00000260484.1 | 2.09E-44 | 1.3450998 | 4.1657636 | -2.820664 |
| ENSG00000241984.2 | 6.69E-28 | 0.6614871 | 2.0922341 | -1.430747 |
| ENSG00000260570.1 | 3.31E-41 | 0.9338587 | 4.3847045 | -3.450846 |
| ENSG00000250490.1 | 3.01E-45 | 2.998159 | 5.1186807 | -2.120522 |
| ENSG00000143816.7 | 1.18E-16 | 8.0419819 | 9.135942 | -1.09396 |
| ENSG00000234617.1 | 1.72E-21 | 1.4467107 | 2.753575 | -1.306864 |
| ENSG00000158482.9 | 6.16E-20 | 1.7619539 | 2.958817 | -1.196863 |
| ENSG00000279932.1 | 5.64E-25 | 0.4540955 | 2.353075 | -1.89898 |
| ENSG00000174498.13 | 2.34E-09 | 4.0540475 | 5.1309057 | -1.076858 |
| ENSG00000275152.4 | 6.53E-19 | 0.3285678 | 2.0631295 | -1.734562 |
| ENSG00000225366.4 | 8.33E-11 | 0.7157771 | 2.0685409 | -1.352764 |
| ENSG00000068781.20 | 6.81E-08 | 2.4943005 | 3.7886 | -1.2943 |
| ENSG00000273987.1 | 9.93E-55 | 2.4473943 | 4.3948591 | -1.947465 |
| ENSG00000261386.2 | 2.38E-25 | 2.7060539 | 3.8851989 | -1.179145 |
| ENSG00000088926.13 | 2.29E-17 | 0.4075804 | 1.5545739 | -1.146993 |
| ENSG00000278769.1 | 1.05E-18 | 2.6955005 | 4.4793705 | -1.78387 |
| ENSG00000272836.1 | 1.41E-80 | 3.2215814 | 5.293142 | -2.071561 |
| ENSG00000245532.5 | 7.85E-59 | 13.551419 | 15.754974 | -2.203555 |
| ENSG00000134202.10 | 2.11E-55 | 9.578132 | 11.78647 | -2.208338 |
| ENSG00000148053.15 | 1.20E-32 | 7.3338888 | 9.4150227 | -2.081134 |
| ENSG00000250327.1 | 1.24E-45 | 2.1159909 | 3.7403102 | -1.624319 |
| ENSG00000279894.1 | 1.48E-67 | 3.5388074 | 5.8529682 | -2.314161 |
| ENSG00000236289.2 | 7.85E-18 | 0.634811 | 1.8226068 | -1.187796 |
| ENSG00000249669.7 | 3.45E-78 | 5.9798924 | 10.158252 | -4.17836 |
| ENSG00000270040.1 | 7.77E-37 | 1.2942475 | 3.1383148 | -1.844067 |
| ENSG00000108799.12 | 5.57E-87 | 10.210119 | 11.86842 | -1.658302 |
| ENSG00000163958.13 | 1.23E-18 | 4.2710644 | 5.8210727 | -1.550008 |
| ENSG00000274769.1 | 1.78E-15 | 1.4922971 | 2.6967125 | -1.204415 |
| ENSG00000272139.1 | 1.65E-27 | 0.1943561 | 2.149825 | -1.955469 |
| ENSG00000151491.12 | 1.62E-42 | 9.8640797 | 11.238184 | -1.374104 |
| ENSG00000175967.3 | 8.99E-56 | 1.5777212 | 4.0941364 | -2.516415 |
| ENSG00000253105.5 | 7.94E-25 | 0.3868778 | 2.0411114 | -1.654234 |
| ENSG00000196970.8 | 2.38E-16 | 0.5204685 | 1.8651977 | -1.344729 |
| ENSG00000254585.2 | 4.82E-18 | 5.5046831 | 6.9817716 | -1.477089 |
| ENSG00000233327.10 | 3.18E-25 | 4.6431048 | 8.0039455 | -3.360841 |
| ENSG00000104722.13 | 2.79E-37 | 1.7690465 | 5.4078807 | -3.638834 |
| ENSG00000249767.1 | 6.50E-36 | 1.2030258 | 3.3021898 | -2.099164 |
| ENSG00000160282.13 | 8.37E-15 | 5.0568957 | 6.2544557 | -1.19756 |
| ENSG00000149488.12 | 7.57E-16 | 1.8778081 | 2.9260011 | -1.048193 |
| ENSG00000189134.3 | 3.43E-69 | 2.7212539 | 4.6956909 | -1.974437 |
| ENSG00000127364.3 | 7.47E-19 | 4.316327 | 5.4131318 | -1.096805 |
| ENSG00000124440.15 | 3.49E-17 | 9.6216976 | 11.344636 | -1.722939 |
| ENSG00000160097.15 | 1.91E-28 | 6.1004048 | 7.7105398 | -1.610135 |
| ENSG00000269920.1 | 7.53E-10 | 1.2681745 | 2.4012682 | -1.133094 |
| ENSG00000189292.15 | 3.33E-68 | 5.6072826 | 9.6222739 | -4.014991 |
| ENSG00000261366.1 | 6.36E-33 | 4.3207406 | 5.8492909 | -1.52855 |
| ENSG00000198624.12 | 3.86E-51 | 8.7783382 | 10.421001 | -1.642663 |
| ENSG00000272983.1 | 1.21E-62 | 4.3225258 | 6.0426114 | -1.720086 |
| ENSG00000061455.10 | 5.81E-52 | 5.7762907 | 7.9903045 | -2.214014 |
| ENSG00000250846.6 | 1.36E-21 | 0.5631496 | 2.2568761 | -1.693726 |
| ENSG00000198626.15 | 2.11E-44 | 6.6763291 | 9.1038807 | -2.427552 |
| ENSG00000251034.1 | 3.26E-28 | 4.0984955 | 5.2948932 | -1.196398 |
| ENSG00000215388.3 | 6.48E-34 | 3.2463499 | 4.5942034 | -1.347854 |
| ENSG00000196136.16 | 5.93E-26 | 7.6847893 | 10.021369 | -2.33658 |
| ENSG00000224958.5 | 1.47E-18 | 2.0006871 | 3.3907602 | -1.390073 |
| ENSG00000004799.7 | 1.99E-64 | 9.5810296 | 13.12331 | -3.542281 |
| ENSG00000262096.2 | 1.61E-59 | 2.4270348 | 4.4046409 | -1.977606 |
| ENSG00000167280.16 | 5.97E-71 | 9.4604752 | 11.237106 | -1.776631 |
| ENSG00000235563.1 | 8.79E-60 | 0.7670148 | 5.0276648 | -4.26065 |
| ENSG00000091536.16 | 3.41E-41 | 6.6501413 | 8.0582864 | -1.408145 |
| ENSG00000106113.18 | 1.30E-15 | 2.9809582 | 4.2409216 | -1.259963 |
| ENSG00000227245.1 | 2.39E-25 | 0.3306327 | 1.7562625 | -1.42563 |
| ENSG00000165507.8 | 4.12E-12 | 11.052272 | 12.488215 | -1.435943 |
| ENSG00000279030.1 | 3.72E-17 | 1.6541685 | 3.0586375 | -1.404469 |
| ENSG00000214544.7 | 4.03E-45 | 3.8852539 | 5.6134875 | -1.728234 |
| ENSG00000108387.14 | 2.62E-42 | 7.8046365 | 9.068683 | -1.264046 |
| ENSG00000272573.5 | 3.43E-45 | 2.5473489 | 7.1325455 | -4.585197 |
| ENSG00000115461.4 | 4.05E-67 | 13.727003 | 16.896616 | -3.169613 |
| ENSG00000279662.1 | 3.05E-20 | 5.3389928 | 6.3491534 | -1.010161 |
| ENSG00000187893.10 | 4.63E-15 | 0.516501 | 1.6751193 | -1.158618 |
| ENSG00000185988.11 | 6.19E-32 | 1.0242482 | 3.5062136 | -2.481965 |
| ENSG00000254414.1 | 2.01E-38 | 2.1620893 | 3.6601955 | -1.498106 |
| ENSG00000277561.4 | 1.50E-63 | 0.8761964 | 4.422625 | -3.546429 |
| ENSG00000249242.7 | 5.19E-113 | 7.290448 | 9.9912943 | -2.700846 |
| ENSG00000003756.16 | 2.36E-74 | 11.75569 | 13.161948 | -1.406258 |
| ENSG00000260953.1 | 2.67E-20 | 1.5137406 | 2.7061568 | -1.192416 |
| ENSG00000100767.15 | 8.88E-22 | 8.8313439 | 10.221634 | -1.39029 |
| ENSG00000145335.15 | 4.50E-48 | 7.2011905 | 8.8069955 | -1.605805 |
| ENSG00000132970.12 | 1.77E-35 | 9.0731967 | 10.317434 | -1.244237 |
| ENSG00000163637.11 | 7.66E-31 | 8.9980525 | 10.009165 | -1.011112 |
| ENSG00000254715.3 | 1.47E-19 | 1.9010267 | 4.0119602 | -2.110933 |
| ENSG00000271382.1 | 1.24E-50 | 1.5271611 | 3.4135841 | -1.886423 |
| ENSG00000132972.18 | 3.12E-06 | 0.3425876 | 1.4471523 | -1.104565 |
| ENSG00000279693.1 | 2.31E-20 | 1.8192174 | 3.123908 | -1.304691 |
| ENSG00000275131.1 | 1.83E-28 | 7.4879422 | 8.5930216 | -1.105079 |
| ENSG00000159648.11 | 2.32E-13 | 5.0981198 | 6.1861761 | -1.088056 |
| ENSG00000167281.18 | 3.70E-27 | 2.9789685 | 4.8000534 | -1.821085 |
| ENSG00000138823.12 | 1.87E-22 | 3.8158909 | 6.0738114 | -2.25792 |
| ENSG00000278341.1 | 1.09E-36 | 3.1925072 | 4.6458239 | -1.453317 |
| ENSG00000237070.1 | 4.70E-23 | 0.301964 | 2.2211295 | -1.919166 |
| ENSG00000162882.14 | 4.08E-79 | 6.8523341 | 8.4036886 | -1.551355 |
| ENSG00000152207.7 | 4.14E-07 | 4.7237274 | 5.7799716 | -1.056244 |
| ENSG00000237187.8 | 4.39E-41 | 7.3465103 | 8.8866693 | -1.540159 |
| ENSG00000175206.10 | 3.15E-22 | 1.8572446 | 4.5587989 | -2.701554 |
| ENSG00000276337.1 | 4.47E-26 | 3.0168191 | 4.833042 | -1.816223 |
| ENSG00000268560.1 | 4.29E-33 | 2.5116838 | 5.6267614 | -3.115078 |
| ENSG00000184205.14 | 3.32E-95 | 9.9742847 | 14.101038 | -4.126753 |
| ENSG00000128606.12 | 1.94E-51 | 7.3510618 | 10.350472 | -2.99941 |
| ENSG00000197813.5 | 3.85E-67 | 3.3147537 | 5.2864159 | -1.971662 |
| ENSG00000198077.10 | 9.15E-25 | 1.0192451 | 3.3151239 | -2.295879 |
| ENSG00000220392.1 | 6.41E-30 | 0.5820358 | 1.7078284 | -1.125793 |
| ENSG00000278045.1 | 8.43E-31 | 1.2231212 | 3.0442307 | -1.821109 |
| ENSG00000102924.11 | 1.21E-09 | 3.7286115 | 4.7627977 | -1.034186 |
| ENSG00000249307.5 | 8.55E-25 | 3.1306229 | 5.9850852 | -2.854462 |
| ENSG00000225206.5 | 1.46E-17 | 0.4509174 | 2.1465352 | -1.695618 |
| ENSG00000278934.1 | 1.25E-80 | 3.3025069 | 6.4986636 | -3.196157 |
| ENSG00000261568.1 | 6.56E-35 | 2.1835394 | 4.5426057 | -2.359066 |
| ENSG00000152779.13 | 5.82E-24 | 3.1504499 | 5.6903886 | -2.539939 |
| ENSG00000170074.19 | 7.43E-30 | 5.0419382 | 7.0608955 | -2.018957 |
| ENSG00000136449.13 | 1.47E-46 | 5.0955126 | 7.4213318 | -2.325819 |
| ENSG00000279108.1 | 1.04E-41 | 4.6560389 | 6.342808 | -1.686769 |
| ENSG00000280339.1 | 4.52E-62 | 6.5926372 | 8.4631455 | -1.870508 |
| ENSG00000226306.6 | 9.26E-24 | 2.4981737 | 3.6898341 | -1.19166 |
| ENSG00000163815.5 | 2.04E-17 | 6.2877107 | 7.7399341 | -1.452223 |
| ENSG00000179636.14 | 9.74E-65 | 0.9043981 | 3.3094409 | -2.405043 |
| ENSG00000262118.1 | 1.26E-44 | 1.5233494 | 4.0110716 | -2.487722 |
| ENSG00000128262.8 | 4.29E-21 | 4.2420618 | 6.2615625 | -2.019501 |
| ENSG00000135976.16 | 8.63E-71 | 8.1162031 | 9.8663045 | -1.750101 |
| ENSG00000159433.11 | 1.30E-195 | 7.8144962 | 11.481567 | -3.667071 |
| ENSG00000206127.10 | 4.53E-40 | 1.8469893 | 5.4486398 | -3.601651 |
| ENSG00000253476.1 | 4.52E-79 | 1.0896597 | 3.6893841 | -2.599724 |
| ENSG00000271430.1 | 2.02E-39 | 8.3936418 | 9.4333068 | -1.039665 |
| ENSG00000182916.7 | 2.05E-109 | 5.7297759 | 8.5706818 | -2.840906 |
| ENSG00000228485.1 | 2.31E-21 | 0.1118408 | 1.5536352 | -1.441794 |
| ENSG00000158859.9 | 5.21E-24 | 8.4127174 | 11.801363 | -3.388645 |
| ENSG00000198739.10 | 1.80E-36 | 1.286569 | 4.6748932 | -3.388324 |
| ENSG00000114654.7 | 7.91E-30 | 6.3088036 | 8.057275 | -1.748471 |
| ENSG00000250329.1 | 3.71E-14 | 0.6529353 | 1.8515057 | -1.19857 |
| ENSG00000236754.5 | 2.28E-15 | 1.1181878 | 2.2069693 | -1.088781 |
| ENSG00000108797.11 | 4.49E-85 | 8.2905014 | 10.490043 | -2.199542 |
| ENSG00000261114.1 | 5.45E-39 | 3.5866198 | 5.8965909 | -2.309971 |
| ENSG00000236333.3 | 1.62E-102 | 4.0621394 | 8.9191261 | -4.856987 |
| ENSG00000143125.5 | 1.13E-126 | 3.593469 | 11.647234 | -8.053765 |
| ENSG00000256040.2 | 1.29E-23 | 2.4699885 | 4.1401477 | -1.670159 |
| ENSG00000269483.1 | 6.26E-19 | 1.2452632 | 2.5128648 | -1.267602 |
| ENSG00000069966.18 | 8.85E-68 | 9.713748 | 10.874573 | -1.160825 |
| ENSG00000260613.1 | 2.32E-27 | 1.2047076 | 2.7166136 | -1.511906 |
| ENSG00000164129.11 | 2.24E-09 | 0.697643 | 1.7824898 | -1.084847 |
| ENSG00000026036.20 | 1.30E-39 | 9.0863702 | 10.107288 | -1.020917 |
| ENSG00000133800.8 | 5.18E-16 | 7.3270442 | 8.9826841 | -1.65564 |
| ENSG00000132840.9 | 1.42E-80 | 5.2495809 | 7.9891614 | -2.73958 |
| ENSG00000278484.1 | 6.55E-21 | 0.2021458 | 2.9031682 | -2.701022 |
| ENSG00000125851.9 | 7.06E-46 | 5.0729895 | 8.537 | -3.464011 |
| ENSG00000279744.1 | 1.30E-27 | 4.2898432 | 5.6297614 | -1.339918 |
| ENSG00000153002.11 | 1.85E-09 | 3.6026107 | 5.0490205 | -1.44641 |
| ENSG00000271576.1 | 2.63E-32 | 4.9936702 | 6.3051295 | -1.311459 |
| ENSG00000232018.4 | 4.55E-61 | 0.8581442 | 4.4306182 | -3.572474 |
| ENSG00000050030.13 | 2.23E-33 | 4.3508124 | 5.9257523 | -1.57494 |
| ENSG00000273041.1 | 1.01E-26 | 1.0600616 | 2.1662466 | -1.106185 |
| ENSG00000273138.1 | 2.13E-39 | 0.985742 | 2.8168239 | -1.831082 |
| ENSG00000186867.10 | 2.52E-14 | 0.8228816 | 1.9084102 | -1.085529 |
| ENSG00000196739.14 | 7.55E-72 | 9.599799 | 11.941351 | -2.341552 |
| ENSG00000225706.1 | 3.86E-82 | 3.1040048 | 7.1086966 | -4.004692 |
| ENSG00000101440.9 | 2.24E-59 | 2.6852988 | 7.680892 | -4.995593 |
| ENSG00000143196.4 | 1.99E-21 | 5.1385508 | 7.9785386 | -2.839988 |
| ENSG00000156219.16 | 7.40E-11 | 1.7678138 | 2.8354102 | -1.067596 |
| ENSG00000279192.1 | 8.94E-97 | 3.7065005 | 6.8498125 | -3.143312 |
| ENSG00000281183.1 | 1.68E-72 | 4.7111296 | 6.654683 | -1.943553 |
| ENSG00000214563.2 | 1.93E-18 | 0.6955527 | 1.9123955 | -1.216843 |
| ENSG00000161381.13 | 9.02E-32 | 8.7988897 | 10.576807 | -1.777917 |
| ENSG00000004848.6 | 2.19E-110 | 3.7815255 | 11.302623 | -7.521097 |
| ENSG00000170801.9 | 8.60E-59 | 9.3752444 | 11.62983 | -2.254585 |
| ENSG00000155816.19 | 8.62E-55 | 4.0952 | 6.794233 | -2.699033 |
| ENSG00000223812.5 | 7.52E-14 | 1.7089757 | 3.1058864 | -1.396911 |
| ENSG00000277741.4 | 1.27E-87 | 0.7055611 | 4.8753011 | -4.16974 |
| ENSG00000262410.1 | 3.19E-21 | 4.0493618 | 6.2442705 | -2.194909 |
| ENSG00000176533.12 | 2.43E-46 | 8.1447222 | 9.6768045 | -1.532082 |
| ENSG00000132693.12 | 5.02E-23 | 0.5276174 | 3.1018273 | -2.57421 |
| ENSG00000276141.4 | 1.24E-134 | 4.7911379 | 7.9004852 | -3.109347 |
| ENSG00000187268.11 | 1.26E-09 | 1.4618265 | 3.7082773 | -2.246451 |
| ENSG00000257524.5 | 1.88E-71 | 5.4773847 | 7.7988477 | -2.321463 |
| ENSG00000272040.1 | 2.85E-33 | 3.8225253 | 5.2100432 | -1.387518 |
| ENSG00000259498.1 | 3.56E-22 | 4.5691718 | 6.2443227 | -1.675151 |
| ENSG00000224940.8 | 9.18E-16 | 3.732148 | 5.0215159 | -1.289368 |
| ENSG00000175646.3 | 1.19E-17 | 0.2081007 | 1.9158193 | -1.707719 |
| ENSG00000261529.1 | 3.81E-25 | 0.933142 | 2.561217 | -1.628075 |
| ENSG00000279384.1 | 3.55E-25 | 2.0703234 | 3.5749455 | -1.504622 |
| ENSG00000165269.12 | 8.51E-36 | 4.521299 | 6.246217 | -1.724918 |
| ENSG00000259039.1 | 7.82E-25 | 0.634701 | 2.0139 | -1.379199 |
| ENSG00000119138.4 | 1.33E-25 | 10.006142 | 11.967193 | -1.961051 |
| ENSG00000232803.1 | 1.19E-08 | 1.5342933 | 2.822733 | -1.28844 |
| ENSG00000229598.1 | 3.20E-51 | 2.3278511 | 4.5505057 | -2.222655 |
| ENSG00000237926.1 | 3.85E-24 | 0.4859769 | 1.5620114 | -1.076035 |
| ENSG00000237438.6 | 6.01E-47 | 6.002374 | 8.6313898 | -2.629016 |
| ENSG00000103056.11 | 4.67E-46 | 6.9764704 | 8.5319614 | -1.555491 |
| ENSG00000274682.1 | 9.87E-24 | 0.1795017 | 1.4494739 | -1.269972 |
| ENSG00000267385.1 | 1.05E-80 | 2.7845914 | 8.7539864 | -5.969395 |
| ENSG00000204290.10 | 5.62E-22 | 0.9415936 | 2.2425273 | -1.300934 |
| ENSG00000277010.1 | 3.17E-60 | 0.4600248 | 4.6068477 | -4.146823 |
| ENSG00000263155.5 | 5.67E-22 | 5.5823742 | 6.851692 | -1.269318 |
| ENSG00000151623.14 | 4.35E-48 | 7.8872542 | 9.2293977 | -1.342144 |
| ENSG00000269067.1 | 6.47E-27 | 2.5839356 | 4.289467 | -1.705531 |
| ENSG00000197291.8 | 1.18E-80 | 4.5684274 | 7.2650932 | -2.696666 |
| ENSG00000185823.3 | 1.06E-38 | 1.4813487 | 5.5428034 | -4.061455 |
| ENSG00000258498.6 | 1.21E-200 | 5.207174 | 12.483339 | -7.276165 |
| ENSG00000263177.1 | 5.86E-48 | 1.1364847 | 3.5945193 | -2.458035 |
| ENSG00000271945.1 | 5.55E-48 | 0.1685451 | 4.3701523 | -4.201607 |
| ENSG00000183580.9 | 8.49E-45 | 9.3361341 | 10.727244 | -1.39111 |
| ENSG00000111077.17 | 1.14E-70 | 11.706891 | 13.371484 | -1.664593 |
| ENSG00000248429.5 | 1.54E-92 | 4.9831248 | 8.6361205 | -3.652996 |
| ENSG00000272037.1 | 7.95E-32 | 4.8057539 | 6.167967 | -1.362213 |
| ENSG00000189367.14 | 1.03E-68 | 4.0228518 | 6.8461148 | -2.823263 |
| ENSG00000100842.12 | 1.52E-67 | 9.000347 | 11.016249 | -2.015902 |
| ENSG00000269300.1 | 6.66E-22 | 0.8790895 | 2.0711977 | -1.192108 |
| ENSG00000163586.9 | 1.14E-15 | 0.8052444 | 1.9536273 | -1.148383 |
| ENSG00000212125.2 | 5.05E-28 | 2.7675064 | 3.939033 | -1.171527 |
| ENSG00000165623.9 | 1.06E-10 | 0.6524279 | 1.784567 | -1.132139 |
| ENSG00000227321.2 | 2.11E-28 | 0.7305033 | 2.137433 | -1.40693 |
| ENSG00000131389.16 | 1.63E-16 | 10.88472 | 12.087618 | -1.202899 |
| ENSG00000145708.10 | 2.32E-17 | 3.2230726 | 4.7909784 | -1.567906 |
| ENSG00000189136.8 | 9.95E-114 | 5.0662033 | 8.1166602 | -3.050457 |
| ENSG00000172465.13 | 9.70E-52 | 9.6098129 | 11.622285 | -2.012472 |
| ENSG00000127366.5 | 1.18E-39 | 4.4991893 | 5.904167 | -1.404978 |
| ENSG00000173258.12 | 8.28E-68 | 4.9580757 | 7.0275352 | -2.06946 |
| ENSG00000189186.10 | 7.03E-07 | 1.132279 | 2.1434511 | -1.011172 |
| ENSG00000145362.16 | 2.82E-55 | 8.4075773 | 10.442331 | -2.034753 |
| ENSG00000099715.14 | 5.90E-18 | 0.3026043 | 1.7065 | -1.403896 |
| ENSG00000237861.1 | 1.87E-22 | 0.7143659 | 2.1503318 | -1.435966 |
| ENSG00000254912.2 | 1.18E-33 | 2.295685 | 4.0985682 | -1.802883 |
| ENSG00000227438.1 | 1.05E-29 | 0.6515578 | 2.0001534 | -1.348596 |
| ENSG00000140488.14 | 2.96E-79 | 6.5903773 | 9.343817 | -2.75344 |
| ENSG00000205923.3 | 1.06E-58 | 4.1918184 | 6.6985443 | -2.506726 |
| ENSG00000133019.11 | 4.93E-12 | 6.5257239 | 7.697308 | -1.171584 |
| ENSG00000241794.1 | 6.51E-22 | 0.9152112 | 3.198142 | -2.282931 |
| ENSG00000235996.1 | 2.00E-20 | 0.0786685 | 1.321308 | -1.242639 |
| ENSG00000063127.15 | 2.59E-65 | 5.5564014 | 7.7504136 | -2.194012 |
| ENSG00000039537.13 | 2.51E-46 | 3.7468346 | 7.9291636 | -4.182329 |
| ENSG00000164106.7 | 5.08E-19 | 2.6180852 | 4.5265807 | -1.908495 |
| ENSG00000261051.1 | 5.19E-30 | 4.1764881 | 5.8865364 | -1.710048 |
| ENSG00000272446.5 | 8.66E-18 | 0.2554582 | 1.4497648 | -1.194307 |
| ENSG00000227487.3 | 4.13E-82 | 1.1350081 | 4.773783 | -3.638775 |
| ENSG00000238260.1 | 3.46E-14 | 2.4082542 | 3.4643443 | -1.05609 |
| ENSG00000243055.1 | 1.26E-21 | 0.9054325 | 1.9377966 | -1.032364 |
| ENSG00000186105.7 | 7.36E-51 | 4.9102969 | 6.8008409 | -1.890544 |
| ENSG00000213244.3 | 1.19E-68 | 1.4239609 | 4.5784955 | -3.154535 |
| ENSG00000171772.15 | 1.21E-19 | 3.6335442 | 5.6742977 | -2.040754 |
| ENSG00000162687.16 | 4.75E-84 | 5.9490983 | 9.2794341 | -3.330336 |
| ENSG00000261615.6 | 8.24E-15 | 0.5777072 | 1.6469318 | -1.069225 |
| ENSG00000091138.12 | 1.90E-19 | 0.7174413 | 2.2944489 | -1.577008 |
| ENSG00000138678.10 | 1.73E-55 | 6.2448511 | 9.1141636 | -2.869313 |
| ENSG00000134853.11 | 6.35E-124 | 9.6575747 | 13.762739 | -4.105164 |
| ENSG00000270704.3 | 1.92E-41 | 0.9052463 | 2.4077034 | -1.502457 |
| ENSG00000121440.14 | 9.58E-71 | 9.4449277 | 11.533663 | -2.088735 |
| ENSG00000255389.1 | 4.18E-44 | 5.6907606 | 7.3728364 | -1.682076 |
| ENSG00000118985.14 | 4.40E-11 | 9.0060511 | 10.221557 | -1.215506 |
| ENSG00000166780.10 | 1.82E-58 | 8.5743924 | 10.110441 | -1.536049 |
| ENSG00000251143.1 | 1.52E-37 | 5.8387644 | 6.9284114 | -1.089647 |
| ENSG00000237949.1 | 8.32E-41 | 0.3692652 | 4.0972148 | -3.72795 |
| ENSG00000152527.13 | 7.08E-107 | 8.1358842 | 11.509832 | -3.373948 |
| ENSG00000261438.1 | 1.18E-32 | 4.1150062 | 5.3670045 | -1.251998 |
| ENSG00000117643.14 | 2.04E-31 | 8.888827 | 10.044084 | -1.155257 |
| ENSG00000272017.1 | 7.63E-22 | 2.6981547 | 3.8753477 | -1.177193 |
| ENSG00000259884.1 | 6.89E-41 | 4.2078186 | 8.0524523 | -3.844634 |
| ENSG00000102010.14 | 2.74E-08 | 3.1914091 | 4.3080114 | -1.116602 |
| ENSG00000204681.10 | 4.34E-125 | 9.465201 | 12.40145 | -2.936249 |
| ENSG00000241362.2 | 1.29E-56 | 1.1355339 | 3.2740943 | -2.13856 |
| ENSG00000272799.1 | 2.05E-20 | 0.9741095 | 2.3916545 | -1.417545 |
| ENSG00000225465.8 | 9.31E-18 | 5.1973105 | 6.598875 | -1.401564 |
| ENSG00000236426.5 | 2.16E-20 | 0.3063976 | 1.9452068 | -1.638809 |
| ENSG00000157152.16 | 5.45E-37 | 5.3854726 | 7.0859818 | -1.700509 |
| ENSG00000226199.1 | 4.47E-25 | 0.1961141 | 1.9359591 | -1.739845 |
| ENSG00000267244.5 | 5.77E-74 | 5.6347575 | 7.2618784 | -1.627121 |
| ENSG00000214548.14 | 9.18E-136 | 9.5535298 | 16.234655 | -6.681125 |
| ENSG00000116962.14 | 6.02E-41 | 10.667572 | 12.659201 | -1.991629 |
| ENSG00000269226.7 | 1.42E-94 | 5.3590169 | 8.0238534 | -2.664836 |
| ENSG00000213931.5 | 1.57E-13 | 1.6350499 | 2.6767625 | -1.041713 |
| ENSG00000172260.13 | 1.45E-42 | 5.6278962 | 8.1258534 | -2.497957 |
| ENSG00000168830.7 | 4.08E-21 | 2.1720721 | 4.7016682 | -2.529596 |
| ENSG00000165197.4 | 8.39E-51 | 2.6926377 | 6.5736705 | -3.881033 |
| ENSG00000162415.6 | 3.04E-72 | 6.9920573 | 9.3447159 | -2.352659 |
| ENSG00000248464.1 | 4.37E-33 | 0.1897356 | 1.9817068 | -1.791971 |
| ENSG00000095397.13 | 4.21E-29 | 9.2321807 | 10.353806 | -1.121625 |
| ENSG00000235505.7 | 4.22E-49 | 5.5834706 | 7.9598523 | -2.376382 |
| ENSG00000244480.1 | 4.05E-101 | 3.6515802 | 6.120317 | -2.468737 |
| ENSG00000146678.9 | 2.95E-17 | 3.2333248 | 6.8204352 | -3.58711 |
| ENSG00000232377.1 | 4.90E-28 | 0.3262446 | 1.8915295 | -1.565285 |
| ENSG00000018189.12 | 5.86E-103 | 9.4882594 | 11.119215 | -1.630955 |
| ENSG00000280334.1 | 1.85E-101 | 3.940642 | 7.4601261 | -3.519484 |
| ENSG00000173209.22 | 9.76E-63 | 10.945102 | 12.483933 | -1.538831 |
| ENSG00000224934.2 | 6.22E-25 | 5.6801208 | 6.9840761 | -1.303955 |
| ENSG00000155970.11 | 1.34E-81 | 6.7898523 | 8.7772466 | -1.987394 |
| ENSG00000168004.9 | 1.95E-14 | 5.2450045 | 6.2651602 | -1.020156 |
| ENSG00000137802.13 | 4.47E-49 | 9.7801496 | 10.874848 | -1.094698 |
| ENSG00000239257.1 | 7.23E-60 | 2.6608174 | 5.449008 | -2.788191 |
| ENSG00000161010.14 | 2.70E-42 | 10.109469 | 11.23986 | -1.130392 |
| ENSG00000243829.1 | 9.29E-46 | 2.6904551 | 4.3171068 | -1.626652 |
| ENSG00000230606.10 | 1.02E-19 | 8.648542 | 9.7787432 | -1.130201 |
| ENSG00000267731.1 | 6.79E-49 | 3.8124141 | 5.6692591 | -1.856845 |
| ENSG00000232653.8 | 9.09E-139 | 7.4014699 | 10.767609 | -3.366139 |
| ENSG00000104691.14 | 9.15E-27 | 8.655069 | 10.184326 | -1.529257 |
| ENSG00000169047.5 | 2.00E-20 | 10.483143 | 11.498124 | -1.014981 |
| ENSG00000254527.1 | 9.28E-21 | 1.6175477 | 2.7847818 | -1.167234 |
| ENSG00000185532.14 | 2.10E-19 | 6.9120403 | 8.2169443 | -1.304904 |
| ENSG00000250107.1 | 3.13E-24 | 2.064869 | 3.5265261 | -1.461657 |
| ENSG00000280083.1 | 1.01E-35 | 0.9788203 | 3.0797216 | -2.100901 |
| ENSG00000108823.15 | 1.15E-20 | 3.0776936 | 5.4327477 | -2.355054 |
| ENSG00000204677.10 | 9.95E-62 | 3.0856728 | 5.9004284 | -2.814756 |
| ENSG00000182841.12 | 6.03E-41 | 8.9881566 | 10.422459 | -1.434303 |
| ENSG00000270972.1 | 1.22E-34 | 1.3836656 | 3.234717 | -1.851051 |
| ENSG00000158301.18 | 3.01E-101 | 7.9719031 | 10.42559 | -2.453687 |
| ENSG00000278864.1 | 6.38E-76 | 4.939284 | 6.8771227 | -1.937839 |
| ENSG00000109099.13 | 7.56E-29 | 10.410653 | 11.440894 | -1.030241 |
| ENSG00000105509.10 | 1.52E-51 | 3.4474079 | 8.4570045 | -5.009597 |
| ENSG00000264012.1 | 4.65E-25 | 0.4157418 | 1.9962489 | -1.580507 |
| ENSG00000007314.11 | 1.80E-22 | 3.8461282 | 5.1610307 | -1.314903 |
| ENSG00000145242.13 | 2.05E-51 | 1.9251415 | 5.7994159 | -3.874274 |
| ENSG00000169860.6 | 3.43E-55 | 7.0158427 | 8.7013318 | -1.685489 |
| ENSG00000070371.15 | 1.06E-43 | 7.1075248 | 8.302075 | -1.19455 |
| ENSG00000158764.6 | 1.43E-48 | 1.4425148 | 4.9361557 | -3.493641 |
| ENSG00000273018.5 | 1.26E-87 | 4.2400098 | 8.3890614 | -4.149052 |
| ENSG00000101203.16 | 2.47E-15 | 2.1086158 | 3.4082409 | -1.299625 |
| ENSG00000248159.1 | 1.04E-30 | 0.9980313 | 2.3263409 | -1.32831 |
| ENSG00000259295.6 | 1.48E-98 | 7.8999095 | 10.626533 | -2.726623 |
| ENSG00000273125.1 | 1.19E-29 | 1.8839358 | 3.4745784 | -1.590643 |
| ENSG00000156486.7 | 1.20E-29 | 3.4510628 | 5.6075693 | -2.156507 |
| ENSG00000183569.17 | 3.03E-35 | 6.9586193 | 8.6361886 | -1.677569 |
| ENSG00000143869.6 | 3.13E-11 | 3.9715888 | 5.2398795 | -1.268291 |
| ENSG00000280216.1 | 1.24E-80 | 1.47521 | 4.8402739 | -3.365064 |
| ENSG00000249451.1 | 3.66E-31 | 0.1989613 | 1.6397239 | -1.440763 |
| ENSG00000179751.6 | 2.06E-24 | 0.3516566 | 2.2673966 | -1.91574 |
| ENSG00000175518.6 | 5.13E-26 | 4.5723074 | 6.0846795 | -1.512372 |
| ENSG00000240050.5 | 1.41E-31 | 1.9656248 | 3.2272148 | -1.26159 |
| ENSG00000157343.8 | 8.59E-29 | 4.2822411 | 5.5171136 | -1.234873 |
| ENSG00000132424.14 | 2.30E-67 | 11.824266 | 13.336555 | -1.512288 |
| ENSG00000272681.2 | 7.53E-41 | 1.8231916 | 2.953825 | -1.130633 |
| ENSG00000184985.16 | 9.52E-22 | 7.385543 | 8.8003011 | -1.414758 |
| ENSG00000267281.2 | 2.13E-67 | 5.7325921 | 7.7495477 | -2.016956 |
| ENSG00000242198.1 | 2.78E-35 | 1.3093148 | 2.6631977 | -1.353883 |
| ENSG00000138738.10 | 4.29E-116 | 6.7698138 | 9.3856852 | -2.615871 |
| ENSG00000250343.1 | 4.93E-37 | 0.432263 | 2.2002966 | -1.768034 |
| ENSG00000130844.16 | 7.07E-18 | 9.6491172 | 11.576583 | -1.927466 |
| ENSG00000275120.1 | 1.23E-34 | 1.1748716 | 2.6291045 | -1.454233 |
| ENSG00000206077.10 | 2.31E-16 | 6.1541191 | 7.2721455 | -1.118026 |
| ENSG00000171189.16 | 1.94E-17 | 1.378099 | 2.663092 | -1.284993 |
| ENSG00000271474.1 | 2.80E-17 | 3.5641938 | 4.821092 | -1.256898 |
| ENSG00000219438.8 | 1.16E-22 | 6.9246473 | 8.2892511 | -1.364604 |
| ENSG00000280439.1 | 6.91E-14 | 1.4927575 | 2.5809148 | -1.088157 |
| ENSG00000277801.1 | 1.29E-55 | 7.244173 | 8.7069114 | -1.462738 |
| ENSG00000274395.1 | 3.40E-13 | 0.5585525 | 1.9586023 | -1.40005 |
| ENSG00000177103.13 | 4.30E-23 | 7.1012802 | 8.7760284 | -1.674748 |
| ENSG00000182253.14 | 5.62E-24 | 7.7273811 | 9.6814875 | -1.954106 |
| ENSG00000280191.3 | 9.34E-11 | 1.0072356 | 2.2787284 | -1.271493 |
| ENSG00000273551.1 | 5.33E-41 | 0.4791229 | 3.7604784 | -3.281355 |
| ENSG00000267707.2 | 7.79E-41 | 3.3689461 | 5.01615 | -1.647204 |
| ENSG00000205041.1 | 1.74E-40 | 4.025942 | 5.6408432 | -1.614901 |
| ENSG00000273340.1 | 7.58E-30 | 0.9167002 | 2.45725 | -1.54055 |
| ENSG00000267632.1 | 2.29E-54 | 3.4404599 | 5.8278795 | -2.38742 |
| ENSG00000091622.15 | 1.14E-32 | 9.7749289 | 11.331418 | -1.556489 |
| ENSG00000265401.1 | 2.12E-79 | 2.7499036 | 7.1294795 | -4.379576 |
| ENSG00000274818.1 | 3.73E-40 | 3.2550723 | 4.8153489 | -1.560277 |
| ENSG00000272144.1 | 9.36E-41 | 2.7501757 | 4.6319409 | -1.881765 |
| ENSG00000188828.11 | 5.32E-50 | 0.4563931 | 3.5890375 | -3.132644 |
| ENSG00000235020.4 | 1.95E-25 | 0.4207026 | 2.1321136 | -1.711411 |
| ENSG00000160801.13 | 6.71E-93 | 5.550837 | 8.8314659 | -3.280629 |
| ENSG00000144891.17 | 1.41E-27 | 4.3057012 | 6.3935489 | -2.087848 |
| ENSG00000134571.10 | 6.23E-16 | 1.800743 | 3.1908795 | -1.390137 |
| ENSG00000170500.12 | 3.22E-21 | 10.197418 | 11.979859 | -1.782441 |
| ENSG00000273082.1 | 5.80E-28 | 0.7560365 | 2.1996477 | -1.443611 |
| ENSG00000184601.10 | 1.98E-21 | 1.2415957 | 3.5792682 | -2.337672 |
| ENSG00000092051.16 | 4.15E-52 | 6.2504093 | 9.2912455 | -3.040836 |
| ENSG00000144218.18 | 9.67E-92 | 6.5143339 | 9.5094693 | -2.995135 |
| ENSG00000118762.7 | 5.58E-58 | 9.9353239 | 11.137659 | -1.202335 |
| ENSG00000272511.1 | 1.93E-12 | 1.4497189 | 2.5342386 | -1.08452 |
| ENSG00000185274.11 | 5.98E-16 | 9.3899279 | 10.633113 | -1.243185 |
| ENSG00000233024.7 | 7.29E-75 | 9.9085597 | 11.661227 | -1.752668 |
| ENSG00000228536.1 | 3.61E-23 | 1.7575406 | 3.4908102 | -1.73327 |
| ENSG00000066230.10 | 4.48E-15 | 3.1977091 | 4.2600057 | -1.062297 |
| ENSG00000272733.1 | 8.30E-52 | 2.5146093 | 5.0429852 | -2.528376 |
| ENSG00000158427.14 | 8.36E-41 | 6.4002859 | 7.812517 | -1.412231 |
| ENSG00000116194.12 | 5.21E-76 | 6.5764 | 10.647276 | -4.070876 |
| ENSG00000120658.12 | 3.58E-70 | 5.9599422 | 8.2252023 | -2.26526 |
| ENSG00000042062.11 | 3.74E-73 | 6.4635766 | 9.357033 | -2.893456 |
| ENSG00000104852.14 | 2.69E-65 | 12.644621 | 14.12304 | -1.478418 |
| ENSG00000054654.15 | 3.73E-49 | 11.694766 | 12.939545 | -1.244779 |
| ENSG00000187240.13 | 1.87E-44 | 8.8583897 | 9.8814159 | -1.023026 |
| ENSG00000071205.11 | 3.74E-119 | 8.2666379 | 10.679847 | -2.413209 |
| ENSG00000174576.8 | 2.29E-07 | 2.1061988 | 3.2449023 | -1.138703 |
| ENSG00000109063.14 | 3.68E-80 | 5.8523186 | 7.9054068 | -2.053088 |
| ENSG00000280328.1 | 8.13E-34 | 5.7190508 | 7.2146136 | -1.495563 |
| ENSG00000198300.12 | 8.90E-113 | 7.905099 | 13.980775 | -6.075676 |
| ENSG00000106991.13 | 2.63E-32 | 12.227075 | 13.349881 | -1.122806 |
| ENSG00000186399.10 | 8.99E-139 | 4.3356084 | 7.9359432 | -3.600335 |
| ENSG00000258559.2 | 1.74E-50 | 5.8706852 | 7.5063523 | -1.635667 |
| ENSG00000171747.8 | 1.36E-30 | 3.8723064 | 5.2688659 | -1.396559 |
| ENSG00000258818.3 | 2.63E-145 | 8.2446912 | 11.222791 | -2.9781 |
| ENSG00000179979.8 | 1.93E-41 | 8.5325473 | 9.7605568 | -1.22801 |
| ENSG00000278918.1 | 6.00E-28 | 3.3788716 | 5.0294159 | -1.650544 |
| ENSG00000187068.2 | 8.31E-36 | 6.1994573 | 7.6605034 | -1.461046 |
| ENSG00000278829.1 | 8.67E-29 | 0.848753 | 2.2117102 | -1.362957 |
| ENSG00000279022.1 | 2.11E-32 | 1.7898134 | 3.3495966 | -1.559783 |
| ENSG00000080293.9 | 3.91E-17 | 1.7575232 | 3.1335636 | -1.37604 |
| ENSG00000272574.1 | 7.26E-24 | 1.4755348 | 3.3999784 | -1.924444 |
| ENSG00000154485.4 | 5.54E-57 | 1.6921298 | 4.2569432 | -2.564813 |
| ENSG00000163884.3 | 7.03E-68 | 8.0704169 | 10.757322 | -2.686905 |
| ENSG00000240522.1 | 5.82E-55 | 2.6839403 | 5.5432261 | -2.859286 |
| ENSG00000141504.11 | 1.53E-46 | 10.099637 | 11.390978 | -1.291342 |
| ENSG00000185008.17 | 8.16E-09 | 6.7766687 | 7.8810727 | -1.104404 |
| ENSG00000198598.6 | 1.70E-84 | 5.8606907 | 8.9684295 | -3.107739 |
| ENSG00000169075.7 | 2.77E-18 | 0.7539988 | 1.9210023 | -1.167003 |
| ENSG00000231160.9 | 3.77E-68 | 6.5055451 | 9.6912761 | -3.185731 |
| ENSG00000259384.6 | 5.30E-13 | 1.0576952 | 3.4696341 | -2.411939 |
| ENSG00000260855.1 | 4.70E-37 | 4.8632453 | 6.4607705 | -1.597525 |
| ENSG00000018236.14 | 1.47E-46 | 8.2062117 | 10.407944 | -2.201733 |
| ENSG00000270011.6 | 5.65E-25 | 6.0814105 | 7.1980761 | -1.116666 |
| ENSG00000272367.1 | 1.14E-36 | 3.3460771 | 5.573117 | -2.22704 |
| ENSG00000261823.1 | 1.19E-18 | 0.6754995 | 1.7060591 | -1.03056 |
| ENSG00000011465.16 | 1.31E-87 | 13.119334 | 15.880819 | -2.761485 |
| ENSG00000204767.3 | 1.37E-14 | 2.9532864 | 4.3556511 | -1.402365 |
| ENSG00000188626.6 | 1.25E-141 | 1.1954945 | 6.9465795 | -5.751085 |
| ENSG00000000005.5 | 7.17E-25 | 2.441616 | 4.3431909 | -1.901575 |
| ENSG00000272812.1 | 7.52E-49 | 4.1467081 | 6.1100955 | -1.963387 |
| ENSG00000227176.1 | 3.40E-43 | 0.6358742 | 2.609533 | -1.973659 |
| ENSG00000222009.8 | 7.48E-45 | 7.9926086 | 9.3144216 | -1.321813 |
| ENSG00000236990.1 | 1.24E-17 | 0.5353496 | 1.7764852 | -1.241136 |
| ENSG00000257894.2 | 2.08E-18 | 4.8392578 | 7.046092 | -2.206834 |
| ENSG00000116741.7 | 1.83E-24 | 8.9796403 | 10.878609 | -1.898969 |
| ENSG00000147852.15 | 1.52E-81 | 9.9304654 | 12.29094 | -2.360474 |
| ENSG00000162669.15 | 4.18E-99 | 4.5791852 | 7.7950091 | -3.215824 |
| ENSG00000280453.1 | 4.26E-11 | 0.348157 | 1.5107705 | -1.162613 |
| ENSG00000268555.1 | 9.95E-19 | 3.0232241 | 4.674733 | -1.651509 |
| ENSG00000198156.10 | 1.05E-28 | 5.0239933 | 6.3341443 | -1.310151 |
| ENSG00000003096.13 | 5.09E-39 | 6.4289716 | 7.7974284 | -1.368457 |
| ENSG00000184451.5 | 1.67E-35 | 5.8763802 | 7.024608 | -1.148228 |
| ENSG00000174348.13 | 4.12E-140 | 9.0663637 | 13.148013 | -4.081649 |
| ENSG00000252355.1 | 8.26E-25 | 0.4786184 | 1.5957739 | -1.117155 |
| ENSG00000236136.1 | 7.08E-19 | 0.5037184 | 1.8507341 | -1.347016 |
| ENSG00000185053.12 | 1.58E-58 | 0.4233232 | 3.9364057 | -3.513083 |
| ENSG00000251414.1 | 4.46E-31 | 2.539374 | 3.8784352 | -1.339061 |
| ENSG00000111341.9 | 1.20E-16 | 11.304127 | 12.713809 | -1.409682 |
| ENSG00000105784.15 | 1.66E-18 | 5.3835826 | 6.404008 | -1.020425 |
| ENSG00000139874.5 | 1.52E-32 | 3.2813069 | 5.3585966 | -2.07729 |
| ENSG00000139211.6 | 3.60E-21 | 8.8670757 | 10.157732 | -1.290656 |
| ENSG00000243759.1 | 6.23E-53 | 3.3029556 | 4.8994 | -1.596444 |
| ENSG00000223704.1 | 1.18E-70 | 2.9895623 | 5.0532023 | -2.06364 |
| ENSG00000248596.6 | 4.33E-37 | 7.2265449 | 10.236953 | -3.010409 |
| ENSG00000057468.6 | 4.74E-32 | 2.9879881 | 4.4920568 | -1.504069 |
| ENSG00000146021.14 | 2.37E-51 | 8.0077322 | 9.2895648 | -1.281833 |
| ENSG00000136546.13 | 3.72E-191 | 4.8006024 | 11.851408 | -7.050806 |
| ENSG00000234456.7 | 1.60E-136 | 8.3436831 | 11.228914 | -2.885231 |
| ENSG00000142494.13 | 1.26E-25 | 6.1677012 | 7.9719239 | -1.804223 |
| ENSG00000123358.19 | 5.08E-54 | 11.458166 | 15.318206 | -3.860039 |
| ENSG00000244578.1 | 1.45E-63 | 2.2484874 | 7.5796886 | -5.331201 |
| ENSG00000254967.6 | 1.81E-12 | 0.6178678 | 1.7678455 | -1.149978 |
| ENSG00000271871.1 | 3.10E-38 | 2.5012019 | 3.7455886 | -1.244387 |
| ENSG00000250295.6 | 4.60E-14 | 2.0992239 | 3.155867 | -1.056643 |
| ENSG00000262119.1 | 4.35E-29 | 1.0921203 | 2.5808818 | -1.488762 |
| ENSG00000182326.14 | 1.90E-53 | 12.999958 | 14.901042 | -1.901084 |
| ENSG00000280551.1 | 8.10E-85 | 2.0095511 | 4.7309602 | -2.721409 |
| ENSG00000118473.21 | 1.16E-18 | 5.4149695 | 6.6493761 | -1.234407 |
| ENSG00000275155.1 | 4.32E-20 | 3.5603637 | 4.7734114 | -1.213048 |
| ENSG00000222112.1 | 4.75E-26 | 0.6952919 | 2.0394455 | -1.344154 |
| ENSG00000237863.2 | 1.81E-107 | 2.1341458 | 6.7334284 | -4.599283 |
| ENSG00000213057.5 | 3.29E-17 | 4.4481835 | 5.4725989 | -1.024415 |
| ENSG00000264727.1 | 3.73E-14 | 0.6419282 | 2.0305966 | -1.388668 |
| ENSG00000230537.1 | 6.56E-25 | 3.0676683 | 4.4098216 | -1.342153 |
| ENSG00000116667.12 | 4.28E-31 | 10.061502 | 11.085927 | -1.024426 |
| ENSG00000228748.2 | 4.99E-22 | 6.5424191 | 7.7449398 | -1.202521 |
| ENSG00000068831.18 | 6.81E-19 | 6.4524489 | 7.9867477 | -1.534299 |
| ENSG00000264112.1 | 1.30E-23 | 8.1213881 | 9.1647761 | -1.043388 |
| ENSG00000255504.2 | 1.26E-38 | 0.4691757 | 2.8517909 | -2.382615 |
| ENSG00000279631.1 | 1.11E-37 | 4.4578535 | 5.7992841 | -1.341431 |
| ENSG00000172023.7 | 3.02E-32 | 0.2842387 | 3.8057534 | -3.521515 |
| ENSG00000277999.1 | 1.90E-39 | 1.5123377 | 4.2407705 | -2.728433 |
| ENSG00000177363.4 | 1.21E-126 | 6.7953857 | 9.8021205 | -3.006735 |
| ENSG00000233264.2 | 1.57E-31 | 1.3590353 | 3.0050705 | -1.646035 |
| ENSG00000204706.14 | 3.24E-53 | 4.198743 | 5.6633307 | -1.464588 |
| ENSG00000272163.1 | 3.47E-09 | 0.9969263 | 2.1358023 | -1.138876 |
| ENSG00000279861.1 | 2.37E-56 | 5.9607384 | 7.5949057 | -1.634167 |
| ENSG00000279875.1 | 3.64E-18 | 0.7046537 | 2.0005784 | -1.295925 |
| ENSG00000083097.14 | 1.19E-74 | 9.1935492 | 10.381843 | -1.188294 |
| ENSG00000271840.1 | 1.36E-21 | 1.492668 | 2.7841341 | -1.291466 |
| ENSG00000166482.11 | 7.97E-62 | 10.239146 | 12.663038 | -2.423891 |
| ENSG00000154175.16 | 1.32E-83 | 8.5236611 | 11.112057 | -2.588396 |
| ENSG00000240180.1 | 2.83E-36 | 1.293432 | 3.862583 | -2.569151 |
| ENSG00000188487.11 | 3.60E-10 | 1.0663893 | 2.284575 | -1.218186 |
| ENSG00000236114.1 | 7.27E-36 | 2.1842263 | 3.7897159 | -1.60549 |
| ENSG00000258274.1 | 4.63E-23 | 0.8628353 | 2.8936545 | -2.030819 |
| ENSG00000267594.5 | 1.92E-15 | 0.7685119 | 3.7474159 | -2.978904 |
| ENSG00000272638.1 | 8.65E-37 | 6.0061141 | 7.4737989 | -1.467685 |
| ENSG00000214897.4 | 3.36E-12 | 3.7224093 | 5.4981068 | -1.775698 |
| ENSG00000128918.14 | 8.85E-18 | 7.7082943 | 9.345492 | -1.637198 |
| ENSG00000260645.1 | 5.71E-85 | 4.0008735 | 6.9145193 | -2.913646 |
| ENSG00000175265.17 | 1.04E-123 | 10.717875 | 13.714088 | -2.996212 |
| ENSG00000108924.13 | 4.30E-151 | 5.880926 | 11.17233 | -5.291404 |
| ENSG00000162804.13 | 4.77E-59 | 9.7049251 | 11.482595 | -1.77767 |
| ENSG00000272690.5 | 1.14E-15 | 3.5448525 | 4.6151841 | -1.070332 |
| ENSG00000275339.1 | 3.14E-21 | 0.2189599 | 1.3291023 | -1.110142 |
| ENSG00000229372.1 | 2.72E-27 | 1.9615785 | 3.6994273 | -1.737849 |
| ENSG00000269736.1 | 5.41E-22 | 1.0647396 | 2.3417489 | -1.277009 |
| ENSG00000226636.1 | 1.01E-22 | 0.6153453 | 1.7933182 | -1.177973 |
| ENSG00000108785.7 | 3.52E-44 | 2.0147062 | 3.794183 | -1.779477 |
| ENSG00000197641.11 | 1.58E-12 | 0.2417601 | 1.7055943 | -1.463834 |
| ENSG00000188778.4 | 1.01E-130 | 2.0262427 | 9.0055466 | -6.979304 |
| ENSG00000266236.1 | 1.06E-54 | 2.5854601 | 4.1621534 | -1.576693 |
| ENSG00000124302.12 | 1.25E-34 | 3.0633384 | 4.8922636 | -1.828925 |
| ENSG00000259171.1 | 3.52E-93 | 6.4548072 | 9.7414716 | -3.286664 |
| ENSG00000267199.1 | 3.22E-41 | 5.3670415 | 6.9718909 | -1.604849 |
| ENSG00000166924.8 | 7.67E-36 | 6.8333893 | 8.2175818 | -1.384193 |
| ENSG00000236404.8 | 9.17E-54 | 5.4992229 | 8.5379716 | -3.038749 |
| ENSG00000174469.17 | 1.19E-15 | 4.8306924 | 6.4563523 | -1.62566 |
| ENSG00000109501.13 | 2.24E-48 | 11.283467 | 12.385955 | -1.102487 |
| ENSG00000152785.6 | 9.79E-16 | 4.3565998 | 6.1035705 | -1.746971 |
| ENSG00000243955.5 | 1.52E-06 | 6.6352334 | 8.0360477 | -1.400814 |
| ENSG00000235790.7 | 1.59E-49 | 2.0706671 | 4.6599568 | -2.58929 |
| ENSG00000187166.1 | 2.37E-49 | 0.9347029 | 3.7909295 | -2.856227 |
| ENSG00000225670.4 | 6.29E-13 | 3.0048241 | 4.9661193 | -1.961295 |
| ENSG00000277050.1 | 7.94E-17 | 2.8087394 | 4.0312261 | -1.222487 |
| ENSG00000145808.8 | 1.50E-42 | 2.5826203 | 5.6657284 | -3.083108 |
| ENSG00000100987.14 | 3.50E-18 | 1.5648766 | 3.3718727 | -1.806996 |
| ENSG00000133169.5 | 5.50E-12 | 6.2724666 | 7.578742 | -1.306275 |
| ENSG00000100321.14 | 2.78E-83 | 9.332143 | 11.608465 | -2.276322 |
| ENSG00000002587.9 | 8.14E-49 | 8.4570055 | 10.894345 | -2.43734 |
| ENSG00000101977.19 | 2.57E-56 | 2.4388208 | 7.1112864 | -4.672466 |
| ENSG00000130558.18 | 1.80E-49 | 6.785216 | 9.0789227 | -2.293707 |
| ENSG00000254352.1 | 3.53E-34 | 0.940264 | 2.5121977 | -1.571934 |
| ENSG00000221978.11 | 8.25E-74 | 12.026042 | 13.501651 | -1.475609 |
| ENSG00000272072.1 | 4.03E-58 | 3.2722363 | 5.4750216 | -2.202785 |
| ENSG00000149294.16 | 5.16E-45 | 9.6241671 | 11.763049 | -2.138882 |
| ENSG00000151617.15 | 6.43E-41 | 8.5416021 | 10.419055 | -1.877452 |
| ENSG00000090006.17 | 3.53E-67 | 12.461633 | 14.282452 | -1.820819 |
| ENSG00000182752.9 | 2.98E-23 | 5.6776167 | 7.18865 | -1.511033 |
| ENSG00000230595.1 | 6.45E-18 | 0.0717845 | 1.0870545 | -1.01527 |
| ENSG00000130173.13 | 6.98E-27 | 1.5997499 | 2.8715966 | -1.271847 |
| ENSG00000235677.1 | 1.11E-38 | 1.9480609 | 3.6408739 | -1.692813 |
| ENSG00000268297.1 | 5.82E-40 | 0.6176134 | 4.0174239 | -3.39981 |
| ENSG00000259948.2 | 9.43E-78 | 5.4933695 | 7.8699409 | -2.376571 |
| ENSG00000233967.6 | 1.67E-67 | 3.5779611 | 5.8739273 | -2.295966 |
| ENSG00000235351.1 | 1.79E-57 | 2.7056124 | 5.3317239 | -2.626111 |
| ENSG00000197561.6 | 1.98E-08 | 0.916168 | 1.9944216 | -1.078254 |
| ENSG00000139352.3 | 3.28E-07 | 1.8676422 | 3.0239955 | -1.156353 |
| ENSG00000227678.7 | 9.24E-30 | 1.8212484 | 3.0186 | -1.197352 |
| ENSG00000276523.1 | 6.74E-59 | 2.3304998 | 4.1278773 | -1.797378 |
| ENSG00000167769.4 | 3.29E-25 | 1.0361203 | 2.5502909 | -1.514171 |
| ENSG00000186479.4 | 1.64E-20 | 4.4078086 | 6.0601341 | -1.652325 |
| ENSG00000261685.2 | 2.10E-12 | 6.5781418 | 7.6261989 | -1.048057 |
| ENSG00000229901.1 | 7.07E-29 | 1.2913079 | 2.8721057 | -1.580798 |
| ENSG00000197769.5 | 1.25E-19 | 3.1674854 | 4.6175636 | -1.450078 |
| ENSG00000256162.2 | 9.19E-26 | 0.5777487 | 2.5154227 | -1.937674 |
| ENSG00000227782.2 | 1.67E-33 | 4.2917198 | 5.734117 | -1.442397 |
| ENSG00000115665.8 | 1.07E-12 | 0.8085499 | 2.2146818 | -1.406132 |
| ENSG00000274911.1 | 1.08E-17 | 2.9607771 | 4.0443432 | -1.083566 |
| ENSG00000258734.2 | 5.06E-23 | 2.7925554 | 3.959675 | -1.16712 |
| ENSG00000224078.12 | 1.72E-30 | 10.056569 | 11.286563 | -1.229994 |
| ENSG00000102904.14 | 2.98E-31 | 6.1301365 | 7.3153432 | -1.185207 |
| ENSG00000149451.17 | 4.95E-86 | 7.8029394 | 11.798544 | -3.995605 |
| ENSG00000171004.17 | 9.89E-64 | 6.1111816 | 9.3096693 | -3.198488 |
| ENSG00000263393.1 | 5.58E-18 | 1.2483442 | 2.363775 | -1.115431 |
| ENSG00000225083.1 | 2.81E-47 | 2.1717248 | 4.3180693 | -2.146344 |
| ENSG00000234362.5 | 4.26E-36 | 2.5280294 | 4.8590023 | -2.330973 |
| ENSG00000279135.1 | 1.20E-24 | 1.7688353 | 3.1776852 | -1.40885 |
| ENSG00000281348.1 | 5.31E-12 | 3.2798375 | 4.2978023 | -1.017965 |
| ENSG00000125675.17 | 3.17E-86 | 3.551963 | 7.3173364 | -3.765373 |
| ENSG00000243175.1 | 3.88E-14 | 1.2855592 | 2.5730625 | -1.287503 |
| ENSG00000147576.15 | 1.33E-55 | 7.5384647 | 10.25207 | -2.713606 |
| ENSG00000226252.1 | 2.21E-38 | 1.1019007 | 3.5072602 | -2.40536 |
| ENSG00000259614.2 | 2.49E-58 | 0.294674 | 3.6757545 | -3.381081 |
| ENSG00000077943.7 | 3.87E-23 | 6.3679105 | 8.365025 | -1.997114 |
| ENSG00000047634.14 | 3.83E-71 | 8.5899286 | 10.880161 | -2.290233 |
| ENSG00000115361.7 | 5.09E-119 | 3.1831881 | 9.080458 | -5.89727 |
| ENSG00000278867.1 | 2.52E-56 | 4.7687282 | 6.7425034 | -1.973775 |
| ENSG00000272578.5 | 5.54E-36 | 7.2076396 | 8.2910398 | -1.0834 |
| ENSG00000141431.9 | 8.69E-108 | 4.8210425 | 8.5049955 | -3.683953 |
| ENSG00000143878.9 | 5.28E-15 | 12.56163 | 13.704211 | -1.142582 |
| ENSG00000154134.14 | 3.08E-62 | 8.8195745 | 11.39238 | -2.572805 |
| ENSG00000219404.2 | 6.32E-19 | 0.2230353 | 1.2677795 | -1.044744 |
| ENSG00000279220.1 | 1.04E-08 | 1.2813506 | 2.3700114 | -1.088661 |
| ENSG00000261087.1 | 4.30E-21 | 6.2244616 | 7.4454875 | -1.221026 |
| ENSG00000269911.1 | 8.78E-81 | 3.7770671 | 7.0201773 | -3.24311 |
| ENSG00000226800.5 | 2.04E-52 | 3.8946721 | 5.3856432 | -1.490971 |
| ENSG00000117791.15 | 5.93E-85 | 7.7007964 | 9.4229034 | -1.722107 |
| ENSG00000280302.1 | 3.62E-21 | 1.1997232 | 2.7611307 | -1.561408 |
| ENSG00000261794.1 | 1.25E-14 | 2.1570072 | 3.6141989 | -1.457192 |
| ENSG00000269106.1 | 3.65E-16 | 0.454663 | 1.7956977 | -1.341035 |
| ENSG00000165795.20 | 5.61E-124 | 11.105343 | 13.933149 | -2.827806 |
| ENSG00000274895.1 | 7.41E-33 | 0.3132909 | 2.1262682 | -1.812977 |
| ENSG00000036530.8 | 1.02E-48 | 5.5593644 | 7.0872682 | -1.527904 |
| ENSG00000163629.12 | 3.38E-60 | 9.2170769 | 11.348591 | -2.131514 |
| ENSG00000154898.15 | 1.68E-52 | 3.3284337 | 5.3270136 | -1.99858 |
| ENSG00000130612.14 | 3.76E-32 | 1.3227759 | 2.7311136 | -1.408338 |
| ENSG00000255445.1 | 1.24E-54 | 1.1843439 | 3.430575 | -2.246231 |
| ENSG00000108381.10 | 5.33E-79 | 3.187605 | 7.3098443 | -4.122239 |
| ENSG00000227212.3 | 4.20E-55 | 2.1357523 | 3.9645148 | -1.828763 |
| ENSG00000165121.10 | 4.55E-50 | 4.9558613 | 6.6251852 | -1.669324 |
| ENSG00000173597.8 | 7.80E-08 | 3.6402771 | 4.7182511 | -1.077974 |
| ENSG00000054803.3 | 1.74E-17 | 3.7047129 | 5.8362784 | -2.131566 |
| ENSG00000226010.1 | 2.41E-23 | 0.3200687 | 1.5787545 | -1.258686 |
| ENSG00000227827.3 | 4.26E-06 | 6.5195797 | 7.9131625 | -1.393583 |
| ENSG00000223731.2 | 3.01E-40 | 0.2524348 | 3.1877205 | -2.935286 |
| ENSG00000235436.10 | 1.55E-62 | 2.6241084 | 5.813833 | -3.189725 |
| ENSG00000168484.12 | 1.28E-16 | 0.4834112 | 2.315283 | -1.831872 |
| ENSG00000273521.1 | 9.74E-37 | 2.3675561 | 4.7784568 | -2.410901 |
| ENSG00000248746.5 | 4.16E-32 | 1.6924239 | 3.3819773 | -1.689553 |
| ENSG00000130653.15 | 3.52E-63 | 7.7087255 | 10.963618 | -3.254893 |
| ENSG00000273066.5 | 9.51E-36 | 6.0181203 | 8.289367 | -2.271247 |
| ENSG00000160808.9 | 4.53E-45 | 3.62641 | 5.5986955 | -1.972285 |
| ENSG00000258806.1 | 1.45E-27 | 1.1338415 | 2.7562091 | -1.622368 |
| ENSG00000240053.8 | 2.83E-62 | 8.1231866 | 9.8601909 | -1.737004 |
| ENSG00000278621.1 | 1.97E-13 | 2.7803315 | 4.2705205 | -1.490189 |
| ENSG00000114374.12 | 1.34E-26 | 0.0262112 | 1.4197239 | -1.393513 |
| ENSG00000237115.2 | 1.04E-36 | 1.4806964 | 3.6603886 | -2.179692 |
| ENSG00000110169.10 | 7.57E-32 | 4.3032735 | 5.5902864 | -1.287013 |
| ENSG00000232186.1 | 6.40E-46 | 3.3661263 | 4.8770943 | -1.510968 |
| ENSG00000122786.19 | 1.23E-36 | 13.122473 | 14.215201 | -1.092729 |
| ENSG00000196730.12 | 4.86E-25 | 10.612153 | 11.909616 | -1.297463 |
| ENSG00000233295.3 | 2.54E-23 | 0.9324797 | 2.4176159 | -1.485136 |
| ENSG00000164309.14 | 5.51E-95 | 7.4230119 | 12.072675 | -4.649663 |
| ENSG00000197776.7 | 7.32E-80 | 6.8771389 | 8.606325 | -1.729186 |
| ENSG00000215837.7 | 4.12E-23 | 0.8407726 | 3.2632375 | -2.422465 |
| ENSG00000215914.4 | 2.07E-10 | 7.5222406 | 8.5833739 | -1.061133 |
| ENSG00000272240.1 | 7.03E-93 | 3.2756508 | 6.0751205 | -2.79947 |
| ENSG00000253330.1 | 8.31E-11 | 3.8748107 | 4.943875 | -1.069064 |
| ENSG00000280200.1 | 1.40E-34 | 0.3450666 | 3.3533307 | -3.008264 |
| ENSG00000120833.13 | 2.78E-31 | 7.1545091 | 8.8455886 | -1.69108 |
| ENSG00000260599.1 | 1.74E-19 | 1.2210635 | 2.43985 | -1.218787 |
| ENSG00000037965.5 | 1.64E-17 | 5.1803647 | 6.6029511 | -1.422586 |
| ENSG00000186310.9 | 2.03E-33 | 6.6390155 | 8.028392 | -1.389377 |
| ENSG00000259158.2 | 5.81E-44 | 3.2409384 | 4.8066477 | -1.565709 |
| ENSG00000236085.1 | 3.19E-12 | 2.1432234 | 3.3028852 | -1.159662 |
| ENSG00000144935.14 | 2.27E-116 | 7.6505663 | 9.9310432 | -2.280477 |
| ENSG00000278448.1 | 4.27E-26 | 0.4742325 | 1.669958 | -1.195725 |
| ENSG00000227078.1 | 1.88E-15 | 0.4538382 | 1.4609739 | -1.007136 |
| ENSG00000134376.14 | 1.69E-17 | 3.0532709 | 4.468717 | -1.415446 |
| ENSG00000146001.5 | 6.63E-48 | 4.2829525 | 5.7644386 | -1.481486 |
| ENSG00000164778.4 | 2.67E-25 | 2.8561146 | 4.756225 | -1.90011 |
| ENSG00000119608.12 | 4.07E-57 | 3.3883618 | 5.5908102 | -2.202448 |
| ENSG00000220842.6 | 2.23E-37 | 12.063237 | 13.195069 | -1.131832 |
| ENSG00000102802.9 | 2.22E-09 | 7.0575258 | 8.1299602 | -1.072434 |
| ENSG00000248677.1 | 1.39E-73 | 1.9476914 | 5.016942 | -3.069251 |
| ENSG00000272541.1 | 3.97E-47 | 1.565301 | 4.2373795 | -2.672079 |
| ENSG00000271730.1 | 7.92E-42 | 3.0149652 | 5.1928659 | -2.177901 |
| ENSG00000150394.13 | 3.85E-07 | 3.7850057 | 4.8013773 | -1.016372 |
| ENSG00000256338.2 | 2.88E-114 | 5.1078062 | 7.9467727 | -2.838967 |
| ENSG00000173114.12 | 2.67E-28 | 4.6488143 | 6.4137114 | -1.764897 |
| ENSG00000182397.14 | 3.01E-53 | 2.151032 | 4.8268295 | -2.675798 |
| ENSG00000162630.5 | 1.44E-37 | 4.2937907 | 6.9785955 | -2.684805 |
| ENSG00000232710.1 | 5.84E-21 | 1.4099174 | 2.5208534 | -1.110936 |
| ENSG00000260082.1 | 1.21E-42 | 1.3921909 | 3.4258034 | -2.033612 |
| ENSG00000214016.3 | 4.14E-13 | 2.3341122 | 3.792517 | -1.458405 |
| ENSG00000171557.16 | 1.67E-27 | 1.1960611 | 4.3567466 | -3.160685 |
| ENSG00000152583.12 | 4.88E-34 | 11.653822 | 13.404149 | -1.750327 |
| ENSG00000110245.11 | 4.59E-13 | 0.5154317 | 1.7459989 | -1.230567 |
| ENSG00000071991.8 | 3.08E-31 | 1.4201761 | 5.4946489 | -4.074473 |
| ENSG00000144847.12 | 1.41E-35 | 6.7848315 | 8.8438205 | -2.058989 |
| ENSG00000266677.1 | 9.23E-33 | 2.3101451 | 3.8738682 | -1.563723 |
| ENSG00000122304.10 | 3.42E-20 | 0.2601348 | 2.5146386 | -2.254504 |
| ENSG00000238273.3 | 6.95E-60 | 3.4981644 | 7.8438841 | -4.34572 |
| ENSG00000115380.18 | 1.54E-78 | 9.8848606 | 12.658586 | -2.773726 |
| ENSG00000099937.10 | 1.82E-26 | 2.3067487 | 4.8627443 | -2.555996 |
| ENSG00000163728.10 | 3.05E-76 | 9.9560838 | 11.77355 | -1.817466 |
| ENSG00000267680.5 | 9.62E-53 | 9.0282711 | 10.041315 | -1.013044 |
| ENSG00000171551.11 | 3.35E-44 | 7.2806181 | 11.3666 | -4.085982 |
| ENSG00000196440.11 | 8.79E-133 | 8.1292518 | 11.100268 | -2.971016 |
| ENSG00000253200.1 | 5.61E-46 | 6.5625272 | 7.6535545 | -1.091027 |
| ENSG00000267436.1 | 7.74E-22 | 0.7410305 | 3.805017 | -3.063986 |
| ENSG00000238099.2 | 5.74E-25 | 1.4270876 | 3.1011091 | -1.674022 |
| ENSG00000240338.5 | 1.90E-12 | 1.2866754 | 2.4150182 | -1.128343 |
| ENSG00000124104.18 | 6.90E-38 | 9.6800446 | 10.824493 | -1.144449 |
| ENSG00000250579.1 | 6.41E-60 | 1.1546422 | 7.0210227 | -5.86638 |
| ENSG00000261126.7 | 2.51E-20 | 5.4163714 | 6.4315875 | -1.015216 |
| ENSG00000271533.1 | 2.60E-70 | 7.2187668 | 8.8757648 | -1.656998 |
| ENSG00000186642.15 | 7.75E-27 | 7.1291759 | 8.8376761 | -1.7085 |
| ENSG00000259780.3 | 2.57E-11 | 1.9971239 | 3.3341875 | -1.337064 |
| ENSG00000166086.12 | 3.52E-115 | 9.0860618 | 11.451588 | -2.365526 |
| ENSG00000114857.17 | 1.33E-117 | 10.998541 | 12.871361 | -1.872821 |
| ENSG00000259744.1 | 1.84E-13 | 1.265805 | 2.57085 | -1.305045 |
| ENSG00000232909.1 | 3.14E-20 | 0.7289444 | 1.8153784 | -1.086434 |
| ENSG00000243323.5 | 4.25E-42 | 3.4669907 | 5.9238557 | -2.456865 |
| ENSG00000172016.15 | 6.13E-17 | 0.5137444 | 2.3487114 | -1.834967 |
| ENSG00000112773.15 | 2.00E-23 | 9.7971919 | 10.85898 | -1.061788 |
| ENSG00000127083.7 | 1.37E-58 | 5.2724678 | 8.4935068 | -3.221039 |
| ENSG00000198795.10 | 1.56E-25 | 8.5208859 | 9.7129955 | -1.19211 |
| ENSG00000263968.2 | 1.26E-25 | 1.1059191 | 2.4543148 | -1.348396 |
| ENSG00000123119.11 | 2.21E-45 | 6.4769105 | 8.0400375 | -1.563127 |
| ENSG00000204172.11 | 6.92E-32 | 8.067306 | 9.3524818 | -1.285176 |
| ENSG00000154065.16 | 1.12E-160 | 5.9681086 | 9.1668443 | -3.198736 |
| ENSG00000260776.5 | 3.14E-09 | 1.9281205 | 3.1632216 | -1.235101 |
| ENSG00000246792.2 | 6.84E-52 | 2.4476783 | 4.5895295 | -2.141851 |
| ENSG00000204311.11 | 1.33E-34 | 5.915189 | 7.0233068 | -1.108118 |
| ENSG00000080546.13 | 9.84E-46 | 9.5003332 | 11.01595 | -1.515617 |
| ENSG00000101049.14 | 4.35E-22 | 6.2330971 | 7.4318932 | -1.198796 |
| ENSG00000267645.5 | 1.60E-59 | 3.7006115 | 8.4771898 | -4.776578 |
| ENSG00000235904.1 | 4.76E-21 | 1.9265241 | 3.0250114 | -1.098487 |
| ENSG00000198633.10 | 5.71E-16 | 1.5056434 | 2.7796818 | -1.274038 |
| ENSG00000279434.1 | 2.23E-54 | 4.0770081 | 5.8820875 | -1.805079 |
| ENSG00000256612.7 | 2.47E-16 | 4.4084601 | 5.5177886 | -1.109328 |
| ENSG00000184313.19 | 2.05E-47 | 7.2127933 | 9.3251398 | -2.112346 |
| ENSG00000259359.1 | 6.26E-55 | 0.9899267 | 3.308083 | -2.318156 |
| ENSG00000279713.1 | 2.37E-42 | 4.8929308 | 6.5063011 | -1.61337 |
| ENSG00000183287.13 | 1.39E-78 | 5.3797819 | 10.36724 | -4.987458 |
| ENSG00000185641.6 | 3.27E-31 | 7.2355754 | 8.4289864 | -1.193411 |
| ENSG00000282798.1 | 1.53E-28 | 2.9161943 | 4.2387591 | -1.322565 |
| ENSG00000239884.3 | 4.89E-35 | 1.7482819 | 3.3272307 | -1.578949 |
| ENSG00000279162.1 | 1.38E-62 | 2.1544172 | 3.9705534 | -1.816136 |
| ENSG00000122194.18 | 6.26E-52 | 1.7686854 | 4.2515727 | -2.482887 |
| ENSG00000171564.11 | 2.14E-25 | 0.2674606 | 3.2243409 | -2.95688 |
| ENSG00000267459.1 | 4.87E-15 | 3.1803303 | 4.6940898 | -1.513759 |
| ENSG00000263990.1 | 1.43E-36 | 2.9950697 | 4.1768261 | -1.181756 |
| ENSG00000231861.2 | 1.67E-42 | 0.4219325 | 2.7106114 | -2.288679 |
| ENSG00000006606.8 | 6.09E-45 | 2.8715501 | 6.4352045 | -3.563654 |
| ENSG00000127472.10 | 1.26E-76 | 4.8204912 | 10.511236 | -5.690745 |
| ENSG00000103489.11 | 1.32E-46 | 7.4194274 | 9.1872705 | -1.767843 |
| ENSG00000261101.2 | 3.01E-30 | 2.0121516 | 3.4582364 | -1.446085 |
| ENSG00000258504.2 | 1.31E-24 | 1.603306 | 4.3455977 | -2.742292 |
| ENSG00000279581.1 | 5.97E-23 | 0.0982881 | 1.6699205 | -1.571632 |
| ENSG00000270012.1 | 4.20E-26 | 6.5054675 | 7.6238625 | -1.118395 |
| ENSG00000178828.6 | 1.04E-19 | 0.6636699 | 2.1938602 | -1.53019 |
| ENSG00000280057.1 | 1.45E-31 | 2.8845017 | 4.4378034 | -1.553302 |
| ENSG00000280119.1 | 1.70E-76 | 2.6880926 | 7.9379727 | -5.24988 |
| ENSG00000266369.1 | 1.92E-16 | 0.7774473 | 2.2652307 | -1.487783 |
| ENSG00000260403.1 | 5.77E-37 | 0.3968189 | 1.907942 | -1.511123 |
| ENSG00000113248.5 | 2.47E-74 | 6.6938026 | 8.9214977 | -2.227695 |
| ENSG00000228363.2 | 7.83E-24 | 2.7888749 | 4.7886761 | -1.999801 |
| ENSG00000171444.17 | 8.31E-65 | 9.2245773 | 12.557914 | -3.333336 |
| ENSG00000270923.1 | 3.18E-54 | 2.6195253 | 4.3355045 | -1.715979 |
| ENSG00000164128.6 | 4.63E-07 | 4.6772578 | 5.7226295 | -1.045372 |
| ENSG00000112139.14 | 6.29E-67 | 7.4436513 | 9.5720557 | -2.128404 |
| ENSG00000016490.15 | 9.13E-25 | 0.1428828 | 1.6669432 | -1.52406 |
| ENSG00000196911.9 | 1.75E-55 | 8.6136733 | 9.8377989 | -1.224126 |
| ENSG00000186466.5 | 2.90E-10 | 0.5493136 | 1.9249693 | -1.375656 |
| ENSG00000163285.7 | 1.25E-35 | 2.3557224 | 5.6900136 | -3.334291 |
| ENSG00000230928.1 | 1.23E-50 | 1.9942017 | 3.9317 | -1.937498 |
| ENSG00000203527.2 | 6.96E-14 | 0.3538117 | 1.4638443 | -1.110033 |
| ENSG00000139547.7 | 1.30E-22 | 4.1512644 | 5.1628784 | -1.011614 |
| ENSG00000171798.17 | 5.57E-89 | 5.5364757 | 9.7335852 | -4.19711 |
| ENSG00000272589.1 | 1.72E-60 | 3.8864045 | 6.5897784 | -2.703374 |
| ENSG00000225914.1 | 3.98E-23 | 2.5761754 | 3.5817023 | -1.005527 |
| ENSG00000240751.1 | 7.41E-26 | 1.4079878 | 2.6421205 | -1.234133 |
| ENSG00000159307.18 | 1.52E-77 | 5.7194062 | 11.424202 | -5.704796 |
| ENSG00000114757.18 | 1.77E-35 | 3.2925026 | 5.0732693 | -1.780767 |
| ENSG00000128872.9 | 7.70E-50 | 7.9931704 | 9.3898341 | -1.396664 |
| ENSG00000198075.9 | 7.19E-41 | 6.4767005 | 8.6073443 | -2.130644 |
| ENSG00000270020.1 | 9.63E-22 | 2.4394981 | 3.8500455 | -1.410547 |
| ENSG00000084734.8 | 2.06E-100 | 3.0020375 | 8.0758909 | -5.073853 |
| ENSG00000280219.1 | 3.85E-27 | 5.4066592 | 6.905933 | -1.499274 |
| ENSG00000250739.1 | 3.02E-31 | 0.1361217 | 2.2826727 | -2.146551 |
| ENSG00000242697.2 | 2.86E-40 | 1.4187186 | 2.9082443 | -1.489526 |
| ENSG00000267769.1 | 5.12E-59 | 2.5019661 | 4.7362795 | -2.234313 |
| ENSG00000239959.1 | 5.16E-41 | 1.6541742 | 3.6903352 | -2.036161 |
| ENSG00000226318.1 | 9.71E-26 | 2.1186329 | 3.6691227 | -1.55049 |
| ENSG00000137392.9 | 9.43E-21 | 1.6964967 | 4.2120557 | -2.515559 |
| ENSG00000173918.14 | 1.29E-18 | 10.057214 | 11.148795 | -1.091582 |
| ENSG00000147408.14 | 3.32E-49 | 7.6134878 | 10.040177 | -2.426689 |
| ENSG00000233627.2 | 7.56E-36 | 4.9463962 | 7.5294966 | -2.5831 |
| ENSG00000242797.3 | 3.85E-21 | 2.4491136 | 4.223975 | -1.774861 |
| ENSG00000168405.14 | 5.51E-44 | 7.2121379 | 8.9834205 | -1.771283 |
| ENSG00000135473.14 | 2.07E-39 | 10.914003 | 11.971023 | -1.05702 |
| ENSG00000264573.2 | 9.41E-23 | 0.4942852 | 1.8198386 | -1.325553 |
| ENSG00000198758.10 | 3.10E-35 | 0.822358 | 2.7313114 | -1.908953 |
| ENSG00000118515.11 | 1.49E-22 | 11.451117 | 13.06766 | -1.616543 |
| ENSG00000235438.7 | 9.92E-18 | 0.8963527 | 2.0163432 | -1.11999 |
| ENSG00000262223.6 | 4.42E-23 | 0.2801501 | 1.5235239 | -1.243374 |
| ENSG00000173068.17 | 3.59E-79 | 9.5989126 | 11.749483 | -2.15057 |
| ENSG00000215483.8 | 1.34E-60 | 1.3113945 | 3.3555875 | -2.044193 |
| ENSG00000233858.4 | 1.93E-35 | 1.0712313 | 2.9760273 | -1.904796 |
| ENSG00000255328.1 | 3.26E-40 | 1.8167721 | 3.6317386 | -1.814967 |
| ENSG00000223756.6 | 2.17E-40 | 5.5694766 | 7.5869398 | -2.017463 |
| ENSG00000266200.6 | 4.42E-29 | 0.4661905 | 2.7168761 | -2.250686 |
| ENSG00000243675.1 | 8.20E-25 | 0.4351489 | 2.0781648 | -1.643016 |
| ENSG00000171408.13 | 3.00E-77 | 6.9964625 | 9.6632091 | -2.666747 |
| ENSG00000138395.14 | 2.67E-39 | 4.4841585 | 7.7163386 | -3.23218 |
| ENSG00000091513.14 | 5.59E-33 | 4.8686697 | 6.3590955 | -1.490426 |
| ENSG00000222005.8 | 1.29E-22 | 1.2570704 | 2.3421375 | -1.085067 |
| ENSG00000178394.4 | 1.38E-17 | 0.3171317 | 2.003783 | -1.686651 |
| ENSG00000107186.16 | 2.24E-80 | 9.524579 | 11.043398 | -1.518819 |
| ENSG00000153823.18 | 3.03E-125 | 6.5169401 | 9.8306239 | -3.313684 |
| ENSG00000184465.15 | 1.75E-52 | 8.7700506 | 10.235722 | -1.465671 |
| ENSG00000143520.6 | 8.71E-63 | 0.9253668 | 4.1882136 | -3.262847 |
| ENSG00000134042.12 | 1.10E-19 | 5.2275957 | 6.7638261 | -1.53623 |
| ENSG00000205562.2 | 1.31E-59 | 2.2831878 | 4.9655989 | -2.682411 |
| ENSG00000267560.1 | 2.09E-35 | 0.6269263 | 2.4633841 | -1.836458 |
| ENSG00000228695.9 | 7.76E-12 | 0.8375499 | 2.4821875 | -1.644638 |
| ENSG00000131002.11 | 8.39E-30 | 0.0287239 | 1.5223 | -1.493576 |
| ENSG00000109625.18 | 1.17E-49 | 9.936899 | 12.263281 | -2.326382 |
| ENSG00000125355.15 | 1.84E-55 | 6.8972766 | 9.0727068 | -2.17543 |
| ENSG00000163126.14 | 6.73E-58 | 6.3459153 | 7.7812034 | -1.435288 |
| ENSG00000227954.6 | 2.00E-52 | 5.7057601 | 8.4582091 | -2.752449 |
| ENSG00000231956.1 | 2.96E-26 | 0.119836 | 1.2235966 | -1.103761 |
| ENSG00000188779.10 | 6.94E-33 | 4.3186396 | 5.6616591 | -1.343019 |
| ENSG00000255986.6 | 3.22E-07 | 0.7590072 | 1.8459807 | -1.086974 |
| ENSG00000163016.9 | 1.28E-39 | 3.2771399 | 5.09785 | -1.82071 |
| ENSG00000226396.1 | 1.77E-28 | 3.9012924 | 5.6490159 | -1.747724 |
| ENSG00000248445.5 | 1.60E-42 | 3.9434138 | 5.8992807 | -1.955867 |
| ENSG00000215386.10 | 1.14E-37 | 7.8134377 | 9.0514193 | -1.237982 |
| ENSG00000232144.1 | 2.86E-24 | 0.1337136 | 1.5144795 | -1.380766 |
| ENSG00000280378.1 | 4.05E-21 | 4.5469172 | 5.8562216 | -1.309304 |
| ENSG00000263874.1 | 8.11E-44 | 4.4628881 | 6.2172523 | -1.754364 |
| ENSG00000249839.1 | 1.81E-05 | 2.9678792 | 4.1270261 | -1.159147 |
| ENSG00000255036.5 | 3.65E-39 | 6.6259165 | 7.7217841 | -1.095868 |
| ENSG00000007392.16 | 4.87E-42 | 10.455256 | 11.520948 | -1.065692 |
| ENSG00000189058.8 | 9.50E-32 | 8.6591418 | 10.500205 | -1.841063 |
| ENSG00000092445.11 | 8.66E-48 | 9.7898718 | 11.917717 | -2.127845 |
| ENSG00000106804.7 | 9.92E-54 | 6.8360604 | 8.179208 | -1.343148 |
| ENSG00000102362.15 | 3.19E-121 | 8.1478847 | 12.249026 | -4.101141 |
| ENSG00000142615.7 | 2.02E-27 | 0.640884 | 3.6130227 | -2.972139 |
| ENSG00000273599.1 | 5.38E-43 | 6.8301289 | 8.1662432 | -1.336114 |
| ENSG00000272631.1 | 1.44E-24 | 6.410285 | 7.4972318 | -1.086947 |
| ENSG00000259905.5 | 1.11E-43 | 1.0563387 | 6.0207216 | -4.964383 |
| ENSG00000282458.1 | 1.29E-33 | 10.242252 | 11.308827 | -1.066575 |
| ENSG00000166762.16 | 7.50E-21 | 7.1780277 | 8.3328239 | -1.154796 |
| ENSG00000183783.6 | 4.37E-67 | 1.5405229 | 5.7274193 | -4.186896 |
| ENSG00000198523.5 | 1.40E-22 | 4.9981377 | 7.7509898 | -2.752852 |
| ENSG00000173376.13 | 1.34E-74 | 5.456784 | 9.4828875 | -4.026103 |
| ENSG00000203877.7 | 6.48E-14 | 0.6577773 | 1.6713 | -1.013523 |
| ENSG00000203867.7 | 7.23E-79 | 5.1378866 | 8.3424545 | -3.204568 |
| ENSG00000278530.4 | 6.89E-13 | 5.4964568 | 6.6058977 | -1.109441 |
| ENSG00000182310.12 | 2.99E-31 | 8.3881215 | 9.4341227 | -1.046001 |
| ENSG00000274767.1 | 1.88E-13 | 2.6723566 | 3.9170205 | -1.244664 |
| ENSG00000235245.1 | 6.70E-39 | 4.0439511 | 5.16125 | -1.117299 |
| ENSG00000272321.1 | 3.79E-34 | 1.3752807 | 3.9736045 | -2.598324 |
| ENSG00000258727.1 | 1.09E-64 | 8.1790229 | 9.7288045 | -1.549782 |
| ENSG00000261812.5 | 8.87E-20 | 2.9717076 | 4.515842 | -1.544134 |
| ENSG00000254855.1 | 3.02E-68 | 1.8493974 | 5.4296761 | -3.580279 |
| ENSG00000229776.1 | 7.56E-36 | 4.9463962 | 7.5294966 | -2.5831 |
| ENSG00000197757.7 | 3.00E-32 | 7.5147186 | 9.2985955 | -1.783877 |
| ENSG00000268654.1 | 3.33E-64 | 1.438832 | 5.2330864 | -3.794254 |
| ENSG00000111452.12 | 1.43E-77 | 6.3037076 | 10.112049 | -3.808341 |
| ENSG00000166917.8 | 2.57E-81 | 2.0711353 | 9.3220773 | -7.250942 |
| ENSG00000112964.13 | 2.19E-34 | 6.6849757 | 8.0901659 | -1.40519 |
| ENSG00000167766.18 | 5.99E-41 | 10.879777 | 12.330159 | -1.450382 |
| ENSG00000231646.5 | 2.01E-32 | 1.865799 | 3.2260955 | -1.360296 |
| ENSG00000003436.14 | 5.79E-39 | 7.7642021 | 9.4293295 | -1.665127 |
| ENSG00000164107.8 | 1.94E-69 | 5.9641017 | 10.109609 | -4.145507 |
| ENSG00000248441.6 | 1.14E-121 | 5.0847379 | 8.5750239 | -3.490286 |
| ENSG00000163644.14 | 3.68E-86 | 9.2821621 | 11.219523 | -1.937361 |
| ENSG00000274471.1 | 4.16E-20 | 4.6683193 | 6.1965432 | -1.528224 |
| ENSG00000279465.1 | 9.16E-19 | 0.9621811 | 2.1250068 | -1.162826 |
| ENSG00000233355.6 | 3.89E-28 | 2.1203532 | 4.8871534 | -2.7668 |
| ENSG00000182836.9 | 3.47E-133 | 5.7382246 | 11.179539 | -5.441314 |
| ENSG00000213240.8 | 5.27E-13 | 0.925927 | 2.7169205 | -1.790993 |
| ENSG00000228446.2 | 2.98E-15 | 2.2072792 | 3.384158 | -1.176879 |
| ENSG00000244119.1 | 5.21E-24 | 4.0723501 | 5.7374477 | -1.665098 |
| ENSG00000133124.11 | 2.26E-121 | 1.9565621 | 6.397358 | -4.440796 |
| ENSG00000238018.2 | 1.08E-69 | 3.4249936 | 5.5726125 | -2.147619 |
| ENSG00000084674.13 | 5.49E-37 | 2.3978074 | 4.7022682 | -2.304461 |
| ENSG00000127920.5 | 1.05E-82 | 9.2514484 | 11.315963 | -2.064514 |
| ENSG00000180769.8 | 1.18E-36 | 6.4292847 | 7.645583 | -1.216298 |
| ENSG00000232372.1 | 1.02E-25 | 0.5773021 | 2.1520818 | -1.57478 |
| ENSG00000168477.17 | 5.54E-104 | 9.4778776 | 13.34211 | -3.864233 |
| ENSG00000242607.1 | 6.29E-22 | 2.8270401 | 4.1377193 | -1.310679 |
| ENSG00000271723.5 | 7.02E-45 | 5.228716 | 7.6536852 | -2.424969 |
| ENSG00000279330.1 | 1.05E-19 | 3.7052229 | 4.8401261 | -1.134903 |
| ENSG00000237560.5 | 6.73E-24 | 0.3894642 | 2.9224977 | -2.533034 |
| ENSG00000258024.1 | 5.86E-28 | 0.6618675 | 2.2126943 | -1.550827 |
| ENSG00000129167.9 | 3.39E-45 | 4.016759 | 5.8739443 | -1.857185 |
| ENSG00000261672.1 | 1.03E-49 | 0.4085456 | 3.5895591 | -3.181014 |
| ENSG00000135111.14 | 5.54E-66 | 8.3164735 | 11.446943 | -3.13047 |
| ENSG00000182575.7 | 1.62E-51 | 6.0696971 | 8.4708057 | -2.401109 |
| ENSG00000234793.1 | 4.59E-08 | 0.1100294 | 1.4007864 | -1.290757 |
| ENSG00000265489.1 | 1.19E-35 | 1.2237263 | 4.0907545 | -2.867028 |
| ENSG00000188388.10 | 9.43E-78 | 1.2922422 | 5.4703898 | -4.178148 |
| ENSG00000197594.11 | 1.36E-28 | 8.5135415 | 9.8808432 | -1.367302 |
| ENSG00000251435.1 | 2.44E-17 | 0.1786387 | 1.262125 | -1.083486 |
| ENSG00000167081.16 | 4.92E-88 | 9.3027081 | 11.279378 | -1.97667 |
| ENSG00000241769.7 | 2.15E-57 | 7.1823136 | 8.9805489 | -1.798235 |
| ENSG00000259709.1 | 3.13E-36 | 1.2724081 | 3.0979205 | -1.825512 |
| ENSG00000147162.13 | 5.49E-44 | 12.702887 | 13.705036 | -1.002149 |
| ENSG00000104936.17 | 3.78E-37 | 10.380757 | 11.499476 | -1.118719 |
| ENSG00000179270.6 | 4.18E-25 | 1.8664475 | 4.2322284 | -2.365781 |
| ENSG00000099957.16 | 8.58E-39 | 3.7708797 | 5.4032602 | -1.632381 |
| ENSG00000053702.14 | 2.17E-34 | 7.0704382 | 8.1612614 | -1.090823 |
| ENSG00000084764.10 | 1.80E-43 | 9.4436315 | 10.626422 | -1.18279 |
| ENSG00000271334.5 | 7.77E-22 | 1.0724461 | 2.1785011 | -1.106055 |
| ENSG00000144908.13 | 1.48E-68 | 6.4584115 | 10.912241 | -4.453829 |
| ENSG00000144026.11 | 9.03E-48 | 9.1019544 | 10.628445 | -1.526491 |
| ENSG00000250286.1 | 4.25E-42 | 1.38139 | 3.1526114 | -1.771221 |
| ENSG00000154736.5 | 3.55E-61 | 7.9278792 | 10.434788 | -2.506908 |
| ENSG00000131018.22 | 3.30E-115 | 9.3905573 | 13.192228 | -3.801671 |
| ENSG00000268975.1 | 1.33E-18 | 0.6624043 | 2.6611159 | -1.998712 |
| ENSG00000237580.1 | 1.22E-12 | 0.8619833 | 2.0295932 | -1.16761 |
| ENSG00000180481.10 | 1.73E-13 | 5.1699282 | 6.2138341 | -1.043906 |
| ENSG00000146250.6 | 1.34E-47 | 4.5875914 | 7.3191955 | -2.731604 |
| ENSG00000204186.7 | 7.70E-64 | 6.9553241 | 10.250332 | -3.295008 |
| ENSG00000244738.1 | 2.17E-44 | 0.2713038 | 2.654292 | -2.382988 |
| ENSG00000128573.22 | 5.67E-113 | 3.9461642 | 8.5855318 | -4.639368 |
| ENSG00000102349.14 | 4.95E-36 | 8.55979 | 9.6376239 | -1.077834 |
| ENSG00000197406.7 | 2.05E-95 | 3.979032 | 8.76 | -4.780968 |
| ENSG00000279645.1 | 1.72E-61 | 1.4101308 | 4.4375318 | -3.027401 |
| ENSG00000253123.3 | 1.81E-16 | 3.7843203 | 5.8799159 | -2.095596 |
| ENSG00000240038.6 | 1.07E-61 | 8.6481093 | 10.608759 | -1.96065 |
| ENSG00000111404.6 | 1.21E-38 | 2.2815613 | 6.779092 | -4.497531 |
| ENSG00000167779.7 | 7.76E-45 | 8.8440776 | 11.497941 | -2.653863 |
| ENSG00000105852.10 | 2.01E-100 | 4.4623604 | 8.7684693 | -4.306109 |
| ENSG00000265413.1 | 4.14E-82 | 2.9393007 | 5.3359534 | -2.396653 |
| ENSG00000226031.5 | 1.34E-21 | 1.6783964 | 2.8123455 | -1.133949 |
| ENSG00000254665.1 | 6.99E-05 | 5.9030043 | 7.0109284 | -1.107924 |
| ENSG00000277449.1 | 8.37E-39 | 5.699557 | 7.3890591 | -1.689502 |
| ENSG00000227644.2 | 2.78E-76 | 0.5608203 | 3.6862864 | -3.125466 |
| ENSG00000254681.6 | 3.45E-12 | 8.1283869 | 9.5440602 | -1.415673 |
| ENSG00000246451.2 | 7.66E-40 | 5.7686394 | 7.0788852 | -1.310246 |
| ENSG00000102271.13 | 2.88E-47 | 5.5948986 | 8.090133 | -2.495234 |
| ENSG00000239677.6 | 3.92E-49 | 0.7085036 | 3.6338034 | -2.9253 |
| ENSG00000235602.5 | 9.42E-68 | 2.5981162 | 4.9657545 | -2.367638 |
| ENSG00000280486.1 | 5.81E-26 | 1.3877759 | 2.8325614 | -1.444785 |
| ENSG00000280332.1 | 8.94E-33 | 5.9907766 | 7.0893216 | -1.098545 |
| ENSG00000164920.9 | 5.94E-55 | 8.6283115 | 11.18025 | -2.551939 |
| ENSG00000072133.10 | 2.87E-26 | 7.0460675 | 8.1571205 | -1.111053 |
| ENSG00000003989.16 | 1.69E-58 | 9.711073 | 13.064506 | -3.353433 |
| ENSG00000262877.4 | 1.65E-132 | 2.7423955 | 8.4989239 | -5.756528 |
| ENSG00000243696.4 | 3.13E-28 | 6.760515 | 7.9674386 | -1.206924 |
| ENSG00000196159.11 | 9.25E-49 | 7.5775924 | 9.3542057 | -1.776613 |
| ENSG00000137960.5 | 4.60E-25 | 5.2781735 | 6.4607045 | -1.182531 |
| ENSG00000248300.1 | 1.10E-24 | 0.1052453 | 2.2716864 | -2.166441 |
| ENSG00000137463.4 | 1.96E-62 | 5.5716418 | 8.1164341 | -2.544792 |
| ENSG00000274654.1 | 2.27E-39 | 0.6173842 | 2.895475 | -2.278091 |
| ENSG00000137558.7 | 1.92E-28 | 5.7079959 | 8.7518534 | -3.043857 |
| ENSG00000179136.6 | 4.62E-16 | 0.4869797 | 1.9249216 | -1.437942 |
| ENSG00000142789.19 | 1.86E-36 | 0.4738172 | 4.839892 | -4.366075 |
| ENSG00000145757.15 | 7.91E-57 | 2.5652017 | 4.4414364 | -1.876235 |
| ENSG00000270871.1 | 1.64E-32 | 2.5425449 | 3.8419511 | -1.299406 |
| ENSG00000225873.1 | 1.91E-12 | 1.1986632 | 2.4591955 | -1.260532 |
| ENSG00000278989.1 | 2.04E-62 | 4.8044045 | 6.7929977 | -1.988593 |
| ENSG00000128159.11 | 7.47E-98 | 10.230103 | 11.789449 | -1.559346 |
| ENSG00000234224.2 | 5.05E-16 | 0.9075442 | 2.0919216 | -1.184377 |
| ENSG00000248144.5 | 6.26E-43 | 3.5519544 | 6.3710886 | -2.819134 |
| ENSG00000233115.4 | 4.29E-17 | 1.0622499 | 2.1968125 | -1.134563 |
| ENSG00000281831.1 | 2.04E-12 | 1.4728212 | 2.8286341 | -1.355813 |
| ENSG00000229052.2 | 1.40E-21 | 1.4255592 | 2.6862625 | -1.260703 |
| ENSG00000261701.6 | 1.64E-29 | 2.6825315 | 4.7738432 | -2.091312 |
| ENSG00000136457.9 | 7.06E-24 | 5.0611239 | 6.2454489 | -1.184325 |
| ENSG00000279812.1 | 2.25E-20 | 0.6239735 | 1.727125 | -1.103151 |
| ENSG00000134030.13 | 5.93E-58 | 10.231309 | 11.421691 | -1.190382 |
| ENSG00000166707.10 | 1.21E-105 | 4.7310544 | 7.9117455 | -3.180691 |
| ENSG00000267765.1 | 1.69E-57 | 1.7889036 | 4.2580239 | -2.46912 |
| ENSG00000273007.1 | 3.13E-69 | 2.8724947 | 4.9688409 | -2.096346 |
| ENSG00000044524.10 | 3.96E-17 | 5.6402072 | 6.9424625 | -1.302255 |
| ENSG00000267662.1 | 1.78E-50 | 0.1937432 | 3.2565659 | -3.062823 |
| ENSG00000118231.4 | 2.96E-30 | 1.6644823 | 4.9966489 | -3.332167 |
| ENSG00000198707.14 | 2.86E-50 | 8.8355714 | 10.216794 | -1.381223 |
| ENSG00000136011.14 | 2.48E-21 | 2.3246594 | 4.5428182 | -2.218159 |
| ENSG00000253667.2 | 7.07E-42 | 3.2747136 | 5.279333 | -2.004619 |
| ENSG00000214575.9 | 7.60E-97 | 5.3329126 | 8.5077102 | -3.174798 |
| ENSG00000134874.17 | 2.39E-34 | 9.6378451 | 10.639114 | -1.001269 |
| ENSG00000282033.1 | 2.21E-84 | 1.9685086 | 4.9977705 | -3.029262 |
| ENSG00000268030.1 | 1.07E-32 | 6.4119642 | 7.5186886 | -1.106724 |
| ENSG00000278058.1 | 2.19E-90 | 2.4661358 | 6.013633 | -3.547497 |
| ENSG00000266777.1 | 9.77E-27 | 5.0208169 | 6.1883489 | -1.167532 |
| ENSG00000104332.11 | 4.74E-25 | 8.8368418 | 10.552691 | -1.715849 |
| ENSG00000080947.14 | 4.02E-90 | 6.9952172 | 9.0778443 | -2.082627 |
| ENSG00000107105.14 | 7.74E-12 | 3.0767129 | 4.1930205 | -1.116308 |
| ENSG00000100068.11 | 3.60E-37 | 7.1057265 | 8.218858 | -1.113131 |
| ENSG00000173867.8 | 6.01E-61 | 2.6545735 | 5.1255216 | -2.470948 |
| ENSG00000162878.12 | 5.21E-60 | 9.7974947 | 12.232108 | -2.434613 |
| ENSG00000233521.5 | 1.01E-15 | 2.9728131 | 4.4264955 | -1.453682 |
| ENSG00000186854.10 | 2.35E-19 | 7.1382704 | 9.5361841 | -2.397914 |
| ENSG00000160953.14 | 9.33E-51 | 10.289408 | 11.374748 | -1.08534 |
| ENSG00000163793.12 | 5.12E-11 | 0.5710267 | 1.8759511 | -1.304924 |
| ENSG00000168229.3 | 9.97E-10 | 3.334942 | 4.4024136 | -1.067472 |
| ENSG00000169629.11 | 2.75E-51 | 5.9799995 | 7.4556557 | -1.475656 |
| ENSG00000206149.10 | 1.67E-56 | 9.193032 | 10.598156 | -1.405124 |
| ENSG00000268836.1 | 8.95E-19 | 1.972652 | 3.0833773 | -1.110725 |
| ENSG00000270661.1 | 2.54E-14 | 1.2999874 | 2.4659886 | -1.166001 |
| ENSG00000248015.6 | 6.69E-19 | 4.4093074 | 5.6123898 | -1.203082 |
| ENSG00000168772.10 | 4.05E-26 | 5.4045666 | 7.1082818 | -1.703715 |
| ENSG00000118495.18 | 2.29E-72 | 9.0116666 | 11.004365 | -1.992698 |
| ENSG00000163209.14 | 6.58E-39 | 0.7210604 | 5.1359136 | -4.414853 |
| ENSG00000258515.1 | 8.18E-96 | 2.797947 | 4.8834273 | -2.08548 |
| ENSG00000136111.12 | 5.30E-72 | 9.357201 | 11.588707 | -2.231506 |
| ENSG00000219073.7 | 3.72E-27 | 0.8980103 | 3.7001636 | -2.802153 |
| ENSG00000117834.12 | 3.29E-44 | 2.7376723 | 4.8058625 | -2.06819 |
| ENSG00000175344.16 | 3.77E-17 | 4.8433842 | 6.1698398 | -1.326456 |
| ENSG00000235946.1 | 9.02E-103 | 2.0837769 | 5.5140443 | -3.430267 |
| ENSG00000203786.6 | 2.78E-14 | 0.1534463 | 1.3531557 | -1.199709 |
| ENSG00000172901.19 | 2.26E-57 | 2.9352346 | 6.4570568 | -3.521822 |
| ENSG00000277578.1 | 3.10E-15 | 0.7171597 | 2.0426523 | -1.325493 |
| ENSG00000251432.6 | 1.75E-27 | 4.9621749 | 6.1095477 | -1.147373 |
| ENSG00000106829.18 | 3.37E-32 | 9.1044718 | 10.280668 | -1.176196 |
| ENSG00000078596.10 | 1.71E-86 | 8.092564 | 11.275557 | -3.182993 |
| ENSG00000272732.1 | 4.06E-21 | 4.0732714 | 5.1434057 | -1.070134 |
| ENSG00000228314.1 | 7.62E-13 | 3.4098322 | 5.4192375 | -2.009405 |
| ENSG00000169154.5 | 2.93E-29 | 0.1302551 | 1.6052818 | -1.475027 |
| ENSG00000087085.13 | 1.36E-14 | 5.8081442 | 7.0517216 | -1.243577 |
| ENSG00000156103.15 | 1.39E-17 | 5.7160833 | 6.8718545 | -1.155771 |
| ENSG00000116761.11 | 3.61E-10 | 7.4014076 | 8.8167432 | -1.415336 |
| ENSG00000161082.12 | 3.25E-08 | 6.7546523 | 7.8542977 | -1.099645 |
| ENSG00000254509.1 | 5.02E-32 | 1.7880348 | 3.1878409 | -1.399806 |
| ENSG00000114115.9 | 1.44E-16 | 12.374045 | 13.565475 | -1.19143 |
| ENSG00000244357.3 | 5.99E-35 | 1.0958172 | 2.4060614 | -1.310244 |
| ENSG00000266709.1 | 5.49E-15 | 2.6824267 | 4.159458 | -1.477031 |
| ENSG00000196557.10 | 6.81E-199 | 8.2338878 | 13.286126 | -5.052238 |
| ENSG00000197915.5 | 2.55E-33 | 3.5601408 | 5.78185 | -2.221709 |
| ENSG00000139112.10 | 1.39E-25 | 10.414492 | 11.836143 | -1.421651 |
| ENSG00000267712.5 | 1.61E-23 | 0.4787613 | 2.5917568 | -2.112995 |
| ENSG00000281404.1 | 9.28E-20 | 3.6720072 | 4.7243523 | -1.052345 |
| ENSG00000262601.1 | 1.62E-36 | 2.6034069 | 4.4743557 | -1.870949 |
| ENSG00000136305.11 | 3.00E-63 | 7.9399217 | 9.5829773 | -1.643056 |
| ENSG00000108848.15 | 1.08E-59 | 12.343995 | 13.63534 | -1.291345 |
| ENSG00000095464.9 | 3.85E-22 | 2.7844986 | 3.7856955 | -1.001197 |
| ENSG00000125409.12 | 9.71E-25 | 3.9330897 | 4.9718477 | -1.038758 |
| ENSG00000272551.1 | 1.28E-18 | 2.6524274 | 3.9693409 | -1.316913 |
| ENSG00000221970.1 | 1.14E-42 | 1.6625391 | 3.816433 | -2.153894 |
| ENSG00000253861.1 | 4.18E-16 | 0.3006986 | 2.87255 | -2.571851 |
| ENSG00000266714.6 | 1.99E-125 | 10.288211 | 13.488628 | -3.200418 |
| ENSG00000140538.16 | 7.32E-25 | 5.1686064 | 8.0289091 | -2.860303 |
| ENSG00000272669.1 | 4.30E-36 | 4.4926623 | 5.6763989 | -1.183737 |
| ENSG00000250685.7 | 3.41E-09 | 0.5853203 | 1.6350443 | -1.049724 |
| ENSG00000108551.4 | 7.59E-06 | 7.9173597 | 9.061242 | -1.143882 |
| ENSG00000170262.12 | 9.56E-19 | 1.2809974 | 3.4824932 | -2.201496 |
| ENSG00000122852.14 | 5.64E-17 | 0.3330232 | 2.1395284 | -1.806505 |
| ENSG00000135919.12 | 8.35E-14 | 10.468577 | 11.672351 | -1.203774 |
| ENSG00000261480.1 | 7.31E-50 | 0.4356265 | 2.7158318 | -2.280205 |
| ENSG00000225511.6 | 5.84E-63 | 2.4630752 | 5.0725568 | -2.609482 |
| ENSG00000147655.10 | 1.09E-16 | 1.1978272 | 2.8919318 | -1.694105 |
| ENSG00000166292.11 | 5.22E-14 | 7.5413752 | 9.0492068 | -1.507832 |
| ENSG00000198873.11 | 2.76E-72 | 7.5992726 | 10.482705 | -2.883432 |
| ENSG00000235407.1 | 8.11E-11 | 0.6093599 | 1.785733 | -1.176373 |
| ENSG00000140093.9 | 3.52E-79 | 0.7684239 | 5.4319205 | -4.663497 |
| ENSG00000156049.6 | 1.66E-60 | 5.3239761 | 7.6125091 | -2.288533 |
| ENSG00000255330.8 | 9.89E-58 | 3.3180344 | 5.7044591 | -2.386425 |
| ENSG00000258388.7 | 4.59E-59 | 7.4974847 | 9.6350227 | -2.137538 |
| ENSG00000260578.1 | 4.17E-48 | 2.1791181 | 3.8890716 | -1.709953 |
| ENSG00000278050.1 | 4.23E-40 | 0.2750599 | 2.9879091 | -2.712849 |
| ENSG00000248124.7 | 1.53E-97 | 7.5393002 | 9.5162011 | -1.976901 |
| ENSG00000251450.1 | 3.31E-32 | 1.0185618 | 3.2320955 | -2.213534 |
| ENSG00000254452.1 | 8.73E-21 | 1.7009845 | 4.1838534 | -2.482869 |
| ENSG00000261069.3 | 1.00E-22 | 4.0430673 | 5.3587682 | -1.315701 |
| ENSG00000136574.17 | 1.49E-103 | 5.7475695 | 11.764943 | -6.017374 |
| ENSG00000228677.1 | 3.02E-27 | 0.743442 | 2.5905307 | -1.847089 |
| ENSG00000232926.1 | 1.21E-54 | 2.0055542 | 4.3781636 | -2.372609 |
| ENSG00000269793.5 | 1.21E-16 | 4.0755919 | 5.4671614 | -1.391569 |
| ENSG00000120693.13 | 3.47E-59 | 7.3835714 | 9.1532591 | -1.769688 |
| ENSG00000231852.6 | 1.19E-20 | 5.8584513 | 7.346192 | -1.487741 |
| ENSG00000143669.13 | 4.81E-53 | 8.8911248 | 9.9168841 | -1.025759 |
| ENSG00000270640.1 | 4.16E-22 | 1.9546148 | 4.7862841 | -2.831669 |
| ENSG00000258808.1 | 1.05E-17 | 0.0224124 | 1.2558102 | -1.233398 |
| ENSG00000143631.10 | 3.88E-17 | 5.5225742 | 7.0314295 | -1.508855 |
| ENSG00000229356.1 | 8.00E-29 | 0.9489315 | 2.2089955 | -1.260064 |
| ENSG00000141068.13 | 3.79E-44 | 9.5467339 | 10.857149 | -1.310415 |
| ENSG00000279106.1 | 4.35E-36 | 0.8409764 | 2.6498284 | -1.808852 |
| ENSG00000196805.7 | 2.74E-34 | 0.4284897 | 3.4269182 | -2.998428 |
| ENSG00000246477.3 | 1.54E-40 | 2.9421212 | 4.529483 | -1.587362 |
| ENSG00000186453.12 | 1.12E-31 | 2.4922623 | 4.0027739 | -1.510512 |
| ENSG00000261390.5 | 2.35E-23 | 2.7119162 | 4.2011182 | -1.489202 |
| ENSG00000170160.16 | 3.05E-20 | 3.8364465 | 6.8166227 | -2.980176 |
| ENSG00000172554.11 | 1.35E-10 | 1.7234203 | 2.7336341 | -1.010214 |
| ENSG00000185909.14 | 1.77E-37 | 10.202866 | 11.359458 | -1.156592 |
| ENSG00000266733.5 | 9.44E-23 | 1.4610153 | 2.6073966 | -1.146381 |
| ENSG00000167202.11 | 4.05E-88 | 10.651907 | 12.328943 | -1.677036 |
| ENSG00000225138.7 | 2.07E-41 | 8.1504931 | 9.9105693 | -1.760076 |
| ENSG00000233775.1 | 8.51E-23 | 1.3668086 | 2.9955795 | -1.628771 |
| ENSG00000141753.6 | 7.86E-58 | 13.088446 | 15.339168 | -2.250722 |
| ENSG00000269553.1 | 1.36E-59 | 1.0003728 | 4.6874443 | -3.687072 |
| ENSG00000165030.3 | 3.44E-14 | 10.061138 | 11.164272 | -1.103133 |
| ENSG00000237399.7 | 4.56E-27 | 5.1933883 | 6.2629795 | -1.069591 |
| ENSG00000168209.4 | 5.45E-31 | 11.749303 | 13.955951 | -2.206648 |
| ENSG00000280304.1 | 4.74E-122 | 2.2700148 | 6.910433 | -4.640418 |
| ENSG00000162367.11 | 2.66E-22 | 4.6007098 | 6.1723352 | -1.571625 |
| ENSG00000155269.11 | 6.84E-65 | 4.3114496 | 9.2698932 | -4.958444 |
| ENSG00000153234.13 | 4.18E-14 | 9.5828255 | 11.599373 | -2.016547 |
| ENSG00000261606.5 | 2.84E-12 | 0.8966105 | 2.1250216 | -1.228411 |
| ENSG00000198838.11 | 4.52E-17 | 5.0441043 | 6.5036705 | -1.459566 |
| ENSG00000280237.1 | 3.36E-25 | 6.014395 | 7.897042 | -1.882647 |
| ENSG00000224376.1 | 8.68E-24 | 5.4201878 | 6.5600102 | -1.139822 |
| ENSG00000279989.1 | 7.88E-33 | 2.5587389 | 4.0018739 | -1.443135 |
| ENSG00000261707.1 | 1.32E-15 | 1.2410363 | 2.4061693 | -1.165133 |
| ENSG00000260802.1 | 6.05E-22 | 1.2954189 | 3.945358 | -2.649939 |
| ENSG00000183833.16 | 8.36E-28 | 8.3653475 | 9.4416375 | -1.07629 |
| ENSG00000232633.4 | 6.39E-18 | 2.2657076 | 3.5238091 | -1.258101 |
| ENSG00000170456.14 | 2.16E-34 | 9.0900286 | 10.416864 | -1.326835 |
| ENSG00000225655.5 | 3.78E-40 | 0.6919449 | 2.8749341 | -2.182989 |
| ENSG00000189269.12 | 1.94E-42 | 2.4220637 | 4.5084932 | -2.086429 |
| ENSG00000196562.14 | 3.81E-63 | 10.882773 | 12.854491 | -1.971718 |
| ENSG00000184911.14 | 7.51E-86 | 2.8048542 | 6.3420705 | -3.537216 |
| ENSG00000279622.1 | 7.46E-16 | 0.949306 | 2.4259841 | -1.476678 |
| ENSG00000142046.14 | 3.13E-27 | 7.375399 | 8.3790909 | -1.003692 |
| ENSG00000138653.9 | 6.00E-16 | 0.7811499 | 2.833267 | -2.052117 |
| ENSG00000260822.1 | 8.45E-46 | 8.2123604 | 9.5904636 | -1.378103 |
| ENSG00000224295.2 | 1.66E-14 | 2.2511833 | 3.292242 | -1.041059 |
| ENSG00000227591.5 | 7.16E-19 | 1.1253857 | 2.3853625 | -1.259977 |
| ENSG00000136931.9 | 1.81E-83 | 5.5158959 | 10.615218 | -5.099322 |
| ENSG00000163431.12 | 9.44E-64 | 8.5213885 | 11.775399 | -3.25401 |
| ENSG00000269936.3 | 2.99E-91 | 6.90509 | 11.351934 | -4.446844 |
| ENSG00000237641.1 | 6.64E-45 | 1.906127 | 3.5248205 | -1.618693 |
| ENSG00000131471.6 | 1.35E-08 | 7.9923683 | 9.2056239 | -1.213256 |
| ENSG00000263165.1 | 1.05E-22 | 4.885858 | 6.5678 | -1.681942 |
| ENSG00000253688.1 | 1.69E-30 | 0.911615 | 2.3334943 | -1.421879 |
| ENSG00000275413.1 | 5.78E-33 | 2.4404792 | 3.816692 | -1.376213 |
| ENSG00000121671.11 | 2.99E-55 | 10.197025 | 11.339574 | -1.142549 |
| ENSG00000171560.14 | 3.24E-27 | 0.6235718 | 3.6853341 | -3.061762 |
| ENSG00000101493.10 | 7.51E-47 | 10.060062 | 11.375492 | -1.31543 |
| ENSG00000257242.6 | 5.67E-13 | 2.2357384 | 3.26245 | -1.026712 |
| ENSG00000229325.1 | 5.05E-21 | 1.8875465 | 2.9334511 | -1.045905 |
| ENSG00000254506.1 | 1.42E-53 | 1.330747 | 3.8009466 | -2.4702 |
| ENSG00000010295.19 | 5.83E-112 | 7.8008243 | 10.236576 | -2.435752 |
| ENSG00000258424.1 | 6.08E-25 | 1.4554928 | 2.893958 | -1.438465 |
| ENSG00000223345.3 | 1.66E-79 | 1.6292189 | 5.1171307 | -3.487912 |
| ENSG00000260807.6 | 3.05E-22 | 3.7481527 | 5.3557057 | -1.607553 |
| ENSG00000203814.6 | 2.66E-08 | 7.8917842 | 8.9933898 | -1.101606 |
| ENSG00000103472.9 | 8.49E-63 | 5.1291835 | 7.5272432 | -2.39806 |
| ENSG00000227370.1 | 5.32E-27 | 2.9627069 | 4.4653625 | -1.502656 |
| ENSG00000146938.14 | 2.83E-36 | 7.8224718 | 9.9289307 | -2.106459 |
| ENSG00000261257.1 | 1.79E-23 | 0.513173 | 1.769067 | -1.255894 |
| ENSG00000065325.12 | 3.41E-25 | 1.9290597 | 4.3424227 | -2.413363 |
| ENSG00000235298.1 | 9.39E-27 | 2.992236 | 4.5841148 | -1.591879 |
| ENSG00000224331.2 | 2.45E-18 | 0.4749043 | 1.7463761 | -1.271472 |
| ENSG00000266302.5 | 2.43E-09 | 0.701601 | 1.8738841 | -1.172283 |
| ENSG00000170893.3 | 2.65E-28 | 2.6082714 | 5.7409023 | -3.132631 |
| ENSG00000106034.17 | 8.74E-110 | 7.6751162 | 11.035368 | -3.360252 |
| ENSG00000069431.10 | 1.83E-36 | 7.7414878 | 9.3959489 | -1.654461 |
| ENSG00000281392.1 | 9.64E-47 | 3.4996394 | 5.9184466 | -2.418807 |
| ENSG00000183798.4 | 3.96E-46 | 5.6773699 | 8.7366273 | -3.059257 |
| ENSG00000242294.6 | 3.73E-35 | 8.2890551 | 9.3893273 | -1.100272 |
| ENSG00000279155.1 | 5.51E-65 | 2.0788095 | 4.4762159 | -2.397406 |
| ENSG00000227262.3 | 5.63E-22 | 3.6933864 | 4.9791966 | -1.28581 |
| ENSG00000099139.13 | 1.90E-42 | 8.6907769 | 10.115981 | -1.425204 |
| ENSG00000226648.1 | 6.97E-52 | 3.0025234 | 4.704092 | -1.701569 |
| ENSG00000261143.1 | 2.14E-21 | 1.4847026 | 2.77655 | -1.291847 |
| ENSG00000138722.9 | 4.29E-12 | 5.5583947 | 7.3906909 | -1.832296 |
| ENSG00000261409.1 | 7.81E-160 | 2.5167532 | 8.3796557 | -5.862902 |
| ENSG00000253671.1 | 2.50E-23 | 3.4758723 | 5.0159409 | -1.540069 |
| ENSG00000250251.6 | 5.18E-48 | 9.2464458 | 10.324184 | -1.077738 |
| ENSG00000248485.1 | 5.09E-12 | 4.2808816 | 5.3014761 | -1.020595 |
| ENSG00000239828.6 | 1.25E-45 | 2.0332399 | 4.1089511 | -2.075711 |
| ENSG00000279659.1 | 2.57E-63 | 2.3116036 | 4.7466773 | -2.435074 |
| ENSG00000220884.2 | 5.56E-28 | 0.7864482 | 2.9732398 | -2.186792 |
| ENSG00000269940.1 | 9.76E-42 | 4.7868654 | 5.9622295 | -1.175364 |
| ENSG00000237161.4 | 8.51E-43 | 3.1942661 | 5.8427227 | -2.648457 |
| ENSG00000156395.12 | 2.04E-27 | 1.597053 | 5.4023795 | -3.805327 |
| ENSG00000227230.1 | 6.89E-59 | 1.3796134 | 3.3625284 | -1.982915 |
| ENSG00000269956.1 | 1.24E-26 | 3.0030451 | 4.0249148 | -1.02187 |
| ENSG00000278893.1 | 8.28E-41 | 2.1285449 | 4.9354398 | -2.806895 |
| ENSG00000238057.8 | 2.80E-72 | 2.4452599 | 4.5190909 | -2.073831 |

Table S7. The 137 candidate mRNA expression pattern in ovarian cancer tissues as compared with normal ovarian tissues. (Page 72-74)

| Ensembl_ID | pvalue | mean_x | mean_y | foldchange | GeneSymbol |
| --- | --- | --- | --- | --- | --- |
| ENSG00000085563 | 8.46E-34 | 5.8860916 | 7.2905307 | -1.404439 | ABCB1 |
| ENSG00000154175 | 1.32E-83 | 8.5236611 | 11.112057 | -2.588396 | ABI3BP |
| ENSG00000114948 | 2.01E-37 | 5.6393434 | 7.2773955 | -1.638052 | ADAM23 |
| ENSG00000154736 | 3.55E-61 | 7.9278792 | 10.434788 | -2.506908 | ADAMTS5 |
| ENSG00000150594 | 1.34E-21 | 8.6138718 | 9.9871739 | -1.373302 | ADRA2A |
| ENSG00000144218 | 9.67E-92 | 6.5143339 | 9.5094693 | -2.995135 | AFF3 |
| ENSG00000196581 | 4.72E-19 | 6.6549248 | 8.6363614 | -1.981437 | AJAP1 |
| ENSG00000131016 | 8.54E-45 | 10.938762 | 13.186206 | -2.247444 | AKAP12 |
| ENSG00000163297 | 6.41E-87 | 9.0860134 | 11.648613 | -2.562599 | ANTXR2 |
| ENSG00000071205 | 3.74E-119 | 8.2666379 | 10.679847 | -2.413209 | ARHGAP10 |
| ENSG00000047648 | 4.30E-115 | 6.8484155 | 10.02247 | -3.174055 | ARHGAP6 |
| ENSG00000004848 | 2.19E-110 | 3.7815255 | 11.302623 | -7.521097 | ARX |
| ENSG00000141431 | 8.69E-108 | 4.8210425 | 8.5049955 | -3.683953 | ASXL3 |
| ENSG00000018625 | 8.60E-49 | 5.6692854 | 9.6229375 | -3.953652 | ATP1A2 |
| ENSG00000107518 | 1.35E-62 | 5.3377766 | 9.15835 | -3.820573 | ATRNL1 |
| ENSG00000188848 | 6.87E-11 | 2.7124305 | 3.9222966 | -1.209866 | BEND4 |
| ENSG00000122870 | 6.86E-44 | 8.4111401 | 9.9265227 | -1.515383 | BICC1 |
| ENSG00000173068 | 3.59E-79 | 9.5989126 | 11.749483 | -2.15057 | BNC2 |
| ENSG00000165995 | 2.64E-128 | 5.4391298 | 9.3914693 | -3.952339 | CACNB2 |
| ENSG00000099954 | 1.22E-46 | 5.606305 | 8.3310727 | -2.724768 | CECR2 |
| ENSG00000048740 | 2.35E-56 | 9.5668814 | 11.33989 | -1.773008 | CELF2 |
| ENSG00000111860 | 1.20E-54 | 7.9921723 | 9.2705659 | -1.278394 | CEP85L |
| ENSG00000175344 | 3.77E-17 | 4.8433842 | 6.1698398 | -1.326456 | CHRNA7 |
| ENSG00000147119 | 6.72E-58 | 6.8340549 | 10.045606 | -3.211551 | CHST7 |
| ENSG00000013297 | 2.18E-72 | 8.8432274 | 11.76345 | -2.920223 | CLDN11 |
| ENSG00000158258 | 9.39E-89 | 8.946395 | 13.135734 | -4.189339 | CLSTN2 |
| ENSG00000130176 | 1.56E-13 | 8.4964203 | 10.043678 | -1.547258 | CNN1 |
| ENSG00000118432 | 2.58E-28 | 4.7591874 | 7.2799534 | -2.520766 | CNR1 |
| ENSG00000214575 | 7.60E-97 | 5.3329126 | 8.5077102 | -3.174798 | CPEB1 |
| ENSG00000109625 | 1.17E-49 | 9.936899 | 12.263281 | -2.326382 | CPZ |
| ENSG00000137504 | 6.38E-65 | 10.895588 | 12.0887 | -1.193112 | CREBZF |
| ENSG00000106113 | 1.30E-15 | 2.9809582 | 4.2409216 | -1.259963 | CRHR2 |
| ENSG00000121671 | 2.99E-55 | 10.197025 | 11.339574 | -1.142549 | CRY2 |
| ENSG00000108342 | 3.16E-05 | 1.8008654 | 2.8251352 | -1.02427 | CSF3 |
| ENSG00000198947 | 1.64E-38 | 8.6148475 | 9.9361295 | -1.321282 | DMD |
| ENSG00000135905 | 4.55E-40 | 8.3050921 | 10.207875 | -1.902783 | DOCK10 |
| ENSG00000134874 | 2.39E-34 | 9.6378451 | 10.639114 | -1.001269 | DZIP1 |
| ENSG00000164330 | 1.62E-33 | 7.6639668 | 9.2578114 | -1.593845 | EBF1 |
| ENSG00000151617 | 6.43E-41 | 8.5416021 | 10.419055 | -1.877452 | EDNRA |
| ENSG00000115468 | 2.79E-27 | 9.5498971 | 10.775002 | -1.225105 | EFHD1 |
| ENSG00000107105 | 7.74E-12 | 3.0767129 | 4.1930205 | -1.116308 | ELAVL2 |
| ENSG00000118985 | 4.40E-11 | 9.0060511 | 10.221557 | -1.215506 | ELL2 |
| ENSG00000135333 | 1.41E-13 | 4.9451332 | 6.0360909 | -1.090958 | EPHA7 |
| ENSG00000151491 | 1.62E-42 | 9.8640797 | 11.238184 | -1.374104 | EPS8 |
| ENSG00000117525 | 4.11E-33 | 8.8445737 | 11.780181 | -2.935607 | F3 |
| ENSG00000186453 | 1.12E-31 | 2.4922623 | 4.0027739 | -1.510512 | FAM228A |
| ENSG00000165323 | 1.68E-10 | 4.6454021 | 5.8292386 | -1.183836 | FAT3 |
| ENSG00000138685 | 4.82E-165 | 6.3523699 | 9.7330477 | -3.380678 | FGF2 |
| ENSG00000150907 | 1.50E-49 | 10.806375 | 12.211823 | -1.405448 | FOXO1 |
| ENSG00000139112 | 1.39E-25 | 10.414492 | 11.836143 | -1.421651 | GABARAPL1 |
| ENSG00000164574 | 2.83E-21 | 10.695505 | 11.82029 | -1.124784 | GALNT10 |
| ENSG00000131459 | 1.10E-68 | 8.2953337 | 10.586377 | -2.291044 | GFPT2 |
| ENSG00000112964 | 2.19E-34 | 6.6849757 | 8.0901659 | -1.40519 | GHR |
| ENSG00000150625 | 1.93E-13 | 5.7384468 | 7.2895614 | -1.551115 | GPM6A |
| ENSG00000155269 | 6.84E-65 | 4.3114496 | 9.2698932 | -4.958444 | GPR78 |
| ENSG00000180875 | 4.68E-44 | 4.0691334 | 7.5931602 | -3.524027 | GREM2 |
| ENSG00000002587 | 8.14E-49 | 8.4570055 | 10.894345 | -2.43734 | HS3ST1 |
| ENSG00000125430 | 8.70E-21 | 6.6246888 | 8.0721636 | -1.447475 | HS3ST3B1 |
| ENSG00000163596 | 1.83E-29 | 8.3326604 | 9.3538648 | -1.021204 | ICA1L |
| ENSG00000174498 | 2.34E-09 | 4.0540475 | 5.1309057 | -1.076858 | IGDCC3 |
| ENSG00000115461 | 4.05E-67 | 13.727003 | 16.896616 | -3.169613 | IGFBP5 |
| ENSG00000169047 | 2.00E-20 | 10.483143 | 11.498124 | -1.014981 | IRS1 |
| ENSG00000188385 | 3.72E-139 | 4.0534053 | 8.0265943 | -3.973189 | JAKMIP3 |
| ENSG00000197256 | 2.56E-60 | 11.717488 | 13.202305 | -1.484817 | KANK2 |
| ENSG00000157404 | 4.03E-60 | 6.3974251 | 10.138955 | -3.741529 | KIT |
| ENSG00000146021 | 2.37E-51 | 8.0077322 | 9.2895648 | -1.281833 | KLHL3 |
| ENSG00000170500 | 3.22E-21 | 10.197418 | 11.979859 | -1.782441 | LONRF2 |
| ENSG00000180660 | 1.73E-78 | 2.2392702 | 4.8121102 | -2.57284 | MAB21L1 |
| ENSG00000141639 | 5.80E-40 | 4.6044384 | 6.7160159 | -2.111577 | MAPK4 |
| ENSG00000171444 | 8.31E-65 | 9.2245773 | 12.557914 | -3.333336 | MCC |
| ENSG00000187098 | 1.58E-60 | 8.2897554 | 9.6714193 | -1.381664 | MITF |
| ENSG00000072952 | 6.61E-30 | 8.8290437 | 10.613152 | -1.784109 | MRVI1 |
| ENSG00000186462 | 1.21E-34 | 5.5061026 | 6.980258 | -1.474155 | NAP1L2 |
| ENSG00000158747 | 1.64E-50 | 12.20664 | 13.864164 | -1.657524 | NBL1 |
| ENSG00000149294 | 5.16E-45 | 9.6241671 | 11.763049 | -2.138882 | NCAM1 |
| ENSG00000165030 | 3.44E-14 | 10.061138 | 11.164272 | -1.103133 | NFIL3 |
| ENSG00000101004 | 1.98E-29 | 9.3475554 | 10.608717 | -1.261162 | NINL |
| ENSG00000151623 | 4.35E-48 | 7.8872542 | 9.2293977 | -1.342144 | NR3C2 |
| ENSG00000119508 | 1.36E-20 | 6.9812394 | 10.091199 | -3.109959 | NR4A3 |
| ENSG00000179915 | 1.31E-26 | 1.7198222 | 4.3468341 | -2.627012 | NRXN1 |
| ENSG00000182752 | 2.98E-23 | 5.6776167 | 7.18865 | -1.511033 | PAPPA |
| ENSG00000169116 | 7.27E-29 | 8.3626043 | 10.229394 | -1.86679 | PARM1 |
| ENSG00000099139 | 1.90E-42 | 8.6907769 | 10.115981 | -1.425204 | PCSK5 |
| ENSG00000112541 | 1.31E-28 | 6.5679055 | 8.2755648 | -1.707659 | PDE10A |
| ENSG00000082175 | 4.42E-81 | 7.398595 | 10.804544 | -3.405949 | PGR |
| ENSG00000118762 | 5.58E-58 | 9.9353239 | 11.137659 | -1.202335 | PKD2 |
| ENSG00000182836 | 3.47E-133 | 5.7382246 | 11.179539 | -5.441314 | PLCXD3 |
| ENSG00000152527 | 7.08E-107 | 8.1358842 | 11.509832 | -3.373948 | PLEKHH2 |
| ENSG00000109099 | 7.56E-29 | 10.410653 | 11.440894 | -1.030241 | PMP22 |
| ENSG00000109819 | 1.21E-09 | 4.7544301 | 5.9024864 | -1.148056 | PPARGC1A |
| ENSG00000142611 | 3.95E-43 | 4.9899434 | 7.5389261 | -2.548983 | PRDM16 |
| ENSG00000163637 | 7.66E-31 | 8.9980525 | 10.009165 | -1.011112 | PRICKLE2 |
| ENSG00000117707 | 3.59E-80 | 4.9008196 | 8.8705386 | -3.969719 | PROX1 |
| ENSG00000206260 | 1.05E-18 | 0.0836606 | 1.112592 | -1.028931 | PRR23A |
| ENSG00000156011 | 3.25E-73 | 8.9174967 | 11.196297 | -2.2788 | PSD3 |
| ENSG00000153707 | 4.47E-79 | 6.9211119 | 10.569483 | -3.648371 | PTPRD |
| ENSG00000171016 | 9.66E-73 | 7.1324888 | 9.0788989 | -1.94641 | PYGO1 |
| ENSG00000091428 | 9.81E-42 | 7.0751835 | 8.4697966 | -1.394613 | RAPGEF4 |
| ENSG00000167281 | 3.70E-27 | 2.9789685 | 4.8000534 | -1.821085 | RBFOX3 |
| ENSG00000203867 | 7.23E-79 | 5.1378866 | 8.3424545 | -3.204568 | RBM20 |
| ENSG00000185008 | 8.16E-09 | 6.7766687 | 7.8810727 | -1.104404 | ROBO2 |
| ENSG00000069667 | 6.92E-52 | 8.5857494 | 10.198116 | -1.612367 | RORA |
| ENSG00000072133 | 2.87E-26 | 7.0460675 | 8.1571205 | -1.111053 | RPS6KA6 |
| ENSG00000079102 | 3.86E-41 | 6.6272158 | 8.2394489 | -1.612233 | RUNX1T1 |
| ENSG00000079689 | 6.63E-20 | 0.2749179 | 2.0548739 | -1.779956 | SCGN |
| ENSG00000153253 | 2.47E-25 | 3.1746351 | 5.6812648 | -2.50663 | SCN3A |
| ENSG00000080546 | 9.84E-46 | 9.5003332 | 11.01595 | -1.515617 | SESN1 |
| ENSG00000118473 | 1.16E-18 | 5.4149695 | 6.6493761 | -1.234407 | SGIP1 |
| ENSG00000142178 | 1.21E-11 | 7.6089979 | 9.5135011 | -1.904503 | SIK1 |
| ENSG00000165646 | 1.27E-47 | 2.8266224 | 7.4264534 | -4.599831 | SLC18A2 |
| ENSG00000185052 | 1.90E-30 | 8.3216623 | 9.7101932 | -1.388531 | SLC24A3 |
| ENSG00000147454 | 1.95E-45 | 10.414245 | 11.719049 | -1.304804 | SLC25A37 |
| ENSG00000131389 | 1.63E-16 | 10.88472 | 12.087618 | -1.202899 | SLC6A6 |
| ENSG00000121871 | 1.55E-09 | 2.0126654 | 3.5404773 | -1.527812 | SLITRK3 |
| ENSG00000080503 | 7.62E-40 | 11.15599 | 12.231372 | -1.075382 | SMARCA2 |
| ENSG00000103056 | 4.67E-46 | 6.9764704 | 8.5319614 | -1.555491 | SMPD3 |
| ENSG00000132639 | 6.94E-18 | 7.2057539 | 8.7529534 | -1.547199 | SNAP25 |
| ENSG00000065609 | 1.68E-30 | 3.8626644 | 5.7856693 | -1.923005 | SNAP91 |
| ENSG00000104852 | 2.69E-65 | 12.644621 | 14.12304 | -1.478418 | SNRNP70 |
| ENSG00000112320 | 7.12E-63 | 8.1675289 | 9.8129545 | -1.645426 | SOBP |
| ENSG00000100242 | 5.48E-29 | 11.644771 | 12.819588 | -1.174817 | SUN2 |
| ENSG00000100321 | 2.78E-83 | 9.332143 | 11.608465 | -2.276322 | SYNGR1 |
| ENSG00000128872 | 7.70E-50 | 7.9931704 | 9.3898341 | -1.396664 | TMOD2 |
| ENSG00000183578 | 6.55E-42 | 6.1374885 | 8.5508489 | -2.41336 | TNFAIP8L3 |
| ENSG00000262304 | 5.84E-34 | 7.917389 | 9.2558057 | -1.338417 | TRPV1 |
| ENSG00000121297 | 3.26E-109 | 8.3982835 | 11.347734 | -2.949451 | TSHZ3 |
| ENSG00000168785 | 4.37E-70 | 8.3424926 | 10.827206 | -2.484713 | TSPAN5 |
| ENSG00000127324 | 5.24E-36 | 6.0184444 | 8.8674636 | -2.849019 | TSPAN8 |
| ENSG00000206538 | 2.28E-48 | 7.4070093 | 9.542833 | -2.135824 | VGLL3 |
| ENSG00000188730 | 2.69E-59 | 4.109957 | 8.9621318 | -4.852175 | VWC2 |
| ENSG00000122574 | 1.38E-52 | 8.501701 | 11.672323 | -3.170622 | WIPF3 |
| ENSG00000103489 | 1.32E-46 | 7.4194274 | 9.1872705 | -1.767843 | XYLT1 |
| ENSG00000102053 | 6.79E-78 | 6.128037 | 7.8195864 | -1.691549 | ZC3H12B |
| ENSG00000169946 | 2.25E-61 | 8.5890568 | 10.507011 | -1.917955 | ZFPM2 |
| ENSG00000101493 | 7.51E-47 | 10.060062 | 11.375492 | -1.31543 | ZNF516 |
| ENSG00000161551 | 1.51E-68 | 8.4297062 | 10.265975 | -1.836269 | ZNF577 |
| ENSG00000162415 | 3.04E-72 | 6.9920573 | 9.3447159 | -2.352659 | ZSWIM5 |

| Table S8. The microarray results of the 46 up-regulated circRNAs and 595 down-regulated circRNAs in OC patients  compared with the benign control (fold change > 2 and P-value < 0.05).(Page 75-88) | | | | | | | |
| --- | --- | --- | --- | --- | --- | --- | --- |
| **ProbeName** | **pvalues** | **foldchange** | **Regulation** | **Genomic**  **_length** | **spliced_seq**  **_length** | **best_**  **transcript** | **hostgene** |
| hsa_circ_0039463 | 0.03587121 | 0.421577309 | down | 640 | 296 | NR_001447 | MT1L |
| hsa_circ_0020712 | 0.00380772 | 0.434468884 | down | 1624 | 153 | NM_004357 | CD151 |
| hsa_circ_0027728 | 3.26191E-05 | 0.15600839 | down | 151709 | 3225 | NM_016122 | CCDC41 |
| hsa_circ_0088518 | 0.014471427 | 0.333918868 | down | 4083 | 460 | NM_007209 | RPL35 |
| hsa_circ_0027869 | 0.001397012 | 0.470968496 | down | 23875 | 667 | NM_139319 | SLC17A8 |
| hsa_circ_0066118 | 0.001175582 | 0.371335132 | down | 142 | 142 | NM_003157 | NEK4 |
| hsa_circ_0044532 | 0.002331294 | 0.312639143 | down | 5280 | 1422 | NM_000088 | COL1A1 |
| hsa_circ_0010589 | 0.019164257 | 0.413300849 | down | 18659 | 4225 | NM_005529 | HSPG2 |
| hsa_circ_0028200 | 0.004389516 | 0.189698329 | down | 187 | 187 | NM_016238 | ANAPC7 |
| hsa_circ_0084861 | 0.025024895 | 0.278557142 | down | 2890 | 256 | NM_003821 | RIPK2 |
| hsa_circ_0025632 | 0.040523139 | 0.373206593 | down | 520 | 220 | NM_006152 | LRMP |
| hsa_circ_0070604 | 0.040482743 | 0.433129209 | down | 7937 | 379 | NM_001163436 | TBCK |
| hsa_circ_0014556 | 0.001903572 | 0.201825538 | down | 27380 | 1788 | NM_018489 | ASH1L |
| hsa_circ_0050117 | 0.000207766 | 0.258847807 | down | 6451 | 731 | NM_012181 | FKBP8 |
| hsa_circ_0074834 | 0.027640095 | 0.395358623 | down | 55044 | 1386 | NM_003314 | TTC1 |
| hsa_circ_0082474 | 0.016198619 | 0.374021078 | down | 2034 | 363 | NM_001628 | AKR1B1 |
| hsa_circ_0037799 | 0.048149193 | 0.311124467 | down | 5528 | 2505 | NM_001042476 | CARHSP1 |
| hsa_circ_0063198 | 0.005262067 | 0.461955134 | down | 124 | 124 | NM_014550 | CARD10 |
| hsa_circ_0063293 | 0.003345075 | 0.462389324 | down | 149 | 149 | NM_152221 | CSNK1E |
| hsa_circ_0086964 | 0.013244889 | 0.432446097 | down | 2883 | 682 | NM_006289 | TLN1 |
| hsa_circ_0016556 | 0.019963121 | 2.089069509 | up | 134 | 134 | NM_005426 | TP53BP2 |
| hsa_circ_0044398 | 0.005958412 | 0.317940742 | down | 14768 | 7617 | NM_006546 | IGF2BP1 |
| hsa_circ_0028823 | 0.033582391 | 0.341965805 | down | 14485 | 1309 | NM_006836 | GCN1L1 |
| hsa_circ_0074638 | 0.038727757 | 0.391654303 | down | 149 | 149 | NM_003118 | SPARC |
| hsa_circ_0036316 | 0.001660525 | 0.289488776 | down | 167 | 167 | NM_004255 | COX5A |
| hsa_circ_0060051 | 0.013455423 | 0.301706546 | down | 285 | 285 | NM_181468 | EIF6 |
| hsa_circ_0000691 | 0.041035267 | 0.447971844 | down | 85 | 85 | NM_014699 | ZNF646 |
| hsa_circ_0010997 | 0.002334184 | 0.498189337 | down | 17437 | 608 | NM_024887 | DHDDS |
| hsa_circ_0025338 | 0.036812264 | 0.387954993 | down | 14235 | 1599 | NM_014718 | CLSTN3 |
| hsa_circ_0070884 | 7.14697E-09 | 0.186648185 | down | 92682 | 932 | NM_145207 | SPATA5 |
| hsa_circ_0051036 | 0.025853586 | 0.231579815 | down | 6335 | 1041 | NM_001436 | FBL |
| hsa_circ_0052531 | 0.013605908 | 0.265575496 | down | 9764 | 229 | NM_003887 | ASAP2 |
| hsa_circ_0003972 | 0.000124309 | 0.156660111 | down | 22631 | 309 | NM_014612 | FAM120A |
| hsa_circ_0033008 | 0.048211391 | 0.390155028 | down | 34880 | 904 | NM_001008530 | LGMN |
| hsa_circ_0027325 | 0.044428468 | 0.437795298 | down | 940 | 450 | NM_000785 | CYP27B1 |
| hsa_circ_0009913 | 0.008925393 | 0.210533917 | down | 15449 | 1396 | NM_014874 | MFN2 |
| hsa_circ_0074857 | 0.019207934 | 0.319335536 | down | 210 | 210 | NM_001122679 | ODZ2 |
| hsa_circ_0060515 | 0.022003402 | 0.230361912 | down | 7501 | 585 | NM_001124756 | PABPC1L |
| hsa_circ_0085239 | 0.035645951 | 0.467610324 | down | 11497 | 688 | NM_015902 | UBR5 |
| hsa_circ_0037782 | 0.000264163 | 0.315272737 | down | 3615 | 684 | NM_024109 | METTL22 |
| hsa_circ_0013981 | 0.036512195 | 0.33959376 | down | 919 | 672 | NM_005850 | SF3B4 |
| hsa_circ_0004738 | 0.043876197 | 2.871415876 | up | 1893 | 354 | NM_022897 | RANBP17 |
| hsa_circ_0083349 | 0.023839934 | 0.270673222 | down | 192 | 192 | NM_004462 | FDFT1 |
| hsa_circ_0090168 | 0.023682412 | 0.416050364 | down | 88898 | 525 | NM_016937 | POLA1 |
| hsa_circ_0025036 | 0.007447221 | 0.243578493 | down | 1167 | 174 | NM_202002 | FOXM1 |
| hsa_circ_0038000 | 0.005060836 | 0.244926009 | down | 379 | 379 | --- | --- |
| hsa_circ_0075328 | 0.045928777 | 0.403638349 | down | 35044 | 2673 | NM_198868 | TBC1D9B |
| hsa_circ_0042669 | 0.01154674 | 0.297985205 | down | 2235 | 890 | NM_003170 | SUPT6H |
| hsa_circ_0064559 | 2.07002E-05 | 0.47720478 | down | 199441 | 1006 | NM_144633 | KCNH8 |
| hsa_circ_0078713 | 0.00269952 | 7.050319171 | up | 3446 | 608 | NM_003247 | THBS2 |
| hsa_circ_0080493 | 0.014521295 | 0.454446521 | down | 9975 | 347 | NM_032408 | BAZ1B |
| hsa_circ_0074590 | 0.015888896 | 0.326636541 | down | 28867 | 1462 | NM_001155 | ANXA6 |
| hsa_circ_0085962 | 6.04645E-06 | 2.541334979 | up | 5605 | 1816 | NM_030974 | SHARPIN |
| hsa_circ_0085827 | 0.019939541 | 0.327750235 | down | 1097 | 965 | NM_002346 | LY6E |
| hsa_circ_0007906 | 0.049904376 | 0.39191322 | down | 4327 | 1006 | NM_133330 | WHSC1 |
| hsa_circ_0002046 | 0.044213804 | 0.325275757 | down | 6000 | 286 | NM_014363 | SACS |
| hsa_circ_0025243 | 0.004739958 | 0.206515155 | down | 835 | 401 | NM_001164094 | COPS7A |
| hsa_circ_0009218 | 0.002005992 | 0.434750672 | down | 2484 | 1077 | NM_198576 | AGRN |
| hsa_circ_0038869 | 0.008810559 | 0.496827217 | down | 1022 | 1022 | NM_001145812 | SH2B1 |
| hsa_circ_0056428 | 1.50865E-05 | 0.174394621 | down | 5661 | 199 | NR_027671 | UGGT1 |
| hsa_circ_0038878 | 0.016574095 | 0.309692506 | down | 207 | 207 | NM_001178098 | CD19 |
| hsa_circ_0017969 | 0.017086591 | 0.352611082 | down | 5607 | 186 | NM_005028 | PIP4K2A |
| hsa_circ_0059784 | 0.046804896 | 2.37892498 | up | 60417 | 422 | NM_015338 | ASXL1 |
| hsa_circ_0015009 | 0.030858402 | 0.490836833 | down | 3741 | 513 | NM_001136219 | FCGR2A |
| hsa_circ_0064375 | 0.042296796 | 0.329380833 | down | 5941 | 309 | NM_018306 | TMEM40 |
| hsa_circ_0064372 | 0.03685325 | 0.490314457 | down | 11705 | 432 | NM_018306 | TMEM40 |
| hsa_circ_0083411 | 0.018051743 | 0.339213635 | down | 6859 | 281 | NM_016353 | ZDHHC2 |
| hsa_circ_0048591 | 0.003157485 | 0.250640175 | down | 7095 | 777 | NM_018074 | CCDC94 |
| hsa_circ_0062359 | 0.046835197 | 0.484345317 | down | 3952 | 356 | NM_058004 | PI4KA |
| hsa_circ_0026581 | 0.025581435 | 0.369576529 | down | 16535 | 3033 | NM_012291 | ESPL1 |
| hsa_circ_0017166 | 0.017330371 | 0.464780681 | down | 46558 | 408 | NM_001035 | RYR2 |
| hsa_circ_0012780 | 0.001593902 | 0.425788904 | down | 58635 | 1458 | NM_176877 | INADL |
| hsa_circ_0034125 | 0.006965177 | 0.288347637 | down | 38883 | 38883 | NR_003325 | SNORD116-10 |
| hsa_circ_0057397 | 0.004089578 | 0.260028886 | down | 5016 | 630 | NM_000090 | COL3A1 |
| hsa_circ_0087303 | 0.020482677 | 0.245195249 | down | 153 | 153 | NM_007005 | TLE4 |
| hsa_circ_0035080 | 0.035280345 | 0.352993708 | down | 27086 | 2685 | NM_025137 | SPG11 |
| hsa_circ_0082680 | 2.02952E-05 | 0.366262073 | down | 100467 | 1208 | NM_022740 | HIPK2 |
| hsa_circ_0086757 | 0.010764728 | 0.338868496 | down | 12309 | 196 | NM_147164 | CNTFR |
| hsa_circ_0062215 | 0.000936198 | 11.42383106 | up | 12283 | 840 | NM_007098 | CLTCL1 |
| hsa_circ_0023074 | 0.006545073 | 0.322481093 | down | 1618 | 433 | NR_030767 | ANKRD13D |
| hsa_circ_0064171 | 0.020674585 | 0.27278511 | down | 4034 | 263 | NM_001198793 | ARPC4-TTLL3 |
| hsa_circ_0049327 | 0.036818903 | 0.322501942 | down | 42621 | 1332 | NM_001005360 | DNM2 |
| hsa_circ_0038307 | 0.005879966 | 0.212559961 | down | 50323 | 2039 | NM_001160364 | TMC7 |
| hsa_circ_0035602 | 0.029581582 | 0.406805356 | down | 46054 | 2203 | NM_015059 | TLN2 |
| hsa_circ_0056516 | 0.000403627 | 0.449234217 | down | 1769 | 1769 | NM_207363 | NCKAP5 |
| hsa_circ_0070008 | 0.028486569 | 0.279161822 | down | 3752 | 456 | NM_003715 | USO1 |
| hsa_circ_0076390 | 0.040485366 | 0.434191339 | down | 5643 | 368 | NM_015255 | UBR2 |
| hsa_circ_0049837 | 0.014708153 | 0.454156186 | down | 14604 | 1398 | NM_058243 | BRD4 |
| hsa_circ_0025136 | 0.027496364 | 0.415549644 | down | 998 | 381 | NM_001159576 | SCNN1A |
| hsa_circ_0022485 | 0.000896138 | 0.200938542 | down | 9353 | 1541 | NM_198335 | GANAB |
| hsa_circ_0062462 | 3.35887E-05 | 0.359586874 | down | 33471 | 33471 | --- | --- |
| hsa_circ_0005453 | 0.004027913 | 0.272776857 | down | 26468 | 645 | NM_006749 | SLC20A2 |
| hsa_circ_0055430 | 0.033802663 | 0.263268852 | down | 7455 | 224 | NM_006634 | VAMP5 |
| hsa_circ_0080413 | 0.00042961 | 0.432386631 | down | 300580 | 1257 | NM_001127231 | AUTS2 |
| hsa_circ_0074663 | 0.007673074 | 0.199720916 | down | 144 | 144 | NM_005754 | G3BP1 |
| hsa_circ_0024301 | 0.030227541 | 0.417189522 | down | 9143 | 299 | NM_001562 | IL18 |
| hsa_circ_0090752 | 0.000709635 | 0.279134773 | down | 30694 | 3553 | NM_031407 | HUWE1 |
| hsa_circ_0078790 | 0.004805226 | 0.445900502 | down | 6211 | 5344 | NM_144781 | PDCD2 |
| hsa_circ_0004426 | 0.016079245 | 19.15948341 | up | 3874 | 227 | NM_005504 | BCAT1 |
| hsa_circ_0049358 | 0.004656598 | 0.492979216 | down | 113 | 113 | NM_199141 | CARM1 |
| hsa_circ_0049328 | 0.001181694 | 0.289628269 | down | 46230 | 1384 | NM_001005360 | DNM2 |
| hsa_circ_0027841 | 0.041097897 | 0.300363655 | down | 49842 | 827 | NM_152788 | ANKS1B |
| hsa_circ_0044884 | 0.021235794 | 0.257743433 | down | 2991 | 198 | NM_030938 | VMP1 |
| hsa_circ_0025237 | 1.29025E-05 | 0.389187233 | down | 6111 | 742 | NM_001135734 | ZNF384 |
| hsa_circ_0012107 | 2.68248E-05 | 0.35578066 | down | 13581 | 255 | NM_174963 | ST3GAL3 |
| hsa_circ_0018443 | 0.015641023 | 0.26500651 | down | 3105 | 240 | NM_001242359 | RHOBTB1 |
| hsa_circ_0016149 | 0.044150832 | 0.468614964 | down | 3511 | 521 | NM_002646 | PIK3C2B |
| hsa_circ_0084845 | 0.037225513 | 0.356402151 | down | 47071 | 4864 | NM_003909 | CPNE3 |
| hsa_circ_0051309 | 0.008313646 | 0.358616599 | down | 28622 | 8033 | NM_001410 | MEGF8 |
| hsa_circ_0063221 | 0.006271802 | 0.291902386 | down | 19492 | 12236 | NM_018957 | SH3BP1 |
| hsa_circ_0020769 | 0.027037221 | 0.497239201 | down | 919 | 919 | NM_001909 | CTSD |
| hsa_circ_0081277 | 0.029952919 | 0.483601882 | down | 16601 | 1262 | NM_001244580 | TRRAP |
| hsa_circ_0049661 | 0.022559871 | 0.31621335 | down | 2751 | 509 | NM_017722 | TRMT1 |
| hsa_circ_0072742 | 0.027169116 | 0.494267518 | down | 51045 | 2578 | NM_001164664 | MAST4 |
| hsa_circ_0025423 | 0.00012967 | 0.181822523 | down | 16457 | 1268 | NM_000014 | A2M |
| hsa_circ_0026685 | 0.013263616 | 0.476435338 | down | 2775 | 148 | NM_001002031 | ATP5G2 |
| hsa_circ_0082676 | 0.030517646 | 0.377801523 | down | 117783 | 1971 | NM_022740 | HIPK2 |
| hsa_circ_0083635 | 0.005357092 | 0.27228446 | down | 441 | 188 | NM_021174 | KIAA1967 |
| hsa_circ_0036781 | 0.011517751 | 0.309804004 | down | 1394 | 468 | NM_006384 | CIB1 |
| hsa_circ_0082460 | 0.047757164 | 0.339846188 | down | 416 | 416 | NM_001628 | AKR1B1 |
| hsa_circ_0045997 | 0.011016192 | 0.413127999 | down | 1430 | 570 | NM_001166348 | SLC26A11 |
| hsa_circ_0002078 | 0.032747828 | 0.336911018 | down | 3495 | 194 | NM_004996 | ABCC1 |
| hsa_circ_0026085 | 0.042052454 | 0.292647113 | down | 24192 | 12032 | NM_003482 | MLL2 |
| hsa_circ_0065887 | 0.035825075 | 0.332847314 | down | 76388 | 1680 | NM_004947 | DOCK3 |
| hsa_circ_0061121 | 0.044389981 | 2.281787203 | up | 166 | 166 | NM_016354 | SLCO4A1 |
| hsa_circ_0026098 | 0.008008282 | 0.265160481 | down | 5521 | 4449 | NM_003482 | MLL2 |
| hsa_circ_0063186 | 0.025073257 | 0.41886185 | down | 6339 | 423 | NM_024681 | KCTD17 |
| hsa_circ_0089323 | 0.002858082 | 0.409319178 | down | 81 | 81 | NM_001122823 | GTF3C5 |
| hsa_circ_0004794 | 0.04800258 | 0.423668301 | down | 9801 | 586 | NM_021818 | SAV1 |
| hsa_circ_0025777 | 0.01962353 | 0.378149135 | down | 1032 | 284 | NM_006390 | IPO8 |
| hsa_circ_0022030 | 0.031781344 | 0.37337675 | down | 11972 | 594 | NM_014342 | MTCH2 |
| hsa_circ_0009861 | 0.0031062 | 0.425048007 | down | 1188 | 350 | NM_005957 | MTHFR |
| hsa_circ_0070050 | 0.026574567 | 0.372514113 | down | 16432 | 4122 | NM_020859 | SHROOM3 |
| hsa_circ_0002709 | 0.008117075 | 0.274789473 | down | 153619 | 561 | NM_000947 | PRIM2 |
| hsa_circ_0004976 | 0.008271501 | 0.468830439 | down | 3958 | 372 | NM_018263 | ASXL2 |
| hsa_circ_0016373 | 0.026289963 | 0.27733622 | down | 57389 | 1727 | NR_037667 | INTS7 |
| hsa_circ_0059815 | 0.030236303 | 0.324619738 | down | 12987 | 1388 | NM_025227 | BPIFB2 |
| hsa_circ_0004104 | 0.019636967 | 0.313115899 | down | 5698 | 553 | NM_003118 | SPARC |
| hsa_circ_0059502 | 0.010361255 | 0.294059124 | down | 5225 | 689 | NM_152227 | SNX5 |
| hsa_circ_0090347 | 0.046556055 | 0.377692918 | down | 55706 | 1399 | NM_032591 | SLC9A7 |
| hsa_circ_0082684 | 0.036038365 | 0.279621538 | down | 22919 | 959 | NM_022750 | PARP12 |
| hsa_circ_0045477 | 0.007375327 | 0.271797035 | down | 7394 | 1370 | NM_002266 | KPNA2 |
| hsa_circ_0042703 | 0.003213084 | 0.226666477 | down | 546 | 546 | NM_138463 | TLCD1 |
| hsa_circ_0027518 | 0.000820421 | 9.989630403 | up | 10102 | 697 | NM_006654 | FRS2 |
| hsa_circ_0048512 | 8.41731E-05 | 0.410613185 | down | 131 | 131 | NM_012398 | PIP5K1C |
| hsa_circ_0081365 | 0.001446669 | 0.251786914 | down | 6690 | 439 | TCONS_l2_00026126 | TCONS_l2_00026126 |
| hsa_circ_0066986 | 4.38368E-05 | 0.214793008 | down | 4947 | 205 | NR_026698 | KPNA1 |
| hsa_circ_0044587 | 0.01898075 | 0.297361006 | down | 6918 | 185 | NM_025149 | ACSF2 |
| hsa_circ_0051035 | 0.040927095 | 0.268329906 | down | 6063 | 870 | NM_001436 | FBL |
| hsa_circ_0008121 | 0.008419107 | 0.286876235 | down | 534 | 178 | NM_004603 | STX1A |
| hsa_circ_0007987 | 0.029320741 | 0.470881959 | down | 14598 | 811 | NM_003704 | FAM193A |
| hsa_circ_0051967 | 0.01415656 | 0.370212072 | down | 4323 | 922 | NM_025129 | FUZ |
| hsa_circ_0014662 | 0.040938806 | 0.4714265 | down | 6559 | 286 | NM_003145 | SSR2 |
| hsa_circ_0025045 | 1.34359E-05 | 0.153027573 | down | 28757 | 1154 | NM_003324 | TULP3 |
| hsa_circ_0081471 | 0.042326461 | 0.428747372 | down | 167 | 167 | NM_030935 | TSC22D4 |
| hsa_circ_0009782 | 0.019587322 | 0.290695572 | down | 146903 | 7024 | NM_004958 | MTOR |
| hsa_circ_0075500 | 0.02623677 | 3.029162749 | up | 4936 | 449 | NM_001135750 | PSMG4 |
| hsa_circ_0026181 | 0.018278719 | 0.183442833 | down | 159 | 159 | NM_001037806 | NCKAP5L |
| hsa_circ_0036608 | 0.005951318 | 0.272390524 | down | 8135 | 2836 | NM_004213 | SLC28A1 |
| hsa_circ_0092140 | 0.016298441 | 0.484282599 | down | 3624 | 523 | NM_001363 | DKC1 |
| hsa_circ_0055853 | 0.014233009 | 0.357324443 | down | 102676 | 263 | NM_002285 | AFF3 |
| hsa_circ_0025092 | 0.007105991 | 0.373034805 | down | 31308 | 1848 | NM_000552 | VWF |
| hsa_circ_0039813 | 0.042062627 | 0.362147134 | down | 8025 | 2304 | NM_013241 | FHOD1 |
| hsa_circ_0087931 | 0.001749112 | 6.670804839 | up | 3983 | 404 | NM_032012 | C9orf5 |
| hsa_circ_0024513 | 0.033024949 | 0.336038105 | down | 3526 | 586 | NM_016146 | TRAPPC4 |
| hsa_circ_0044886 | 0.01211746 | 0.260276227 | down | 29602 | 363 | NM_030938 | VMP1 |
| hsa_circ_0034844 | 0.014854516 | 0.27596206 | down | 2856 | 1517 | NM_138477 | CDAN1 |
| hsa_circ_0081978 | 0.004885933 | 0.468194376 | down | 68768 | 1043 | NM_014705 | DOCK4 |
| hsa_circ_0050991 | 0.010575791 | 0.325577505 | down | 11566 | 6689 | NM_022835 | PLEKHG2 |
| hsa_circ_0032233 | 0.006609371 | 0.35414459 | down | 3660 | 994 | NM_002083 | GPX2 |
| hsa_circ_0065053 | 0.02058216 | 0.332203741 | down | 330 | 330 | NM_016598 | ZDHHC3 |
| hsa_circ_0059221 | 0.024440951 | 0.393367666 | down | 82 | 82 | NM_001110514 | EBF4 |
| hsa_circ_0011481 | 0.015422315 | 0.283682994 | down | 33339 | 1095 | NM_198040 | PHC2 |
| hsa_circ_0026615 | 0.001582065 | 0.313601967 | down | 7375 | 1024 | NM_015665 | AAAS |
| hsa_circ_0034786 | 0.010334753 | 0.292944172 | down | 353 | 242 | NM_015289 | VPS39 |
| hsa_circ_0059368 | 0.019894381 | 0.492424425 | down | 68478 | 605 | NM_203327 | SLC23A2 |
| hsa_circ_0001047 | 0.017915624 | 0.273392792 | down | 5916 | 524 | NM_015341 | NCAPH |
| hsa_circ_0042621 | 0.035268114 | 0.306261699 | down | 7641 | 3013 | NM_014680 | KIAA0100 |
| hsa_circ_0009520 | 0.041171095 | 0.319923007 | down | 6065 | 722 | NM_024654 | NOL9 |
| hsa_circ_0063566 | 0.004605453 | 0.443363743 | down | 4510 | 454 | NM_001098 | ACO2 |
| hsa_circ_0067360 | 0.043225629 | 0.341368431 | down | 36775 | 2973 | NM_015268 | DNAJC13 |
| hsa_circ_0052623 | 0.006954911 | 0.346539539 | down | 16632 | 608 | NM_152391 | PQLC3 |
| hsa_circ_0021434 | 0.006059065 | 0.225387412 | down | 4666 | 161 | NM_181507 | HPS5 |
| hsa_circ_0011262 | 0.010214223 | 0.484461744 | down | 31820 | 883 | NM_004814 | SNRNP40 |
| hsa_circ_0052284 | 0.012471566 | 0.45405156 | down | 2400 | 1379 | NM_018337 | ZNF444 |
| hsa_circ_0040211 | 0.037132188 | 0.458902469 | down | 4495 | 531 | NM_007242 | DDX19B |
| hsa_circ_0022758 | 0.003151136 | 0.468375766 | down | 15498 | 2510 | NM_013265 | C11orf2 |
| hsa_circ_0066419 | 0.027899137 | 0.340983883 | down | 84499 | 2526 | NM_002841 | PTPRG |
| hsa_circ_0057734 | 0.040030774 | 0.353235369 | down | 11227 | 659 | NR_033915 | ORC2 |
| hsa_circ_0000862 | 0.01936556 | 0.329733804 | down | 1799 | 1799 | NM_025078 | PQLC1 |
| hsa_circ_0070713 | 0.001304872 | 6.44442685 | up | 4330 | 345 | NM_001221 | CAMK2D |
| hsa_circ_0038846 | 0.03367131 | 0.381879873 | down | 9171 | 434 | NM_138414 | CCDC101 |
| hsa_circ_0046107 | 0.042190097 | 0.371121885 | down | 1813 | 1231 | NM_138570 | SLC38A10 |
| hsa_circ_0091830 | 0.035166832 | 0.379198549 | down | 1034 | 709 | NM_001110556 | FLNA |
| hsa_circ_0086444 | 0.037564075 | 0.396319146 | down | 6486 | 185 | NM_017645 | HAUS6 |
| hsa_circ_0041117 | 3.88981E-05 | 0.476800485 | down | 10750 | 956 | NM_014972 | TCF25 |
| hsa_circ_0049042 | 0.03314779 | 0.498133701 | down | 2579 | 568 | NM_024552 | CERS4 |
| hsa_circ_0070576 | 3.56414E-05 | 0.378055974 | down | 96395 | 384 | NM_024751 | GSTCD |
| hsa_circ_0000302 | 0.018079171 | 0.471361378 | down | 1197 | 351 | NM_001080547 | SPI1 |
| hsa_circ_0022374 | 0.001121926 | 0.470378712 | down | 98 | 98 | NM_013402 | FADS1 |
| hsa_circ_0002848 | 0.01418277 | 0.360565754 | down | 9532 | 9532 | NM_002841 | PTPRG |
| hsa_circ_0038469 | 0.002312225 | 0.250095779 | down | 13483 | 735 | NM_003366 | UQCRC2 |
| hsa_circ_0039123 | 0.04726828 | 0.393395789 | down | 5855 | 2188 | NM_152288 | ORAI3 |
| hsa_circ_0019707 | 0.005524053 | 0.241974747 | down | 15401 | 2706 | NM_004193 | GBF1 |
| hsa_circ_0047716 | 0.004956919 | 0.295372958 | down | 7322 | 404 | NM_005359 | SMAD4 |
| hsa_circ_0073975 | 0.017014506 | 0.316911583 | down | 32866 | 1576 | NM_021982 | SEC24A |
| hsa_circ_0019894 | 0.020633213 | 0.415591883 | down | 10251 | 2890 | NM_000494 | COL17A1 |
| hsa_circ_0013257 | 0.003872187 | 0.294888005 | down | 351735 | 1026 | NM_000110 | DPYD |
| hsa_circ_0039393 | 0.015312359 | 0.198615072 | down | 34159 | 850 | NM_001080432 | FTO |
| hsa_circ_0010770 | 0.033861265 | 0.495881983 | down | 473 | 377 | NM_005529 | HSPG2 |
| hsa_circ_0042266 | 0.034149028 | 2.517718841 | up | 13871 | 314 | NM_001082968 | TOM1L2 |
| hsa_circ_0051857 | 0.000347051 | 0.260980969 | down | 4226 | 2384 | NM_002152 | HRC |
| hsa_circ_0048258 | 0.02307796 | 0.489059912 | down | 3361 | 662 | NM_213604 | ADAMTSL5 |
| hsa_circ_0023042 | 0.0004149 | 0.430107509 | down | 4311 | 2397 | NM_001040716 | PC |
| hsa_circ_0041990 | 0.024371277 | 0.436408075 | down | 535 | 535 | NM_001037144 | CNTROB |
| hsa_circ_0020492 | 0.024149576 | 0.285395865 | down | 2876 | 376 | NM_002417 | MKI67 |
| hsa_circ_0034937 | 0.047627114 | 0.355796722 | down | 2659 | 390 | NM_024956 | TMEM62 |
| hsa_circ_0058792 | 0.002612992 | 0.436349505 | down | 41310 | 510 | NM_001037131 | AGAP1 |
| hsa_circ_0054190 | 0.01464503 | 0.20977623 | down | 8074 | 344 | NM_003618 | MAP4K3 |
| hsa_circ_0090349 | 0.002829687 | 0.234252278 | down | 72818 | 1667 | NM_032591 | SLC9A7 |
| hsa_circ_0070172 | 0.007494083 | 0.494427195 | down | 53195 | 3460 | NM_025074 | FRAS1 |
| hsa_circ_0063359 | 1.43108E-06 | 0.173492034 | down | 2450 | 516 | NM_004286 | GTPBP1 |
| hsa_circ_0049790 | 0.024015289 | 0.315786212 | down | 22724 | 897 | NM_032433 | ZNF333 |
| hsa_circ_0022465 | 0.043321164 | 2.974510484 | up | 8448 | 2334 | NM_153265 | EML3 |
| hsa_circ_0084793 | 0.007676105 | 0.313805101 | down | 17041 | 9725 | NM_024721 | ZFHX4 |
| hsa_circ_0071731 | 0.025567694 | 0.34355309 | down | 143 | 143 | NM_024830 | LPCAT1 |
| hsa_circ_0060937 | 0.040711529 | 0.351658097 | down | 3117 | 350 | NM_000782 | CYP24A1 |
| hsa_circ_0041957 | 0.022377459 | 0.463645013 | down | 1323 | 377 | NM_001080424 | KDM6B |
| hsa_circ_0025381 | 0.025856233 | 3.070584707 | up | 12881 | 1632 | NM_144670 | A2ML1 |
| hsa_circ_0022445 | 0.020440446 | 0.335615472 | down | 130 | 130 | NM_001404 | EEF1G |
| hsa_circ_0066939 | 0.023734797 | 0.30174437 | down | 9215 | 756 | NM_199420 | POLQ |
| hsa_circ_0049379 | 0.010475214 | 0.344297998 | down | 1468 | 504 | NM_000527 | LDLR |
| hsa_circ_0040917 | 0.036555464 | 0.343515387 | down | 3807 | 1451 | NM_001242757 | SLC22A31 |
| hsa_circ_0022036 | 0.0149079 | 0.401555709 | down | 7398 | 340 | NM_014342 | MTCH2 |
| hsa_circ_0018160 | 0.013630887 | 0.373515003 | down | 137287 | 2478 | NM_001024628 | NRP1 |
| hsa_circ_0015386 | 0.036718264 | 0.37283283 | down | 23643 | 3305 | NM_033127 | SEC16B |
| hsa_circ_0031890 | 0.015042403 | 0.341224726 | down | 4106 | 339 | NM_002863 | PYGL |
| hsa_circ_0010960 | 0.000207297 | 0.449941806 | down | 2378 | 2378 | NM_178422 | PAQR7 |
| hsa_circ_0081573 | 0.032704576 | 0.357516761 | down | 308 | 188 | NM_000602 | SERPINE1 |
| hsa_circ_0022143 | 1.40138E-05 | 0.352272404 | down | 2947 | 538 | NM_003627 | SLC43A1 |
| hsa_circ_0027054 | 0.014208134 | 0.210756611 | down | 230 | 230 | NM_013449 | BAZ2A |
| hsa_circ_0035512 | 0.004261844 | 0.427190507 | down | 98092 | 2641 | NM_004998 | MYO1E |
| hsa_circ_0063321 | 0.021837067 | 0.310596898 | down | 6095 | 775 | NM_001098504 | DDX17 |
| hsa_circ_0078984 | 0.005761033 | 0.215114517 | down | 970 | 970 | --- | --- |
| hsa_circ_0085535 | 0.001097972 | 0.279048595 | down | 3187 | 1811 | NM_002467 | MYC |
| hsa_circ_0055035 | 0.011642498 | 0.377673826 | down | 29248 | 245 | NM_032208 | ANTXR1 |
| hsa_circ_0020676 | 0.016094045 | 0.425319208 | down | 34096 | 1076 | NM_021008 | DEAF1 |
| hsa_circ_0014545 | 0.015223553 | 0.414354519 | down | 126 | 126 | NM_001105203 | RUSC1 |
| hsa_circ_0075798 | 0.005215809 | 0.288479383 | down | 23897 | 2356 | NM_001080480 | MBOAT1 |
| hsa_circ_0036053 | 0.020816797 | 0.416207149 | down | 9014 | 582 | NM_017882 | CLN6 |
| hsa_circ_0045730 | 0.0260318 | 0.421634504 | down | 641 | 308 | NM_199242 | UNC13D |
| hsa_circ_0016135 | 0.000627356 | 0.471166638 | down | 3629 | 377 | NM_014935 | PLEKHA6 |
| hsa_circ_0091908 | 0.027310491 | 0.360852806 | down | 13868 | 5392 | NM_001110556 | FLNA |
| hsa_circ_0010121 | 0.041975843 | 0.407879032 | down | 3381 | 931 | NM_004431 | EPHA2 |
| hsa_circ_0038880 | 0.004017914 | 0.447065853 | down | 431 | 431 | NM_032815 | NFATC2IP |
| hsa_circ_0010688 | 0.00796973 | 0.446174151 | down | 4573 | 1294 | NM_005529 | HSPG2 |
| hsa_circ_0074356 | 0.03271653 | 0.402244035 | down | 5694 | 723 | NM_014773 | KIAA0141 |
| hsa_circ_0016368 | 0.022668815 | 0.214787811 | down | 38027 | 1604 | NR_037667 | INTS7 |
| hsa_circ_0023967 | 0.019845509 | 0.297501589 | down | 10783 | 3804 | NM_007173 | PRSS23 |
| hsa_circ_0075784 | 0.020253215 | 0.340895508 | down | 19893 | 5517 | NM_153042 | KDM1B |
| hsa_circ_0013714 | 0.049754221 | 0.323192011 | down | 12862 | 1942 | NM_003594 | TTF2 |
| hsa_circ_0026547 | 0.043032893 | 0.317757512 | down | 5021 | 2866 | NM_015319 | TENC1 |
| hsa_circ_0042724 | 0.023237642 | 0.445036516 | down | 3124 | 967 | NM_004475 | FLOT2 |
| hsa_circ_0087543 | 0.025998624 | 0.3090062 | down | 67216 | 1438 | NM_014612 | FAM120A |
| hsa_circ_0031180 | 0.022024549 | 0.319674248 | down | 24795 | 5939 | NM_001170629 | CHD8 |
| hsa_circ_0034755 | 0.01920611 | 0.259216325 | down | 5931 | 1927 | NM_001164273 | MGA |
| hsa_circ_0019377 | 0.005473132 | 6.650526827 | up | 10836 | 642 | NM_018425 | PI4K2A |
| hsa_circ_0007898 | 0.017150844 | 0.312815323 | down | 12807 | 797 | NM_002645 | PIK3C2A |
| hsa_circ_0034399 | 0.023263766 | 0.342504525 | down | 164212 | 781 | NM_001130010 | C15orf41 |
| hsa_circ_0091097 | 0.007072763 | 0.240764579 | down | 2853 | 577 | NM_000489 | ATRX |
| hsa_circ_0029155 | 0.024689987 | 0.377802838 | down | 4527 | 1503 | NM_003959 | HIP1R |
| hsa_circ_0070757 | 0.0058634 | 0.280457464 | down | 699 | 404 | NM_014822 | SEC24D |
| hsa_circ_0072231 | 0.044269458 | 0.345145378 | down | 65117 | 3498 | NM_153485 | NUP155 |
| hsa_circ_0075955 | 0.025728902 | 0.380137942 | down | 3518 | 3518 | --- | --- |
| hsa_circ_0053627 | 0.003674035 | 13.53934967 | up | 169258 | 10288 | NM_016252 | BIRC6 |
| hsa_circ_0023092 | 0.0142702 | 0.263161383 | down | 848 | 449 | NM_003952 | RPS6KB2 |
| hsa_circ_0044775 | 0.044730498 | 0.377454559 | down | 1356 | 1356 | NM_017763 | RNF43 |
| hsa_circ_0036521 | 0.023748725 | 0.485930485 | down | 709 | 709 | NM_021214 | FAM108C1 |
| hsa_circ_0084060 | 0.016793806 | 0.392156866 | down | 41582 | 3611 | NM_003012 | SFRP1 |
| hsa_circ_0055848 | 0.033688245 | 0.41048487 | down | 153 | 153 | NM_016316 | REV1 |
| hsa_circ_0051856 | 0.000284834 | 0.148342478 | down | 134 | 134 | NM_002152 | HRC |
| hsa_circ_0086084 | 0.044674349 | 0.37800896 | down | 5574 | 2588 | NM_013291 | CPSF1 |
| hsa_circ_0032158 | 0.023578485 | 0.283381048 | down | 8312 | 449 | NM_182914 | SYNE2 |
| hsa_circ_0049153 | 0.034440335 | 0.424113127 | down | 1492 | 240 | NM_152289 | ZNF561 |
| hsa_circ_0023873 | 0.017748569 | 0.232326884 | down | 1213 | 147 | NR_028026 | CREBZF |
| hsa_circ_0010548 | 0.020876067 | 2.440899726 | up | 27390 | 5874 | NM_005529 | HSPG2 |
| hsa_circ_0058404 | 0.000207746 | 0.408739641 | down | 3083 | 1395 | NM_015311 | OBSL1 |
| hsa_circ_0027120 | 0.000471822 | 0.328880484 | down | 3155 | 532 | NM_001178078 | STAT6 |
| hsa_circ_0023105 | 0.035514779 | 0.323927627 | down | 190 | 190 | NM_004910 | PITPNM1 |
| hsa_circ_0070230 | 0.016869075 | 0.276137245 | down | 72606 | 4256 | NM_014933 | SEC31A |
| hsa_circ_0086996 | 0.003022355 | 0.266716788 | down | 45708 | 15491 | NM_021111 | RECK |
| hsa_circ_0064242 | 0.024224538 | 0.34521438 | down | 7588 | 437 | NM_033084 | FANCD2 |
| hsa_circ_0092217 | 0.014442792 | 0.224447564 | down | 26888 | 996 | NM_002414 | CD99 |
| hsa_circ_0022972 | 5.15834E-06 | 0.165114608 | down | 664 | 250 | NM_020470 | YIF1A |
| hsa_circ_0086560 | 0.010776502 | 0.289902772 | down | 7879 | 211 | NM_017794 | KIAA1797 |
| hsa_circ_0029020 | 0.017160001 | 0.414446147 | down | 110 | 110 | NR_002809 | LOC338799 |
| hsa_circ_0044394 | 0.004785789 | 6.858345414 | up | 5383 | 799 | NM_006546 | IGF2BP1 |
| hsa_circ_0048806 | 0.015734876 | 0.337642819 | down | 4024 | 485 | NM_000635 | RFX2 |
| hsa_circ_0035092 | 0.021851649 | 0.281060028 | down | 4550 | 914 | NM_025137 | SPG11 |
| hsa_circ_0086645 | 0.026467935 | 0.304996071 | down | 8022 | 530 | NM_002504 | NFX1 |
| hsa_circ_0081543 | 0.00228701 | 0.398282458 | down | 5347 | 1345 | NM_003302 | TRIP6 |
| hsa_circ_0030346 | 0.049534756 | 0.357331957 | down | 18872 | 744 | NM_016075 | VPS36 |
| hsa_circ_0051784 | 0.026599551 | 0.333592653 | down | 292 | 174 | NM_020904 | PLEKHA4 |
| hsa_circ_0084344 | 0.032798809 | 0.48813607 | down | 43878 | 3401 | NM_006904 | PRKDC |
| hsa_circ_0034230 | 0.007618443 | 0.333862183 | down | 13755 | 1086 | NM_004667 | HERC2 |
| hsa_circ_0053836 | 0.000148617 | 0.140194055 | down | 3957 | 1055 | NM_016252 | BIRC6 |
| hsa_circ_0010639 | 0.037688345 | 0.278332744 | down | 13102 | 3280 | NM_005529 | HSPG2 |
| hsa_circ_0022343 | 0.021993601 | 0.347760869 | down | 9031 | 4391 | NM_001252065 | SYT7 |
| hsa_circ_0013466 | 0.024763075 | 0.406971725 | down | 540 | 540 | NM_000561 | GSTM1 |
| hsa_circ_0057989 | 0.000326949 | 0.326022601 | down | 12718 | 1232 | NM_015040 | PIKFYVE |
| hsa_circ_0009900 | 0.001606691 | 0.226580958 | down | 6011 | 378 | NM_000302 | PLOD1 |
| hsa_circ_0051410 | 0.009473962 | 0.491796647 | down | 4612 | 868 | NM_002856 | PVRL2 |
| hsa_circ_0084099 | 0.014472747 | 0.31991993 | down | 57132 | 2279 | NR_033818 | IKBKB |
| hsa_circ_0000166 | 0.028471769 | 0.254764021 | down | 12995 | 366 | NM_001164245 | C1orf27 |
| hsa_circ_0048924 | 0.03784685 | 0.338156341 | down | 122 | 122 | NM_004240 | TRIP10 |
| hsa_circ_0038055 | 0.043957374 | 0.484319612 | down | 26588 | 746 | NM_001143979 | NDE1 |
| hsa_circ_0052002 | 0.016804911 | 0.256375455 | down | 5735 | 288 | NM_001145809 | MYH14 |
| hsa_circ_0083580 | 0.045214651 | 2.075109123 | up | 5433 | 2716 | NM_022749 | FAM160B2 |
| hsa_circ_0064066 | 0.03177499 | 0.241700769 | down | 120048 | 4140 | NM_001168272 | ITPR1 |
| hsa_circ_0006789 | 0.009081194 | 0.261215792 | down | 173 | 173 | NM_145305 | SLC25A43 |
| hsa_circ_0063652 | 0.049905328 | 0.312027517 | down | 96 | 96 | NM_032311 | POLDIP3 |
| hsa_circ_0072666 | 0.001416974 | 0.320762594 | down | 94449 | 1335 | NM_197941 | ADAMTS6 |
| hsa_circ_0011045 | 0.032736461 | 2.580980607 | up | 1040 | 1040 | NM_001013642 | TRNP1 |
| hsa_circ_0050719 | 0.032873001 | 0.475854559 | down | 570 | 232 | NM_001083961 | WDR62 |
| hsa_circ_0079542 | 0.037052429 | 0.444769955 | down | 166 | 166 | NM_002214 | ITGB8 |
| hsa_circ_0020641 | 0.047115093 | 2.550457447 | up | 823 | 534 | NM_001135054 | SIGIRR |
| hsa_circ_0019303 | 0.026795276 | 0.379511528 | down | 7588 | 346 | NM_152309 | PIK3AP1 |
| hsa_circ_0028426 | 0.001376805 | 0.183912804 | down | 59596 | 5766 | NM_002834 | PTPN11 |
| hsa_circ_0002928 | 0.002750344 | 0.184980783 | down | 1528 | 462 | NM_001039619 | PRMT5 |
| hsa_circ_0087885 | 0.042059257 | 3.555256859 | up | 76021 | 7049 | NM_032012 | C9orf5 |
| hsa_circ_0091709 | 0.039996902 | 0.318983404 | down | 9120 | 590 | NM_004961 | GABRE |
| hsa_circ_0058363 | 0.000662054 | 0.394392403 | down | 149 | 149 | NM_006000 | TUBA4A |
| hsa_circ_0017110 | 0.007139077 | 0.351256866 | down | 252 | 252 | NM_002508 | NID1 |
| hsa_circ_0041859 | 0.02589531 | 0.377616491 | down | 221 | 221 | NM_000937 | POLR2A |
| hsa_circ_0046992 | 0.015308141 | 0.290479496 | down | 16385 | 2847 | NM_031216 | SEH1L |
| hsa_circ_0078457 | 0.000193671 | 0.304406416 | down | 139609 | 2216 | NM_020245 | TULP4 |
| hsa_circ_0022032 | 0.023722833 | 0.371240579 | down | 10099 | 546 | NM_014342 | MTCH2 |
| hsa_circ_0031232 | 0.03642974 | 0.398739044 | down | 70 | 70 | NM_001077351 | RBM23 |
| hsa_circ_0042582 | 0.004000735 | 0.266643395 | down | 11671 | 1722 | NM_014680 | KIAA0100 |
| hsa_circ_0037600 | 0.010048614 | 0.303346575 | down | 116 | 116 | NM_001083601 | NAA60 |
| hsa_circ_0054259 | 0.015129002 | 0.288316087 | down | 39148 | 289 | NM_020744 | MTA3 |
| hsa_circ_0014552 | 0.018295931 | 0.29838885 | down | 27670 | 1961 | NM_018489 | ASH1L |
| hsa_circ_0053981 | 0.02783986 | 0.301563677 | down | 12538 | 305 | NM_016441 | CRIM1 |
| hsa_circ_0062047 | 0.007198022 | 0.478324954 | down | 1659 | 1659 | NM_001848 | COL6A1 |
| hsa_circ_0017532 | 0.022588449 | 0.429367299 | down | 11040 | 944 | NM_001353 | AKR1C1 |
| hsa_circ_0057594 | 0.001690503 | 4.632699075 | up | 272273 | 1168 | NM_020760 | HECW2 |
| hsa_circ_0031340 | 0.013990921 | 0.40534338 | down | 1043 | 566 | NM_017999 | RNF31 |
| hsa_circ_0049326 | 0.009794992 | 3.144428723 | up | 17479 | 527 | NM_001005360 | DNM2 |
| hsa_circ_0027939 | 0.017601086 | 0.370506227 | down | 6094 | 1367 | NM_003299 | HSP90B1 |
| hsa_circ_0026579 | 0.044678151 | 0.385038325 | down | 24620 | 6451 | NM_012291 | ESPL1 |
| hsa_circ_0043296 | 0.017229102 | 0.419376044 | down | 3092 | 2131 | NM_007247 | SYNRG |
| hsa_circ_0022829 | 2.78901E-05 | 0.296126644 | down | 13789 | 2756 | NM_031904 | FRMD8 |
| hsa_circ_0046899 | 0.034867404 | 0.380044417 | down | 72 | 72 | NM_006868 | RAB31 |
| hsa_circ_0084960 | 0.006719806 | 0.409931756 | down | 26722 | 1584 | NM_152416 | C8orf38 |
| hsa_circ_0005783 | 0.004617553 | 0.291233875 | down | 547 | 547 | NM_007249 | KLF12 |
| hsa_circ_0055003 | 0.042543228 | 0.349758196 | down | 146 | 146 | NM_002398 | MEIS1 |
| hsa_circ_0010958 | 0.006830973 | 0.356083925 | down | 110 | 110 | NM_019557 | FAM54B |
| hsa_circ_0028489 | 0.04899007 | 0.3425479 | down | 11980 | 651 | NM_001143819 | TPCN1 |
| hsa_circ_0040059 | 0.009052823 | 0.276743429 | down | 11003 | 2495 | NM_032382 | COG8 |
| hsa_circ_0049725 | 0.039768873 | 2.061492581 | up | 4837 | 904 | NM_001008701 | LPHN1 |
| hsa_circ_0039324 | 0.000808217 | 0.460208521 | down | 14159 | 370 | NM_182922 | HEATR3 |
| hsa_circ_0087362 | 0.002335861 | 0.37655065 | down | 585 | 585 | NM_032307 | C9orf64 |
| hsa_circ_0000147 | 0.000721988 | 0.217458164 | down | 17668 | 735 | NM_012474 | UCK2 |
| hsa_circ_0040384 | 0.026177722 | 0.408895223 | down | 27971 | 986 | NM_001030007 | AP1G1 |
| hsa_circ_0086438 | 7.86969E-05 | 10.89626217 | up | 119717 | 2540 | NM_001040272 | ADAMTSL1 |
| hsa_circ_0086024 | 0.026014021 | 0.368599801 | down | 220 | 151 | NM_015201 | BOP1 |
| hsa_circ_0074920 | 0.004467733 | 2.321340852 | up | 24019 | 202 | NM_003062 | SLIT3 |
| hsa_circ_0063003 | 0.011511373 | 3.839013994 | up | 457331 | 1812 | NM_004737 | LARGE |
| hsa_circ_0073047 | 0.011807595 | 0.462834106 | down | 77233 | 1386 | NM_001130105 | COL4A3BP |
| hsa_circ_0023698 | 0.041429523 | 156.0511162 | up | 137 | 137 | NM_001293 | CLNS1A |
| hsa_circ_0006727 | 0.034614206 | 0.338210354 | down | 16165 | 16165 | NM_145305 | SLC25A43 |
| hsa_circ_0014524 | 0.020967393 | 0.243994864 | down | 2428 | 475 | NM_003993 | CLK2 |
| hsa_circ_0028801 | 0.028165253 | 0.330091819 | down | 22585 | 2972 | NM_006836 | GCN1L1 |
| hsa_circ_0080909 | 0.03401719 | 0.458614418 | down | 18135 | 435 | NM_006379 | SEMA3C |
| hsa_circ_0056431 | 0.000615995 | 0.229182668 | down | 17561 | 1419 | NR_027671 | UGGT1 |
| hsa_circ_0082679 | 0.007455581 | 0.295689236 | down | 100904 | 1328 | NM_022740 | HIPK2 |
| hsa_circ_0031290 | 0.013468977 | 0.388069044 | down | 3950 | 933 | NM_016609 | SLC22A17 |
| hsa_circ_0046350 | 0.026929474 | 0.379509301 | down | 3628 | 2053 | NM_004104 | FASN |
| hsa_circ_0023523 | 0.043302213 | 0.407081171 | down | 451 | 451 | NM_003355 | UCP2 |
| hsa_circ_0050200 | 0.024581556 | 0.418605198 | down | 657 | 386 | NM_017814 | TMEM161A |
| hsa_circ_0000948 | 0.045275863 | 0.356369705 | down | 2121 | 2121 | NM_006184 | NUCB1 |
| hsa_circ_0091808 | 0.025939007 | 0.373403167 | down | 23812 | 8426 | NM_005334 | HCFC1 |
| hsa_circ_0056147 | 0.014345953 | 0.358286027 | down | 9450 | 913 | NM_019014 | POLR1B |
| hsa_circ_0045006 | 0.004568446 | 0.37436646 | down | 9645 | 396 | NM_001099432 | BCAS3 |
| hsa_circ_0026655 | 0.033071254 | 0.374852623 | down | 16403 | 2230 | NM_005016 | PCBP2 |
| hsa_circ_0063162 | 0.025537475 | 0.421712787 | down | 171 | 171 | NM_024955 | FOXRED2 |
| hsa_circ_0070759 | 0.044096595 | 0.443212906 | down | 9118 | 289 | NM_014822 | SEC24D |
| hsa_circ_0008077 | 0.032681413 | 0.28926613 | down | 39402 | 303 | NM_001173489 | PRRG1 |
| hsa_circ_0010585 | 0.018677317 | 0.438918548 | down | 20275 | 4482 | NM_005529 | HSPG2 |
| hsa_circ_0041153 | 0.008680214 | 0.260401459 | down | 8160 | 387 | NM_006987 | RPH3AL |
| hsa_circ_0060500 | 0.003517744 | 0.279070563 | down | 4808 | 926 | NM_000022 | ADA |
| hsa_circ_0069917 | 0.022638381 | 0.234814521 | down | 7604 | 897 | NM_002092 | GRSF1 |
| hsa_circ_0014772 | 0.016641868 | 0.468623938 | down | 8634 | 5557 | NM_006617 | NES |
| hsa_circ_0016257 | 0.024125966 | 0.41696686 | down | 9196 | 701 | NM_006893 | EIF2D |
| hsa_circ_0089155 | 0.023952142 | 0.446160142 | down | 23407 | 1677 | NM_005085 | NUP214 |
| hsa_circ_0083280 | 0.007786641 | 0.150772101 | down | 66112 | 2022 | NM_024596 | MCPH1 |
| hsa_circ_0050960 | 0.009622648 | 0.28342721 | down | 392 | 157 | NM_001145901 | SARS2 |
| hsa_circ_0013205 | 0.048290815 | 0.386666214 | down | 16671 | 3208 | NM_001938 | DR1 |
| hsa_circ_0038032 | 0.020737124 | 0.361323745 | down | 6882 | 6882 | --- | --- |
| hsa_circ_0027057 | 0.005240149 | 0.232210149 | down | 12593 | 3341 | NM_013449 | BAZ2A |
| hsa_circ_0057499 | 0.04694524 | 0.485230111 | down | 27850 | 3637 | NM_001142645 | TMEM194B |
| hsa_circ_0059768 | 0.042602029 | 0.420774683 | down | 7893 | 482 | NM_014742 | TM9SF4 |
| hsa_circ_0059210 | 0.036288888 | 0.329450554 | down | 5364 | 1503 | NM_174856 | IDH3B |
| hsa_circ_0039015 | 0.029447773 | 0.295231115 | down | 949 | 872 | NM_001031827 | BOLA2 |
| hsa_circ_0026997 | 0.035644065 | 0.393066081 | down | 14206 | 4062 | NM_005419 | STAT2 |
| hsa_circ_0072442 | 0.022026019 | 0.314521183 | down | 475 | 475 | NM_015946 | PELO |
| hsa_circ_0026582 | 0.017831272 | 0.285350468 | down | 20092 | 4221 | NM_012291 | ESPL1 |
| hsa_circ_0051679 | 0.008676726 | 0.381590593 | down | 103 | 103 | NM_014681 | DHX34 |
| hsa_circ_0021760 | 0.015699116 | 0.192552421 | down | 38075 | 521 | NM_138787 | C11orf74 |
| hsa_circ_0071029 | 0.038523954 | 0.408238283 | down | 190175 | 1521 | NM_003866 | INPP4B |
| hsa_circ_0067475 | 0.019334262 | 0.219753959 | down | 104031 | 1601 | NM_005862 | STAG1 |
| hsa_circ_0002456 | 0.030187167 | 0.442658552 | down | 2291 | 224 | NM_001380 | DOCK1 |
| hsa_circ_0031474 | 0.005911526 | 0.304351667 | down | 123 | 123 | NM_015382 | HECTD1 |
| hsa_circ_0053980 | 0.007980225 | 0.217675056 | down | 122 | 122 | NM_016441 | CRIM1 |
| hsa_circ_0084976 | 0.025934573 | 0.494160874 | down | 9929 | 473 | NM_014754 | PTDSS1 |
| hsa_circ_0091992 | 0.027372294 | 0.392070594 | down | 4970 | 1957 | NM_001110556 | FLNA |
| hsa_circ_0037446 | 0.04039823 | 0.316677797 | down | 22231 | 10284 | NM_001009944 | PKD1 |
| hsa_circ_0050144 | 0.00120218 | 0.464123803 | down | 7763 | 381 | NM_001098482 | CRTC1 |
| hsa_circ_0091266 | 0.012980419 | 5.342730775 | up | 19052 | 403 | NR_038988 | LOC100287765 |
| hsa_circ_0018729 | 0.00783325 | 0.437075993 | down | 7829 | 6640 | NM_004273 | CHST3 |
| hsa_circ_0022613 | 0.007593528 | 0.447338493 | down | 22506 | 4516 | NM_017490 | MARK2 |
| hsa_circ_0088810 | 0.001129573 | 0.223650731 | down | 16548 | 1030 | NM_001003722 | GLE1 |
| hsa_circ_0071674 | 0.013244522 | 0.348610872 | down | 5082 | 417 | NR_027633 | BRD9 |
| hsa_circ_0078145 | 0.020806472 | 0.422701108 | down | 257 | 257 | NM_001042683 | SHPRH |
| hsa_circ_0091624 | 0.041968877 | 0.401540731 | down | 11751 | 3495 | NM_173470 | MMGT1 |
| hsa_circ_0074646 | 0.000337684 | 0.397270794 | down | 2318 | 255 | NM_003118 | SPARC |
| hsa_circ_0076974 | 0.026752056 | 0.374688088 | down | 12112 | 1253 | NM_138441 | MB21D1 |
| hsa_circ_0001405 | 0.040946026 | 0.391550038 | down | 4072 | 385 | NM_001100399 | PDS5A |
| hsa_circ_0078994 | 0.022494433 | 2.235478117 | up | 3995 | 3995 | --- | --- |
| hsa_circ_0088444 | 0.023428941 | 0.384937883 | down | 51912 | 274 | NM_020946 | DENND1A |
| hsa_circ_0045839 | 0.008067612 | 0.297928277 | down | 34573 | 925 | NM_001242532 | MFSD11 |
| hsa_circ_0039861 | 0.004264109 | 0.431744121 | down | 68640 | 707 | NM_020850 | RANBP10 |
| hsa_circ_0049325 | 0.019213 | 0.286202791 | down | 16169 | 428 | NM_001005360 | DNM2 |
| hsa_circ_0070309 | 0.045228207 | 0.476230236 | down | 46108 | 2673 | NM_014991 | WDFY3 |
| hsa_circ_0051045 | 0.041849122 | 0.273953851 | down | 133 | 133 | NM_001436 | FBL |
| hsa_circ_0037423 | 0.01627274 | 0.395489357 | down | 8741 | 1415 | NM_000548 | TSC2 |
| hsa_circ_0058141 | 0.001920488 | 13.09908403 | up | 26135 | 2388 | NM_212482 | FN1 |
| hsa_circ_0016069 | 0.010388227 | 0.382999063 | down | 81318 | 2454 | NM_001001396 | ATP2B4 |
| hsa_circ_0057760 | 0.034913019 | 0.300742206 | down | 10096 | 678 | NM_015049 | TRAK2 |
| hsa_circ_0068492 | 0.020228228 | 0.353316764 | down | 23514 | 410 | NM_001879 | MASP1 |
| hsa_circ_0024703 | 0.048964109 | 0.378461695 | down | 187 | 187 | NM_032873 | UBASH3B |
| hsa_circ_0027942 | 0.000820219 | 2.298770002 | up | 118 | 118 | NM_001135570 | C12orf73 |
| hsa_circ_0070111 | 0.006732677 | 0.27850043 | down | 15041 | 672 | NM_025074 | FRAS1 |
| hsa_circ_0011692 | 0.032834279 | 0.270068051 | down | 584 | 230 | NM_032017 | STK40 |
| hsa_circ_0051424 | 0.032681385 | 0.363959021 | down | 1408 | 805 | NM_001294 | CLPTM1 |
| hsa_circ_0023091 | 0.049104698 | 0.387943396 | down | 617 | 341 | NM_003952 | RPS6KB2 |
| hsa_circ_0062948 | 0.001825672 | 0.421052257 | down | 4692 | 342 | NM_001242896 | DEPDC5 |
| hsa_circ_0071728 | 0.022507496 | 0.281604706 | down | 27647 | 1044 | NM_024830 | LPCAT1 |
| hsa_circ_0055086 | 0.04860428 | 0.42060195 | down | 46147 | 324 | NM_001153 | ANXA4 |
| hsa_circ_0087771 | 0.046986266 | 0.31044998 | down | 4797 | 267 | NM_017746 | TEX10 |
| hsa_circ_0053983 | 0.039585275 | 0.396325963 | down | 47861 | 911 | NM_016441 | CRIM1 |
| hsa_circ_0071408 | 0.007708324 | 0.353049315 | down | 2896 | 463 | NM_001166108 | PALLD |
| hsa_circ_0040608 | 0.000508657 | 0.289051254 | down | 172352 | 4141 | NM_002661 | PLCG2 |
| hsa_circ_0012692 | 0.0026752 | 0.37434937 | down | 120 | 120 | NM_015306 | USP24 |
| hsa_circ_0013796 | 0.000675333 | 0.468240853 | down | 336 | 336 | NM_024408 | NOTCH2 |
| hsa_circ_0085231 | 0.00890658 | 0.29073996 | down | 9519 | 1511 | NM_015902 | UBR5 |
| hsa_circ_0070720 | 0.00458115 | 0.383057739 | down | 64567 | 312 | NM_022569 | NDST4 |
| hsa_circ_0019419 | 0.0374321 | 0.255417318 | down | 18430 | 540 | NM_020354 | ENTPD7 |
| hsa_circ_0004595 | 0.026784063 | 0.298348778 | down | 5493 | 502 | NM_003272 | GPR137B |
| hsa_circ_0030715 | 0.014226555 | 0.372523597 | down | 122 | 122 | NM_001130048 | DOCK9 |
| hsa_circ_0059617 | 0.026582123 | 0.400810829 | down | 4281 | 775 | NM_000099 | CST3 |
| hsa_circ_0088852 | 0.019664628 | 0.40879651 | down | 11766 | 1362 | NM_001130438 | SPTAN1 |
| hsa_circ_0023834 | 0.006226627 | 0.446137932 | down | 25419 | 582 | NM_001098816 | ODZ4 |
| hsa_circ_0082484 | 0.013512007 | 0.380324393 | down | 1989 | 365 | NM_014149 | WDR91 |
| hsa_circ_0060459 | 0.005067606 | 0.359441183 | down | 10747 | 865 | NM_002466 | MYBL2 |
| hsa_circ_0080772 | 0.018399043 | 0.307185609 | down | 152 | 152 | NM_005918 | MDH2 |
| hsa_circ_0028294 | 0.03039254 | 0.316797595 | down | 182 | 182 | NM_001136538 | ACAD10 |
| hsa_circ_0021386 | 0.005643897 | 0.366533435 | down | 7498 | 360 | NM_002645 | PIK3C2A |
| hsa_circ_0037234 | 0.033294059 | 0.402856764 | down | 809 | 649 | NM_003961 | RHBDL1 |
| hsa_circ_0070320 | 0.027775211 | 2.493769562 | up | 20358 | 1569 | NM_014991 | WDFY3 |
| hsa_circ_0045962 | 0.010306055 | 0.310371292 | down | 68 | 68 | NM_001042573 | ENGASE |
| hsa_circ_0012586 | 0.020530071 | 0.302427632 | down | 1828 | 461 | NM_001199080 | PODN |
| hsa_circ_0006241 | 0.001708263 | 0.436446828 | down | 36370 | 300 | NM_001080463 | DYNC2H1 |
| hsa_circ_0016402 | 0.001679818 | 0.237865091 | down | 5652 | 148 | NM_001146171 | TATDN3 |
| hsa_circ_0067048 | 0.009337063 | 0.39520287 | down | 87 | 87 | NM_053025 | MYLK |
| hsa_circ_0016961 | 0.01608061 | 0.323945199 | down | 39982 | 654 | NM_014801 | PCNXL2 |
| hsa_circ_0024135 | 0.046484915 | 0.373216137 | down | 114666 | 2337 | NM_001080463 | DYNC2H1 |
| hsa_circ_0082217 | 0.004205107 | 0.270405892 | down | 10714 | 4349 | NM_001458 | FLNC |
| hsa_circ_0068347 | 0.001919942 | 0.153802945 | down | 1133 | 307 | NM_182917 | EIF4G1 |
| hsa_circ_0046112 | 0.014944457 | 0.282611437 | down | 31462 | 2330 | NM_138570 | SLC38A10 |
| hsa_circ_0009233 | 0.005400814 | 0.291080508 | down | 5419 | 2740 | NM_198576 | AGRN |
| hsa_circ_0067161 | 0.001455775 | 0.211936196 | down | 3256 | 373 | NM_016372 | TPRA1 |
| hsa_circ_0052271 | 0.003976603 | 0.43005833 | down | 10451 | 820 | NM_001130072 | EPN1 |
| hsa_circ_0079110 | 0.031409687 | 2.299305688 | up | 2561 | 518 | NM_001080453 | INTS1 |
| hsa_circ_0033636 | 0.034095531 | 0.476756827 | down | 1098 | 1098 | --- | --- |
| hsa_circ_0071345 | 0.00861106 | 0.367325015 | down | 39063 | 2711 | NM_014247 | RAPGEF2 |
| hsa_circ_0070410 | 0.045164178 | 0.413390294 | down | 101 | 101 | NM_152542 | PPM1K |
| hsa_circ_0005762 | 0.042809247 | 0.26833371 | down | 8337 | 309 | NM_015910 | WDPCP |
| hsa_circ_0035746 | 0.044039224 | 0.38578686 | down | 172976 | 9371 | NM_003922 | HERC1 |
| hsa_circ_0032765 | 0.020281853 | 0.301434202 | down | 109 | 109 | NM_013382 | POMT2 |
| hsa_circ_0081508 | 0.006592259 | 0.222159449 | down | 7900 | 4947 | NM_022574 | GIGYF1 |
| hsa_circ_0010617 | 0.024079804 | 0.292689856 | down | 14116 | 3135 | NM_005529 | HSPG2 |
| hsa_circ_0052255 | 0.014708526 | 0.49044388 | down | 1064 | 1064 | NM_153219 | ZNF524 |
| hsa_circ_0023524 | 0.040453324 | 0.491642994 | down | 1001 | 632 | NM_003355 | UCP2 |
| hsa_circ_0088570 | 0.009558905 | 0.236476585 | down | 27700 | 2064 | NM_015635 | GAPVD1 |
| hsa_circ_0074377 | 0.017999567 | 0.382433086 | down | 13415 | 290 | NM_015071 | ARHGAP26 |
| hsa_circ_0046377 | 0.015477812 | 2.170097688 | up | 906 | 322 | NM_198082 | CCDC57 |
| hsa_circ_0058854 | 0.011132199 | 0.345692723 | down | 71 | 71 | NM_080678 | UBE2F |
| hsa_circ_0078410 | 0.002686541 | 0.486050173 | down | 67743 | 4295 | NM_020732 | ARID1B |
| hsa_circ_0000181 | 0.003833053 | 0.313358486 | down | 3529 | 279 | NM_001146171 | TATDN3 |
| hsa_circ_0034202 | 0.004553933 | 0.238534539 | down | 88967 | 6435 | NM_004667 | HERC2 |
| hsa_circ_0025157 | 0.038317289 | 0.477788243 | down | 5982 | 1029 | NM_014865 | NCAPD2 |
| hsa_circ_0088862 | 0.01995408 | 0.385136936 | down | 32405 | 3470 | NM_001130438 | SPTAN1 |
| hsa_circ_0012073 | 0.018680699 | 2.416908406 | up | 19411 | 570 | NM_002840 | PTPRF |
| hsa_circ_0055207 | 0.030379985 | 2.485385095 | up | 6638 | 6370 | NM_015470 | RAB11FIP5 |
| hsa_circ_0090430 | 0.008502846 | 0.332013371 | down | 7052 | 2315 | NM_004651 | USP11 |
| hsa_circ_0004377 | 0.019052521 | 0.494507803 | down | 4967 | 380 | NM_032802 | SPPL2A |
| hsa_circ_0019484 | 0.015762186 | 0.381499372 | down | 18475 | 1828 | NM_015221 | DNMBP |
| hsa_circ_0060815 | 0.018052387 | 0.250731657 | down | 144 | 144 | NM_015266 | SLC9A8 |
| hsa_circ_0074669 | 0.007433734 | 0.20986876 | down | 13775 | 1133 | NM_005754 | G3BP1 |
| hsa_circ_0053198 | 0.032124523 | 0.379305372 | down | 6616 | 960 | NM_004341 | CAD |
| hsa_circ_0055318 | 0.0301783 | 0.350943243 | down | 681 | 424 | NM_032118 | WDR54 |
| hsa_circ_0090691 | 0.048001061 | 0.29643091 | down | 59806 | 8552 | NM_031407 | HUWE1 |
| hsa_circ_0081506 | 0.014928031 | 0.258619081 | down | 6927 | 4626 | NM_022574 | GIGYF1 |
| hsa_circ_0069237 | 0.025923055 | 0.36054374 | down | 45055 | 511 | NM_001145848 | PROM1 |
| hsa_circ_0075943 | 0.007668377 | 0.241304705 | down | 10940 | 10940 | --- | --- |
| hsa_circ_0051699 | 0.009835311 | 0.219588589 | down | 17323 | 2832 | NM_014601 | EHD2 |
| hsa_circ_0058977 | 0.01662871 | 0.253568651 | down | 3174 | 342 | NM_001080437 | SNED1 |
| hsa_circ_0055507 | 0.000273095 | 0.192392345 | down | 1032 | 177 | NM_017952 | PTCD3 |
| hsa_circ_0004357 | 0.000815398 | 0.35533238 | down | 2596 | 300 | NM_032591 | SLC9A7 |
| hsa_circ_0007288 | 3.94621E-05 | 0.125622253 | down | 25273 | 758 | NM_005486 | TOM1L1 |
| hsa_circ_0071399 | 1.08304E-05 | 0.272575778 | down | 35095 | 1949 | NM_001012967 | DDX60L |
| hsa_circ_0088877 | 0.000118254 | 0.325915104 | down | 7897 | 1696 | NM_001130438 | SPTAN1 |
| hsa_circ_0074276 | 0.034625585 | 0.402457077 | down | 1746 | 593 | NM_020690 | ANKHD1-EIF4EBP3 |
| hsa_circ_0036741 | 0.039768946 | 0.358785445 | down | 3879 | 450 | NM_001150 | ANPEP |
| hsa_circ_0039416 | 0.013553737 | 0.490559733 | down | 30090 | 1316 | NM_001172501 | SLC6A2 |
| hsa_circ_0091331 | 0.022498171 | 0.432877086 | down | 41800 | 117 | NM_032227 | TMEM164 |
| hsa_circ_0004517 | 0.034717825 | 0.314493609 | down | 5908 | 686 | NM_003704 | FAM193A |
| hsa_circ_0087783 | 0.043361016 | 0.378222025 | down | 18412 | 1620 | NM_019592 | RNF20 |
| hsa_circ_0014765 | 0.023303349 | 2.109917186 | up | 7292 | 853 | NM_178229 | IQGAP3 |
| hsa_circ_0079229 | 0.000680233 | 0.333962038 | down | 2729 | 498 | NM_001037165 | FOXK1 |
| hsa_circ_0079126 | 0.047504649 | 0.383573796 | down | 3058 | 811 | NM_001080453 | INTS1 |
| hsa_circ_0075743 | 0.027972592 | 2.006973211 | up | 12877 | 2639 | NM_001105568 | KIF13A |
| hsa_circ_0023180 | 0.023214844 | 0.440902914 | down | 10001 | 595 | NM_002335 | LRP5 |
| hsa_circ_0065376 | 0.000810214 | 0.197738488 | down | 6224 | 2274 | NM_002673 | PLXNB1 |
| hsa_circ_0016865 | 0.001310025 | 0.425394984 | down | 233 | 233 | NM_007357 | COG2 |
| hsa_circ_0078484 | 0.048636327 | 0.404612934 | down | 17928 | 2049 | NM_020823 | TMEM181 |
| hsa_circ_0063271 | 0.023395854 | 0.483080233 | down | 7011 | 745 | NM_033386 | MICALL1 |
| hsa_circ_0092203 | 0.025105338 | 0.244739254 | down | 39179 | 1903 | NM_004192 | ASMTL |
| hsa_circ_0010432 | 0.030558061 | 0.378167844 | down | 102 | 102 | NM_020816 | KIF17 |
| hsa_circ_0075387 | 0.03150809 | 0.38152884 | down | 1484 | 348 | NM_006098 | GNB2L1 |
| hsa_circ_0017828 | 0.032670733 | 0.364221042 | down | 13027 | 726 | NM_001033858 | DCLRE1C |
| hsa_circ_0072565 | 0.023622858 | 0.482433384 | down | 141785 | 163 | NM_001197220 | PDE4D |
| hsa_circ_0085140 | 0.021723257 | 0.301137857 | down | 17791 | 2416 | NM_002568 | PABPC1 |
| hsa_circ_0042530 | 0.008151451 | 0.330696049 | down | 6387 | 2077 | NM_015584 | POLDIP2 |
| hsa_circ_0066371 | 0.04137556 | 0.389915033 | down | 1354 | 357 | NM_001164317 | FLNB |
| hsa_circ_0044603 | 0.025001089 | 0.291994809 | down | 7410 | 1116 | NM_003786 | ABCC3 |
| hsa_circ_0051224 | 0.046945871 | 0.377112433 | down | 27217 | 1774 | NM_000709 | BCKDHA |
| hsa_circ_0036434 | 0.016287927 | 0.273316484 | down | 52569 | 2336 | NM_144572 | TBC1D2B |
| hsa_circ_0087559 | 0.011074137 | 0.312288887 | down | 8947 | 653 | NM_005392 | PHF2 |
| hsa_circ_0020224 | 0.045246416 | 0.381349587 | down | 42682 | 2765 | NM_018117 | WDR11 |
| hsa_circ_0079109 | 0.019910621 | 0.341787642 | down | 18837 | 4292 | NM_001080453 | INTS1 |
| hsa_circ_0091162 | 0.0141828 | 0.360328712 | down | 7243 | 2213 | NM_015975 | TAF9B |
| hsa_circ_0013932 | 0.022655599 | 0.456852479 | down | 10238 | 943 | NM_183372 | NBPF11 |
| hsa_circ_0060318 | 0.040673555 | 2.435638787 | up | 19756 | 1202 | NM_014657 | TTI1 |
| hsa_circ_0037218 | 0.005220706 | 0.484944751 | down | 5878 | 1713 | NM_145294 | WDR90 |
| hsa_circ_0081972 | 0.047561883 | 0.317734777 | down | 199464 | 3949 | NM_014705 | DOCK4 |
| hsa_circ_0079006 | 0.016217197 | 0.289301137 | down | 201 | 201 | NM_001161376 | C6orf136 |
| hsa_circ_0079704 | 0.003398018 | 0.263253087 | down | 10029 | 743 | NR_038889 | LOC401320 |
| hsa_circ_0013148 | 0.032800736 | 0.272034686 | down | 8790 | 738 | NM_053274 | GLMN |
| hsa_circ_0015893 | 0.006220786 | 0.314698564 | down | 366 | 366 | NM_020443 | NAV1 |
| hsa_circ_0010705 | 0.018470748 | 0.394491044 | down | 136 | 136 | NM_005529 | HSPG2 |
| hsa_circ_0034938 | 0.030476335 | 0.33187146 | down | 8339 | 706 | NM_024956 | TMEM62 |
| hsa_circ_0074966 | 0.032209341 | 0.409013288 | down | 13216 | 604 | NM_004946 | DOCK2 |
| hsa_circ_0088616 | 0.033148697 | 0.487206041 | down | 95 | 95 | NM_033446 | FAM125B |
| hsa_circ_0049682 | 0.000102607 | 0.190599385 | down | 83 | 83 | NM_030818 | CCDC130 |
| hsa_circ_0009116 | 0.015099572 | 0.274901576 | down | 1472 | 232 | NM_033386 | MICALL1 |
| hsa_circ_0025337 | 0.040836997 | 0.366660936 | down | 3538 | 1023 | NM_014718 | CLSTN3 |
| hsa_circ_0038319 | 0.043353998 | 0.327444354 | down | 620 | 620 | NM_016524 | SYT17 |
| hsa_circ_0049702 | 0.005956458 | 0.417888279 | down | 74 | 74 | NM_017721 | CC2D1A |
| hsa_circ_0083412 | 0.011649254 | 0.362787797 | down | 12345 | 2758 | NM_016353 | ZDHHC2 |
| hsa_circ_0092150 | 0.012846342 | 0.27418478 | down | 21210 | 1941 | NM_001166462 | MPP1 |
| hsa_circ_0036732 | 0.016950045 | 0.352183432 | down | 9728 | 296 | NM_152259 | C15orf42 |
| hsa_circ_0042500 | 0.007351452 | 0.170346189 | down | 1556 | 222 | NM_015626 | WSB1 |
| hsa_circ_0061095 | 0.002576339 | 0.417721822 | down | 779 | 439 | NM_005560 | LAMA5 |
| hsa_circ_0010102 | 0.04854953 | 0.414799633 | down | 9420 | 2160 | NM_017556 | FBLIM1 |
| hsa_circ_0042174 | 0.024674585 | 0.34578961 | down | 556 | 556 | NM_001190440 | NCOR1 |
| hsa_circ_0011352 | 0.039451628 | 0.274121491 | down | 953 | 243 | NM_012316 | KPNA6 |
| hsa_circ_0013896 | 0.021886395 | 0.490792812 | down | 3861 | 610 | NM_006099 | PIAS3 |
| hsa_circ_0089016 | 0.022636461 | 0.373433139 | down | 101 | 101 | NM_020438 | DOLPP1 |
| hsa_circ_0081500 | 0.010328047 | 0.455424618 | down | 3271 | 2908 | NM_022574 | GIGYF1 |
| hsa_circ_0022657 | 0.003658054 | 0.418353909 | down | 111 | 111 | NM_001160389 | TRPT1 |
| hsa_circ_0050918 | 0.014809218 | 0.319072323 | down | 135 | 135 | NM_004924 | ACTN4 |
| hsa_circ_0023107 | 0.020056643 | 0.414913135 | down | 891 | 170 | NM_004910 | PITPNM1 |
| hsa_circ_0083498 | 0.007562506 | 0.240712709 | down | 13860 | 1116 | NM_018142 | INTS10 |
| hsa_circ_0060960 | 0.019927456 | 0.291669066 | down | 800 | 800 | NM_016407 | C20orf43 |
| hsa_circ_0077013 | 0.019881467 | 0.381853266 | down | 34204 | 1165 | NM_012434 | SLC17A5 |
| hsa_circ_0050265 | 0.019777574 | 0.482026914 | down | 109 | 109 | NM_020410 | ATP13A1 |
| hsa_circ_0081600 | 0.04879337 | 0.335837985 | down | 11251 | 582 | NM_133457 | EMID2 |
| hsa_circ_0042080 | 0.004513521 | 0.332815147 | down | 38094 | 202 | NM_201433 | GAS7 |
| hsa_circ_0068573 | 0.000198609 | 0.456687073 | down | 141123 | 1566 | NM_012287 | ACAP2 |
| hsa_circ_0076339 | 0.049007074 | 0.369111736 | down | 3297 | 203 | NM_033502 | TRERF1 |
| hsa_circ_0001620 | 0.008472048 | 0.189157654 | down | 227 | 227 | NM_014611 | MDN1 |
| hsa_circ_0006517 | 0.01493411 | 0.405254945 | down | 40196 | 236 | NM_005578 | LPP |
| hsa_circ_0026652 | 0.013716614 | 0.328269955 | down | 6340 | 378 | NM_031989 | PCBP2 |
| hsa_circ_0028161 | 0.004894016 | 0.238583336 | down | 7427 | 570 | NM_057169 | GIT2 |
| hsa_circ_0066034 | 0.004217459 | 0.436753655 | down | 2490 | 842 | NM_020163 | SEMA3G |
| hsa_circ_0088568 | 0.013241656 | 0.297183273 | down | 217 | 217 | NM_015635 | GAPVD1 |
| hsa_circ_0042647 | 0.015386561 | 0.35911952 | down | 400 | 400 | NM_014680 | KIAA0100 |
| hsa_circ_0050863 | 0.003967579 | 0.467001973 | down | 8244 | 950 | NM_007181 | MAP4K1 |
| hsa_circ_0010631 | 0.042244233 | 0.287563452 | down | 16387 | 3583 | NM_005529 | HSPG2 |
| hsa_circ_0058476 | 0.007402576 | 0.428054611 | down | 7249 | 397 | NM_006216 | SERPINE2 |
| hsa_circ_0025501 | 0.006775655 | 0.474877487 | down | 5004 | 2413 | NM_004064 | CDKN1B |
| hsa_circ_0053221 | 0.002650124 | 7.356935418 | up | 589 | 466 | NM_004341 | CAD |
| hsa_circ_0076604 | 0.013703545 | 0.327623755 | down | 136 | 136 | NM_019096 | GTPBP2 |
| hsa_circ_0018655 | 0.031255787 | 0.339815532 | down | 24514 | 7531 | NM_004096 | EIF4EBP2 |
| hsa_circ_0055939 | 0.000101251 | 10.60890759 | up | 2710 | 182 | NM_025076 | UXS1 |
| hsa_circ_0026341 | 0.009552746 | 0.264487298 | down | 1009 | 474 | NM_000020 | ACVRL1 |
| hsa_circ_0003321 | 0.013713349 | 0.235632307 | down | 26037 | 381 | NM_138787 | C11orf74 |
| hsa_circ_0016151 | 0.00612114 | 0.446967658 | down | 3367 | 325 | NM_002646 | PIK3C2B |
| hsa_circ_0044783 | 0.002230726 | 0.44919502 | down | 22975 | 5626 | NM_004687 | MTMR4 |
| hsa_circ_0010541 | 0.014305098 | 0.188909179 | down | 29683 | 7378 | NM_005529 | HSPG2 |
| hsa_circ_0016150 | 9.80528E-05 | 0.356392331 | down | 2582 | 423 | NM_002646 | PIK3C2B |
| hsa_circ_0063994 | 0.002024096 | 0.339448385 | down | 3720 | 2220 | NM_033200 | LMF2 |
| hsa_circ_0088432 | 0.009573906 | 0.431116644 | down | 37090 | 221 | NM_020946 | DENND1A |
| hsa_circ_0074606 | 0.011172308 | 0.245467392 | down | 9141 | 659 | NM_001155 | ANXA6 |
| hsa_circ_0009063 | 0.004601949 | 0.315469235 | down | 4315 | 1686 | NM_152318 | C12orf45 |
| hsa_circ_0069257 | 0.000600267 | 0.259182083 | down | 25680 | 1665 | NM_015907 | LAP3 |
| hsa_circ_0013127 | 0.000269253 | 0.435945088 | down | 14223 | 1243 | NM_001134420 | CDC7 |
| hsa_circ_0084920 | 0.037139943 | 0.32902914 | down | 7384 | 428 | NM_015496 | KIAA1429 |
| hsa_circ_0058124 | 0.034433424 | 2.623571609 | up | 3502 | 864 | NM_212482 | FN1 |
| hsa_circ_0034471 | 0.042464789 | 0.341445494 | down | 7333 | 1741 | NM_003246 | THBS1 |
| hsa_circ_0091804 | 0.047943717 | 0.351344677 | down | 8855 | 5263 | NM_005334 | HCFC1 |
| hsa_circ_0009377 | 0.018816423 | 2.704674633 | up | 5152 | 372 | NM_007033 | RER1 |
| hsa_circ_0006276 | 0.014964487 | 0.260729519 | down | 4339 | 436 | NM_004034 | ANXA7 |
| hsa_circ_0067159 | 0.010360167 | 0.409507303 | down | 407 | 164 | NM_016372 | TPRA1 |
| hsa_circ_0087540 | 0.004685655 | 0.175507746 | down | 21344 | 212 | NM_014612 | FAM120A |
| hsa_circ_0017919 | 0.002759028 | 0.462030086 | down | 124 | 124 | --- | --- |
| hsa_circ_0021587 | 0.041240983 | 0.296603112 | down | 2558 | 1016 | NM_001326 | CSTF3 |
| hsa_circ_0056436 | 0.02194171 | 0.358609438 | down | 5477 | 705 | NR_027671 | UGGT1 |
| hsa_circ_0044688 | 0.006272813 | 0.200028888 | down | 36379 | 998 | NM_005486 | TOM1L1 |
| hsa_circ_0024985 | 0.002080978 | 0.427522071 | down | 77965 | 43029 | NM_134424 | RAD52 |
| hsa_circ_0082673 | 0.00990979 | 0.325899539 | down | 127988 | 2236 | NM_022740 | HIPK2 |
| hsa_circ_0006016 | 0.008007282 | 0.225423085 | down | 6955 | 331 | NM_015156 | RCOR1 |
| hsa_circ_0023695 | 0.031420929 | 0.418122652 | down | 18201 | 828 | NM_001293 | CLNS1A |
| hsa_circ_0020340 | 0.032963271 | 0.414576799 | down | 754 | 271 | NM_017580 | ZRANB1 |
| hsa_circ_0031665 | 0.003146071 | 0.22971803 | down | 129595 | 3181 | NM_014990 | RALGAPA1 |
| hsa_circ_0083128 | 0.008242662 | 0.358836967 | down | 7991 | 983 | NM_022458 | LMBR1 |
| hsa_circ_0063907 | 0.049415087 | 0.245494671 | down | 1948 | 1568 | NM_002751 | MAPK11 |
| hsa_circ_0090583 | 0.005863088 | 0.283242478 | down | 119056 | 626 | NM_001127899 | CLCN5 |
| hsa_circ_0037388 | 0.029454943 | 0.396576044 | down | 2357 | 1207 | NM_006453 | TBL3 |

Table S9. The expression difference of candidate circRNAs between patients with uterine myoma or other benign diseases. (Page 89)

|  |  | circ-0003972 | | circ-0007288 | |
| --- | --- | --- | --- | --- | --- |
|  | N (%) | Average±SD (2^-ΔCT^) | P | Average±SD (2^-ΔCT^) | P |
| Uterine myoma | 37 (61.7) | 0.792±1.509 | 0.621 | 0.024±0.063 | 0.897 |
| Others | 30 (38.3) | 1.597±3.467 |  | 0.012± 0.021 |  |

Table S10. Comparison of AUC in circ-0003972+CA125, circ-0007288+CA125, circCOMBO+CA125. (Page 90)

| Groups | P-value |
| --- | --- |
| circ-0003972+CA125 vs circ-0007288+CA125 | 0.8036 |
| circ-0007288+CA125 vs circCOMBO+CA125 | 0.792 |
| circCOMBO+CA125 vs circ-0003972+CA125 | 0.3076 |
